# Crown Ether–Peptide Rotaxanes

**DOI:** 10.1002/anie.202513115

**Published:** 2025-08-06

**Authors:** Peng‐Lai Wang, Peng Chen, Raorao Yang, Daniel J. Tetlow, Zhi‐Hui Zhang, Jing Han, Stephen D. P. Fielden, Prodip Howlader, Liang Zhang, David A. Leigh

**Affiliations:** ^1^ School of Chemistry and Molecular Engineering East China Normal University Shanghai 200062 P.R. China; ^2^ Department of Chemistry University of Manchester Oxford Road Manchester M13 9PL UK

**Keywords:** Active template synthesis, Lasso peptides, Mechanically interlocked molecules, Rotaxanes

## Abstract

We report on the metal‐free active template synthesis of crown ether–peptide rotaxanes. A 24‐crown‐8 ring is sufficiently small that the side chains of canonical branched amino acids act as barriers that trap the macrocycle on the particular glycine residue used to assemble the rotaxane. The resulting crown ether–tripeptide rotaxane can subsequently be extended from either or both N‐ and C‐termini of the axle. Three distinct positional isomers of a heptapeptide [2]rotaxane containing three glycine units were selectively synthesized, and in each case the unique position of the crown ether on the peptide axle was confirmed by ^1^H nuclear magnetic resonance spectroscopy and tandem mass spectrometry. The three positional isomers adopt different conformations in the region adjacent to the trapped macrocycle, and have different chemical stabilities and secondary interactions in comparison to the unthreaded peptide axle. The crown ether does not inhibit enzymatic proteolysis over the entire length of the heptapeptide–axle rotaxanes, but rather provides significant protection from degradation for the three to four residues local to the encapsulated region. The strategy opens a pathway to new analogs of naturally occurring mechanically interlocked peptides.

## Graphical abstract

The synthesis of crown ether‐peptide rotaxanes is reported, incorporating a broad range of canonical and unnatural peptides into a threaded peptide backbone without the need for additional nonamide template sites in the axle. The 24C8 macrocycle is locked on the glycine residue used to assemble the rotaxane, allowing the formation of different kinetically stable positional isomers.

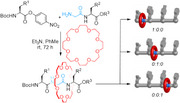

## Introduction

Naturally occurring mechanically interlocked peptides include a large family (>10 000 examples) of ribosomally and post‐translationally modified (RiPP) lasso peptides.^[^ ^1^, ^2^, ^3^, ^4^, ^5^, ^6^, ^7^, ^8^, ^9^, ^10^, ^11^, ^12^, ^13^, ^14^, ^15^ ^]^ The sheathing of the peptide strand can produce a range of attractive properties, including metabolic stability and a lack of immunogenicity, and a number of lasso peptides also exhibit antimicrobial, antiviral and/or enzyme‐inhibitory activity.^[^ ^1^, ^2^, ^3^, ^4^, ^5^, ^6^, ^7^, ^8^, ^9^, ^10^, ^11^, ^12^, ^13^, ^14^, ^15^ ^]^ However, the small ring size and tendency for cyclic peptide cavities to collapse through intramolecular hydrogen bonding makes designing synthetic intermediates that undergo threading difficult.^[^ ^16^, ^17^, ^18^ ^]^ Accordingly, few synthetic analogs of peptide rotaxanes have been made to date.^[^ ^19^, ^20^, ^21^, ^22^, ^23^, ^24^, ^25^, ^26^, ^27^ ^]^ However, metal‐free^[^ ^28^, ^29^ ^]^ active template^[^ ^30^, ^31^, ^32^, ^33^, ^34^, ^35^, ^36^, ^37^, ^38^ ^]^ synthesis has been shown to generate rotaxanes from crown ethers, primary amines, and a range of electrophiles.^[^ ^39^, ^40^, ^41^, ^42^, ^43^, ^44^ ^]^ Here, we demonstrate that metal‐free active template synthesis can be used to make crown ether–peptide rotaxanes using a macrocycle that is sufficiently small that, like the recently discovered lasso peptide antibiotic lariocidin^[^ ^10^, ^11^ ^]^ and the antibacterials triculamin,^[^ ^12^, ^13^ ^]^ paucinodin,^[^ ^14^ ^]^ and stlassin,^[^ ^15^ ^]^ the ring is locked in place by the side chains of the canonical amino acids on either side of a glycine residue on the peptide axle. The strategy adds to the growing number^[^ ^19^, ^20^, ^21^, ^22^, ^23^, ^24^, ^25^, ^26^, ^27^, ^45^, ^46^, ^47^, ^48^, ^49^, ^50^, ^51^, ^52^, ^53^ ^]^ of synthetic catenanes, cages and knots containing peptide components.

The methodology involves the condensation of the amine of an N‐terminal amino acid residue with the C‐terminal *p*‐nitrophenol ester of a second peptide. The reaction is accelerated through the cavity of 24‐crown‐8 to form a crown ether–peptide [2]rotaxane. The reaction occurs under kinetic control that arises from transition state stabilization by the crown ether causing amidation to proceed faster through the cavity than outside of it. As the rotaxane‐forming reaction is most effective in toluene, a nonpolar solvent, we reasoned that a convenient strategy to rotaxanes of oligopeptides could be to prepare crown ether–tripeptide [2]rotaxanes and subsequently extend them at either or both termini. With sufficiently large amino acid side chains, the crown ether should remain trapped on the glycine residue used to assemble the rotaxane in both the tripeptide rotaxane building block and the oligopeptide rotaxane final product. The strategy could therefore give access to crown ether–oligopeptide rotaxane isomers that differ only in the position of the macrocycle on the axle. Such positional isomerism (i.e., mechanically restricted co‐conformational exchange between the positions of a threaded ring on an axle) is a form of diastereoisomerism possible for threaded molecular structures that is conceptually similar to atropisomerism (i.e., mechanically restricted rotation about a single bond).^[^ ^54^ ^]^

## Results and Discussion

### Metal‐Free Active Template Synthesis of Crown Ether‐Tripeptide [2]Rotaxanes

**Figure 1:**
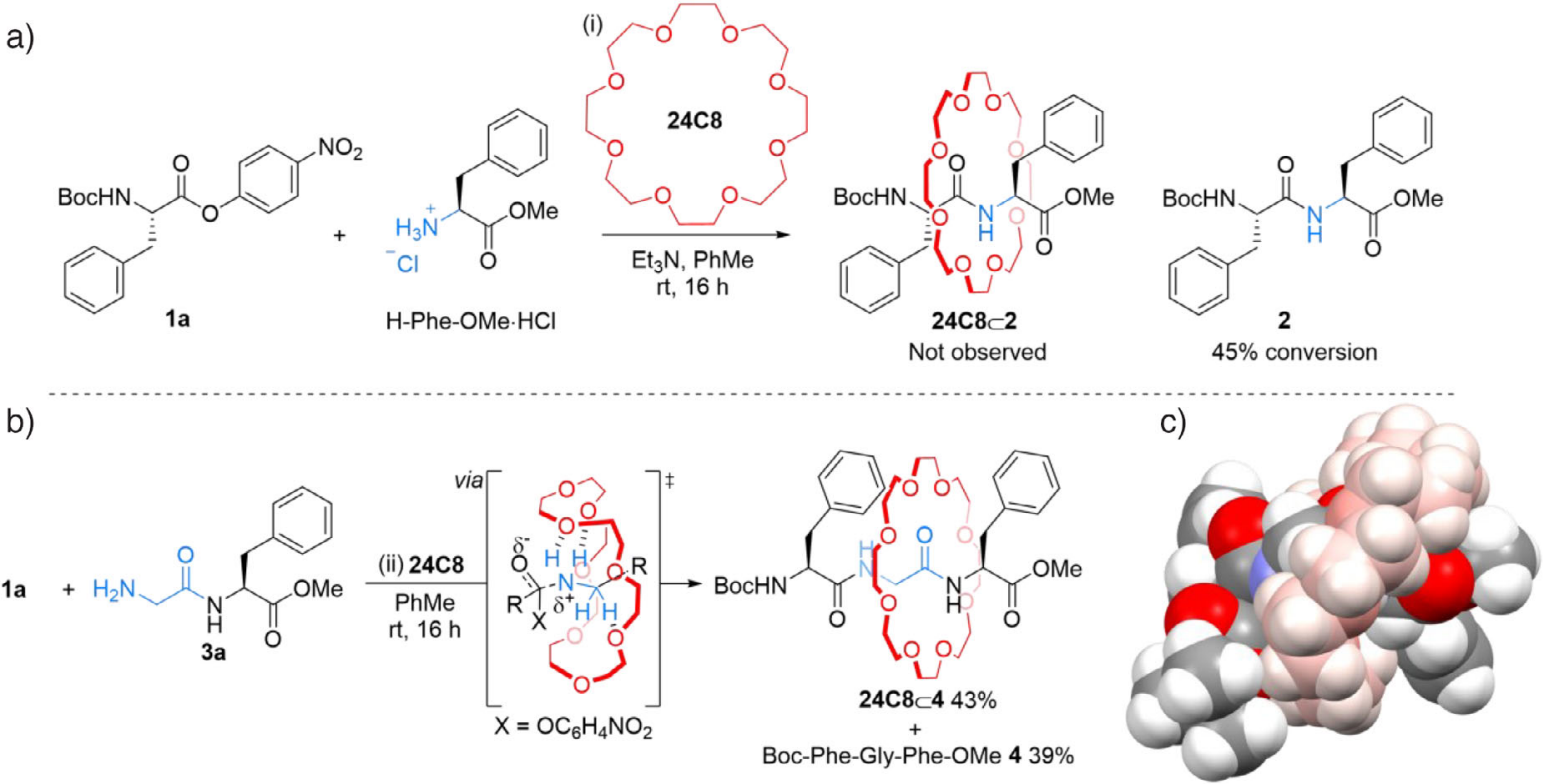
Crown‐ether–accelerated acylation of amino acids a) Unsuccessful [2]rotaxane formation by crown ether‐accelerated amide formation from H‐Phe‐OMe, activated ester **1a** and **24C8**. b) Tripeptide–based [2]rotaxane formation by crown‐ether‐accelerated amide formation from H‐Gly‐Phe‐OMe **3a**, activated ester **1a** and **24C8**. Reagents and conditions: (i) **1a** (1.0 equiv.), H‐Phe‐OMe·HCl (1.0 equiv.), **24C8** (1.0 equiv.), Et_3_N (2.5 equiv.), toluene (0.14 M), rt, 16 h. (ii) **1a** (1.0 equiv.), **3a** (1.0 equiv.), **24C8** (1.0 equiv.), toluene (0.14 M), rt, 16 h. c) Optimized structure of **24C8**⊂**4** calculated by density functional theory (DFT) using Gaussian 16 (see Supporting Information for coordinates and details). The crown ether is shaded pink to accentuate the different interlocked components.

Density functional theory (DFT) modeling indicated that canonical branched amino acids (other than alanine and serine) have side chains that are sufficiently large to trap 24‐crown‐8 (**24C8**) on a peptide axle. However, reacting the *p*‐nitrophenol ester of phenylalanine **1a** and the methyl ester of phenylalanine (H‐Phe‐OMe) with **24C8** in toluene (0.14 M of **1a**) at room temperature did not afford rotaxane **24C8**⊂**2** (Figure 1a). We did, however, observe an increase in the rate of formation of the unthreaded dipeptide, Boc‐Phe‐Phe‐OMe **2**, in the presence of the crown ether.^[^ ^55^ ^]^ It appeared that the steric bulk of the amino acid side chain of the nucleophile was preventing rotaxane formation from occurring. To circumvent this, we elongated the nucleophilic amino acid residue by a unit of glycine (Figure 1b). When H‐Gly‐Phe‐OMe **3a** was reacted with **1a** in the presence of **24C8**, the crown ether–tripeptide rotaxane **24C8**⊂**4** was formed in 43% yield, along with the unthreaded tripeptide **4** in 39% yield. The structure of the rotaxane was confirmed by high resolution mass spectrometry (HRMS) and tandem MS–MS of the [**24C8**⊂**4**+Na]^+^ ion (Supporting Information, Section 8, Figure S9). Molecular modeling (Figure 1c and Supporting Information, Section 9) shows the macrocycle is locked on the glycine unit by the sidechains of the adjacent Phe residues.

**Figure 2:**
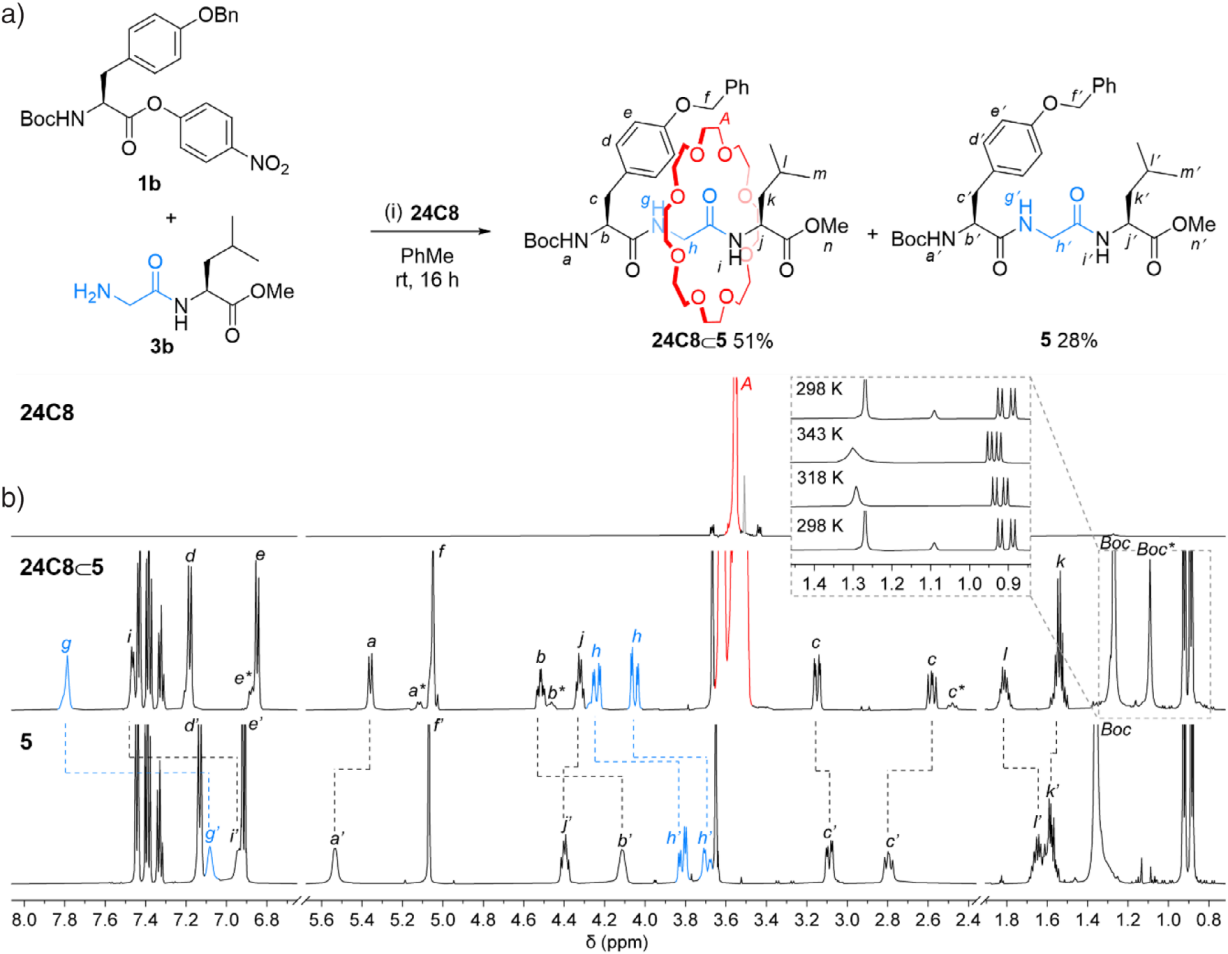
[2]Rotaxane formation by crown‐ether‐accelerated amide formation from a glycine‐based dipeptide, an activated ester and **24C8**. a) Synthesis of **24C8⊂5** from glycine dipeptide **3b**, activated ester **1b** and **24C8**. Reagents and conditions: **1b** (1.0 equiv.), **3b** (1.0 equiv.), **24C8** (1.0 equiv.), toluene (0.14 M), rt, 16 h. b) Partial ^1^H NMR spectra (600 MHz, CD_3_CN, 298 K) of **24C8** (top), **24C8⊂5** (middle), and **5** (bottom). Inset: variable temperature ^1^H NMR (600 MHz, CD_3_CN) at 298 K, 318 K, 343 K, and 298 K. Peaks in grey correspond to residual solvent peaks. Smaller peaks suffixed with * arise from restricted rotation of the Boc group in **24C8⊂5**.

We repeated this process with different amino acid building blocks (Table 1). H‐Gly‐Leu‐OMe **3b** was reacted with Boc‐Tyr(Bzl)‐ONp **1b** and **24C8**, to give **24C8**⊂**5** in 51% yield (Figure 2a). The interlocked structure of **24C8**⊂**5** was confirmed by ^1^H NMR spectroscopy (Figure 2b). In CD_3_CN substantial downfield shifts are observed for protons H_g_, H_h_, and H_i_ of the glycine residue in **24C8**⊂**5** compared to the noninterlocked thread **5** (Δ*δ*H_g_ = +0.70 ppm; Δ*δ*H_h_ = +0.33 ppm; Δ*δ*H_i_ = +0.52 ppm), a result of hydrogen bonding between the crown ether and the Gly unit of the tripeptide axle (Figure 2). Downfield shifts in H_b_ and H_l_ (+0.44 and +0.19 ppm, respectively), located at the different termini of the tripeptide backbone, show that the macrocycle interacts not only with protons in the central region of the rotaxane but also with those of the amino acid residues at both termini.

**Table 1:** Synthesis of crown ether–tripeptide [2]rotaxanes by a metal‐free active template reaction between activated esters **1a**–**i**, N‐terminus glycine dipeptides **3a**–**e** and **24C8**.^a)^

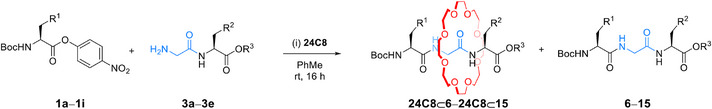

Entry	Starting materials	Rotaxane	Yield (%)	Entry	Starting materials	Rotaxane	Yield (%)
1	Boc‐Tyr(TIPS)‐ONp **1c** + H‐Gly‐Phe‐OMe **3a**	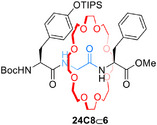	45	6	Boc‐Trp‐ONp **1f** + H‐Gly‐Tyr(Bzl)‐OMe **3c**	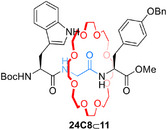	32
2	Boc‐Leu‐ONp **1d** + H‐Gly‐Tyr(Bzl)‐OMe **3c**	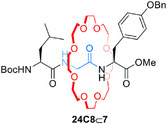	35	7	Boc‐Lys(Boc)‐ONp **1g** + H‐Gly‐Phe‐O^t^Bu **3d**	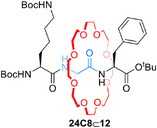	39
3	Boc‐Phe‐ONp **1a** + H‐Gly‐Leu‐OMe **3b**	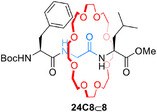	47	8	Boc‐Glu(OcHx)‐ONp **1h** + H‐Gly‐Tyr‐OMe **3e**	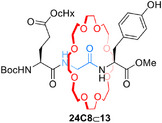	24
4	Boc‐Leu‐ONp **1d** + H‐Gly‐Phe‐O^t^Bu **3d**	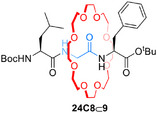	42	9	Boc‐Trp‐ONp **1f** + H‐Gly‐Leu‐OMe **3b**	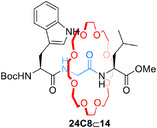	31
5	Boc‐Cha‐ONp **1e** + H‐Gly‐Tyr‐OMe **3e**	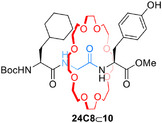	26	10	Boc‐Cys(Trt)‐ONp **1i** + H‐Gly‐Phe‐O^t^Bu **3d**	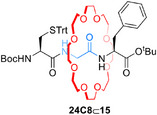	25

^a)^ Reagents and conditions: **1a**–**i** (1.0 equiv.), **3a**–**e** (1.0 equiv.), **24C8** (1.0 equiv.), toluene (0.14 M), rt, 16 h, 24%–47%. Abbreviations: Bzl = Benzyl, cHx = cyclohexyl, Np = *p*‐nitrophenyl, TIPS = triisopropylsilane, Trt = trityl.

The increase in the diastereotopic splitting of H_c_ (3.14 and 2.58 ppm for **24C8**⊂**5** compared to 3.08 and 2.79 ppm for **5**), coupled with a large difference in the observed ^3^ *J*_HbHc_ values between H_b_ and H_c_ (3.6 and 9.7 Hz in **24C8**⊂**5** compared to 5.2 and 9.7 Hz in **5**), indicates a restriction in rotation of the tyrosine side chain on the ^1^H NMR timescale. These effects become more pronounced in less polar solvents such as CDCl_3_ (see Supporting Information, Figure S1). The restriction in rotation of the amino acid side chain is accompanied by the appearance of rotamers from restricted rotation of the Boc group (labeled * in Figure 2b), the signals of which coalesce at 343 K (inset of Figure 2b and Figure S2). This behavior shows that the presence of the threaded crown ether can significantly influence the conformational dynamics of the amino acid side chains beyond the immediate encapsulated region of the axle. We explored the generality of the methodology by preparing a series of tripeptide rotaxanes with different amino acids on either side of glycine.

Tripeptide [2]rotaxanes **24C8**⊂**6**–**15** were synthesized (Table 1) from a range of activated esters **1a**–**1i** (1.0 equiv.) and N‐terminus glycine dipeptides **3a**–**3e** (1.0 equiv.). Reaction with **24C8** (1.0 equiv.) in toluene (0.14 M) at room temperature over 16 h (Table 1) yielded the [2]rotaxanes in good‐to‐modest yields (47%–24%), which is lower than typical^[^ ^28^, ^29^ ^]^ metal‐free active template reactions of alkylamines with crown ethers. In all cases the noninterlocked peptides **6**–**15** were the major byproducts, consistent with the modest yields of the rotaxanes. This results from the nucleophilicity of the glycine amine causing it to react relatively quickly with the electrophile, rather than requiring acceleration through interactions with the crown ether.

Triisopropylsilyl (TIPS), benzyl (Bzl), *t*‐butyloxycarbonyl (Boc), trityl (Trt), and cyclohexyl (cHx) protecting groups are all well tolerated by the metal‐free active template reaction, allowing the incorporation of tyrosine, lysine, tryptophan, glutamic acid, and cysteine residues within the rotaxane axle (Table 1, entries 1, 2, 6–10). Steric bulk is tolerated on the ester group (Table 1, entry 4), as are aliphatic side chains, including leucine and cyclohexyl alanine (Table 1, entry 5). The modest yield (26%) of **24C8**⊂**10** is a result of some acylation occurring at the unprotected phenol group. However, attempts to use amino acids branched at the beta position (e.g., the activated ester of valine: Boc‐Val‐ONp; see Supporting Information, Section 5.2) afforded only the noninterlocked tripeptide, presumably due to the increased steric bulk adjacent to the electrophilic center.

### Synthesis of Heptapeptide [2]Rotaxanes Mechanical Positional Isomers

**Scheme 1:**
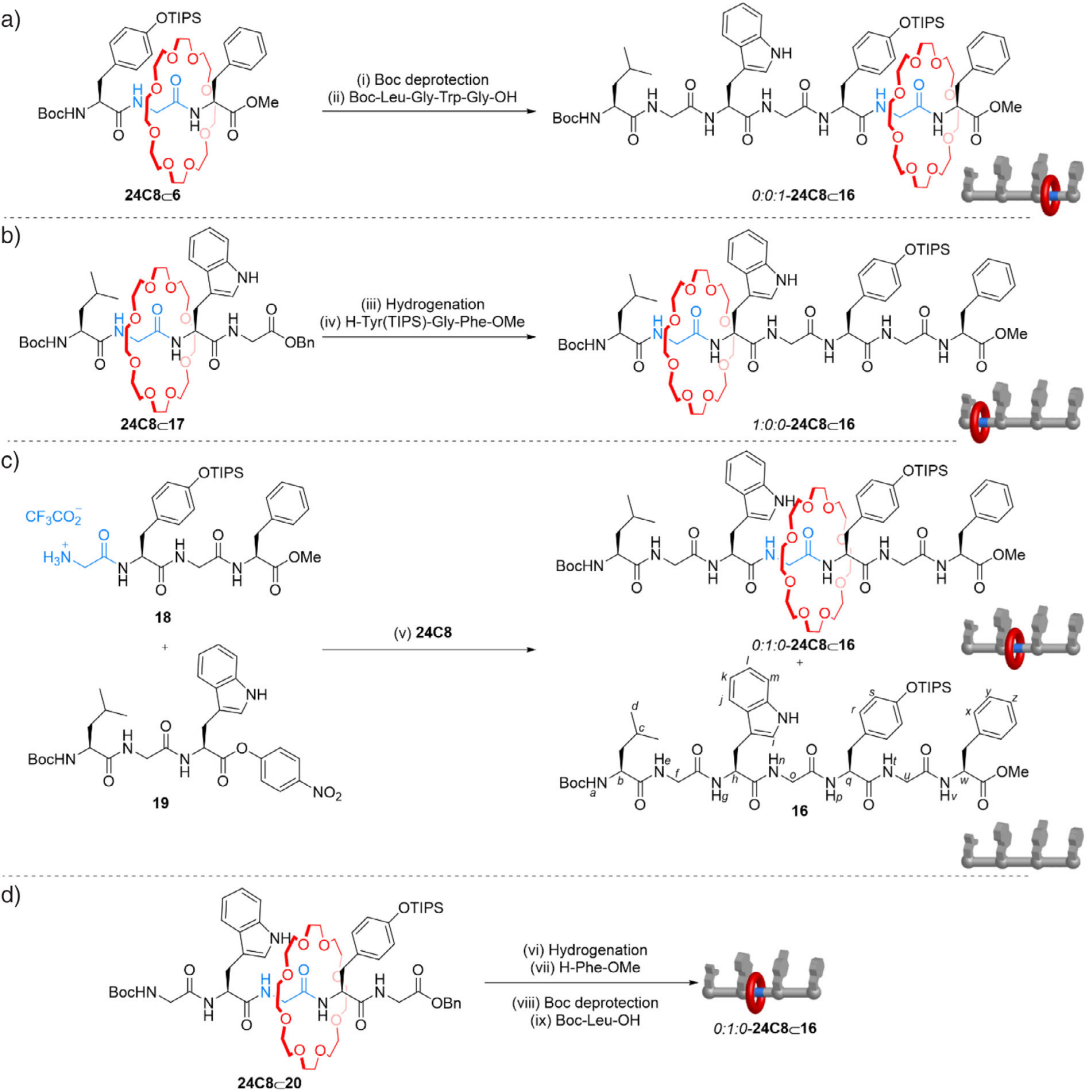
Diastereoselective synthesis of each of three positional isomers of a 24C8‐heptapeptide [2]rotaxane featuring a Boc‐Leu‐Gly‐Trp‐Gly‐Tyr(TIPS)‐Gly‐Phe‐OMe axle (**16**). a) Synthesis of *0:0:1*–**24C8**⊂**16** by N‐terminus elongation of rotaxane **24C8**⊂**6**. b) Synthesis of *1:0:0*–**24C8**⊂**16** by C‐terminus elongation of rotaxane **24C8**⊂**17**; c) Direct synthesis of *0:1:0*–**24C8**⊂**16** by metal‐free active template reaction between **18**, **19**, and **24C8**; d) Synthesis of *0:1:0*–**24C8**⊂**16** by stepwise C‐ and N‐elongation of pentapeptide **24C8**⊂**20**. Reagents and conditions: (i) CF_3_CO_2_H, CH_2_Cl_2_, rt, 1 h. (ii) Boc‐Leu‐Gly‐Trp‐Gly‐OH (1.0 equiv.), EDC·HCl (1.5 equiv.), HOBt (1.2 equiv.), *i*‐Pr_2_NEt (2.0 equiv.), CH_2_Cl_2_, 0 °C to rt, 12 h, 80% over two steps (iii) Pd/C, H_2_, MeOH, rt, 8 h. (iv) H‐Tyr(TIPS)‐Gly‐Phe‐OMe (1.0 equiv.), EDC·HCl (1.5 equiv.), HOBt (1.2 equiv.), *i*‐Pr_2_NEt (2.0 equiv.), CH_2_Cl_2_, 0 °C to rt, 12 h, 74% over two steps. (v) **18** (1.0 equiv.), **19** (1.0 equiv.), **24C8** (1.0 equiv.) Et_3_N (2.5 equiv.), toluene, rt, 36 h, *0:1:0*–**24C8**⊂**16** 11% and **16** 28%. (vi) Pd/C, H_2_, MeOH, rt, 8 h. (vii) H‐Phe‐OMe (1.0 equiv.), HOBt (1.2 equiv.), EDC·HCl (1.5 equiv.), *i*‐Pr_2_EtN (2.0 equiv.), CH_2_Cl_2_, 0 °C to rt, 12 h, 88% over two steps. (viii) CF_3_CO_2_H, CH_2_Cl_2_, rt, 1 h. (ix) Boc‐Leu‐OH, HOBt (1.2 equiv.), EDC·HCl (1.5 equiv.), *i‐*Pr_2_EtN (2.5 equiv.), CH_2_Cl_2_, 0 °C to rt, 12 h, 81% over two steps. The italicized prefix indicates the different glycine residues, starting from the N‐terminus, with the presence of the crown ether indicated by the digit 1.^[^ ^60^ ^]^

With the conditions for the formation of crown ether–tripeptide rotaxanes established, we next sought to create longer peptide rotaxanes featuring multiple different glycine “compartments” in which the crown ether could be selectively locked. An oligopeptide axle of Boc‐Leu‐Gly‐Trp‐Gly‐Tyr(TIPS)‐Gly‐Phe‐OMe **16** was chosen as an arbitrary sequence with an alternating pattern of branched amino acids and glycine residues. The steric bulk offered by the branched amino acids should be sufficient to prevent passage of **24C8** between glycine sites, providing a kinetically robust form of positional isomerism.^[^ ^54^, ^56^, ^57^, ^58^, ^59^ ^]^ Through metal‐free active template synthesis followed by elongation at the N‐ (Scheme 1a), C‐ (Scheme 1b) or both (Scheme 1d) or neither (Scheme 1c) termini, all possible positional isomers of the crown ether–heptapeptide [2]rotaxane **24C8**⊂**16** were synthesized.

To distinguish between the different positional isomers we use an italicized prefix to indicate the different glycine residues, starting from the N‐terminus of the axle, with the presence of the crown ether indicated by the digit 1.^[^ ^60^ ^]^ So *0:0:1*–**24C8**⊂**16** refers to the rotaxane diastereomer in which the ring is located on the right‐hand glycine residue of the axle.

In rotaxane *0:0:1*–**24C8**⊂**16** the ring is located on the glycine between the C‐terminal phenylalanine methyl ester and a tyrosine residue. The positional isomer was selectively synthesized via N‐terminal elongation of tripeptide rotaxane **24C8**⊂**6** (Table 1, entry 1). Boc deprotection with trifluoroacetic acid (CF_3_CO_2_H) followed by amide coupling with tetrapeptide Boc‐Leu‐Gly‐Trp‐Gly‐OH furnished *0:0:1*–**24C8**⊂**16** in good yield over two steps (Scheme 1a).

In rotaxane *1:0:0*–**24C8**⊂**16** the ring is located on the left‐hand glycine between the N‐terminus Boc‐protected leucine and a tryptophan residue. Positional isomer *1:0:0*–**24C8**⊂**16** was synthesized via C‐terminal elongation of the tetrapeptide rotaxane **24C8**⊂**17** (prepared by metal‐free active template synthesis from H‐Gly‐Trp‐Gly‐OBn, **1d** and **24C8**, see Supporting Information, Section 3.4).

Removal of the benzyl ester by hydrogenation, followed by amide coupling with tetrapeptide H‐Gly‐Tyr(TIPS)‐Gly‐Phe‐OMe, furnished the left‐hand positional isomer of the heptapeptide rotaxane, *1:0:0*–**24C8**⊂**16** (Scheme 1b).

In rotaxane *0:1:0*–**24C8**⊂**16** the ring is locked on the central glycine residue, between the tryptophan and a TIPS‐protected tyrosine. This rotaxane positional isomer was selectively synthesized in 33% overall yield (Supporting Information, Section 3.4) by stepwise elongation from each terminus (Scheme 1d), or directly from **24C8**, tetrapeptide **18** and *p*‐nitrophenyl ester tripeptide **19** to give *0:1:0*–**24C8**⊂**16** in 11% isolated yield (Scheme 1c), together with the noninterlocked parent heptapeptide thread **16** (28%).

### Spectroscopic Comparisons of the Positional Isomers of Crown Ether‐Heptapeptide Rotaxane 24C8⊂16

**Figure 3:**
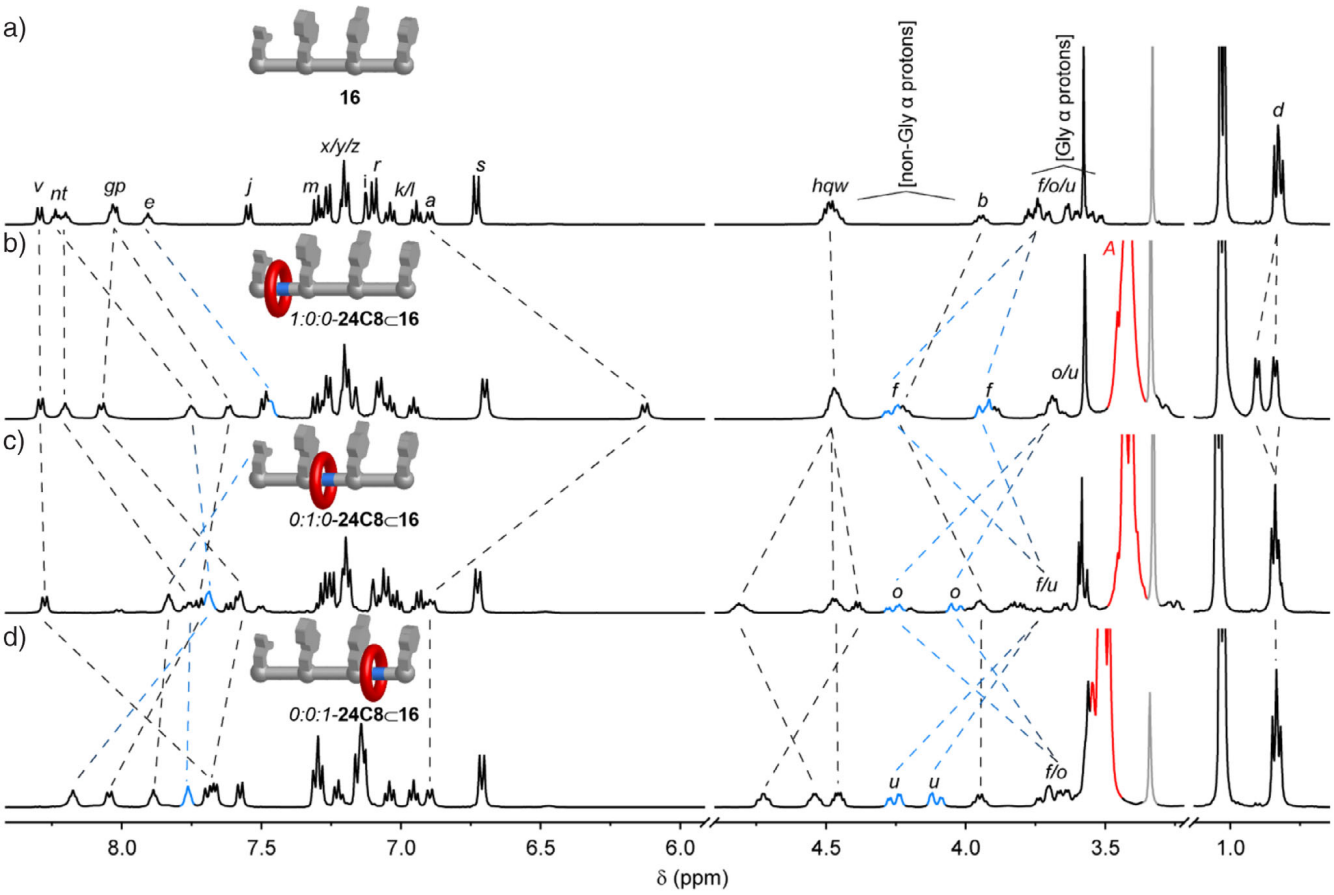
Partial ^1^H NMR spectra (500 MHz, DMSO‐*d_6_*, 298 K) of a) free peptide thread **16**, b) rotaxane positional isomer *1:0:0*–**24C8**⊂**16**, c) rotaxane positional isomer *0:1:0*–**24C8**⊂**16** and d) rotaxane positional isomer *0:0:1*–**24C8**⊂**16**. Dashed lines indicate equivalent axle proton resonances. Letters correspond to the labelling shown in Scheme 1. Residual solvent resonances shown in grey.

The properties of the *1:0:0*‐, *0:1:0*‐ and *0:0:1*‐diastereomers of **24C8**⊂**16** were probed using a range of spectroscopic and spectrometric techniques. No change in the ^1^H NMR spectra of the rotaxane positional isomers occurred upon heating at 70 °C in DMSO‐*d_6_* for 2 h, or after prolonged periods at room temperature. This indicates that, similar to lasso peptides, the side chains of leucine, tryptophan, tyrosine, and phenyl alanine are sufficiently bulky to prevent slippage of the 24C8 macrocycle over the side chains, which would allow the positional isomers to interconvert and/or dethreading of the macrocycle. In each rotaxane positional isomer, the presence of the macrocycle significantly alters the chemical shift of the α‐proton of the amino acid attached to the encapsulated glycine nitrogen atom (Figure 3). When compared to the noninterlocked thread peptide **16**; significant downfield shifts are observed for H_b_ in *1:0:0*–**24C8**⊂**16** (Δ*δ*H_b_ = +0.45 ppm), H_h_ in *0:1:0*–**24C8**⊂**16** (Δ*δ*H_h_ = +0.30 ppm) and H_q_ in *0:0:1*–**24C8**⊂**16** (Δ*δ*H_q_ = +0.21 ppm). The protons associated with the encapsulated glycine residue are also downshifted (protons H_f_, H_o_ and H_u_, in *1:0:0*–**24C8**⊂**16**, *0:1:0*–**24C8**⊂**16** and *0:0:1*–**24C8**⊂**16**, respectively). Substantial upfield shifts for the amide protons H_a_, H_e_, H_g_, H_n_ (Δ*δ*H = −0.78, −0.43, −0.40, and −0.49 ppm) in *1:0:0*–**24C8**⊂**16**, H_g_, H_n_, H_p_, and H_t_ in *0:1:0*–**24C8**⊂**16** (Δ*δ*H = −0.26, −0.48, −0.45, and −0.44 ppm), and H_p_, H_t_ and H_v_ (Δ*δ*H = −0.33, −0.44, and −0.63 ppm) in *0:0:1*–**24C8**⊂**16** indicate that the crown ether also interacts with the amide group of the *C*‐adjacent residue. As in the tripeptide rotaxanes **24C8**⊂**4**–**14**, the diastereotopic splitting for the encapsulated glycine methylene becomes more pronounced, as do the protons of the leucine residue (H_d_), within *1:0:0*–**24C8**⊂**16**. The presence of the macrocycle reduces the rotation of nearby amides within the compartment it occupies.

The chiroptical properties of each of the rotaxane isomers were also investigated. The UV−visible absorption (UV−vis) spectra of all of the three positional isomers of rotaxane **24C8**⊂**16** are virtually identical to that of the unthreaded heptapeptide **16**. However, the peptide rotaxane isomers and thread all differ in their circular dichroism (CD) spectra (Supporting information Section 6, Figure S3), with differences in the near UV region at 237 and 275 nm indicating differences in the conformation of the aromatic side chains in each of the three isomers.^[^ ^61^ ^]^

### Enzymatic Degradation of the Positional Isomers of Crown Ether‐Heptapeptide Rotaxane 24C8⊂16

The crown ether–heptapeptide rotaxane positional isomers of **24C8**⊂**16** were each subjected to enzymatic proteolysis. The rotaxanes were treated with pronase—a broad scope mixture of endo‐ and exopeptidases isolated from *Streptomyces griseus* ^[^ ^62^ ^]^—in phosphate buffer (pH 7.4) at 37 °C, and all underwent overall peptidic hydrolysis at similar rates (See Supporting Information, Section 7, Figure S4), with complete consumption of each rotaxane observed within 8 h. The different positional isomers produce different products from the enzymatic degradation; in each case the major product was a shorter rotaxane, with residues cleaved from either the N or C terminus, or from both in the case of *0:1:0–* **24C8**⊂**16** (see Supporting Information, Section 7, Figures S5–S7). These results demonstrate that the crown ether does not inhibit enzymatic proteolysis for the entire length of the heptapeptide rotaxane axle, but rather provides substantial protection from degradation only for the three to four residues local to the encapsulated region. This contrasts with the global protection provided to peptidic axles in benzylic amide macrocycle rotaxanes,^[^ ^20^ ^]^ but is similar to the protection provided by natural lasso peptides which has been used to generate intermediates for unnatural catenane and rotaxane synthesis.^[^ ^25^ ^]^

### Tandem Mass Spectrometry Fragmentation of the Positional Isomers of Crown Ether‐Heptapeptide Rotaxane 24C8⊂16

**Figure 4:**
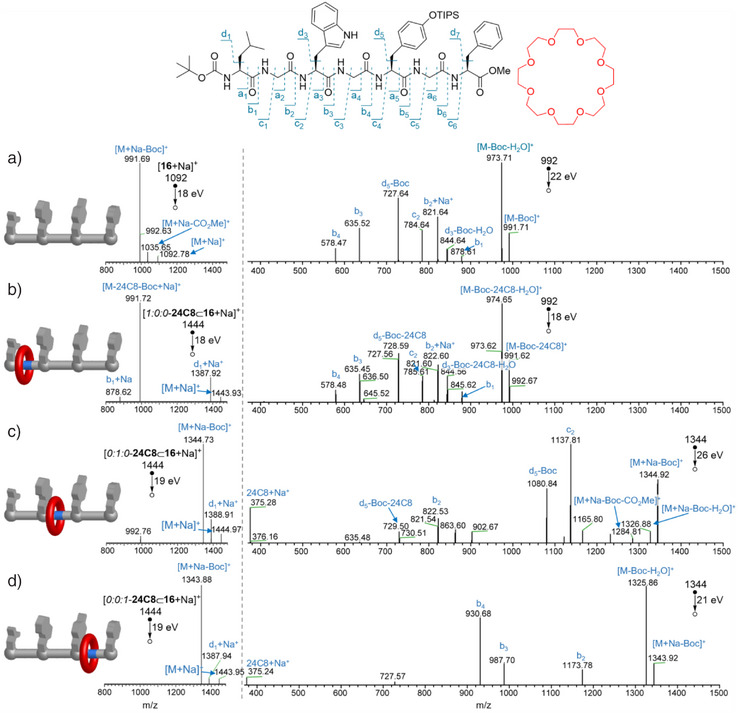
Tandem MS/MS spectrometry and collision‐induced dissociation (CID) analysis of crown ether‐heptapeptide rotaxane positional isomers *1:0:0*–**24C8**⊂**16**, *0:1:0*–**24C8**⊂**16** and *0:0:1*–**24C8**⊂**16** and unthreaded heptapeptide **16**. a) left: Tandem MS/MS of [**16**+Na]^+^ (18 eV) at *m*/*z*=1092, and right: MS/MS (22 eV) of *m*/*z*=992. b) left: Tandem MS/MS of [*1:0:0*–**24C8**⊂**16**+Na]^+^ (18 eV) at *m*/*z*=1444, and right: MS/MS (18 eV) of *m*/*z*=1387. c) left: Tandem MS/MS of [*0:1:0*–**24C8**⊂**16**+Na]^+^ (19 eV) at *m*/*z*=1444, and right: MS/MS (26 eV) of *m*/*z*=1344, and d) left: Tandem MS/MS of [*0:0:1*–**24C8**⊂**16**+Na]^+^ (19 eV) at *m*/*z*=1444, and right: MS/MS (21 eV) of *m*/*z*=1344.

Analysis of each of the different positional isomers of rotaxane **24C8**⊂**16** by tandem mass spectrometry (ESI(+)‐MS/MS; Figure 4a–d, left‐hand spectra) was carried out with CID (collision‐induced dissociation) analysis^[^ ^63^ ^]^ of the most abundant fragment (Figure 4a–d, right‐hand spectra).

When subjected to CID, the unthreaded heptapeptide **16** initially undergoes loss of the Boc group (*m*/*z* 991, Figure 4a left‐hand). This fragment was further ionized at 18 eV, giving typical b‐type amide at *m*/*z* = 821 (b_2_), *m*/*z* 635 (b_3_) and *m*/*z* 578 (b_4_), along with d‐type side chain fragmentation of the tryptophan and tyrosine residues (peaks observed at *m*/*z* 844 (d_3_) and *m*/*z* 727 (d_5_)).^[^ ^64^ ^]^

ESI‐MS/MS of *1:0:0*–**24C8**⊂**16** gave similar results: CID of the parent adduct [*1:0:0*–**24C8**⊂**16**+Na]^+^ *m*/*z* = 1444, gave fragmentation of the Boc group and loss of the crown ether macrocycle, indicated by *m*/*z* = 991, along with some d_1_ side chain fragmentation of the leucine residue (*m*/*z* = 1387) and b_1_ amide fragmentation and concomitant dethreading of the crown ether (*m*/*z* = 878). CID of the ion at *m*/*z* 991, gave a nearly identical spectrum to that of **16** (Figure 4b, right‐hand spectra), indicating that once the Boc group has been removed, the macrocycle dethreads from its compartment over the N‐terminus leucine residue.

ESI‐MS/MS of *0:1:0*–**24C8**⊂**16** gives a different outcome. CID of the major ion produced by ESI (*m*/*z* = 1444, [*0:1:0–* **24C8**⊂**16**+Na]^+^) resulted in fragmentation of the Boc group (Figure 4c, left‐hand spectra). However, the fragment now retained the crown ether on the axle (*m*/*z* = 1344), indicating that the location of the macrocycle in the central region of the axle prevents its dethreading. CID of *m*/*z* 1344 led first to loss of the carboxyl group at the C‐terminus (*m*/*z* = 1284) and then to c and d‐type fragmentation where the rotaxane architecture was again maintained (*m/z* = 1137 (c_2_) and 1080 (d_5_)).

Finally, CID of the molecular ion of rotaxane positional isomer *0:0:1*–**24C8**⊂**1** (*m*/*z* = 1444) also resulted in initial cleavage of the Boc group (Figure 4d, left‐hand spectra). The peak at *m*/*z* 1344 was again fragmented, which led predominantly to b‐type fragmentations of the peptide axle (*m*/*z* = 1173 (b_2_), 987 (b_3_) and 930 (b_4_)), similar to the behavior of thread **16** and isomer *1:0:0*–**24C8**⊂**16**, although for all of these b‐type fragmentations the rotaxane structure remains intact.

Overall, the tandem MS/MS and CID results show that chemical protection is afforded by the macrocycle to the region of the rotaxane axle occupied by the crown ether. Again, this mirrors the behavior and properties of naturally occurring lasso peptides,^[^ ^5^, ^22^ ^]^ indicating that crown ether‐peptide rotaxanes can mimic key aspects of the structural features that give lasso peptides their attractive properties.

## Conclusions

Our findings demonstrate that metal‐free active template synthesis is an effective and general route to 24‐crown‐8–tripeptide rotaxanes. The methodology is distinctive in its ability to incorporate a broad range of canonical (and unnatural) peptides into a threaded peptide backbone without the need for additional nonamide template sites in the axle. The tripeptide of the rotaxanes can subsequently be elongated at either or both the N‐ and C‐termini to produce longer oligopeptide rotaxanes. The crown ether is locked on the glycine residue used to assemble the rotaxane by the side chains of the adjacent amino acid residues. This enables the preparation of different diastereomeric positional isomers of rotaxanes that differ only in the position of the macrocycle on the peptide axle. The presence of the crown ether influences the conformation and side‐chain dynamics principally in, and adjacent to, the region where the macrocycle is locked in place. The macrocycle protects this localized region of the peptide axle from enzymatic degradation and from chemical reactions such as fragmentation caused by collisions with reactive ions during mass spectrometry. Crown ether–peptide rotaxanes represent a new class of readily accessible analogs of naturally occurring mechanically interlocked peptides.

## Conflict of Interests

The authors L.Z., R.Y., P.C. and Z.‐H.Z. declare that they are named inventors in a patent application based on this system [CN 119192279A]. Other authors declare no conflict of interest.

## Supporting information



Supporting Information

## Data Availability

The data that support the findings of this study are available in the supplementary material of this article.
