# Supplementary material for: Crown Ether–Peptide Rotaxanes
Source: Angew Chem Int Ed Engl. 2025 Aug 6;64(38):e202513115. doi: 10.1002/anie.202513115 (PMC12435445; doi:10.1002/anie.202513115)
Supplement: Supplementary file 1 — Supporting Information [file ANIE-64-e202513115-s001.pdf]

Crown Ether-Peptide Rotaxanes

Peng-Lai Wang,[1,2‡] Peng Chen,[1‡] Raorao Yang,[1] Daniel J. Tetlow,[2] Zhi-Hui Zhang,[1] Stephen D. P. Fielden,[2] Prodip Howlader,[2] Liang Zhang[1] and David A. Leigh*[,1,2]

[1]School of Chemistry and Molecular Engineering, East China Normal University, Shanghai, 200062, China

[2]Department of Chemistry, University of Manchester, Oxford Road, Manchester, M13 9PL, UK.

*E-mail: david.leigh@manchester.ac.uk

Supporting Information

Table of Contents

1. Abbreviations 2
2. General information 2
3. Synthetic procedures and characterization details 3
3.1 Synthesis of activated esters **1a** – **1i** 3
3.2 Synthesis of dipeptides **3a**·CF_3CO_2H – **3e**·CF_3CO_2H 9
3.3 Synthesis of peptide rotaxanes **24C8⊂4** – **24C8⊂15** 12
3.4. Synthesis of heptapeptide rotaxane positional isomers 25
3.4.1. Synthesis of *0:0:1*-**24C8⊂16** 25
3.4.2. Synthesis of *1:0:0*-**24C8⊂16** 27
3.4.3. Synthesis of *0:1:0*-**24C8⊂16** and **16** 32
3.4.4. Alternative synthesis of *0:1:0*-**24C8⊂16** 39
4. ^{1}H NMR analysis of **24C8⊂5** 46
5. Limitations 48
6. CD and UV spectra of *1:0:0*-**24C8⊂16**, *0:1:0*-**24C8⊂16**, *0:0:1*-**24C8⊂16** and **16** 49
7. Pronase hydrolysis of *1:0:0*-**24C8⊂16**, *0:1:0*-**24C8⊂16** and *0:0:1*-**24C8⊂16** 50
8. Tandem MS/MS analysis of **24C8⊂4**, **16**, *1:0:0*-**24C8⊂16**, *0:1:0*-**24C8⊂16** and *0:0:1*-**24C8⊂16** 52
9. Molecular modelling of **24C8⊂4** 57
10. NMR spectra 58
11. Coordinates of rotaxane **24C8⊂4** from molecular modelling studies 114

1. Abbreviations

aq.: aqueous; ASAP: atmospheric solids analysis probe ionization; Bn: benzyl; Boc: *tert*-butoxycarbonyl; Cha: cyclohexyl-L-alanine; cHx: cyclohexyl; Cys: Cysteine; DCM: dichloromethane; DIPEA: *N*,*N*-Diisopropylethylamine; DMAP: 4-(dimethylamino)pyridine; DMSO: dimethylsulfoxide; DCE: 1,2-dichloroethane; EDC·HCl: *N*-(3-dimethylaminopropyl)-*N'*-ethylcarbodiimide hydrochloride; eq: equivalents; ESI: electrospray ionization; Et: ethyl; Et_3N: triethylamine; EtOAc: ethyl acetate; Glu: L-glutamic acid; Gly: glycine; h: hour; HOBt·H_2O: 1-Hydroxybenzotriazole hydrate; HPLC: high-performance liquid chromatography; HRMS: high-resolution mass spectrometry; Leu: L-leucine; Lys: L-Lysine; Me: methyl; min: minutes; NMR: nuclear magnetic resonance; Np: 4-nitrophenol; OTf: trifluoromethanesulfonate; Pd/C: palladium on carbon; PE: petroleum ether (boiling point 40-60 °C); Phe: L-phenylalanine; ppm: parts per million; Prep TLC: preparative thin layer chromatography; quant.: quantitative; RP: reverse phase; rt: room temperature; TIPS: triisopropylsilyl; *t*-Bu: tertiary butyl; TFA: trifluoroacetic acid; THF: tetrahydrofuran; TLC: thin-layer chromatography; Trp: L-tryptophan; Ts: 4-toluenesulfonyl; Trt: Trityl; Tyr: L-tyrosine; Val: L-valine.

2. General Information

Unless stated otherwise, reagents were obtained from commercial sources and used without purification. ^{1}H NMR and ^{13}C NMR were measured on a Bruker 400 MHz Spectrometer, a Bruker 500 MHz and a Bruker 600 MHz Spectrometer. Chemical shifts are reported in parts per million (ppm) relative to tetramethylsilane from high to low frequency using the residual solvent peak as the internal reference ($CDCl_3$ = 7.26 ppm, CD_2Cl_2 = 5.32 ppm, $(CD_3)_2SO$ = 2.50 ppm, CD_3CN = 1.94 ppm). All ^{1}H resonances are reported to the nearest 0.01 ppm. The multiplicity of ^{1}H signals are indicated as: s = singlet; d = doublet; t = triplet; q = quartet; multiplet; br = broad; or combinations of thereof. Coupling constants (*J*) are quoted in Hz and reported to the nearest 0.1 Hz. Where appropriate, averages of the signals from peaks displaying multiplicity were used to calculate the value of the coupling constant. ^{13}C NMR spectra were recorded on the same spectrometer at 298 K with the central resonance of the solvent peak as the internal reference ($CDCl_3$ = 77.16 ppm, CD_2Cl_2 = 53.46 ppm, $(CD_3)_2SO$ = 39.52 ppm, CD_3CN = 118.26 ppm). All ^{13}C resonances are reported to the nearest 0.01 ppm. DEPT, COSY, HSQC and HMBC experiments were used to aid structural determination and spectral assignment. Fully characterized compounds were chromatographically homogeneous. Reactions were monitored by thin layer chromatography (TLC) using silicycle pre-coated silica gel plates. Flash column chromatography was performed using silica gel 60 (particle size 200-400 mesh ASTM, purchased from Yantai, China) as the stationary phase. Preparative TLC was performed using UNIPLATE^{TM} 1000 micron, 20 × 20 cm, UV254 preparatory plates (ANALTECH). Low resolution ESI mass spectrometry was performed with a Thermo Scientific LCQ Fleet Ion Trap Mass Spectrometer. High-resolution mass spectrometry was carried out by staff at the Mass Spectrometry Service, School of Chemistry and Molecular Engineering, East China Normal University.

3. Synthetic procedures and characterization details

3.1. Synthesis of activated esters 1a – 1i

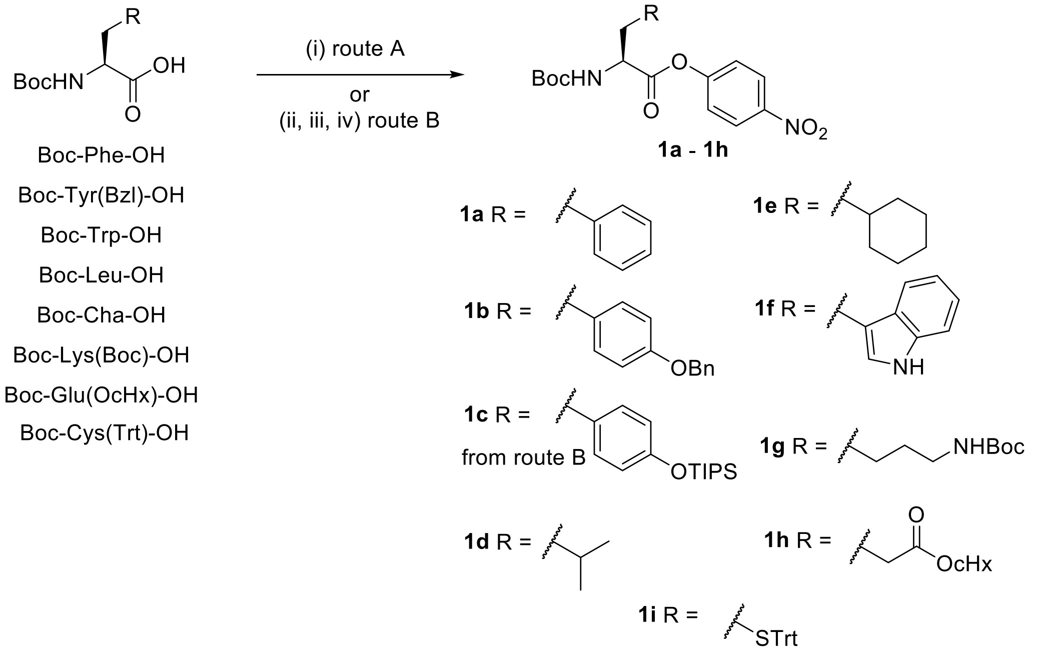

Scheme S1. Reagents and conditions: **1a**, **1b**, **1d** –**1i** synthesized by route A (i) 4- Nitrophenol, EDC·HCl, DMAP, CH_2Cl_2, rt, 16 h, 61 – 88%. **1c** synthesized by route B (ii) TIPSCl, 1-imidazole, CH_2Cl_2, rt, 2 h, 91%. (iii) $LiOH·H_2O$, MeOH/THF/H_2O, rt, 1 h. (iv) 4-Nitrophenol, EDC·HCl, DMAP, CH_2Cl_2, rt, 16 h, 70% over two steps.

Synthesis of **1a**

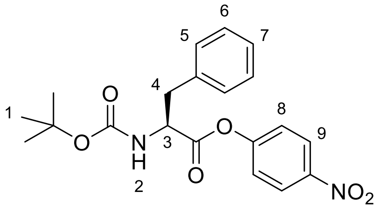

A solution of Boc-Phe-OH (345 mg, 1.3 mmol, 1.0 eq.), 4-nitrophenol (222 mg, 1.6 mmol, 1.2 eq.), EDC·HCl (368 mg, 1.9 mmol, 1.5 eq.) and DMAP (31 mg, 0.26 mmol, 0.2 eq.) in CH_2Cl_2 (15 mL) was stirred at room temperature for 16 hours. The reaction mixture was concentrated under reduced pressure. Purification of the crude residue by flash column chromatography (SiO_2, EtOAc/hexane 1:5) afforded **1a** (351 mg, 0.9 mmol, 69%) as a yellow solid. **^{1}H NMR** (400 MHz, $CDCl_3$, 298 K) δ 8.25 (d, J = 9.1 Hz, 2H, H_9), 7.38 – 7.28 (m, 3H, $H_{6,7}$), 7.25 – 7.21 (m, 2H, H_5), 7.14 (d, J = 9.1 Hz, 2H, H_8), 5.05 (d, J = 7.9 Hz, 1H, H_2), 4.80 (q, J = 6.9 Hz, 1H, H_3), 3.28 – 3.15 (m, 2H, H_4), 1.45 (s, 9H, H_1). **^{13}C NMR** ($CDCl_3$, 101 MHz, 298 K) δ 170.15, 155.29, 155.07, 145.62, 135.44, 129.45, 129.03, 127.66, 125.38, 122.41, 80.74, 54.96, 38.31, 28.40.

Synthesis of **1b**

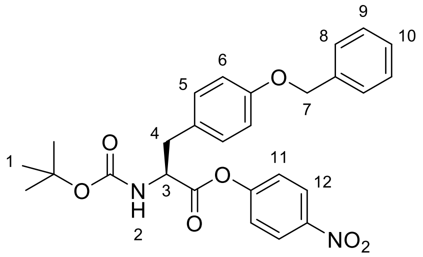

A solution of Boc-Tyr(Bzl)-OH (500 mg, 1.3 mmol, 1.0 eq.), 4-nitrophenol (222 mg, 1.6 mmol, 1.2 eq.), EDC·HCl (368 mg, 1.9 mmol, 1.5 eq.) and DMAP (31 mg, 0.26 mmol, 0.2 eq.) in anhydrous CH_2Cl_2 (15 mL) was stirred at room temperature for 16 hours. The reaction mixture was concentrated under reduced pressure. Purification of the crude residue by flash column chromatography (SiO_2, EtOAc/hexane 1:5) afforded **1b** (541 mg, 1.1 mmol, 84%) as a colorless solid. **^{1}H NMR** (400 MHz, $CDCl_3$, 298 K) δ 8.24 (d, J = 9.1 Hz, 2H, H_{12}), 7.47 – 7.30 (m, 5H, H_{8-10}), 7.16 – 7.11 (m, 4H, $H_{5,11}$), 6.96 (d, J = 8.6 Hz, 2H, H_{12}), 5.07 (s, 2H, H_7), 5.02 (d, J = 8.0 Hz, 1H, H_2), 4.75 (q, J = 6.8 Hz, 1H, H_3), 3.23 – 3.10 (m, 2H, H_4), 1.45 (s, 9H, H_1). **^{13}C NMR** (101 MHz, $CDCl_3$, 298 K) δ 170.24, 158.31, 155.29, 155.13, 145.62, 136.94, 130.52, 128.79, 128.21, 127.56, 125.39, 122.42, 115.34, 80.68, 70.14, 55.08, 37.47, 28.42. **HRMS** (ESI$^+$) calculated for $C_{27}H_{28}N_2O_7Na$ $[M+Na]^+$ = 515.1794, found 515.1778.

Synthesis of **S1**

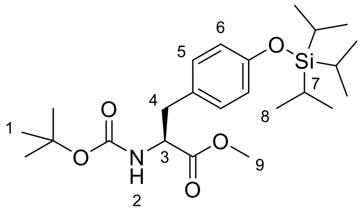

To a solution of Boc-Tyr-OMe (2.95 g, 10 mmol, 1.0 equiv.) in dichloromethane (40 mL) was added TIPSCl (2.88 g, 15 mmol, 1.5 equiv.) and 1-imidazole (1.36 g, 20 mmol, 2.0 equiv.). The reaction mixture was stirred for 2 hours at room temperature. The solvent was removed under reduced pressure. Flash column chromatography of the crude residue (SiO_2, EtOAc/hexane 1:50) afforded **S1** (4.1 g, 9.1 mmol, 91%) as a colorless oil. **^{1}H NMR** (500 MHz, $CDCl_3$, 298 K) δ 6.98 – 6.95 (m, 2H, H_5), 6.81 – 6.78 (m, 2H, H_6), 4.95 (d, J = 8.3 Hz, 1H, H_2), 4.52 (q, J = 6.7 Hz, 1H, H_3), 3.67 (s, 3H, H_9), 3.05 – 2.94 (m, 2H, H_4), 1.42 (s, 9H, H_1), 1.29 – 1.19 (m, 3H, H_7), 1.08 (d, J = 7.4 Hz, 18H, H_8). **^{13}C NMR** (125 MHz, $CDCl_3$, 298 K) δ 172.64, 155.25, 130.31, 128.46, 120.12, 79.99, 54.70, 52.24, 37.79, 28.44, 18.04, 12.77. **HRMS** (ESI$^+$) Calculated for $C_{24}H_{41}O_5NNaSi$ $[M+Na]^+$ = 474.2646, found 474.2642.

Synthesis of **1c**

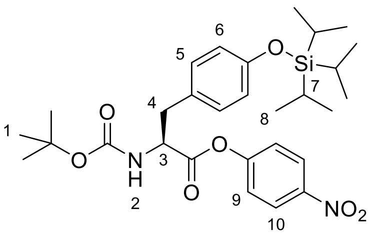

To a solution of **S1** (2.25 g, 5.0 mmol, 1.0 equiv.) in MeOH/THF/H_2O (2:2:1, 25 mL) was added LiOH·H_2O (315 mg, 7.5 mmol, 1.5 equiv.). The reaction mixture was stirred for 1 hour at room temperature. NH_4Cl (405 mg, 7.5 mmol, 1.5 equiv.) was added to quench the reaction. The solvent was removed under reduced pressure and the crude acid was used without further purification.

A solution of the crude acid, 4-nitrophenol (222 mg, 1.6 mmol, 1.2 eq.), EDC·HCl (1.44 g, 7.5 mmol, 1.5 eq.) and DMAP (122 mg, 1.0 mmol, 0.2 eq.) in anhydrous CH_2Cl_2 (55 mL) was stirred at room temperature for 16 hours. The reaction mixture was concentrated under reduced pressure. Purification of the crude residue by flash column chromatography (SiO_2, EtOAc/hexane 1:5) afforded **1c** (1.95 g, 3.5 mmol, 70%) as a colorless oil. **^{1}H NMR** (500 MHz, CD_2Cl_2, 298 K) δ 8.25 – 8.22 (m, 2H, H_{10}), 7.24 – 7.17 (m, 2H, H_5), 7.16 – 7.08 (m, 2H, H_9), 6.90 – 6.84 (m, 2H, H_5), 5.09 (d, *J* = 7.6 Hz, 1H, H_2), 4.70 (q, *J* = 7.0 Hz, 1H, H_3), 3.15 (d, *J* = 6.7 Hz, 2H, H_4), 1.43 (s, 9H, H_1), 1.31 – 1.19 (m, 3H, H_7), 1.10 (d, *J* = 7.5 Hz, 18H, H_8). **^{13}C NMR** (125 MHz, CD_2Cl_2, 298 K) δ 170.76, 156.07, 155.76, 155.64, 146.09, 130.86, 128.52, 125.70, 122.91, 120.70, 80.70, 55.69, 37.81, 28.56, 18.23, 13.22. **HRMS** (ESI$^+$) Calculated for $C_{29}H_{42}O_7N_2SiNa$ $[M+Na]^+$ = 581.2630, found 581.2637.

Synthesis of **1d**

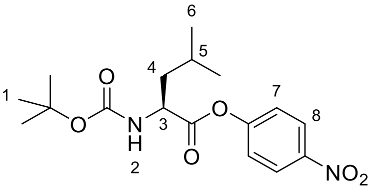

A solution of Boc-Leu-OH (500 mg, 1.3 mmol, 1.0 eq.), 4-nitrophenol (222 mg, 1.6 mmol, 1.2 eq.), EDC·HCl (368 mg, 1.9 mmol, 1.5 eq.) and DMAP (31 mg, 0.26 mmol, 0.2 eq.) in CH_2Cl_2 (15 mL) was stirred at room temperature for 16 hours. The reaction mixture was concentrated under reduced pressure. Purification of the crude residue by flash column chromatography (SiO_2, EtOAc/hexane 1:5) afforded **1d** (376 mg, 1.0 mmol, 77%) as a yellow solid. **^{1}H NMR** (400 MHz, $CDCl_3$, 298 K) *δ* 8.26 (d, *J* = 9.1 Hz, 2H, H_8), 7.29 (d, *J* = 9.1 Hz, 2H, H_7), 4.96 (d, *J* = 8.2 Hz, 1H, H_2), 4.51 (td, *J* = 8.9, 4.5 Hz, 1H, H_3), 1.87 – 1.71 (m, 2H, $H_{4a,5}$), 1.71 – 1.58 (m, 1H, H_{4b}), 1.45 (s, 9H, H_1), 1.28 – 0.99 (m, 6H, H_6). **^{13}C NMR** (101 MHz, $CDCl_3$, 298 K) *δ* 171.57, 155.62, 155.39, 145.57, 125.36, 122.45, 80.52, 52.58, 41.19, 28.40, 25.05, 23.01, 21.87. **HRMS** (ESI$^+$) calculated for $C_{17}H_{24}N_2O_6Na$ $[M+Na]^+$ = 375.1532, found 375.1536.

Synthesis of **1e**

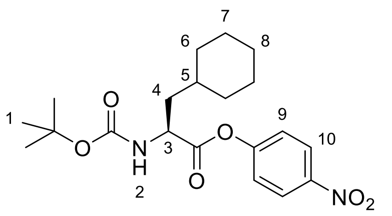

A solution of Boc-L-Cha-OH (352 mg, 1.3 mmol, 1.0 eq.), 4-nitrophenol (222 mg, 1.6 mmol, 1.2 eq.), EDC·HCl (368 mg, 1.9 mmol, 1.5 eq.) and DMAP (31 mg, 0.26 mmol, 0.2 eq.) in CH_2Cl_2 (15 mL) was stirred at room temperature for 16 hours. The reaction mixture was concentrated under reduced pressure. Purification of the crude residue by flash column chromatography (SiO_2, EtOAc/hexane 1:4) afforded **1e** (382 mg, 1.0 mmol, 77%) as a colorless solid. **^{1}H NMR** (400 MHz, $CDCl_3$, 298 K) δ 8.22 (d, J = 9.1 Hz, 2H, H_{10}), 7.25 (d, J = 9.1 Hz, 2H, H_{11}), 4.96 (d, J = 8.1 Hz, 1H, H_2), 4.49 (td, J = 8.7, 5.1 Hz, 1H, H_3), 1.86 – 1.46 (m, 7H, $H_{4,6a,7a,8a}$), 1.48 – 1.34 (m, 10H, $H_{1,5}$), 1.27 – 0.84 (m, 5H, $H_{6b/7b/8b}$). **^{13}C NMR** (101 MHz, $CDCl_3$, 298 K) δ 171.66, 155.62, 155.38, 145.48, 125.32, 122.43, 80.46, 51.90, 39.71, 34.22, 33.67, 32.42, 28.36, 26.37, 26.27, 26.05. **HRMS** (ESI$^+$) calculated for $C_{20}H_{28}N_2O_6Na$ $[M+Na]^+$ = 415.1845, found 415.1884.

Synthesis of **1f**

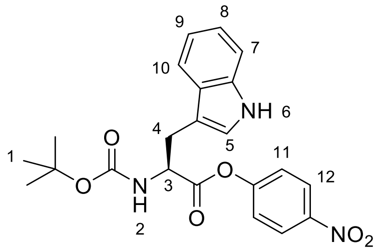

A solution of Boc-Trp-OH (395 mg, 1.3 mmol, 1.0 eq.), 4-nitrophenol (222 mg, 1.5 mmol, 1.2 eq.), EDC·HCl (368 mg, 1.9 mmol, 1.5 eq.) and DMAP (31 mg, 0.26 mmol, 0.2 eq.) in CH_2Cl_2 (15 mL) was stirred at room temperature for 16 hours. The reaction mixture was concentrated under reduced pressure. Purification of the crude residue by flash column chromatography (SiO_2, EtOAc/hexane 1:5) afforded **1f** (414 mg, 1.0 mmol, 77%) as a yellow solid. **^{1}H NMR** (400 MHz, $CDCl_3$, 298 K) δ 8.31 – 8.14 (m, 3H, $H_{6,12}$), 7.60 (d, J = 7.9 Hz, 1H, H_{10}), 7.41 (d, J = 8.1 Hz, 1H, H_7), 7.26 – 7.21 (m, 1H, H_8), 7.16 – 7.09 (m, 2H, $H_{9,5}$), 6.99 (d, J = 9.1 Hz, 2H, H_{11}), 5.16 (d, J = 7.8 Hz, 1H, H_2), 4.86 (q, J = 6.5 Hz, 1H, H_3), 3.51 – 3.33 (m, 2H, H_4), 1.45 (s, 9H, H_1). **^{13}C NMR** (101 MHz, $CDCl_3$, 298 K) δ 170.57, 155.44, 155.21, 145.53, 136.34, 127.50, 125.24, 123.05, 122.75, 122.44, 120.13, 118.91, 111.52, 109.81, 80.60, 54.71, 28.43, 28.05. **HRMS** (ESI$^+$) calculated for $C_{22}H_{23}N_3O_6Na$ $[M+Na]^+$ = 448.1485, found 448.1514.

Synthesis of **1g**

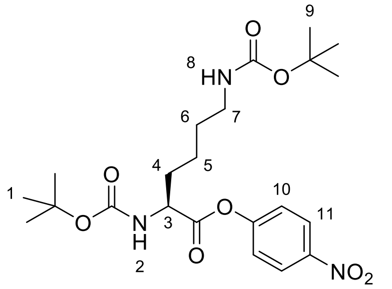

A solution of Boc-Lys(Boc)-OH (450 mg, 1.3 mmol, 1.0 eq.), 4-nitrophenol (222 mg, 1.6 mmol, 1.2 eq.), EDC·HCl (368 mg, 1.9 mmol, 1.5 eq.) and DMAP (31 mg, 0.26 mmol, 0.2 eq.) in CH_2Cl_2 (15 mL) was stirred at room temperature for 16 hours. The reaction mixture was concentrated under reduced pressure. Purification of the crude residue by flash column chromatography (SiO_2, EtOAc/hexane 1:5) afforded **1g** (413 mg, 0.9 mmol, 68%) as a yellow solid. **^{1}H NMR** (400 MHz, $CDCl_3$, 298 K) δ 8.27 (d, J = 8.9 Hz, 2H, H_{11}), 7.30 (d, J = 9.1 Hz, 2H, H_{10}), 5.29 (d, J = 6.9 Hz, 1H, H_2), 4.61 (s, 1H, H_8), 4.45 (q, J = 7.2 Hz, 1H, H_3), 3.23 – 3.05 (m, 2H, H_7), 2.04 – 1.92 (m, 1H, H_{4a}), 1.91 – 1.76 (m, 1H, H_{4b}), 1.63 – 1.38 (m, 4H, $H_{5,6}$), 1.46 (s, 9H, H_1), 1.44 (s, 9H, H_9). **^{13}C NMR** (101 MHz, $CDCl_3$, 298 K) δ 170.97, 156.45, 155.79, 155.35, 145.58, 125.38, 122.46, 80.48, 79.49, 53.94, 39.71, 31.45, 29.86, 28.55, 28.42, 22.58. **HRMS** (ESI^+) calculated for $C_{22}H_{33}N_3O_8Na$ $[M+Na]^+$ = 490.2165, found 490.2189.

Synthesis of **1h**

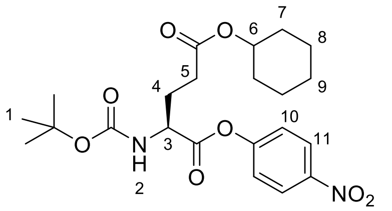

A solution of Boc-Glu(OcHx)-OH (428 mg, 1.3 mmol, 1.0 eq.), 4-nitrophenol (222 mg, 1.6 mmol, 1.2 eq.), EDC·HCl (368 mg, 1.9 mmol, 1.5 eq.) and DMAP (31 mg, 0.26 mmol, 0.2 eq.) in CH_2Cl_2 (15 mL) was stirred at room temperature for 16 hours. The reaction mixture was concentrated under reduced pressure. Purification of the crude residue by flash column chromatography (SiO_2, EtOAc/hexane 1:4) afforded **1h** (440 mg, 1.0 mmol, 77%) as a colorless solid. **^{1}H NMR** (400 MHz, $CDCl_3$, 298 K) δ 8.26 (d, J = 9.1 Hz, 2H, H_{11}), 7.30 (d, J = 9.1 Hz, 2H, H_{10}), 5.26 (d, J = 8.3 Hz, 1H, H_2), 4.76 (tt, J = 8.9, 3.8 Hz, 1H, H_6), 4.52 (td, J = 8.4, 4.9 Hz, 1H, H_3), 2.56 – 2.42 (m, 2H, H_5), 2.37 – 2.25 (m, 1H, H_{4a}), 2.21 – 2.06 (m, 1H, H_{4b}), 1.88 – 1.75 (m, 2H, H_{7a}), 1.74 – 1.63 (m, 2H, H_{8a}), 1.58 – 1.44 (m, 13H, $H_{1,9,7b,8b}$). **^{13}C NMR** (101 MHz, $CDCl_3$, 298 K) δ 172.16, 170.42, 155.55, 155.23, 145.61, 125.35, 122.44, 80.59, 73.44, 53.56, 31.66, 30.76, 28.36, 27.01, 25.39, 23.81. **HRMS** (ESI^+) calculated for $C_{22}H_{30}N_2O_8Na$ $[M+Na]^+$ = 473.1900, found 473.1895.

Synthesis of **1i**

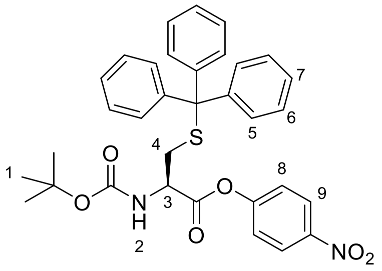

A solution of Boc-Cys(Trt)-OH (220 mg, 0.47 mmol, 1.0 eq.), 4-nitrophenol (80 mg, 0.56 mmol, 1.2 eq.), EDC·HCl (90 mg, 0.70 mmol, 1.5 eq.) and DMAP (11.5 mg, 0.09 mmol, 0.2 eq.) in CH_2Cl_2 (5.5 mL) was stirred at room temperature for 16 hours. The reaction mixture was concentrated under reduced pressure. Purification of the crude residue by flash column chromatography (SiO_2, EtOAc/hexane 1:4) afforded **1i** (168 mg, 0.29 mmol, 61%) as a yellow solid. **^{1}H NMR** (600 MHz, $CDCl_3$, 298 K) δ 8.26 (d, J = 9.1 Hz, 2H, H_9), 7.44 – 7.40 (m, 6H, H_5), 7.32 – 7.26 (m, 6H, H_6), 7.25 7.21 (m, 5H, $H_{7,9}$), 5.07 (d, J = 8.4 Hz, 1H, H_2), 4.42 – 4.36 (m, H_3), 2.82 (dd, J = 12.6, 6.1 Hz , 1H, H_{4a}), 2.70 (dd, J = 12.7, 4.7 Hz, H_{4b}) 1.45 (s, 9H, H_1). **^{13}C NMR** ($CDCl_3$, 101 MHz, 298 K) δ 168.98, 155.28, 155.19, 149.69, 144.20, 129.59, 128.29, 127.22, 125.39, 122.46, 80.73, 67.39, 53.05, 33.86, 28.42. **HRMS** (ESI$^+$) calculated for $C_{33}H_{32}N_2O_6SNa$ $[M+Na]^+$ = 607.1873, found 607.1900.

3.2. Synthesis of dipeptides 3a·CF_3CO_2H – 3e·CF_3CO_2H

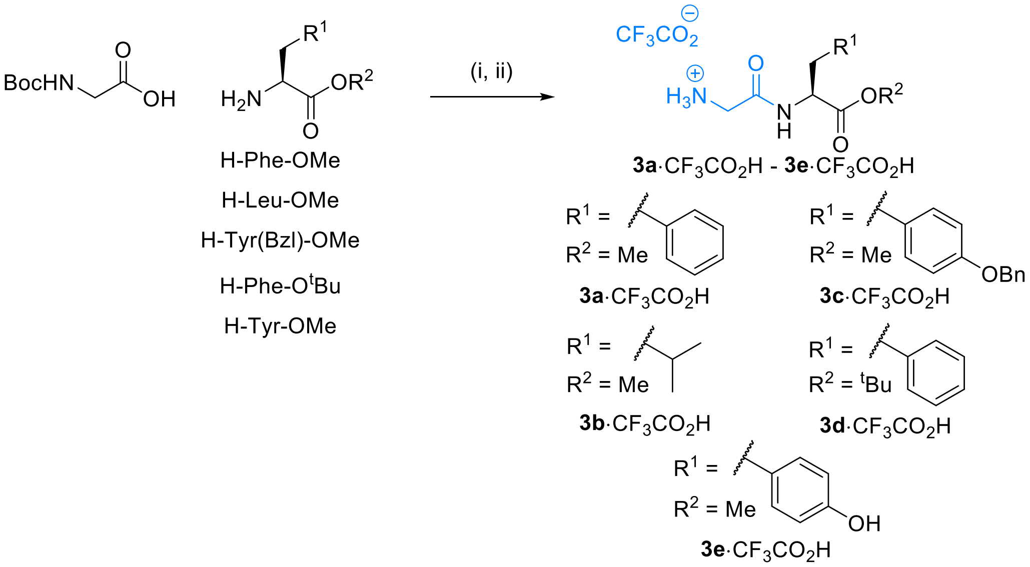

Scheme S2. Reagents and conditions: (i) EDC·HCl, HOBt·H_2O, CH_2Cl_2, rt, 16 h (ii) CF_3CO_2H, CH_2Cl_2, rt, 2 h, 60 – 80%.

Synthesis of **3a**·CF_3CO_2H

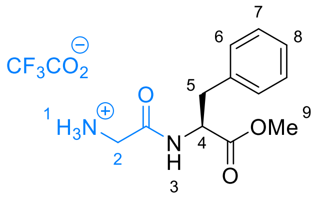

A solution of H-Phe-OMe·HCl (950 mg, 4.4 mmol, 1.0 eq.), Boc-Gly-OH (924 mg, 5.3 mmol, 1.2 eq.), EDC·HCl (1.2 g, 6.6 mmol, 1.5 eq.) HOBt·H_2O (811 mg, 5.3 mmol, 1.2 eq.) and Et_3N (2.5 mL, 17.6 mmol, 4.0 eq.) in CH_2Cl_2 (25 mL) was stirred at room temperature for 16 hours. The reaction mixture was concentrated under reduced pressure. Purification of the crude residue by flash column chromatography (SiO_2, EtOAc/hexane 1:3) afforded a colorless liquid. The dipeptide was taken up in a solution of CF_3CO_2H (5 mL) in CH_2Cl_2 (20 mL), and the reaction mixture was stirred for 2 hours at room temperature. The solvents were removed under reduced pressure to afford **3a**·CF_3CO_2H (1.0 g, 2.9 mmol, 66%) as a colorless solid. **^{1}H NMR** (DMSO-*d*$_6$, 400 MHz, 298 K) δ 8.82 (d, J = 7.8 Hz, 1H, H_3), 7.33 – 7.16 (m, 5H, $H_{6,7,8}$), 4.55 (td, J = 8.4, 5.7 Hz, 1H, H_4), 3.61 (s, 3H, H_9), 3.50 – 3.39 (m, 2H, H_2), 3.05 (dd, J = 13.8, 5.5 Hz, 1H, H_{5a}), 2.92 (dd, J = 13.8, 8.8 Hz, 1H, H_{5b}). **^{13}C NMR** (DMSO-*d*$_6$, 101 MHz, 298 K) δ 171.65, 167.58, 136.87, 129.19, 128.45, 126.82, 53.74, 52.13, 40.86, 36.81. **HRMS** (ESI$^+$) calculated for $C_{12}H_{17}N_2O_3$ [M-CF_3CO_2]$^+$ = 237.1239, found 237.1249.

Synthesis of **3b**·CF_3CO_2H

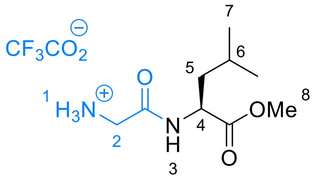

A solution of H-Leu-OMe·HCl (800 mg, 4.4 mmol, 1.0 eq.), Boc-Gly-OH (924 mg, 5.3 mmol, 1.2 eq.), EDC·HCl (1.2 g, 6.6 mmol, 1.5 eq.), HOBt·H_2O (811 mg, 5.3 mmol, 1.2 eq.) and Et_3N (2.5 mL, 17.6 mmol, 4.0 eq.) in CH_2Cl_2 (25 mL) was stirred at room temperature for 16 hours. The reaction mixture was concentrated under reduced pressure. Purification of the crude residue by flash column chromatography (SiO_2, EtOAc/hexane 1:3) afforded a colorless liquid which was used directly. The dipeptide was taken up in a solution of CF_3CO_2H (5 mL) in CH_2Cl_2 (20 mL), and the reaction mixture was stirred for 2 hours at room temperature. The solvents were removed under reduced pressure to afford **3b**·CF_3CO_2H (1.0 g, 3.2 mmol, 72%) as a colorless solid. **^{1}H NMR** (DMSO-d_6, 400 MHz, 298 K) δ 8.88 – 8.87 (mi, 1H, H_3), 8.21 – 8.00 (m, 3H, H_1), 4.35 (td, J = 8.8, 6.2 Hz, 1H, H_4), 3.73 – 3.54 (m, 5H, $H_{2,8}$), 1.68 – 1.45 (m, 3H, $H_{5,6}$), 0.92 – 0.88 (m, 6H, H_7). **^{13}C NMR** (DMSO-d_6, 101 MHz, 298 K) δ 172.54, 166.21, 52.10, 52.06, 50.42, 24.16, 22.74, 21.26. **HRMS** (ESI$^+$) calculated for $C_9H_{19}N_2O_3$ $[M\text{-}CF_3CO_2]^+$ = 203.1396, found 203.1378.

Synthesis of **3c**·CF_3CO_2H

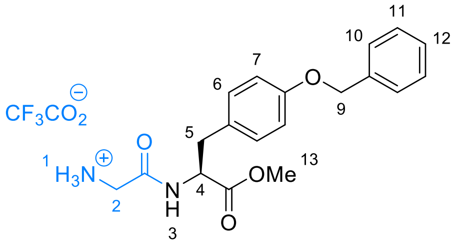

A solution of H-Tyr(Bzl)-OMe·HCl (1.4 g, 4.4 mmol, 1.0 eq.), Boc-Gly-OH (924 mg, 5.3 mmol, 1.2 eq.), EDC·HCl (1.2 g, 6.6 mmol, 1.5 eq.), HOBt·H_2O (811 mg, 5.3 mmol, 1.2 eq.) and Et_3N (2.5 mL, 17.6 mmol, 4.0 eq.) in CH_2Cl_2 (25 mL) was stirred at room temperature for 16 hours. The reaction mixture was concentrated under reduced pressure. Purification of the crude residue by flash column chromatography (SiO_2, EtOAc/hexane 1:3) afforded a colorless liquid which was used directly. The dipeptide was taken up in a solution of CF_3CO_2H (5 mL) in CH_2Cl_2 (20 mL), and reaction mixture was stirred for 2 hours at room temperature. The solvents were removed under reduced pressure to afford **3c**·CF_3CO_2H (1.3 g, 2.8 mmol, 65%) as a colorless solid. **^{1}H NMR** (DMSO-d_6, 400 MHz, 298 K) δ 8.95 – 8.85 (m, 1H, H_3), 8.05 (brs, 3H, H_1), 7.47 – 7.29 (m, 5H, $H_{10\text{-}12}$), 7.14 (d, J = 8.5 Hz, 2H, H_6), 6.93 (d, J = 8.6 Hz, 2H, H_7), 5.06 (s, 2H, H_9), 4.51 (td, J = 8.4, 5.6 Hz, 1H, H_4), 3.61 (s, 3H, H_{13}), 3.60 – 3.47 (m, 2H, H_2), 2.99 (dd, J = 13.9, 5.5 Hz, 1H, H_{5a}), 2.85 (dd, J = 13.9, 8.8 Hz, 1H, H_{5b}). **^{13}C NMR** (DMSO-d_6, 101 MHz, 298 K) δ 171.58, 166.14, 157.24, 137.17, 130.23, 128.83, 128.48, 127.87, 127.72, 114.68, 69.15, 54.02, 52.08, 40.03, 35.97. **HRMS** (ESI$^+$) calculated for $C_{19}H_{22}N_2O_4Na$ $[M+Na\text{-}CF_3CO_2H]^+$ = 365.1477, found 365.1494.

Synthesis of **3d**·CF_3CO_2H

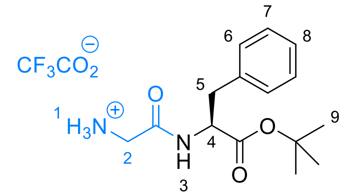

A solution of H-Phe-O^{t}Bu·HCl (1.1 g, 4.4 mmol, 1.0 eq.), Boc-Gly-OH (924 mg, 5.3 mmol, 1.2 eq.), EDC·HCl (1.2 g, 6.6 mmol, 1.5 eq.), HOBt·H_2O (811 mg, 5.3 mmol, 1.2 eq.) and Et_3N (2.5 mL, 17.6 mmol, 4.0 eq.) in CH_2Cl_2 (20 mL) was stirred at room temperature for 16 hours. The reaction mixture was concentrated under reduced pressure. Purification by flash column chromatography (SiO_2, EtOAc/hexane 1:3) afforded a colorless liquid which was used directly. The dipeptide was taken up in a solution of CF_3CO_2H (5 mL) in CH_2Cl_2 (20 mL), and the reaction mixture was stirred for 2 hours at room temperature. The solvents were removed under reduced pressure to afford **3d**·CF_3CO_2H (1.0 g, 2.6 mmol, 59%) as a colorless solid. **^{1}H NMR** (DMSO-d_6, 400 MHz, 298 K) δ 8.89 – 8.82 (m, 1H, H_3), 8.13 (bs, 3H, H_1), 7.33 – 7.27 (m, 2H, H_7), 7.25 – 7.19 (m, 3H, $H_{6,8}$), 4.44 (td, J = 8.0, 6.4 Hz, 1H, H_4), 3.65 – 3.49 (m, 2H, H_2), 3.04 – 2.85 (m, 2H, H_5), 1.32 (s, 9H, H_9). ^{13}C NMR (DMSO-d_6, 101 MHz, 298 K) δ 170.19, 166.08, 136.88, 129.29, 128.33, 126.74, 81.17, 54.37, 40.04, 37.09, 27.55. **HRMS** (ESI$^+$) calculated for $C_{15}H_{23}N_2O_3$ [M-CF_3CO_2]$^+$ = 279.1709, found 279.1708.

Synthesis of **3e**·CF_3CO_2H

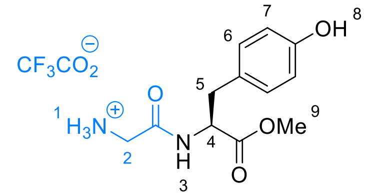

A solution of H-Tyr-OMe·HCl (1.0 g, 4.4 mmol, 1.0 eq.), Boc-Gly-OH (924 mg, 5.3 mmol, 1.2 eq.), EDC·HCl (1.2 g, 6.6 mmol, 1.5 eq.), HOBt·H_2O (811 mg, 5.3 mmol, 1.2 eq.) and Et_3N (2.5 mL, 17.6 mmol, 4.0 eq.) in CH_2Cl_2 (25 mL) was stirred at room temperature for 16 hours. The reaction mixture was concentrated under reduced pressure. Purification of the crude residue by flash column chromatography (SiO_2, EtOAc/hexane 1:4) afforded a colorless liquid which was used directly. The dipeptide was taken up in a solution of CF_3CO_2H (5 mL) in CH_2Cl_2 (20 mL), and reaction mixture was stirred for 2 hours at room temperature. The solvents were removed under reduced pressure to afford **3e**·CF_3CO_2H (1.0 g, 2.7 mmol, 62%) as a colorless solid. **^{1}H NMR** (DMSO-d_6, 400 MHz, 298 K) δ 8.85 (d, J = 7.7 Hz, 1H, H_3), 8.05 (brs, 3H, H_1), 6.99 (d, J = 8.3 Hz, 2H, H_6), 6.67 (d, J = 8.4 Hz, 2H, H_7), 4.47 (td, J = 8.2, 5.6 Hz, 1H, H_4), 3.64 – 3.48 (m, 5H, $H_{2,9}$), 2.93 (dd, J = 13.9, 5.5 Hz, 1H, H_{5a}), 2.79 (dd, J = 13.9, 8.7 Hz, 1H, H_{5b}). **^{13}C NMR** (DMSO-d_6, 101 MHz, 298 K) δ 171.71, 166.14, 156.28, 130.15, 126.72, 115.25, 54.19, 52.10, 36.14. **HRMS** (ESI$^+$) calculated for $C_{12}H_{17}N_2O_4Na$ [M-CF_3CO_2]$^+$ = 253.1188, found 253.1176.

3.3. Synthesis of tripeptide based-rotaxanes 24C8⊂4 – 24C8⊂15

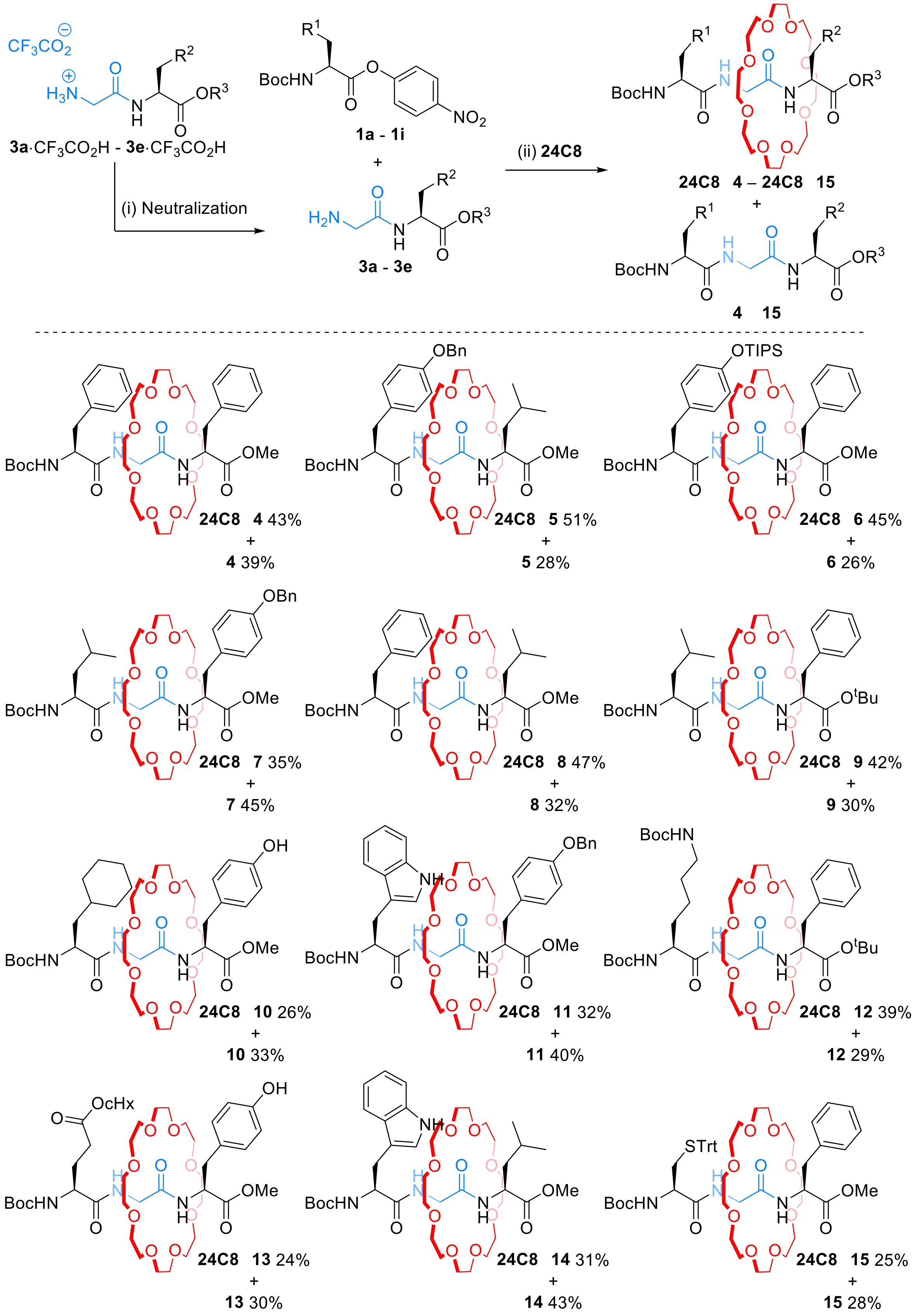

Scheme S3. Reagents and conditions: (i) $NaHCO_3$ (sat. aq.), CH_2Cl_2. (ii) 24C8, toluene, rt, 16 h, 24 – 51%.

General procedure 1: Neutralization of amino acid CF_3CO_2H salts.

The amine CF_3CO_2H salt was dissolved in CH_2Cl_2 (10 mL) and washed with a solution of saturated aqueous $NaHCO_3$ (5 mL). The organic layer was separated, dried (Na_2SO_4) and the solvent removed under reduced pressure. The neutralized amine was used immediately in the rotaxane forming reaction. Prolonged storage of the neutralized amines leads to the formation of insoluble precipitates.

Synthesis of **24C8⊂4** and **4**

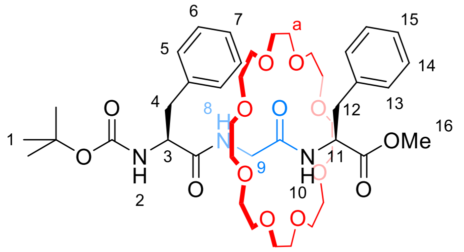

To a stirred solution of 24-crown-8 (49 mg, 0.14 mmol, 1.0 eq.) and **3a** (33 mg, 0.14 mmol, 1.0 eq., obtained from **3a**·CF_3CO_2H, using general procedure 1) in toluene (1.0 mL) was added **1a** (54 mg, 0.14 mmol, 1.0 eq.). The mixture was stirred at room temperature for 16 hours. The reaction mixture was concentrated under reduced pressure. Purification of the crude residue by prep TLC (SiO_2, EtOAc/hexane (1:1→10:1, v/v)) afforded **24C8⊂4** (50 mg, 0.060 mmol, 43%) as a colorless solid and non-interlocked tripeptide **4** (27 mg, 0.056 mmol, 39%) as a colorless solid. **^{1}H NMR** (400 MHz, $CDCl_3$, 298 K) δ 8.04 (t, J = 4.6 Hz, 1H, H_8), 7.64 (d, J = 7.3 Hz, 1H, H_{10}), 7.31 – 7.14 (m, 10H, $H_{5\text{-}7,13\text{-}15}$), 5.38 (d, J = 9.2 Hz, 1H, H_2), 4.83 – 4.72 (m, 1H, H_{11}), 4.66 (dt, J = 9.6, 4.8 Hz, 1H, H_3), 4.49 (dd, J = 17.7, 5.1 Hz, 1H, H_{9a}), 4.22 (dd, J = 17.5, 3.9 Hz, 1H, H_{9b}), 3.71 – 3.54 (m, 32H, H_a), 3.53 (s, 3H, H_{16}), 3.29 (dd, J = 13.5, 3.9 Hz, 1H, H_{4a}), 3.14 (dd, J = 13.5, 5.2 Hz, 1H, H_{12a}), 2.97 (dd, J = 13.5, 8.9 Hz, 1H, H_{12b}), 2.68 (dd, J = 13.5, 9.9 Hz, 1H_{4b}), 1.24 (s, 9H, H_1). **^{13}C NMR** (101 MHz, $CDCl_3$, 298 K) δ 172.59, 171.20, 170.01, 154.76, 138.50, 137.04, 129.94, 129.41, 128.49, 128.05, 126.75, 126.14, 78.36, 70.74, 70.65, 54.69, 54.39, 51.72, 43.44, 39.97, 38.77, 28.34. **HRMS** (ESI$^+$) calculated for $C_{42}H_{65}N_3O_{14}Na$ $[M+Na]^+$ = 858.4359, found 858.4355.

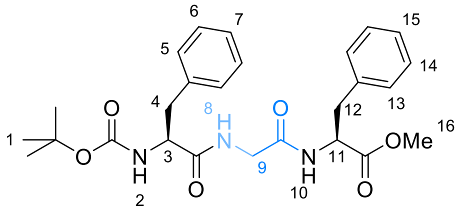

^{1}H NMR (400 MHz, $CDCl_3$, 298 K) δ 7.20 (m, 11H, $H_{5\text{-}7,13\text{-}14,10}$), 7.06 (t, J = 5.4 Hz, 1H, H_8), 5.32 (d, J = 8.0 Hz, 1H, H_2), 4.82 (q, J = 6.8 Hz, 1H, H_{11}), 4.41 (q, J = 7.3 Hz, 1H, H_3), 3.97 (dd, J = 16.8, 5.6 Hz, 1H, H_{9a}), 3.76 (dd, J = 16.7, 5.0 Hz, 1H, H_{9b}), 3.67 (s, 3H, H_{16}), 3.15 – 2.99 (m, 3H, $H_{12,4a}$), 2.94 (dd, J = 14.4, 8.1 Hz, 1H, H_{4b}), 1.36 (s, 9H, H_1). **^{13}C NMR** (101 MHz, $CDCl_3$, 298 K) δ 172.06, 171.90, 168.47, 155.54, 136.72, 135.94, 129.30, 129.27, 128.61, 128.50, 127.15, 126.91, 80.14, 55.82, 53.56, 52.40, 43.00, 38.44, 37.89, 28.31. **HRMS** (ESI$^+$) calculated for $C_{26}H_{34}N_3O_6$ $[M+H]^+$ = 484.2448, found 484.2452.

Synthesis of **24C8⊂5** and **5**

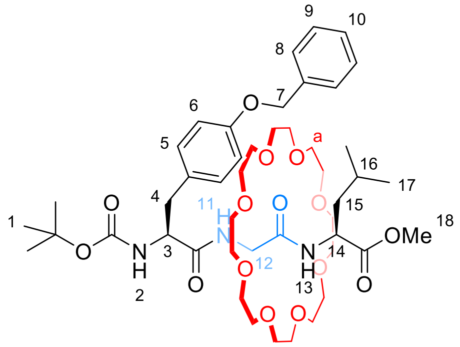

To a stirred solution of 24-crown-8 (49 mg, 0.14 mmol, 1.0 eq.) and **3b** (28 mg, 0.14 mmol, 1.0 eq., obtained from **3b**·CF_3CO_2H, using general procedure 1) in toluene (1.0 mL) was added **1b** (69 mg, 0.14 mmol, 1.0 eq.). The mixture was stirred at room temperature for 16 hours. The reaction mixture was concentrated under reduced pressure. Purification of the crude residue by prep TLC (SiO_2, EtOAc/hexane (1:1→10:1, v/v)) afforded **24C8⊂5** (65 mg, 0.072 mmol, 51%) as a colorless solid and non-interlocked tripeptide **5** (22 mg, 0.040 mmol, 28%) as a colorless solid. **^{1}H NMR** (400 MHz, $CDCl_3$, 298 K) δ 8.01 (t, J = 4.5 Hz, 1H, H_{11}), 7.46 – 7.28 (m, 6H, $H_{8-10,13}$), 7.19 (d, J = 8.1 Hz, 2H, H_5), 6.84 (d, J = 8.0 Hz, 2H, H_6), 5.35 (d, J = 9.0 Hz, 1H, H_2), 5.05 – 4.93 (m, 2H, H_7), 4.65 – 4.49 (m, 2H, $H_{3,14}$), 4.40 (dd, J = 17.6, 4.7 Hz, 1H, H_{12a}), 4.16 (dd, J = 17.6, 4.4 Hz, 1H, H_{12b}), 3.72 – 3.57 (m, 35H, $H_{18,a}$), 3.19 (dd, J = 13.6, 3.9 Hz, 1H, H_{4a}), 2.59 (dd, J = 13.5, 9.8 Hz, 1H, H_{4b}), 1.78 (septet, J = 6.8 Hz, 1H, H_{16}), 1.54 (t, J = 7.2 Hz, 2H, H_{15}), 1.24 (s, 9H, H_1), 0.92 – 0.82 (m, 6H, H_{17}). **^{13}C NMR** (101 MHz, $CDCl_3$, 298 K) δ 173.84, 171.19, 169.85, 157.34, 154.71, 137.44, 130.90, 130.81, 128.56, 127.84, 127.48, 114.39, 78.25, 70.71, 70.58, 69.97, 54.70, 51.83, 50.69, 43.32, 41.67, 38.93, 28.32, 24.07, 22.89, 22.31. **HRMS** (ESI$^+$) calculated for $C_{46}H_{75}N_3O_{15}Na$ $[M+Na]^+$ = 930.4939, found 930.4932.

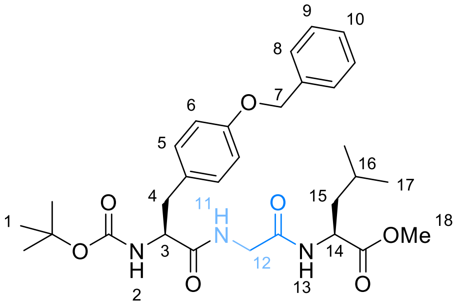

^{1}H NMR (400 MHz, $CDCl_3$, 298 K) δ 7.46 – 7.29 (m, 5H, H_{8-10}), 7.23 – 7.15 (m, 1H, H_{13}), 7.12 – 7.01 (m, 3H, $H_{5,11}$), 6.89 (d, J = 8.3 Hz, 2H, H_6), 5.27 – 5.15 (m, 1H, H_2), 5.01 (s, 2H, H_7), 4.57 (dt, J = 8.3, 4.9 Hz, 1H, H_{14}), 4.31 (q, J = 7.0 Hz, 1H, H_3), 4.08 (dd, J = 16.8, 5.8 Hz, 1H, H_{12a}), 3.88 – 3.77 (m, 1H, H_{12b}), 3.69 (s, 3H, H_{18}), 3.08 (dd, J = 14.0, 5.8 Hz, 1H, H_{4a}), 2.93 (dd, J = 13.9, 7.7 Hz, 1H, H_{4b}), 1.70 – 1.54 (m, 3H, $H_{15,16}$), 1.37 (s, 9H, H_1), 0.95 – 0.88 (m, 6H, H_{17}). **^{13}C NMR** (101 MHz, $CDCl_3$, 298 K) δ 173.34, 172.15, 168.77, 157.84, 155.65, 136.99, 130.34, 128.86, 128.63, 128.03, 127.55, 115.03, 80.31, 70.01, 56.21, 52.38, 50.92, 43.10, 41.04, 37.25, 28.33, 24.83, 22.85, 21.89. **HRMS** (ESI$^+$) calculated for $C_{30}H_{42}N_3O_7$ $[M+H]^+$ = 556.3023, found 556.3025.

Synthesis of **24C8⊂6** and **6**

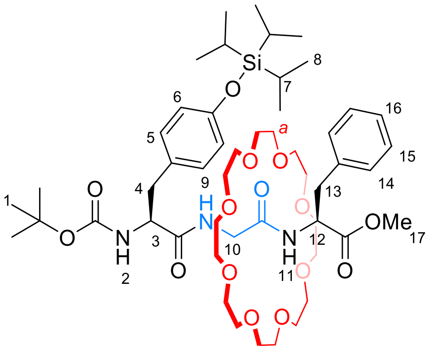

To a stirred solution of 24-crown-8 (49 mg, 0.14 mmol, 1.0 eq.) and **3a** (33 mg, 0.14 mmol, 1.0 eq., obtained from **3a**·CF_3CO_2H, using general procedure 1) in toluene (1.0 mL) was added **1c** (78 mg, 0.14 mmol, 1.0 eq.). The mixture was stirred at room temperature for 16 hours. The reaction mixture was concentrated under reduced pressure. Purification of the crude residue by prep TLC (SiO_2, EtOAc/hexane (1:1→10:1, v/v)) afforded **24C8⊂6** (63 mg, 0.063 mmol, 45%) as a colorless oil and **6** (24 mg, 0.037 mmol, 26%) as a colorless oil. **^{1}H NMR** (500 MHz, $CDCl_3$, 298 K) δ 7.99 (t, *J* = 4.6 Hz, 1H, H_9), 7.61 (d, *J* = 7.3 Hz, 1H, H_{11}), 7.24 (d, *J* = 7.0 Hz, 2H, H_{14}), 7.21 – 7.15 (m, 3H, $H_{15,16}$), 7.10 (d, *J* = 8.2 Hz, 2H, H_5), 6.73 (d, *J* = 8.0 Hz, 2H, H_6), 5.34 (d, *J* = 9.2 Hz, 1H, H_2), 4.81 – 4.71 (m, 1H, H_{12}), 4.62 (dt, *J* = 9.4, 4.1 Hz, 1H, H_3), 4.47 (dd, *J* = 17.6, 5.3 Hz, 1H, H_{10a}), 4.19 (dd, *J* = 17.5, 3.8 Hz, 1H, H_{10b}), 3.73 – 3.54 (m, 32H, H_a), 3.51 (s, 3H, H_{17}), 3.20 (dd, *J* = 13.8, 4.1 Hz, 1H, H_{4a}), 3.13 (dd, *J* = 13.5, 5.3 Hz, 1H, H_{13a}), 2.95 (dd, *J* = 13.5, 8.9 Hz, 1H, H_{13b}), 2.62 (dd, *J* = 13.9, 9.7 Hz, 1H, H_{4b}), 1.25 (s, 9H, H_1), 1.23 – 1.16 (m, 3H, H_7), 1.06 (d, *J* = 7.4 Hz, 18H, H_8). **^{13}C NMR** (125 MHz, $CDCl_3$, 298 K) δ 172.53, 171.40, 169.94, 154.76, 154.34, 137.02, 130.92, 130.64, 130.63, 129.34, 129.28, 128.41, 126.67, 119.28, 119.24, 78.21, 70.68, 70.61, 70.56, 70.54, 54.55, 54.31, 51.62, 43.37, 38.96, 38.72, 28.33, 28.07, 18.02, 12.76, 12.72. **HRMS** (ESI$^+$) Calculated for $C_{51}H_{86}O_{15}N_3Si$ $[M+H]^+$ = 1008.5823, found 1008.5794.

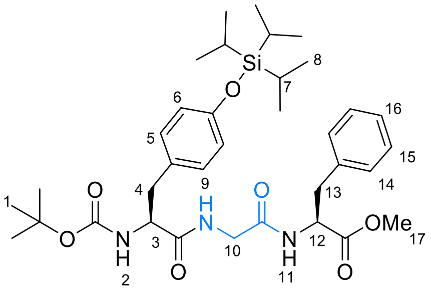

^{1}H NMR (500 MHz, $CDCl_3$, 298 K) δ 7.28 – 7.23 (m, 2H, H_{15}), 7.23 – 7.16 (m, 1H, H_{16}), 7.11 (d, *J* = 6.9 Hz, 2H, H_{14}), 7.04 – 6.94 (m, 3H, $H_{5,11}$), 6.84 (t, *J* = 5.4 Hz, 1H, H_9), 6.81 – 6.74 (m, 2H, H_6), 5.12 (d, *J* = 7.5 Hz, 1H, H_2), 4.81 (dt, *J* = 7.9, 6.4 Hz, 1H, H_{12}), 4.36 – 4.22 (m, 1H, H_3), 3.99 (dd, *J* = 16.8, 5.8 Hz, 1H, H_{10a}), 3.73 (dd, *J* = 16.9, 5.0 Hz, 1H, H_{10b}), 3.66 (s, 3H, H_{17}), 3.12 (dd, *J* = 13.9, 6.0 Hz, 1H, H_{13a}), 3.07 – 2.97 (m, 2H, $H_{4a,13b}$), 2.87 (dd, *J* = 13.9, 8.0 Hz, 1H, H_{4b}), 1.37 (s, 9H, H_1), 1.26 – 1.17 (m, 3H, H_7), 1.07 (d, *J* = 7.4 Hz, 18H, H_8). **^{13}C NMR** (125 MHz, $CDCl_3$, 298 K) δ 172.19, 171.88, 168.45, 155.63, 155.11, 135.97, 130.22, 129.31, 128.97, 128.66, 127.18, 120.15, 80.23, 56.13, 53.55, 52.39, 43.06, 37.92, 37.51, 28.34, 17.98, 12.70. **HRMS** (ESI$^+$) Calculated for $C_{35}H_{54}O_7N_3Si$ $[M+H]^+$ = 656.3726, found 656.3732.

Synthesis of **24C8⊂7** and **7**

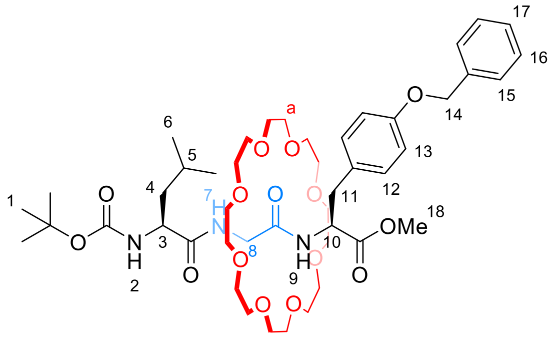

To a stirred solution of 24-crown-8 (49 mg, 0.14 mmol, 1.0 eq.) and **3c** (48 mg, 0.14 mmol, 1.0 eq., obtained from **3c**·CF_3CO_2H, using general procedure 1) in toluene (1.0 mL) was added **1d** (49 mg, 0.14 mmol, 1.0 eq.). The mixture was stirred at room temperature for 16 hours. The reaction mixture was concentrated under reduced pressure. Purification of the crude residue by prep TLC (SiO_2, EtOAc/hexane (1:1→10:1, v/v)) afforded **24C8⊂7** (44 mg, 0.049 mmol, 35%) as a colorless solid and non-interlocked tripeptide **7** (35 mg, 0.063 mmol, 45%) as a colorless solid. **^{1}H NMR** (400 MHz, $CDCl_3$, 298 K) δ 7.84 (t, J = 4.6 Hz, 1H, H_7), 7.62 (d, J = 6.7 Hz, 1H, H_9), 7.45 – 7.26 (m, 5H, $H_{15\text{-}17}$), 7.10 (d, J = 8.4 Hz, 2H, H_{12}), 6.87 (d, J = 8.6 Hz, 2H, H_{13}), 5.42 (d, J = 9.2 Hz, 1H, H_2), 5.02 (s, 2H, H_{14}), 4.67 (dt, J = 8.7, 5.5 Hz, 1H, H_{10}), 4.54 – 4.40 (m, 2H, $H_{3,8a}$), 4.11 (dd, J = 17.6, 3.4 Hz, 1H, H_{8b}), 3.67 – 3.49 (m, 35H, $H_{18,a}$), 3.08 (dd, J = 13.4, 4.9 Hz 1H, H_{11a}), 2.89 (dd, J = 13.6, 9.0 Hz, 1H, H_{11b}), 1.75 – 1.63 (m, 1H, H_5), 1.62 – 1.54 (m, 1H, H_{4a}), 1.39 (m, 10H, $H_{1,4b}$), 1.02 (d, J = 6.3 Hz, 3H, H_{6a}), 0.90 (d, J = 6.5 Hz, 3H, H_{6b}). **^{13}C NMR** (101 MHz, $CDCl_3$, 298 K) δ 173.27, 173.05, 170.65, 158.20, 155.91, 137.74, 131.01, 129.95, 129.25, 128.62, 128.13, 115.42, 79.07, 78.05, 71.17, 70.62, 55.23, 52.87, 52.28, 44.19, 44.11, 38.37, 29.08, 25.32, 24.74, 22.67. **HRMS** (ESI$^+$) calculated for $C_{46}H_{73}N_3O_{15}Na$ $[M+Na]^+$ = 930.4934, found 930.4932.

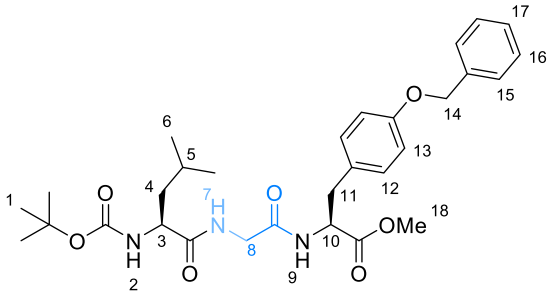

^{1}H NMR (400 MHz, $CDCl_3$, 298 K) δ 7.46 – 7.28 (m, 5H, $H_{15\text{-}17}$), 7.08 – 6.99 (m, 3H, $H_{7,12}$), 6.97 – 6.85 (m, 3H, $H_{9,13}$), 5.09 (s, 1H, H_2), 5.01 (s, 2H, H_{14}), 4.78 (q, J = 6.6 Hz, 1H, H_{10}), 4.15 (m, 1H, H_3), 4.01 (dd, J = 17.2, 5.6 Hz, 1H, H_{8a}), 3.85 (dd, J = 16.9, 4.9 Hz, 1H, H_{8b}), 3.68 (s, 3H, H_{18}), 3.10 – 2.96 (m, 2H, H_{11}), 1.70 – 1.57 (m, 2H, $H_{5,4a}$), 1.54 – 1.35 (m, 10H, $H_{1,4b}$), 0.97 – 0.88 (m, 6H, H_6). **^{13}C NMR** (101 MHz, $CDCl_3$, 298 K) δ 173.46, 171.93, 168.67, 157.98, 155.92, 136.99, 130.39, 128.68, 128.08, 127.64, 114.99, 80.37, 70.00, 53.75, 53.30, 52.50, 43.05, 41.24, 37.07, 28.41, 24.82, 23.14, 21.87. **HRMS** (ESI$^+$) calculated for $C_{30}H_{42}N_3O_7$ $[M+H]^+$ = 556.3023, found 556.3022.

Synthesis of **24C8⊂8** and **8**

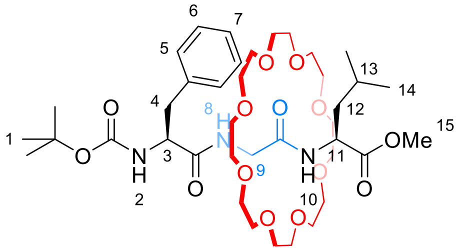

To a stirred solution of 24-crown-8 (49 mg, 0.14 mmol, 1.0 eq.) and **3b** (28 mg, 0.14 mmol, 1.0 eq., obtained from **3b**·CF_3CO_2H, using general procedure 1) in toluene (1.0 mL) was added **1a** (54 mg, 0.14 mmol, 1.0 eq.). The mixture was stirred at room temperature for 16 hours. The reaction mixture was concentrated under reduced pressure. Purification of the crude residue by prep TLC (SiO_2, EtOAc/hexane (1:1→10:1, v/v)) afforded **24C8⊂8** (53 mg, 0.066 mmol, 47%) as a colorless solid and non-interlocked tripeptide **8** (20 mg, 0.045 mmol, 32%) as a colorless solid. **^{1}H NMR** (400 MHz, $CDCl_3$, 298 K) δ 8.01 (t, J = 4.4 Hz, 1H, H_8), 7.37 (d, J = 7.4 Hz, 1H, H_{10}), 7.32 – 7.09 (m, 5H, $H_{5\text{-}7}$), 5.34 (d, J = 9.3 Hz, 1H, H_2), 4.67 – 4.47 (m, 2H, $H_{3,11}$), 4.39 (dd, J = 17.5, 4.6 Hz, 1H, H_{9a}), 4.20 – 4.08 (m, 1H, H_{9b}), 3.69 – 3.55 (m, 35H, $H_{15,\ a}$), 3.24 (dd, J = 13.2, 3.9 Hz, 1H, H_{4a}), 2.69 – 2.53 (m, 1H, H_{4b}), 1.85 – 1.70 (m, 1H, H_{13}), 1.53 (t, J = 7.1 Hz, 2H, H_{12}), 1.20 (s, 9H, H_1), 0.92 – 0.85 (m, 6H, H_{14}). **^{13}C NMR** (101 MHz, $CDCl_3$, 298 K) δ 173.85, 171.11, 169.84, 154.65, 138.55, 129.88, 127.95, 126.02, 78.21, 70.70, 70.57, 54.61, 51.82, 50.67, 43.32, 41.70, 39.85, 28.27, 24.07, 22.90, 22.32. **HRMS** (ESI^+) calculated for $C_{39}H_{68}N_3O_{14}$ $[M+H]^+$ = 802.4696, found 802.4716.

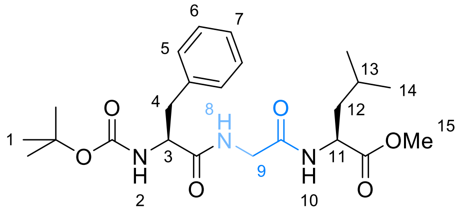

^{1}H NMR (400 MHz, $CDCl_3$, 298 K) δ 7.33 – 7.17 (m, 5H, $H_{5\text{-}7}$), 6.83 (d, J = 8.1 Hz, 1H, H_{10}), 6.74 (t, J = 5.4 Hz, 1H, H_8), 5.04 (d, J = 7.2 Hz, 1H, H_2), 4.57 (td, J = 8.3, 4.8 Hz, 1H, H_{11}), 4.30 (q, J = 7.1 Hz, 1H, H_3), 4.07 (dd, J = 16.8, 6.0 Hz, 1H, H_{9a}), 3.79 (dd, J = 16.6, 5.0 Hz, 1H, H_{9b}), 3.71 (s, 3H, H_{15}), 3.16 (dd, J = 13.9, 6.1 Hz, 1H, H_{4a}), 3.01 (dd, J = 13.8, 7.9 Hz, 1H, H_{4b}), 1.72 – 1.54 (m, 3H, $H_{12,13}$), 1.38 (s, 9H, H_1), 0.97 – 0.86 (m, 6H, H_{14}). **^{13}C NMR** (101 MHz, $CDCl_3$, 298 K) δ 173.31, 171.90, 168.62, 155.70, 136.65, 129.32, 128.87, 127.17, 80.62, 56.34, 52.47, 50.95, 43.16, 41.20, 37.98, 28.37, 24.89, 22.90, 21.96. **HRMS** (ESI^+) calculated for $C_{23}H_{35}N_3O_6Na$ $[M+Na]^+$ = 472.2424, found 472.2429.

Synthesis of **24C8⊂9** and **9**

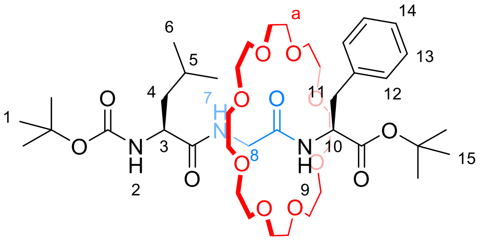

To a stirred solution of 24-crown-8 (49 mg, 0.14 mmol, 1.0 eq.) and **3d** (39 mg, 0.14 mmol, 1.0 eq., obtained from **3d**·CF_3CO_2H, using general procedure 1) in toluene (1.0 mL) was added **1d** (49 mg, 0.14 mmol, 1.0 eq.). The mixture was stirred at room temperature for 16 hours. The reaction mixture was concentrated under reduced pressure. Purification of the crude residue by prep TLC (SiO_2, EtOAc/hexane (1:1→10:1, v/v)) afforded **24C8⊂9** (50 mg, 0.059 mmol, 42%) as a colorless solid and non-interlocked tripeptide **9** (21 mg, 0.043 mmol, 30%) as a colorless solid. **^{1}H NMR** (400 MHz, $CDCl_3$, 298 K) δ 7.90 (t, J = 4.4 Hz, 1H, H_7), 7.57 (d, J = 7.4 Hz, 1H, H_9), 7.26 – 7.16 (m, 5H, H_{12-14}), 5.41 (d, J = 9.2 Hz, 1H, H_2), 4.77 – 4.65 (m, 1H, H_{10}), 4.52 – 4.39 (m, 2H, $H_{3,8a}$), 4.13 (dd, J = 17.6, 3.6 Hz, 1H, H_{8b}), 3.67 – 3.57 (m, 32H, H_a), 3.11 (dd, J = 13.4, 4.9 Hz, 1H, H_{11a}), 2.85 (dd, J = 13.4, 9.7 Hz, 1H, H_{11b}), 1.75 – 1.53 (m, 3H, $H_{4,5}$), 1.39 (s, 9H, H_1), 1.20 (s, 9H, H_{15}), 1.02 (d, J = 6.3 Hz, 3H, H_{6a}), 0.89 (d, J = 6.5 Hz, 3H, H_{6b}). **^{13}C NMR** (101 MHz, $CDCl_3$, 298 K) δ 172.52, 171.24, 169.67, 155.33, 137.24, 129.76, 128.29, 126.59, 81.02, 78.49, 70.73, 70.62, 54.61, 52.29, 43.62, 43.48, 39.57, 28.51, 27.91, 24.76, 24.17, 22.10. **HRMS** (ESI$^+$) calculated for $C_{42}H_{73}N_3O_{14}Na$ $[M+Na]^+$ = 866.4990, found 866.4967.

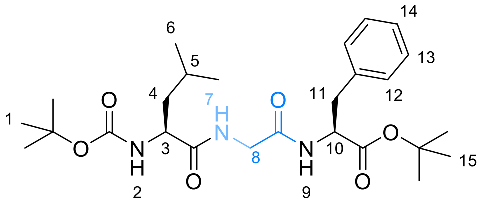

^{1}H NMR (400 MHz, $CDCl_3$, 298 K) δ 7.29 – 7.19 (m, 3H, $H_{13,14}$), 7.18 – 7.11 (m, 2H, H_{12}), 6.82 (t, J = 5.3 Hz, 1H, H_7), 6.65 (d, J = 7.8 Hz, 1H, H_9), 4.95 (d, J = 7.8 Hz, 1H, H_2), 4.73 (dt, J = 7.8, 6.2 Hz, 1H, H_{10}), 4.18 – 4.08 (m, 1H, H_3), 3.94 (dd, J = 16.8, 5.6 Hz, 1H, H_{8a}), 3.85 (dd, J = 16.8, 5.1 Hz, 1H, H_{8b}), 3.07 (d, J = 6.2 Hz, 2H, H_{11}), 1.73 – 1.48 (m, 3H, $H_{4,5}$), 1.43 (s, 9H, H_1), 1.38 (s, 9H, H_{15}), 0.98 – 0.87 (m, 6H, H_6). **^{13}C NMR** (101 MHz, $CDCl_3$, 298 K) δ 173.21, 170.45, 168.25, 155.87, 136.14, 129.60, 128.54, 127.12, 82.58, 80.33, 53.81, 53.31, 43.08, 41.37, 38.19, 28.44, 28.05, 24.85, 23.14, 21.93. **HRMS** (ESI$^+$) calculated for $C_{26}H_{42}N_3O_6$ $[M+H]^+$ = 492.3074, found 492.3065.

Synthesis of **24C8⊂10** and **10**

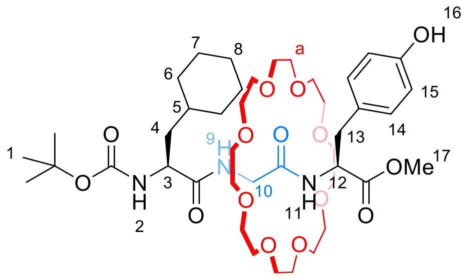

To a stirred solution of 24-crown-8 (49 mg, 0.14 mmol, 1.0 eq.), triethylamine (21 μL, 0.15 mmol, 1.1 eq.) and **3e** (35 mg, 0.14 mmol, 1.0 eq., obtained from **3e**·CF_3CO_2H, using general procedure 1) in toluene (1.0 mL) was added **1e** (55 mg, 0.14 mmol, 1.0 eq.). The mixture was stirred at room temperature for 16 hours. The reaction mixture was concentrated under reduced pressure. Purification of the crude residue by prep TLC (SiO_2, EtOAc/hexane (1:1→10:1, v/v)) afforded **24C8⊂10** (31 mg, 0.036 mmol, 26%) as a colorless solid and the non-interlocked tripeptide **10** (23 mg, 0.046 mmol, 33%) as a colorless solid. **^{1}H NMR** (400 MHz, $CDCl_3$, 298 K) δ 7.89 (t, J = 5.9, 1H, H_9), 7.71 (d, J = 6.6 Hz, 1H, H_{11}), 7.51 (s, 1H, H_{16}), 6.98 (d, J = 8.2 Hz, 2H, H_{14}), 6.76 (d, J = 8.4 Hz, 2H, H_{15}), 5.39 (d, J = 9.4 Hz, 1H, H_2), 4.65 – 4.57 (m, 1H, H_9), 4.56 – 4.44 (m, 2H, $H_{3,10a}$), 4.11 (dd, J = 17.5, 3.4 Hz, 1H, H_{10b}), 3.71 – 3.51 (m, 32H, H_a), 3.47 (s, 3H, H_{17}), 3.04 (dd, J = 13.6, 5.0 Hz, 1H, H_{13a}), 2.82 (dd, J = 13.5, 9.6 Hz, 1H, H_{13b}), 2.85 (d, J = 12.0 Hz, 1H, H_5) 1.75 – 1.55 (m, 5H, $H_{4,6a/7a,8a}$), 1.50 – 0.74 (m, 16H, $H_{1,6a/7a,6b,7b,8b}$). **^{13}C NMR** (101 MHz, $CDCl_3$, 298 K) δ 172.81, 172.72, 170.20, 155.39, 155.31, 130.35, 128.13, 115.53, 78.59, 70.67, 70.61, 54.92, 51.67, 51.59, 43.58, 42.08, 37.83, 34.82, 34.26, 32.63, 28.47, 26.78, 26.76, 26.53. **HRMS** (ESI$^+$) calculated for $C_{42}H_{71}N_3O_{15}Na$ $[M+Na]^+$ = 880.4777, found 880.4758.

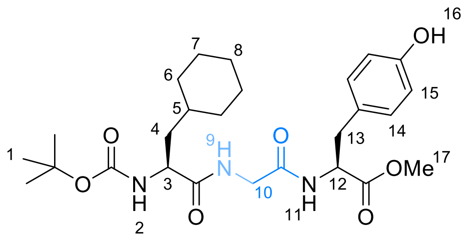

^{1}H NMR (400 MHz, $CDCl_3$, 298 K) δ 7.53 (s, 1H, H_{16}), 7.17 – 7.05 (m, 2H, $H_{9,11}$), 6.91 (d, J = 8.0 Hz, 2H, H_{14}), 6.70 (d, J = 8.1 Hz, 2H, H_{15}), 5.23 (d, J = 7.9 Hz, 1H, H_2), 4.79 (td, J = 7.3, 5.3 Hz, 1H, H_{12}), 4.22 – 4.09 (m, 1H, H_3), 3.93 – 3.67 (m, 5H, $H_{10,17}$), 3.08 (ddd, J = 14.2, 5.2 Hz, 1H, H_{13a}), 2.94 (dd, J = 14.0, 6.9 Hz, 1H, H_{13b}) 1.82 – 1.51 (m, 6H, $H_{4a,6/7,8a}$), 1.50 – 1.38 (m, 10H, $H_{1,4b}$), 1.17 (m, 3H, $H_{5,6/7}$), 1.00 – 0.81 (m, 3H, $H_{6/7,8b}$). **^{13}C NMR** (101 MHz, $CDCl_3$, 298 K) δ 173.83, 172.07, 168.96, 156.15, 155.71, 130.45, 127.01, 115.80, 80.57, 53.67, 52.74, 52.59, 42.93, 40.02, 37.09, 34.17, 33.86, 32.33, 28.45, 26.49, 26.36, 26.13. **HRMS** (ESI$^+$) calculated for $C_{26}H_{39}N_3O_7Na$ $[M+Na]^+$ = 528.2686, found 528.2712.

Synthesis of **24C8⊂11** and **11**

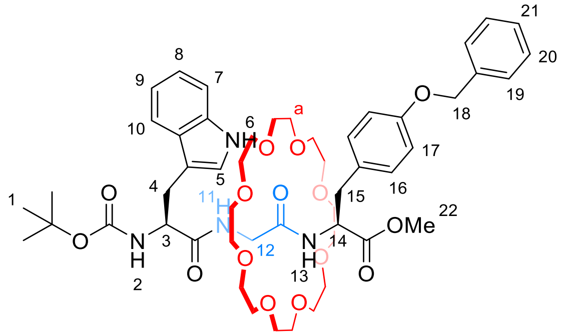

To a stirred solution of 24-crown-8 (49 mg, 0.14 mmol, 1.0 eq.) and **3c** (48 mg, 0.14 mmol, 1.0 eq., obtained from **3c**·CF_3CO_2H, using general procedure 1) in toluene (1.0 mL) was added **1f** (60 mg, 0.14 mmol, 1.0 eq.). The mixture was stirred at room temperature for 16 hours. The reaction mixture was concentrated under reduced pressure. Purification of the crude residue by prep TLC (SiO_2, EtOAc/methanol (50:1→20:1, v/v)) afforded **24C8⊂11** (44 mg, 0.045 mmol, 32%) as a colorless solid and non-interlocked tripeptide **11** (35 mg, 0.056 mmol, 40%) as a colorless solid. ^{1}**H NMR** (400 MHz, $CDCl_3$, 298 K) δ 8.21 (s, 1H, H_6), 7.98 (t, J = 4.8 Hz, 1H, H_{11}), 7.69 – 7.59 (m, 2H, $H_{10,13}$), 7.47 – 7.29 (m, 6H, $H_{7,19\text{-}21}$), 7.23 (s, 1H, H_5), 7.15 – 7.04 (m, 4H, $H_{8,9,16}$), 6.87 (d, J = 8.1 Hz, 2H, H_{17}), 5.51 (d, J = 9.0 Hz, 1H, H_2), 5.03 (s, 2H, H_{18}), 4.80 – 4.68 (m, 2H, $H_{3,14}$), 4.51 (dd, J = 17.6, 5.6 Hz, 1H, H_{12a}), 4.20 (dd, J = 17.6, 3.9 Hz, 1H, H_{12b}), 3.70 – 3.53 (m, 35H, $H_{22,a}$), 3.38 (dd, J = 14.8, 4.2 Hz, 1H, H_{4a}), 3.13 – 2.98 (m, 2H, $H_{4b,15a}$), 2.91 (dd, J = 13.3, 6.3 Hz, 1H, H_{15b}), 1.23 (s, 9H, H_1). ^{13}**C NMR** (101 MHz, $CDCl_3$, 298 K) δ 172.61, 171.64, 169.97, 157.61, 155.12, 137.13, 136.18, 130.40, 129.32, 128.65, 128.02, 127.53, 126.15, 122.93, 121.45, 118.98, 116.06, 114.83, 112.32, 110.99, 78.42, 70.63, 70.60, 70.02, 54.55, 51.69, 43.44, 37.86, 29.40, 28.30, 27.66. **HRMS** (ESI$^+$) calculated for $C_{51}H_{72}N_4O_{15}Na$ $[M+Na]^+$ = 1003.4886, found 1003.4841.

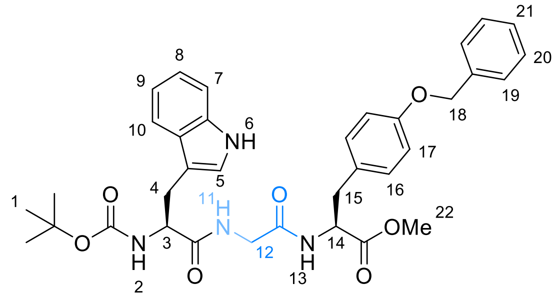

^{1}**H NMR** (400 MHz, $CDCl_3$, 298 K) δ 8.31 (s, 1H, H_6), 7.60 (d, J = 7.8 Hz, 1H, H_{10}), 7.42 – 7.27 (m, 6H, $H_{7,19\text{-}21}$), 7.17 (t, J = 7.7 Hz, H, H_8), 7.10 (t, J = 7.3 Hz, 1H, H_9) 7.00 (d, J = 8.4 Hz, 2H, H_{16}), 6.94 (s, 1H, H_5), 6.88 (d, J = 7.3 Hz, 2H, H_{17}) 6.66 (d, J = 7.8 Hz, 1H, H_{13}), 6.49 (t, J = 6.8 Hz, 1H, H_{11}), 5.17 (d, J = 7.3 Hz, 1H, H_2), 4.98 (s, 2H, H_{18}), 4.73 (q, J = 6.6 Hz, 1H, H_{14}), 4.43 (q, J = 6.9 Hz, 1H, H_3), 3.85 (dd, J = 16.8, 5.6 Hz, 1H, H^{12a}), 3.69 (m, 4H, $H_{12b,22}$), 3.09 – 2.92 (m, 2H, H_4), 3.09 – 2.89 (m, 2H, H_{15}), 1.40 (s, 9H, H_1). ^{13}**C NMR** (101 MHz, $CDCl_3$, 298 K) δ 172.52, 171.96, 168.37, 158.06, 155.67, 136.99, 136.29, 130.45, 128.69, 128.10, 127.65, 123.39, 122.37, 119.81, 118.80, 115.09, 111.45, 110.34, 80.43, 70.09, 58.11, 55.52, 53.65, 52.51, 43.24, 37.05, 29.45, 28.42, 27.34. **HRMS** (ESI$^+$) calculated for $C_{35}H_{40}N_4O_7Na$ $[M+Na]^+$ = 651.2795, found 651.2743.

Synthesis of **24C8⊂12** and **12**

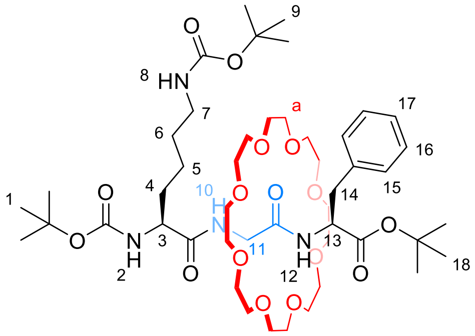

To a stirred solution of 24-crown-8 (49 mg, 0.14 mmol, 1.0 eq.) and **3d** (39 mg, 0.14 mmol, 1.0 eq., obtained from **3d**·CF_3CO_2H, using general procedure 1) in toluene (1.0 mL) was added **1g** (65 mg, 0.14 mmol, 1.0 eq.). The mixture was stirred at room temperature for 16 hours. The reaction mixture was concentrated under reduced pressure. Purification of the crude residue by prep TLC (SiO_2, EtOAc/hexane (1:1→10:1, v/v)) afforded **24C8⊂12** (52 mg, 0.054 mmol, 39%) as a colorless solid and non-interlocked tripeptide **12** (25 mg, 0.041 mmol, 29%) as a colorless solid. **^{1}H NMR** (400 MHz, $CDCl_3$, 298 K) δ 7.90 – 7.81 (m, 1H, H_{10}), 7.52 (d, J = 8.0 Hz, 1H, H_{12}), 7.28 – 7.17 (m, 5H, $H_{15\text{-}17}$), 5.50 (d, J = 8.7 Hz, 1H, H_2), 5.11 – 4.99 (m, 1H, H_8), 4.75 (dt, J = 9.5, 7.7 Hz, 1H, H_{13}), 4.49 – 4.38 (m, 2H, $H_{3,11a}$), 4.19 (dd, J = 18.0, 3.8 Hz, 1H, H_{11b}), 3.70 – 3.57 (m, 32H, H_a), 3.22 – 2.98 (m, 3H, $H_{7,14a}$), 2.88 (dd, J = 13.5, 9.4 Hz, 1H, H_{14b}), 1.89 (m, 1H, H_{4a}), 1.56 – 1.40 (m, 23H, $H_{4b,5,6,9,18}$), 1.23 (s, 9H, H_1). **^{13}C NMR** (101 MHz, $CDCl_3$, 298 K) δ 171.85, 171.11, 169.63, 156.16, 155.56, 137.10, 129.73, 128.29, 126.61, 81.08, 78.79, 78.68, 70.77, 70.60, 54.42, 53.36, 43.40, 40.19, 39.58, 33.85, 29.28, 28.60, 28.52, 27.88, 22.83. **HRMS** (ESI$^+$) calculated for $C_{47}H_{83}N_3O_{16}$ $[M+H]^+$ = 959.5799, found 959.5776.

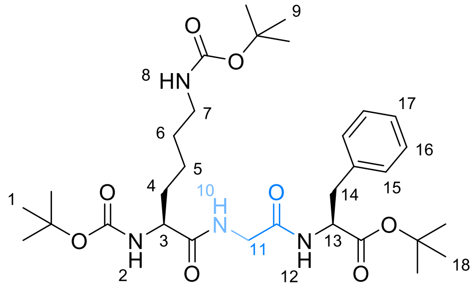

^{1}H NMR (400 MHz, $CDCl_3$, 298 K) δ 7.27 – 7.20 (m, 3H, $H_{16,17}$), 7.18 – 7.11 (m, 2H, H_{15}), 6.86 (t, J = 5.4 Hz, 1H, H_{10}), 6.61 (d, J = 7.7 Hz, 1H, H_{12}), 5.23 (d, J = 7.4 Hz, 1H, H_2), 4.76 – 4.66 (m, 2H, $H_{8,13}$), 4.14 – 4.04 (m, 1H, H_3), 4.01 – 3.93 (m, 1H, H_{11a}), 3.87 (dd, J = 16.8, 5.3 Hz, 1H, H_{11b}), 3.13 – 3.01 (m, $H_{7,14}$), 1.85 – 1.76 (m, 1H, H_{4a}), 1.69 – 1.56 (m, 1H, H_{4b}), 1.54 – 1.38 (m, 31H, $H_{1,5,6,9,18}$). **^{13}C NMR** (101 MHz, $CDCl_3$, 298 K) δ 172.74, 170.45, 168.21, 156.36, 155.99, 136.11, 129.63, 128.55, 127.13, 82.63, 80.35, 79.28, 54.71, 53.79, 43.05, 39.92, 38.15, 31.98, 29.78, 28.59, 28.47, 28.06, 22.62. **HRMS** (ESI$^+$) calculated for $C_{31}H_{51}N_4O_8$ $[M+H]^+$ = 607.3733, found 607.3707.

Synthesis of **24C8⊂13** and **13**

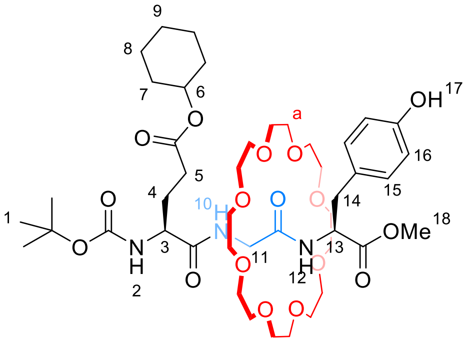

To a stirred solution of 24-crown-8 (49 mg, 0.14 mmol, 1.0 eq.), triethylamine (21 µL, 0.15 mmol, 1.1 eq.) and **3e** (35 mg, 0.14 mmol, 1.0 eq., obtained from **3e**·CF_3CO_2H, using general procedure 1) in toluene (1.0 mL) was added **1h** (63 mg, 0.14 mmol, 1.0 eq.). The mixture was stirred at room temperature for 16 hours. The reaction mixture was concentrated under reduced pressure. Purification of the crude residue by prep TLC (SiO_2, EtOAc/hexane (1:1→10:1, v/v)) afforded **24C8⊂13** (31 mg, 0.034 mmol, 24%) as a colorless solid and non-interlocked tripeptide **13** (24 mg, 0.043 mmol, 30%) as a colorless solid. **^{1}H NMR** (400 MHz, $CDCl_3$, 298 K) δ 7.89 (t, J = 4.4 Hz, 1H, H_{10}), 7.63 (d, J = 7.0 Hz, 1H, H_{12}), 7.01 (d, J = 8.4 Hz, 2H, H_{15}), 6.75 (d, J = 8.4 Hz, 2H, H_{16}), 5.52 (d, J = 8.6 Hz, 1H, H_2), 4.76 – 4.65 (m, 2H, $H_{6,13}$), 4.56 – 4.42 (m, 2H, $H_{3,11a}$), 4.14 (dd, J = 17.6, 3.5 Hz, 1H, H_{11b}), 3.68 – 3.52 (m, H_a), 3.50 (s, 3H, H_{18}), 3.03 (dd, J = 13.6, 5.1 Hz, 1H, H_{14a}), 2.86 (dd, J = 12.4, 9.1 Hz, 1H, H_{14b}), 2.45 – 2.35 (m, 2H, H_5), 2.28 – 2.15 (m, 1H, H_{4a}), 1.87 – 1.74 (m, 3H, $H_{4b,7a}$), 1.74 – 1.61 (m, 2H, H_{8a}), 1.39 (m, 13H, $H_{1,9,7b,8b}$). **^{13}C NMR** (101 MHz, $CDCl_3$, 298 K) δ 173.34, 172.67, 171.13, 170.10, 155.42, 155.13, 130.48, 128.35, 115.47, 78.88, 72.43, 70.74, 70.69, 54.63, 52.95, 51.74, 43.42, 37.86, 31.83, 31.80, 31.37, 29.57, 28.50, 28.42, 25.51, 23.90. **HRMS** (ESI$^+$) calculated for $C_{44}H_{74}N_3O_{17}$ $[M+H]^+$ = 916.5013, found 916.5085.

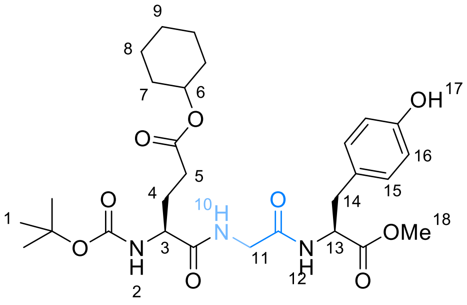

^{1}H NMR (400 MHz, $CDCl_3$, 298 K) δ 7.38 (s, 1H, H_{17}), 7.20 (t, J = 5.7 Hz, 1H, H_{10}), 7.00 (d, J = 8.1 Hz, 1H, H_{12}), 6.91 (d, J = 8.2 Hz, 2H, H_{15}), 6.71 (d, J = 8.2 Hz, 2H, H_{16}), 5.56 (d, J = 7.9 Hz, 1H, H_2), 4.84 – 4.69 (m, 2H, $H_{6,13}$), 4.20 – 4.09 (m, 1H, H_3), 3.91 – 3.78 (m, H_{11}), 3.72 (s, 3H, H_{18}), 3.05 (dd, J = 14.0, 5.3 Hz, 1H, H_{14a}), 2.95 (dd, J = 14.0, 6.7 Hz, 1H, H_{14b}), 2.48 – 2.31 (m, 2H, H_5), 2.13 – 2.02 (m, 1H, H_{4a}), 1.92 – 1.78 (m, 3H, $H_{4b,7a}$), 1.75 – 1.65 (m, 2H, H_{8a}), 1.58 – 1.48 (m, 1H, H_{9a}), 1.42 (m, 14H, $H_{1,7b,8b,9b}$). **^{13}C NMR** (101 MHz, $CDCl_3$, 298 K) δ 173.23, 172.48, 172.09, 168.78, 156.07, 155.71, 130.48, 127.01, 115.85, 80.56, 73.53, 54.08, 53.47, 52.59, 43.03, 37.01, 31.69, 31.66, 31.00, 28.44, 27.76, 25.42, 23.85. **HRMS** (ESI$^+$) calculated for $C_{28}H_{42}N_3O_9$ $[M+H]^+$ = 564.2921, found 564.2885.

Synthesis of **24C8⊂14** and **14**

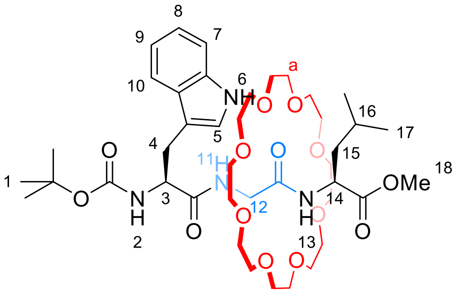

To a stirred solution of 24-crown-8 (49 mg, 0.14 mmol, 1.0 eq.) and **3b** (28 mg, 0.14 mmol, 1.0 eq., obtained from **3b**·CF_3CO_2H, using general procedure 1) in toluene (1.0 mL) was added **1f** (60 mg, 0.14 mmol, 1.0 eq.). The mixture was stirred at room temperature for 16 hours. The reaction mixture was concentrated under reduced pressure. Purification of the crude residue by prep TLC (SiO_2, EtOAc/methanol (50:1→20:1, v/v)) afforded **24C8⊂14** (36 mg, 0.043 mmol, 31%) as a colorless solid and non-interlocked tripeptide **14** (29 mg, 0.060 mmol, 43%) as a colorless solid. **^{1}H NMR** (400 MHz, $CDCl_3$, 298 K) δ 8.58 (s, 1H, H_6), 7.99 (t, J = 4.5 Hz, 1H, H_{11}), 7.64 (d, J = 7.7 Hz, 1H, H_{10}), 7.41 (d, J = 7.1 Hz, 1H, H_{13}), 7.30 (d, J = 8.1 Hz, 1H, H_7), 7.20 (d, J = 2.3 Hz, 1H, H_5), 7.12 – 6.98 (m, 2H, $H_{8,9}$), 5.51 (d, J = 9.1 Hz, 1H, H_2), 4.75 (td, J = 8.9, 4.2 Hz, 1H, H_3), 4.61 – 4.40 (m, 2H, $H_{12a,14}$), 4.14 (dd, J = 17.5, 3.9 Hz, 1H, H_{12b}), 3.67 – 3.52 (m, 35H, $H_{18,a}$), 3.36 (dd, J = 14.8, 4.2 Hz, 1H, H_{4a}), 3.01 (dd, J = 14.8, 8.8 Hz, 1H, H_{4b}), 1.75 (septet, J = 6.6 Hz, 1H, H_{16}), 1.53 (t, J = 7.2 Hz, 2H, H_{15}), 1.20 (s, 9H, H_1), 0.94 – 0.83 (m, 6H, H_{17}). **^{13}C NMR** (101 MHz, $CDCl_3$, 298 K) δ 173.84, 171.58, 169.87, 155.07, 136.19, 128.57, 122.92, 121.31, 119.16, 118.84, 112.30, 111.01, 78.30, 70.62, 70.55, 54.49, 51.82, 50.75, 43.37, 41.63, 28.25, 24.11, 22.82, 22.40. **HRMS** (ESI$^+$) calculated for $C_{41}H_{69}N_4O_{14}$ $[M+H]^+$ = 841.4805, found 841.4803.

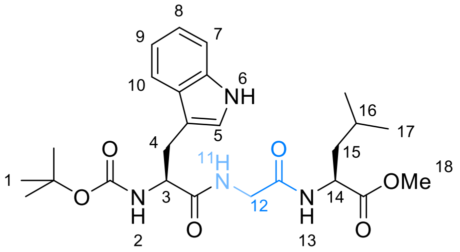

^{1}H NMR (400 MHz, $CDCl_3$, 298 K) δ 8.70 (s, 1H, H_6), 7.55 (d, J = 7.9 Hz, 1H, H_{10}), 7.33 (d, J = 8.0 Hz, 1H, H_7), 7.20 – 6.98 (m, 4H, $H_{5,8,9,13}$), 6.84 (t, J = 5.4 Hz, 1H, H_{11}), 5.28 (d, J = 7.0 Hz, 1H, H_2), 4.53 (td, J = 8.4, 5.0 Hz, 1H, H_{14}), 4.39 (q, J = 6.7 Hz, 1H, H_3), 3.91 (dd, J = 16.7, 5.8 Hz, 1H, H_{12a}), 3.68 (m, 4H, $H_{12b,18}$), 3.22 (d, J = 6.4 Hz, 2H, H_4), 1.69 – 1.51 (m, 3H, $H_{15,16}$), 1.39 (s, 9H, H_1), 0.95 – 0.83 (m, 6H, H_{17}). **^{13}C NMR** (101 MHz, $CDCl_3$, 298 K) δ 173.47, 172.63, 168.91, 155.77, 136.28, 127.51, 123.43, 122.16, 119.59, 118.64, 111.49, 110.12, 80.33, 77.48, 77.16, 76.84, 55.67, 52.42, 50.99, 43.14, 40.98, 28.38, 24.84, 22.82, 21.93. **HRMS** (ESI$^+$) calculated for $C_{25}H_{37}N_4O_6$ $[M+H]^+$ = 489.2713, found 489.2744.

Synthesis of **24C8⊂15** and **15**

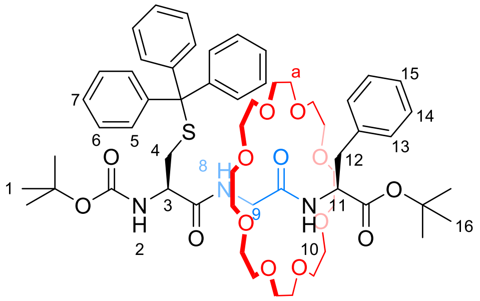

To a stirred solution of 24-crown-8 (49 mg, 0.14 mmol, 1.0 eq.) and **3d** (39 mg, 0.14 mmol, 1.0 eq., obtained from **3d**·CF_3CO_2H, using general procedure 1) in toluene (1.0 mL) was added **1g** (80 mg, 0.14 mmol, 1.0 eq.). The mixture was stirred at room temperature for 16 hours. The reaction mixture was concentrated under reduced pressure. Purification of the crude residue by prep TLC (SiO_2, EtOAc/hexane (1:1→10:1, v/v)) afforded **24C8⊂15** (38 mg, 0.035 mmol, 25%) as a colorless solid and non-interlocked tripeptide **15** (28 mg, 0.039 mmol, 28%) as a colorless solid. **^{1}H NMR** (600 MHz, $CDCl_3$, 298 K) δ 7.91 –7.81 (m, 1H, H_8), 7.50 – 7.41 (m, 7H, $H_{5,10}$), 7.23 – 7.14 (m, 14H, $H_{6,7,13\text{-}15}$), 5.41 (d, J = 9.3 Hz, 1H, H_2), 4.73 – 4.68 (m, 1H, H_{11}), 4.67 – 4.51 (m, 1H, H_3), 4.32 (dd, J = 17.6, 4.6 Hz, 1H, H_{9a}), 4.12 (dd, J = 17.7, 4.1 Hz, 1H, H_{9b}), 3.58 – 3.50 (m, 32H, H_a), 3.06 (m, 1H, H_{12a}), 2.83 (m, 1H, H_{12b}), 2.72 (m, 1H, H_{4a}), 2.38 (m, 1H, H_{4b}), 1.44 (s, 9H, H_1), 1.20 (s, 9H, H_{16}) **^{13}C NMR** (151 MHz, $CDCl_3$, 298 K) δ 171.15, 169.81, 169.46, 155.06, 145.33, 137.27, 129.93, 129.78, 129.72, 128.28, 127.83, 126.58, 126.47, 81.02, 78.71, 70.72, 70.63, 66.15, 54.42, 52.03, 45.82, 43.45, 39.65, 36.46, 29.85, 28.63, 27.92. **HRMS** (ESI$^+$) calculated for $C_{58}H_{82}N_3O_{14}S$ $[M+H]^+$ = 1076.5512, found 1076.5521.

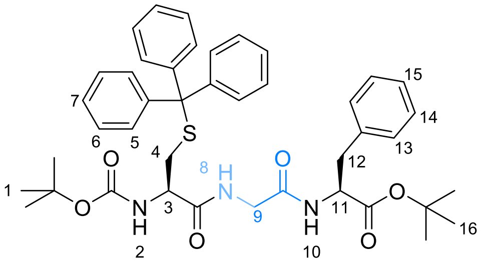

^{1}H NMR (600 MHz, $CDCl_3$, 298 K) δ 7.43 – 7.42 (m, 6H, H_5), 7.30 – 7.20 (m, 12H, $H_{6,7,14,15}$), 7.12 – 7.10 (m, 2H, H_{13}), 6.54 (m, 1H, H_8), 6.40 (m, 1H, H_{10}), 4.75 (m, 1H, H_2), 4.69 (dt, J = 7.8, 6.3 Hz, 1H, H_{11}), 3.91 (dd, J = 16.9, 5.8 Hz, 1H, H_{9a}), 3.81 – 3.74 (m, 2H, $H_{3,9b}$), 3.06 – 2.97 (m, 2H, H_{12}), 2.78 – 2.70 (m, 1H, H_{4a}), 2.58 (dd, J = 13.1, 5.2 Hz, 1H, H_{4b}), 1.42 (s, 9H, $H_{1/16}$), 1.38 (s, 9H, $H_{1/16}$). **^{13}C NMR** (151 MHz, $CDCl_3$, 298 K) δ 170.95, 170.34, 144.47, 129.71, 129.60, 128.56, 128.25, 127.13, 127.10, 67.47, 53.80, 43.16, 38.15, 29.85, 28.43, 28.07. **HRMS** (ESI$^+$) calculated for $C_{42}H_{49}N_3O_6SNa$ $[M+Na]^+$ = 746.3234, found 746.3236.

3.4. Synthesis of heptapeptide based-rotaxane positional isomers

3.4.1. Synthesis of *0:0:1*-24C8⊂16

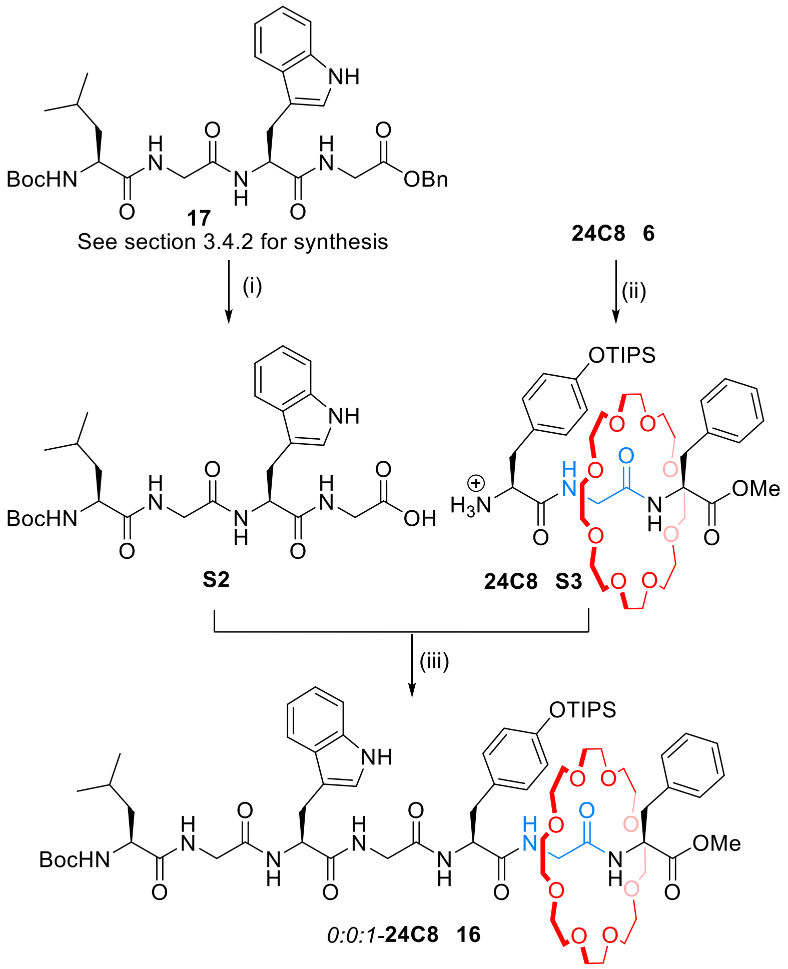

Scheme S4. Reagents and conditions: (i) Pd/C, H_2, MeOH, rt, 8 h, quant. (ii) CF_3CO_2H, CH_2Cl_2, rt, 1 h. (iii) HOBt·H_2O, EDC·HCl, *i*-Pr_2NEt, CH_2Cl_2, 0 °C – rt, 12 h, 80% over two steps.

Synthesis of **S2**

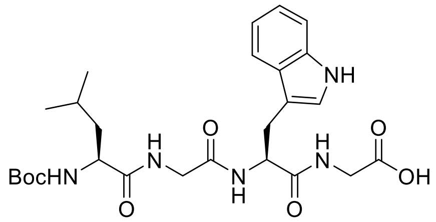

To a sealed solution of **17** (62 mg, 0.10 mmol, 1.0 eq.) in MeOH (5 mL) was added 10% Pd/C (10 mg, 0.1 eq.), the vessel was then evacuated and backfilled with 1 atm H_2 (3 ×) and stirred under a balloon of H_2 for 8 hours at room temperature. The reaction mixture was filtered over celite. The residue was concentrated under reduced pressure to give crude **S2** (assumed quant., 0.10 mmol), which was used without further purification.

Synthesis of *0:0:1*-**24C8⊂16**

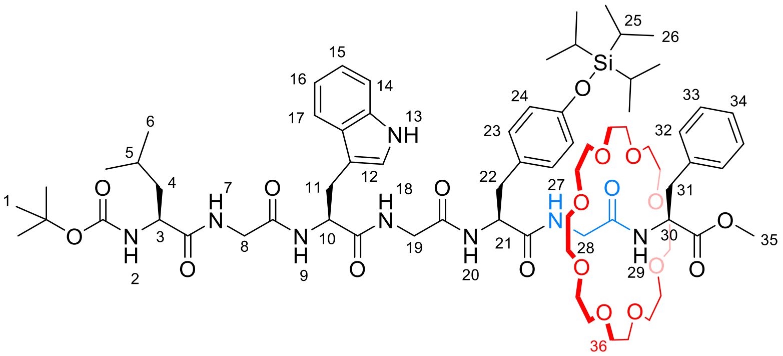

To a solution of **24C8⊂6** (100 mg, 0.1 mmol, 1.0 equiv.) in dichloromethane (2 mL) was added CF_3CO_2H (0.5 mL). The solution was stirred at room temperature for 1 hour before toluene (2 mL) was added and the mixture was concentrated to dryness under reduced pressure to give the amine salt **24C8⊂S3**, which was used without further purification (assumed quant., 0.1 mmol).

To the crude **24C8⊂S3** (assumed 0.1 mmol) in dichloromethane (5 mL), **S2** (0.1 mmol), HOBt·H_2O (18 mg, 0.12 mmol, 1.2 equiv.) and EDC·HCl (29 mg, 0.15 mmol, 1.5 equiv.) were added and stirred for 30 minutes at 0 °C. *N*,*N*-Diisopropylethylamine (35 µL, 0.2 mmol, 2.0 equiv.) was added to the reaction mixture and stirred for 12 hours at room temperature. The reaction mixture was concentrated under reduced pressure and H_2O (5 mL) was added. The resulting solution was extracted with dichloromethane (3 × 5 mL). The combined organic extracts were washed with brine (5 mL), dried with $MgSO_4$ and concentrated under reduced pressure. Flash column chromatography of the crude residue (SiO_2, MeOH/EtOAc 1:20) afforded *0:0:1*-**24C8⊂16** (114 mg, 0.08 mmol, 80%) as a colorless oil. **^{1}H NMR** (500 MHz, DMSO-*d*$_6$, 298 K) δ 10.76 (s, 1H, H_{13}), 8.17 (t, *J* = 5.7 Hz, 1H, H_{18}), 8.04 (d, *J* = 8.1 Hz, 1H, H_9), 7.89 (t, *J* = 5.6 Hz, 1H, H_7), 7.76 (t, *J* = 4.8 Hz, 1H, H_{27}), 7.69 (d, *J* = 9.0 Hz, 1H, H_{20}), 7.66 (d, *J* = 6.3 Hz, 1H, H_{29}), 7.57 (d, *J* = 7.9 Hz, 1H, H_{14}), 7.30 (t, *J* = 8.5 Hz, 3H, $H_{17,32}$), 7.22 (t, *J* = 7.3 Hz, 1H, H_{34}), 7.19 – 7.10 (m, 5H, $H_{12,23,33}$), 7.04 (t, *J* = 7.5 Hz, 1H, H_{15}), 6.95 (t, *J* = 7.4 Hz, 1H, H_{16}), 6.89 (d, *J* = 8.2 Hz, 1H, H_2), 6.71 (d, *J* = 8.1 Hz, 2H, H_{24}), 4.75 – 4.68 (m, 1H, H_{21}), 4.57 – 4.50 (m, 1H, H_{10}), 4.45 (dt, *J* = 10.7, 5.7 Hz, 1H, H_{30}), 4.25 (dd, *J* = 17.7, 5.0 Hz, 1H, H_{28a}), 4.10 (dd, *J* = 17.6, 4.3 Hz, 1H, H_{28b}), 3.95 (q, *J* = 7.8 Hz, 1H, H_3), 3.77 – 3.62 (m, 3H, $H_{8,19a}$), 3.59 – 3.49 (m, 36H, $H_{19b,35,36}$), 3.20 – 3.07 (m, 3H, $H_{11a,22a,31a}$), 2.96 – 2.82 (m, 2H, H_{11b}, H_{31b}), 2.58 (dd, *J* = 13.8, 9.8 Hz, 1H, H_{22b}), 1.58 (hept, *J* = 7.4, 6.6 Hz, 1H, H_5), 1.41 – 1.32 (m, 11H, $H_{1,5}$), 1.25 – 1.12 (m, 3H, H_{25}), 1.03 (d, *J* = 7.4 Hz, 18H, H_{26}), 0.87 – 0.79 (m, 6H, H_6). **^{13}C NMR** (125 MHz, DMSO-*d*$_6$, 298 K) δ 172.73, 171.81, 171.55, 170.22, 169.44, 168.47, 167.44, 155.35, 153.57, 136.84, 136.03, 130.93, 130.46, 128.91, 128.38, 127.30, 126.62, 123.56, 120.74, 118.96, 118.33, 118.13, 111.22, 110.05, 78.01, 70.04, 69.83, 69.77, 54.49, 53.39, 53.20, 52.71, 51.40, 42.56, 42.03, 41.90, 40.77, 37.53, 37.18, 28.17, 27.87, 24.22, 23.01, 21.40, 17.73, 12.03. **HRMS** (ESI$^+$) Calculated for $C_{72}H_{113}O_{19}N_8Si$ $[M+H]^+$ = 1421.7886, found 1421.7849.

3.4.2 Synthesis of *1:0:0*-24C8⊂16

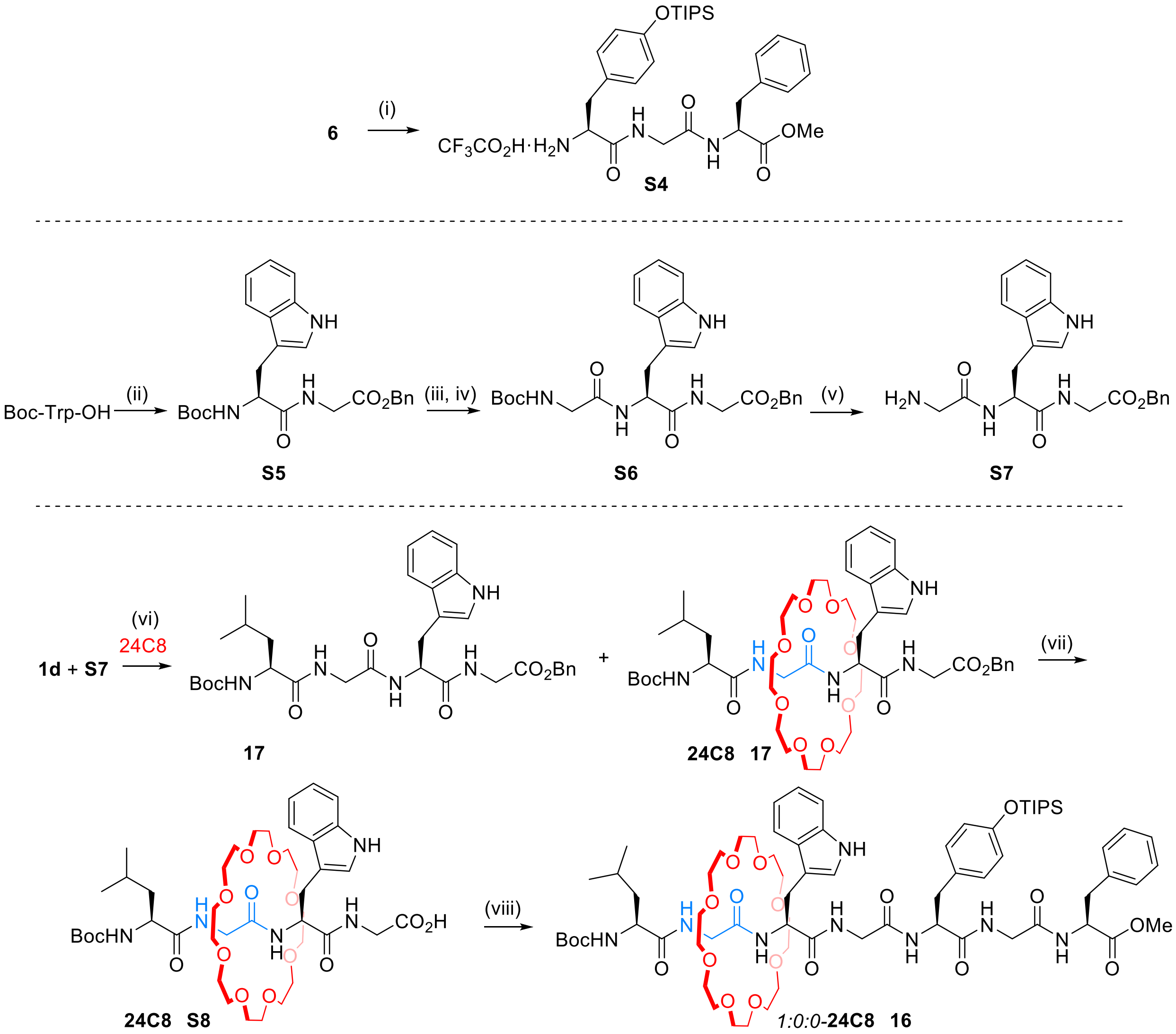

Scheme S5. Reagents and conditions: (i) CF_3CO_2H, CH_2Cl_2, rt, 1 h, assumed quant. (ii) Glycine benzyl ester hydrochloride, HOBt·H_2O, EDC·HCl, *i*-Pr_2NEt, CH_2Cl_2, 0 °C – rt, 12 h, 83%. (iii) CF_3CO_2H, CH_2Cl_2, rt, 1 h. (iv) Boc-Gly-OH, HOBt·H_2O, EDC·HCl, *i*-Pr_2NEt, CH_2Cl_2, 0 °C – rt, 12 h, 87% over two steps. (v) CF_3CO_2H, CH_2Cl_2, rt, 1 h, assumed quant. (vi) 24-Crown-8, Et_3N, toluene, rt, 36 h, **24C8⊂17**, 35%, **17**, 44%. (vii) Pd/C, H_2, MeOH, rt, 8 h. (viii) **S4**, EDC·HCl, HOBt·H_2O, *i*-Pr_2NEt, CH_2Cl_2, 0 °C – rt, 12 h, 74% over two steps.

Synthesis of **S4**

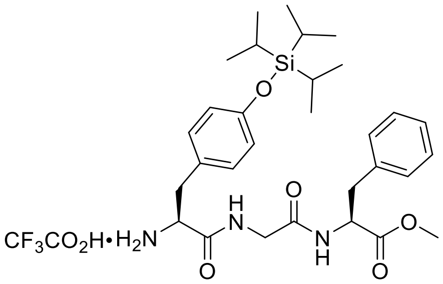

To a solution of **6** (65 mg, 0.1 mmol, 1.0 equiv., see section 3.3 for synthesis and characterization) in dichloromethane (2 mL) was added CF_3CO_2H (0.5 mL). The solution was stirred at room temperature for 1 hour before toluene (2 mL) was added. The mixture was concentrated to dryness under reduced pressure. The crude residue was used immediately without further purification (assumed quant., 0.1 mmol).

Synthesis of **S5**

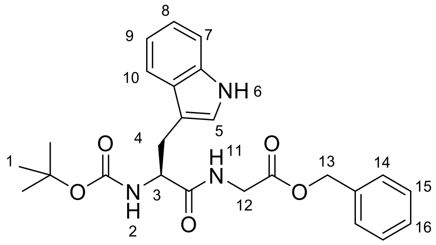

To a solution of Boc-Trp-OH (3.04 g, 10 mmol, 1.0 equiv.) in dichloromethane (40 mL) were added H-Gly-OBn.HCl (2.01 g, 10 mmol, 1.0 equiv.), HOBt·H_2O (1.84 g, 12 mmol, 1.2 equiv.) and EDC·HCl (2.88 g, 15 mmol, 1.5 equiv.). The reaction mixture was stirred for 0.5 hours at 0 °C. *N*,*N*-Diisopropylethylamine (3.5 mL, 20 mmol, 2 equiv.) was added to the reaction mixture and stirred for 12 hours at room temperature. The reaction mixture was concentrated under reduced pressure and H_2O (100 mL) was added. The resulting solution was extracted with dichloromethane (3 × 100 mL). The combined organic extracts were washed with brine (100 mL), dried with $MgSO_4$ and concentrated under reduced pressure. Flash column chromatography of the crude residue (SiO_2, EtOAc/hexane 1:1) afforded **S5** (3.74 g, 8.3 mmol, 83%) as a white foam. **^{1}H NMR** (500 MHz, $CDCl_3$, 298 K) δ 8.46 (s, 1H, H_6), 7.61 (d, *J* = 7.9 Hz, 1H, H_7), 7.40 – 7.27 (m, 6H, $H_{9,14,15,16}$), 7.17 (t, *J* = 7.5 Hz, 1H, H_{10}), 7.09 (t, *J* = 7.5 Hz, 1H, H_8), 7.02 (s, 1H, H_5), 6.56 (t, *J* = 5.4 Hz, 1H, H_{11}), 5.33 – 5.21 (m, 1H, H_2), 5.09 (s, 2H, H_{13}), 4.71 – 4.44 (m, 1H, H_3), 4.09 – 3.77 (m, 2H, H_{12}), 3.37 – 3.15 (m, 2H, H_4), 1.42 (s, 9H, H_1). **^{13}C NMR** (125 MHz, $CDCl_3$, 298 K) δ 172.27, 169.43, 155.66, 136.25, 135.20, 128.70, 128.65, 128.63, 128.59, 128.41, 127.60, 123.47, 122.13, 119.60, 118.76, 111.36, 110.24, 80.23, 77.37, 67.16, 55.15, 41.39, 28.34. **HRMS** (ESI$^+$) Calculated for $C_{25}H_{29}O_5N_3Na$ $[M+Na]^+$ = 474.1999, found 474.1993.

Synthesis of **S6**

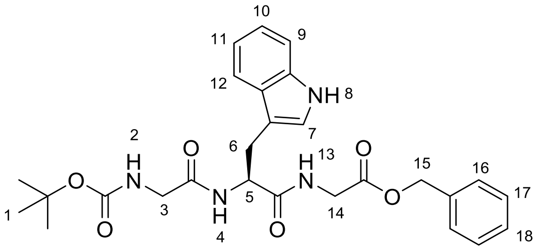

To a solution of **S5** (900 mg, 2.0 mmol, 1.0 equiv.) in dichloromethane (20 mL) was added CF_3CO_2H (5 mL). The solution was stirred at room temperature for 1 hour before toluene (10 mL) was added and the mixture was concentrated to dryness under reduced pressure to give the crude amine (assumed quant., 2.0 mmol). The crude salt was dissolved in dichloromethane (20 mL), Boc-Gly-OH (350 mg, 2.0 mmol, 1.0 equiv.), HOBt·H_2O (367 mg, 2.4 mmol, 1.2 equiv.) and EDC·HCl (576 mg, 3.0 mmol, 1.5 equiv.) were added and the reaction mixture was stirred for 30 minutes at 0 °C. *N*,*N*-Diisopropylethylamine (0.71 mL, 4.0 mmol, 2 equiv.) was added to the reaction mixture and stirred for 12 hours at room temperature. The reaction mixture was concentrated under reduced pressure and H_2O (20 mL) was added. The resulting solution was extracted with dichloromethane (3 × 20 mL). The combined organic extracts were washed with brine (20 mL), dried with $MgSO_4$ and concentrated under reduced pressure. Flash column chromatography of the crude residue (SiO_2, acetone/hexane 1:3) afforded **S6** (883 mg, 1.74 mmol, 87%) as a colorless solid. **^{1}H NMR** (500 MHz, $CDCl_3$, 298 K) δ 9.01 – 8.30 (m, 1H, H_8), 7.58 – 7.53 (m, 1H, H_9), 7.38 – 7.27 (m, 6H, $H_{11,16,17,18}$), 7.21 – 7.10 (m, 2H, $H_{12,13}$), 7.10 – 7.02 (m, 2H, $H_{4,10}$), 6.99 – 6.95 (m, 1H, H_7), 5.42 – 5.24 (m, 1H, H_2), 5.14 – 5.01 (m, 2H, H_{15}), 4.86 – 4.76 (m, 1H, H_5), 3.97 – 3.81 (m, 2H, H_{14}), 3.61 (d, J = 5.8 Hz, 2H, H_3), 3.35 – 3.10 (m, 2H, H_6), 1.38 (s, 9H, H_1). **^{13}C NMR** (125 MHz, $CDCl_3$, 298 K) δ 172.00, 169.86, 169.63, 156.38, 136.18, 135.25, 128.69, 128.56, 128.40, 127.56, 123.72, 121.99, 119.49, 118.52, 111.48, 109.87, 80.31, 77.78, 67.16, 53.74, 44.19, 41.42, 28.31. **HRMS** (ESI$^+$) Calculated for $C_{27}H_{32}O_6N_4Na$ $[M+Na]^+$ = 531.2214, found 531.2209.

Synthesis of **S7**

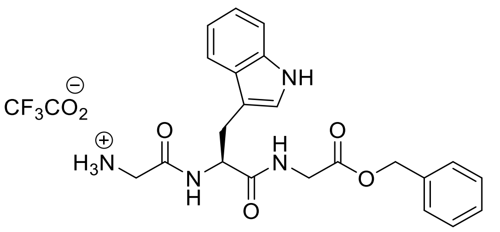

To a solution of **S6** (203 mg, 0.4 mmol, 1.0 equiv.) in dichloromethane (4 mL) was added CF_3CO_2H (1 mL). The solution was stirred at room temperature for 1 hour before toluene (10 mL) was added and the mixture was concentrated to dryness under reduced pressure to give the crude amine (assumed quant., 0.4 mmol).

Synthesis of **24C8⊂17** and **17**

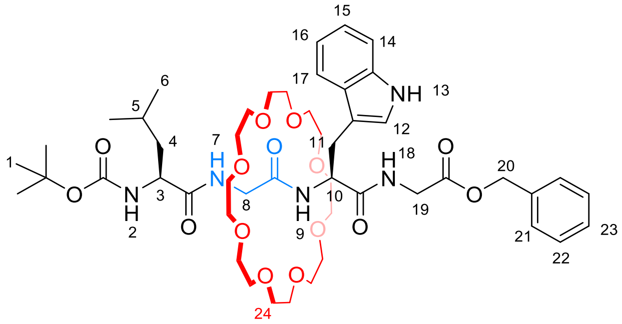

S7 (0.4 mmol) was dissolved in toluene (3 mL), Et_3N (0.13 mL, 1 mmol, 2.5 equiv.) was added followed by activated ester **1d** (140 mg, 0.4 mmol, 1.0 equiv.) and 24-crown-8 (140 mg, 0.4 mmol, 1.0 equiv.). The reaction mixture was stirred for 36 hours at room temperature. The solvent was removed under reduced pressure. Flash column chromatography of the crude residue (SiO_2, MeOH/EtOAc 1:20) afforded **24C8⊂17** (136 mg, 0.140 mmol, 35%) as a colorless oil and **17** (109 mg, 0.176 mmol, 44%) as a colorless oil. **^{1}H NMR** (500 MHz, $CDCl_3$, 298 K) δ 8.24 – 8.12 (m, 1H, H_{13}), 7.90 – 7.84 (m, 1H, H_7), 7.81 (t, *J* = 6.1 Hz, 1H, H_{18}), 7.58 (d, *J* = 7.8 Hz, 1H, H_{14}), 7.54 (d, *J* = 8.0 Hz, 1H, H_9), 7.36 – 7.31 (m, 5H, $H_{21,22,23}$), 7.29 (d, *J* = 8.1 Hz, 1H, H_{17}), 7.17 (s, 1H, H_{12}), 7.12 (t, *J* = 7.5 Hz, 1H, H_{16}), 7.06 (t, *J* = 7.4 Hz, 1H, H_{15}), 5.37 (d, *J* = 9.4 Hz, 1H, H_2), 5.22 – 5.05 (m, 2H, H_{20}), 4.94 – 4.85 (m, 1H, H_{10}), 4.49 – 4.33 (m, 2H, $H_{3,8a}$), 4.21 (dd, *J* = 17.3, 6.5 Hz, 1H, H_{19a}), 4.01 (d, *J* = 17.7, 1H, H_{8b}), 3.75 (dd, *J* = 17.3, 5.3 Hz, 1H, H_{19b}), 3.53 – 3.38 (m, 33H, $H_{11a,24}$), 3.18 (dd, *J* = 15.6, 8.9 Hz, 1H, H_{11b}), 1.73 – 1.62 (m, 1H, H_{4a}), 1.61 – 1.51 (m, 1H, H_{4b}), 1.48 – 1.34 (m, 10H, $H_{1,5}$), 0.97 (d, *J* = 6.4 Hz, 3H, H_{6a}), 0.89 (d, *J* = 6.6 Hz, 3H, H_{6b}). **^{13}C NMR** (125 MHz, $CDCl_3$, 298 K) δ 173.21, 172.62, 170.12, 170.01, 155.33, 136.13, 135.72, 128.68, 128.50, 128.48, 128.02, 123.28, 121.75, 119.16, 118.86, 111.83, 111.81, 110.95, 78.59, 70.92, 70.58, 70.40, 66.89, 53.40, 52.49, 43.19, 43.13, 41.42, 28.51, 26.91, 24.81, 23.92, 21.99. **HRMS** (ESI^+) Calculated for $C_{49}H_{76}O_{15}N_5$ $[M+H]^+$ = 974.5332, found 974.5302.

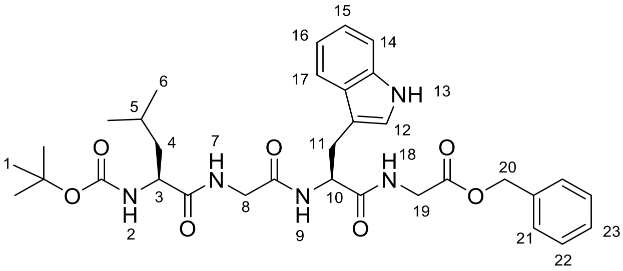

^{1}H NMR (400 MHz, $CDCl_3$, 298 K) δ 8.71 (s, 1H, H_{13}), 7.49 (d, *J* = 7.9 Hz, 1H, H_{14}), 7.36 – 7.28 (m, 7H, $H_{9,18,21,22,23}$), 7.27 – 7.21 (m, 2H, $H_{7,17}$), 7.12 – 7.06 (m, 1H, H_{16}), 7.04 – 6.96 (m, 2H, $H_{12,15}$), 5.39 (d, *J* = 8.0 Hz, 1H, H_2), 5.12 – 5.05 (m, 2H, H_{20}), 4.84 (q, *J* = 7.0 Hz, 1H, H_{10}), 4.23 – 4.12 (m, 1H, H_7), 3.95 (d, *J* = 5.5 Hz, 2H, H_{19}), 3.80 – 3.61 (m, 2H, H_8), 3.26 – 3.11 (m, 2H, H_{11}), 1.70 – 1.60 (m, 1H, H_5), 1.58 – 1.50 (m, 1H, H_{4a}), 1.48 – 1.36 (m, 10H, $H_{1,4b}$), 0.88 (d, *J* = 6.4 Hz, 6H, H_6). **^{13}C NMR** (100 MHz, $CDCl_3$, 298 K) δ 173.84, 172.14, 169.89, 169.24, 156.06, 136.23, 135.28, 128.73, 128.60, 128.51, 128.43, 127.56, 123.71, 121.96, 119.41, 118.59, 111.46, 110.02, 80.29, 77.36, 67.24, 53.77, 53.34, 43.07, 41.56, 41.48, 28.44, 28.08, 24.84, 23.17, 21.84. **HRMS** (ESI^+) Calculated for $C_{33}H_{44}O_7N_5$ $[M+H]^+$ = 622.3235, found 622.3228.

Synthesis of *1:0:0*-**24C8⊂16**

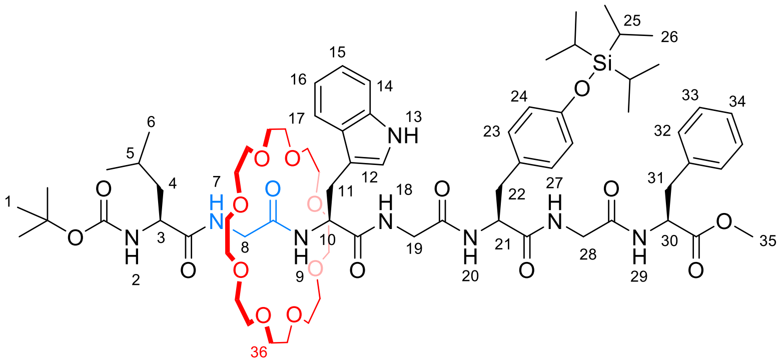

To a sealed solution of **24C8⊂17** (97 mg, 0.1 mmol, 1.0 eq.) in MeOH (5 mL) was added 10% Pd/C (10 mg, 0.1 eq.), the reaction vessel was then evacuated and backfilled three times with 1 atm H_2 (from a balloon) and stirred under a balloon of H_2 for 8 hours at room temperature. The mixture was filtered over celite and concentrated under reduced pressure to give the crude acid **24C8⊂S7** (assumed quant., 0.1 mmol), which was used without further purification.

To **24C8⊂S7** (assumed 0.1 mmol) in dichloromethane (5 mL) was added **S4** (0.1 mmol), HOBt·H_2O (18 mg, 0.12 mmol, 1.2 equiv.) and EDC·HCl (29 mg, 0.15 mmol, 1.5 equiv.) and stirred for 30 minutes at 0 °C. *N*,*N*-Diisopropylethylamine (35 µL, 0.2 mmol, 2.0 equiv.) was added to the reaction mixture and stirred for 12 hours at room temperature. The reaction mixture was concentrated under reduced pressure and H_2O (5 mL) was added. The resulting solution was extracted with dichloromethane (3 × 5 mL). The combined organic extracts were washed with brine (5 mL), dried with $MgSO_4$ and concentrated under reduced pressure. Flash column chromatography of the crude residue (SiO_2, MeOH/EtOAc 1:20) afforded *1:0:0*-**24C8⊂16** (105 mg, 0.074 mmol, 74%) as a colorless oil. **^{1}H NMR** (500 MHz, DMSO-d_6, 298 K) δ 10.79 (s, 1H, H_{13}), 8.29 (d, J = 7.6 Hz, 1H, H_{29}), 8.20 (t, J = 5.9 Hz, 1H, H_{27}), 8.07 (d, J = 8.3 Hz, 1H, H_{20}), 7.75 (t, J = 6.4 Hz, 1H, H_{18}), 7.62 (d, J = 6.6 Hz, 1H, H_9), 7.52 – 7.43 (m, 2H, $H_{7,17}$), 7.31 (d, J = 8.1 Hz, 1H, H_{14}), 7.27 (t, J = 7.5 Hz, 2H, H_{33}), 7.23 – 7.13 (m, 4H, $H_{12,32,34}$), 7.08 (d, J = 8.2 Hz, 2H, H_{23}), 7.04 (t, J = 7.7 Hz, 1H, H_{15}), 6.95 (t, J = 7.5 Hz, 1H, H_{16}), 6.70 (d, J = 7.9 Hz, 2H, H_{24}), 6.12 (d, J = 9.0 Hz, 1H, H_2), 4.52 – 4.40 (m, 3H, $H_{10,21,30}$), 4.31 – 4.17 (m, 2H, $H_{3,8a}$), 4.02 – 3.82 (m, 2H, $H_{19a,8b}$), 3.76 – 3.62 (m, 2H, H_{28}), 3.57 (s, 3H, H_{35}), 3.49 – 3.36 (m, 33H, $H_{19b,36}$), 3.15 (dd, J = 15.0, 6.8 Hz, 1H, H_{11a}), 3.08 – 2.85 (m, 4H, $H_{11b,22a,31}$), 2.69 (dd, J = 13.9, 9.8 Hz, 1H, H_{22b}), 1.62 – 1.53 (m, 1H, H_5), 1.53 – 1.45 (m, 1H, H_{4a}), 1.40 – 1.29 (m, 10H, $H_{1,4b}$), 1.24 – 1.13 (m, 3H, H_{25}), 1.03 (d, J = 7.5 Hz, 18H, H_{26}), 0.87 (d, J = 6.5 Hz, 6H, H_6). **^{13}C NMR** (125 MHz, DMSO-d_6, 298 K) δ 171.80, 171.76, 171.62, 171.28, 169.93, 168.67, 168.60, 154.91, 153.73, 136.98, 136.05, 130.63, 130.29, 129.07, 128.27, 127.30, 126.58, 123.33, 120.79, 119.03, 118.13, 118.01, 111.23, 109.99, 77.52, 69.67, 69.58, 54.21, 54.04, 53.64, 51.84, 42.65, 41.75, 41.55, 36.84, 28.15, 24.19, 23.67, 21.35, 17.72, 12.02. **HRMS** (ESI$^+$) Calculated for $C_{72}H_{113}O_{19}N_8Si$ $[M+H]^+$ = 1421.7886, found 1421.7848.

3.4.3. Synthesis of *0:1:0*-24C8⊂16 and 16

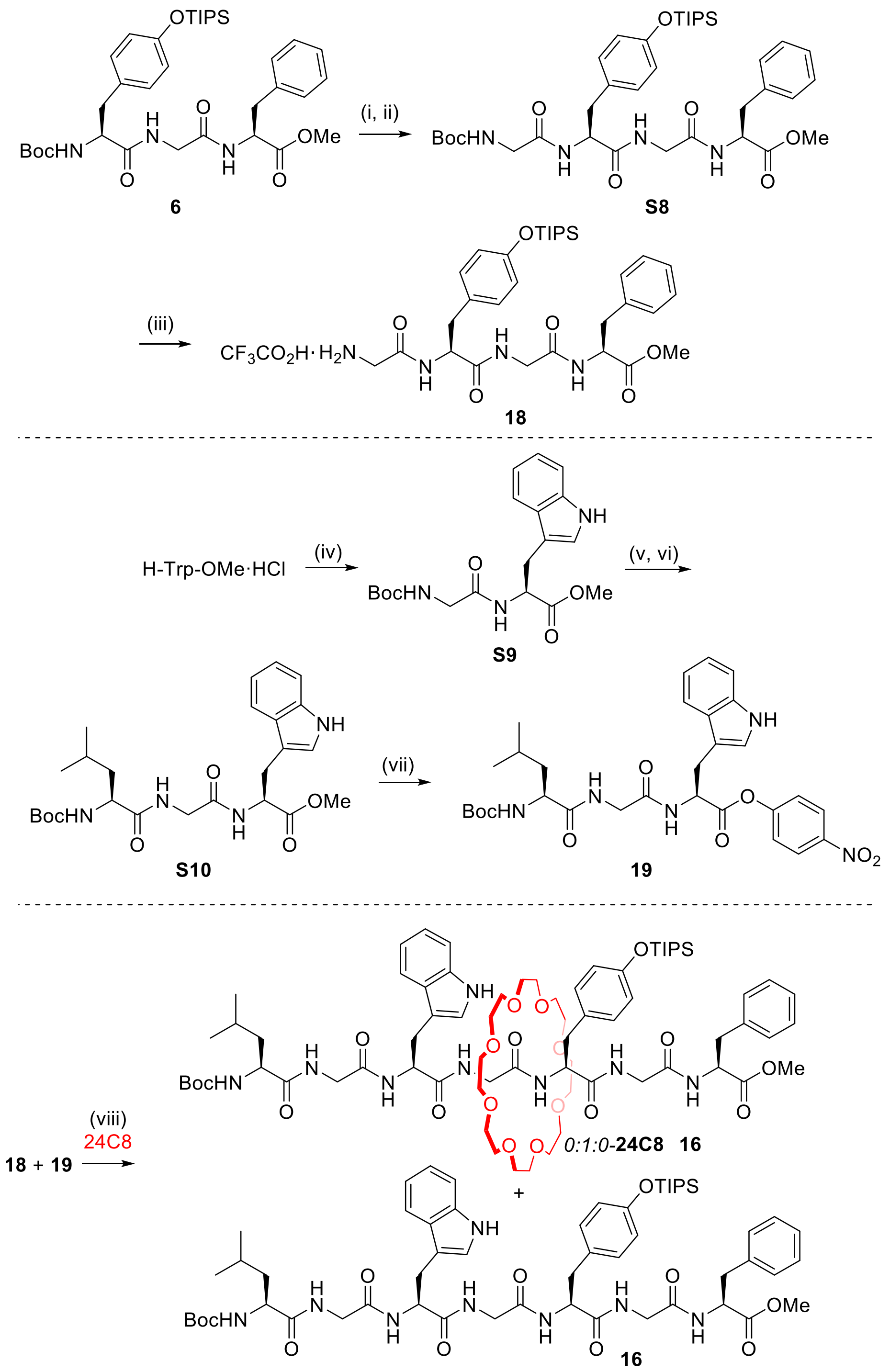

Scheme S6. Reagents and conditions: (i) CF_3CO_2H, CH_2Cl_2, rt, 2 h. (ii) Boc-Gly-OH, HOBt·H_2O, EDC·HCl, *i*-Pr_2NEt, CH_2Cl_2, 0 °C – rt, 12 h, 85% over two steps. (iii) CF_3CO_2H, CH_2Cl_2, rt, 2 h, assumed quant. (iv) Boc-Gly-OH, HOBt·H_2O, EDC·HCl, *i*-Pr_2NEt, CH_2Cl_2, 0 °C – rt, 12 h, 83%. (v) CF_3CO_2H, CH_2Cl_2, rt, 2 h. (vi) Boc-Leu-OH, HOBt·H_2O, EDC·HCl, *i*-Pr_2NEt, CH_2Cl_2, 0 °C – rt, 12 h, 74% over two steps. (vii) Me_3SnOH, 1,2-dichloroethane, 70 °C, 24 h, then 4-nitrophenyl chloroformate, DMAP, Et_3N, 0 °C, 2 h, 57%. (viii) 24-Crown-8, Et_3N, toluene, rt, 36 h, *0:1:0*-**24C8⊂16**, 11%, **16**, 70%.

Synthesis of **S8**

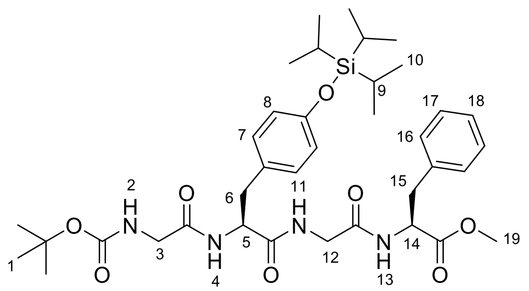

To a solution of **6** (327 mg, 0.5 mmol, 1.0 equiv., see section 3.3 for synthesis and characterization) in dichloromethane (8 mL) was added CF_3CO_2H (2 mL). The solution was stirred at room temperature for 1 hour before toluene (10 mL) was added and the mixture was concentrated to dryness under reduced pressure to give the crude amine (assumed quant., 0.5 mmol). The crude salt was dissolved in CH_2Cl_2 (10 mL), Boc-Gly-OH (88 mg, 0.5 mmol, 1.0 equiv.), HOBt·H_2O (92 mg, 0.6 mmol, 1.2 equiv.) and EDC·HCl (144 mg, 0.75 mmol, 1.5 equiv.) were added and the mixture was stirred for 30 minutes at 0 °C. *N*,*N*-Diisopropylethylamine (0.175 mL, 1.0 mmol, 2.0 equiv.) was added to the reaction mixture and stirred for 12 hours at room temperature. The liquors were concentrated under reduced pressure and H_2O (10 mL) was added. The resulting solution was extracted with dichloromethane (3 × 10 mL). The combined organic extracts were washed with brine (10 mL), dried with $MgSO_4$ and concentrated under reduced pressure. Flash column chromatography of the crude residue (SiO_2, EtOAc/hexane 1:2) afforded **S8** (303 mg, 0.43 mmol, 85%) as a colorless oil. **^{1}H NMR** (500 MHz, $CDCl_3$, 298 K) δ 7.31 (d, *J* = 8.0 Hz, 1H, H_{13}), 7.28 – 7.21 (m, 4H, $H_{4,11,17}$), 7.20 – 7.16 (m, 1H, H_{18}), 7.13 – 7.08 (m, 2H, H_{16}), 7.03 – 6.96 (m, 2H, H_7), 6.81 – 6.71 (m, 2H, H_8), 5.48 (t, *J* = 5.3 Hz, 1H, H_2), 4.82 (q, *J* = 6.9 Hz, 1H, H_{14}), 4.69 (q, *J* = 7.1 Hz, 1H, H_5), 3.97 (dd, *J* = 16.8, 5.7 Hz, 1H, H_{12a}), 3.82 – 3.69 (m, 3H, $H_{3,12b}$), 3.65 (s, 3H, H_{19}), 3.11 (dd, *J* = 13.8, 6.0 Hz, 1H, H_{15a}), 3.02 (dd, *J* = 13.9, 7.0 Hz, 1H, H_{25b}), 2.96 (dd, *J* = 13.6, 6.6 Hz, 1H, H_{6a}), 2.90 (dd, *J* = 13.9, 7.1 Hz, 1H, H_{6b}), 1.40 (s, 9H, H_1), 1.26 – 1.15 (m, 3H, H_9), 1.05 (d, *J* = 7.5 Hz, 18H, H_{10}). **^{13}C NMR** (125 MHz, $CDCl_3$, 298 K) δ 172.06, 171.51, 169.64, 168.54, 156.18, 155.09, 136.17, 130.27, 129.33, 128.78, 128.56, 127.06, 120.14, 80.02, 77.36, 54.66, 53.57, 52.39, 44.15, 43.01, 37.99, 37.63, 28.38, 17.95, 12.66. **HRMS** (ESI$^+$) Calculated for $C_{37}H_{57}O_8N_4Si$ $[M+H]^+$ = 713.3940, found 713.3916.

Synthesis of **18**

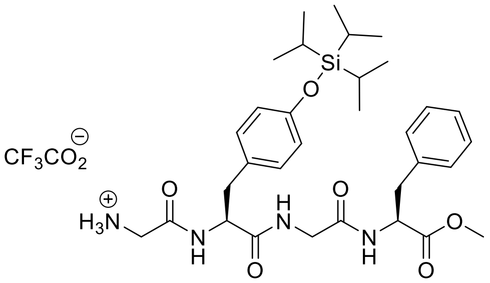

To a solution of **S8** (285 mg, 0.4 mmol, 1.0 equiv.) in CH_2Cl_2 (4 mL) was added CF_3CO_2H (1 mL). The solution was stirred at room temperature for 1 hour before toluene (10 mL) was added and the mixture was concentrated to dryness under reduced pressure. The crude amine salt **18** was used directly without further purification (assumed quant., 0.4 mmol).

Synthesis of **S9**

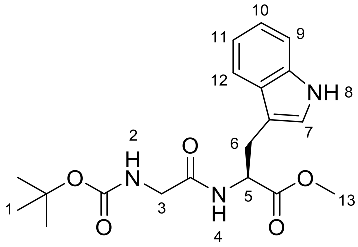

To a solution of H-Trp-OMe·HCl (2.54 g, 10 mmol, 1.0 equiv.) in dichloromethane (40 mL) were added Boc-Gly-OH (1.75 g, 10 mmol, 1.0 equiv.), HOBt·H_2O (1.84 g, 12 mmol, 1.2 equiv.) and EDC·HCl (2.88 g, 15 mmol, 1.5 equiv.). The reaction mixture was stirred for 0.5 hours at 0 °C. *N*,*N*-Diisopropylethylamine (3.5 mL, 20 mmol, 2 equiv.) was added to the reaction mixture and stirred for 12 hours at room temperature. The reaction mixture was concentrated under reduced pressure and H_2O (100 mL) was added. The resulting solution was extracted with dichloromethane (3 × 100 mL). The combined organic extracts were washed with brine (100 mL), dried with $MgSO_4$ and concentrated under reduced pressure. Flash column chromatography of the crude residue (SiO_2, EtOAc/hexane 1:1) afforded **S9** (3.1 g, 8.3 mmol, 83%) as a white foam. **^{1}H NMR** (500 MHz, $CDCl_3$) δ 8.68 – 8.50 (m, 1H, H_8), 7.49 (d, *J* = 7.9 Hz, 1H, H_{10}), 7.31 (d, *J* = 8.0 Hz, 1H, H_{12}), 7.16 (t, *J* = 7.5 Hz, 1H, H_{11}), 7.09 (t, *J* = 7.4 Hz, 1H, H_9), 6.94 (d, *J* = 2.6 Hz, 1H, H_7), 6.83 – 6.64 (m, 1H, H_4), 5.23 (s, 1H, H_2), 5.04 – 4.59 (m, 1H, H_5), 3.79 – 3.68 (m, 2H, H_3), 3.64 (s, 3H, H_{13}), 3.29 (d, *J* = 5.5 Hz, 2H, H_6), 1.42 (s, 9H, H_1). **^{13}C NMR** (125 MHz, $CDCl_3$) δ 172.31, 172.29, 169.40, 156.15, 136.23, 127.55, 123.26, 122.19, 119.64, 118.39, 111.51, 109.51, 80.27, 52.97, 52.51, 44.16, 28.36, 27.61. **HRMS** (ESI$^+$) Calculated for $C_{19}H_{25}O_5N_3Na$ $[M+Na]^+$ = 398.1686, found 398.1677.

Synthesis of **S10**

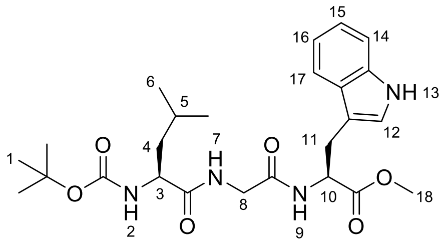

To a solution of **S9** (1.88 g, 5.0 mmol) in CH_2Cl_2 (30 mL), was added Boc-Leu-OH (1.15 g, 5.0 mmol, 1.0 equiv.), HOBt·H_2O (918 mg, 6.0 mmol, 1.2 equiv.) and EDC·HCl (1.44 g, 7.5 mmol, 1.5 equiv.) and stirred for 30 minutes at 0 °C. *N*,*N*-Diisopropylethylamine (1.75 mL, 10 mmol, 2 equiv.) was added to the reaction mixture and stirred for 12 hours at room temperature. The reaction mixture was concentrated under reduced pressure and H_2O (30 mL) was added. The resulting solution was extracted with dichloromethane (3 × 30 mL). The combined organic extracts were washed with brine (30 mL), dried with $MgSO_4$ and concentrated under reduced pressure. Flash column chromatography of the crude residue (SiO_2, acetone/hexane 1:1) afforded **S10** (1.8 g, 3.68 mmol, 74%) as a colorless solid. **^{1}H NMR** (500 MHz, $CDCl_3$, 298 K) δ 8.95 (s, 1H, H_{13}), 7.45 (d, *J* = 7.9 Hz, 1H, H_{15}), 7.28 (d, *J* = 8.1 Hz, 1H, H_{17}), 7.12 (t, *J* = 7.9 Hz, 1H, H_{16}), 7.09 – 7.02 (m, 3H, $H_{7,9,14}$), 6.96 (d, *J* = 2.5 Hz, 1H, H_{12}), 5.26 (d, *J* = 7.9 Hz, 1H, H_2), 4.81 (q, *J* = 6.2 Hz, 1H, H_{10}), 4.23 – 4.01 (m, 1H, H_3), 3.77 (dd, *J* = 16.7, 5.5 Hz, 1H, H_{8a}), 3.62 – 3.68 (m, 4H, $H_{8b,18}$), 3.29 (dd, *J* = 14.9, 5.9 Hz, 1H, H_{11a}), 3.23 (dd, *J* = 14.9, 5.3 Hz, 1H, H_{11b}), 1.70 – 1.58 (m, 1H, H_{4a}), 1.57 – 1.48 (m, 1H, H_{4b}), 1.43 (s, 10H, $H_{1,5}$), 0.89 – 0.85 (m, 6H, H_6). **^{13}C NMR** (125 MHz, $CDCl_3$, 298 K) δ 173.67, 172.28, 168.97, 156.08, 136.24, 127.38, 123.81, 122.00, 119.42, 118.35, 111.60, 109.14, 80.35, 77.36, 53.31, 52.66, 52.50, 42.96, 41.52, 28.43, 27.38, 24.80, 23.10, 21.70. **HRMS** (ESI$^+$) Calculated for $C_{25}H_{36}O_6N_4Na$ $[M+Na]^+$ = 511.2527, found 511.2517.

Synthesis of **19**

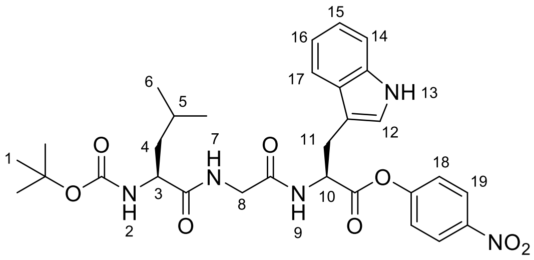

To a solution of **S10** (488 mg, 1.0 mmol, 1.0 equiv.) in 1,2-dichloroethane (10 mL) was added Me_3SnOH (900 mg, 5.0 mmol, 5.0 equiv.). The reaction mixture was stirred for 24 hours at 70 °C and cooled down to room temperature. 4-Nitrophenyl chloroformate (201 mg, 1.0 mmol, 1.0 equiv.) and Et_3N (0.13 mL, 1.0 mmol, 1.0 equiv.) were added to the reaction mixture and stirred for 30 minutes at 0 °C. DMAP (12 mg, 0.1 mmol, 0.1 equiv.) was added to the reaction mixture and stirred for additional 1.5 hours at 0 °C. The solvent was removed under reduced pressure. Flash column chromatography of the crude residue (SiO_2, EtOAc/hexane 1:5) afforded **19** (340 mg, 0.57 mmol, 57%) as a yellow foam. **^{1}H NMR** (500 MHz, CD_2Cl_2, 298 K) δ 8.66 (s, 1H, H_{13}), 8.21 – 8.11 (m, 2H, H_{19}), 7.56 (d, *J* = 8.0 Hz, 1H, H_{15}), 7.40 (d, *J* = 8.1 Hz, 1H, H_{17}), 7.22 – 7.17 (m, 2H, $H_{9,16}$), 7.15 (d, *J* = 2.4 Hz, 1H, H_{12}), 7.11 – 7.06 (m, 1H, H_{14}), 7.05 – 7.01 (m, 2H, H_{18}), 6.82 (d, *J* = 5.9 Hz, 1H, H_7), 5.04 – 4.93 (m, 2H, $H_{2,10}$), 4.16 – 4.02 (m, 1H, H_3), 3.93 (dd, *J* = 16.8, 6.1 Hz, 1H, H_{8a}), 3.84 (dd, *J* = 16.8, 5.5 Hz, 1H, H_{8b}), 3.51 – 3.37 (m, 2H, H_{11}), 1.69 – 1.54 (m, 2H, H_4), 1.48 – 1.40 (m, 10H, $H_{1,5}$), 0.93 – 0.88 (m, 6H, H_6). **^{13}C NMR** (125 MHz, CD_2Cl_2, 298 K) δ 173.87, 170.35, 170.27, 169.56, 156.61, 155.71, 146.02, 136.80, 127.83, 127.79, 125.59, 125.57, 124.27, 124.20, 122.93, 122.91, 122.81, 120.19, 118.99, 118.97, 112.06, 109.80, 80.99, 53.94, 43.65, 41.49, 32.15, 31.17, 28.62, 28.58, 27.80, 25.31, 23.30, 23.28, 23.22, 21.95, 14.44. **HRMS** (ESI^+) Calculated for $C_{30}H_{37}O_8N_5Na$ $[M+Na]^+$ = 618.2534, found 618.2523.

Synthesis of *0:1:0*-**24C8⊂16** and **16**

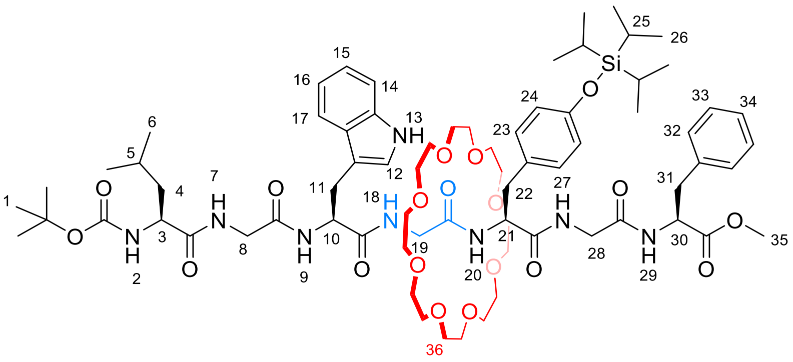

18 (0.4 mmol) was dissolved in toluene (3 mL), Et_3N (0.13 mL, 1 mmol, 2.5 equiv.) was added followed by **19** (240 mg, 0.4 mmol, 1.0 equiv.) and 24-crown-8 (140 mg, 0.4 mmol, 1.0 equiv.). The reaction mixture was stirred for 36 hours at room temperature. The solvent was removed under reduced pressure. Flash column chromatography of the crude residue (SiO_2, MeOH/EtOAc 1:20) afforded *0:1:0*-**24C8⊂16** (62 mg, 0.044 mmol, 11%) as a colorless oil and **16** (120 mg, 0.112 mmol, 28%) as a colorless solid. **^{1}H NMR** (500 MHz, DMSO-d_6, 298 K) δ 10.69 (s, 1H, H_{13}), 8.28 (d, *J* = 7.9 Hz, 1H, H_{29}), 7.83 (t, *J* = 6.0 Hz, 1H, H_7), 7.79 – 7.63 (m, 3H, $H_{9,18,27}$), 7.62 (d, *J* = 7.9 Hz, 1H, H_{17}), 7.57 (d, *J* = 3.6 Hz, 1H, H_{20}), 7.28 (d, *J* = 7.9 Hz, 1H, H_{14}), 7.26 – 7.23 (m, 2H, H_{33}), 7.23 – 7.16 (m, 3H, $H_{32,34}$), 7.10 (d, *J* = 2.4 Hz, 1H, H_{12}), 7.06 (d, *J* = 8.8 Hz, 2H, H_{23}), 7.03 – 6.99 (m, 1H, H_{15}), 6.96 – 6.86 (m, 2H, $H_{2,16}$), 6.75 – 7.63 (m, 2H, H_{24}), 4.85 – 4.76 (m, 1H, H_{10}), 4.51 – 4.43 (m, 1H, H_{30}), 4.39 (q, *J* = 6.7 Hz, 1H, H_{21}), 4.30 – 4.17 (m, 1H, H_{19a}), 4.10 – 3.96 (m, 1H, H_{19b}), 3.95 – 3.90 (m, 1H, H_3), 3.87 – 3.76 (m, 1H, H_{28a}), 3.66 (dd, *J* = 16.8, 5.5 Hz, 1H, H_{8a}), 3.61 – 3.53 (m, 4H, $H_{8b,35}$), 3.49 – 3.35 (m, 33H, $H_{28b,36}$), 3.29 – 3.19 (m, 1H, H_{11a}), 3.06 – 2.78 (m, 5H, $H_{11b,22,41}$), 1.64 – 1.53 (m, 1H, H_5), 1.45 – 1.29 (m, 11H, $H_{1,4}$), 1.27 – 1.16 (m, 3H, H_{25}), 1.07 – 0.98 (m, 18H, H_{26}), 0.86 – 0.81 (m, 6H, H_6). **^{13}C NMR** (125 MHz, DMSO-d_6, 298 K) δ 173.14, 173.05, 172.33, 172.29, 172.15, 171.69, 170.92, 170.82, 170.17, 169.74, 169.04, 168.99, 168.04, 167.96, 155.76, 154.18, 137.66, 137.55, 136.47, 136.44, 130.91, 130.81, 130.50, 130.37, 129.66, 129.56, 129.54, 129.49, 128.75, 128.69, 128.66, 128.42, 128.31, 128.29, 127.03, 126.98, 123.68, 121.03, 119.62, 119.59, 118.94, 118.48, 111.53, 111.17, 111.02, 78.44, 70.22, 70.14, 70.11, 55.12, 54.47, 54.13, 54.07, 54.03, 53.18, 53.02, 52.34, 52.30, 43.20, 42.41, 41.91, 41.25, 37.37, 37.30, 36.79, 31.71, 30.30, 28.65, 24.71, 23.48, 21.91, 21.87, 18.21, 12.51. **HRMS** (ESI$^+$) Calculated for $C_{72}H_{113}O_{19}N_8Si$ $[M+H]^+$ = 1421.7886, found 1421.7844.

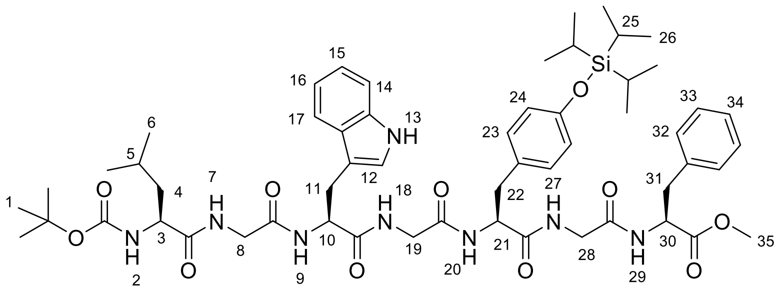

^{1}H NMR (500 MHz, DMSO-d_6, 298 K) δ 10.77 (s, 1H, H_{13}), 8.29 (d, J = 7.7 Hz, 1H, H_{29}), 8.24 (t, J = 5.8 Hz, 1H, H_{18}), 8.20 (t, J = 5.6 Hz, 1H, H_{27}), 8.05 – 7.99 (m, 2H, $H_{9,20}$), 7.91 (t, J = 5.6 Hz, 1H, H_{7}), 7.55 (d, J = 7.9 Hz, 1H, H_{17}), 7.30 (d, J = 8.1 Hz, 1H, H_{14}), 7.29 – 7.24 (m, 2H, H_{33}), 7.22 – 7.17 (m, 3H, $H_{32,34}$), 7.13 (d, J = 2.3 Hz, 1H, H_{12}), 7.09 (d, J = 8.5 Hz, 2H, H_{23}), 7.04 (t, J = 7.7 Hz, 1H, H_{15}), 6.97 – 6.92 (m, 1H, H_{16}), 6.90 (d, J = 8.2 Hz, 1H, H_{2}), 6.73 (d, J = 8.4 Hz, 2H, H_{24}), 4.56 – 4.40 (m, 3H, $H_{10,21,30}$), 4.03 – 3.89 (m, 1H, H_{3}), 3.80 – 3.68 (m, 3H, $H_{8a,19a,28a}$), 3.65 – 3.48 (m, 6H, $H_{8b,19b,28b,35}$), 3.14 (dd, J = 14.7, 4.8 Hz, 1H, H_{11a}), 3.02 (dd, J = 13.7, 5.9 Hz, 1H, H_{31a}), 2.97 – 2.88 (m, 3H, $H_{11b,22a,31b}$), 2.70 (dd, J = 13.9, 9.4 Hz, 1H, H_{22b}), 1.62 – 1.52 (m, 1H, H_{5}), 1.40 – 1.31 (m, 11H, $H_{1,4}$), 1.26 – 1.14 (m, 3H, H_{25}), 1.03 (d, J = 7.4 Hz, 18H, H_{26}), 0.85 – 0.80 (m, 6H, H_{6}). **^{13}C NMR** (125 MHz, DMSO-d_6, 298 K) δ 172.78, 171.81, 171.79, 171.57, 171.26, 168.68, 168.66, 168.51, 155.36, 153.81, 136.99, 136.02, 130.41, 130.28, 129.07, 128.27, 127.28, 126.58, 123.59, 120.77, 119.17, 118.28, 118.17, 111.24, 109.92, 78.03, 54.20, 53.63, 53.54, 52.72, 51.85, 41.92, 41.51, 40.73, 36.88, 36.84, 28.18, 27.69, 24.22, 23.00, 21.40, 17.73, 12.02. **HRMS** (ESI$^+$) Calculated for $C_{56}H_{81}O_{11}N_8Si$ $[M+H]^+$ = 1069.5789, found 1069.5756.

3.4.4. Alternative synthesis of *0:1:0*-24C8⊂16

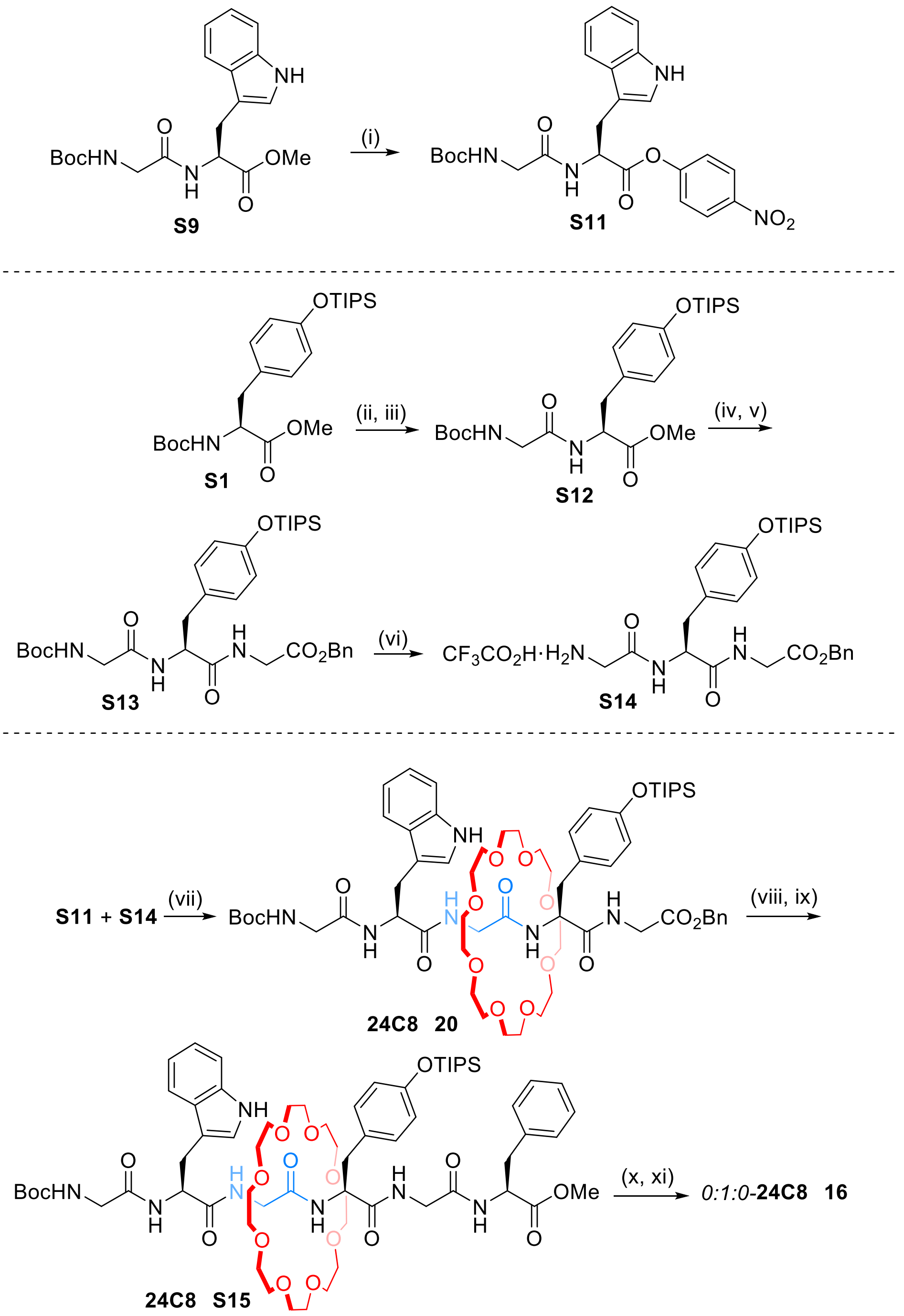

Scheme S7. Reagents and conditions: (i) Me_3SnOH, 1,2-dichloroethane, 70 °C, 24 h, then 4-nitrophenyl chloroformate, DMAP, Et_3N, 0 °C, 2 h, 51%. (ii) CF_3CO_2H, CH_2Cl_2, rt, 2 h. (iii) Boc-Gly-OH, HOBt·H_2O, EDC·HCl, *i*-Pr_2EtN, CH_2Cl_2, 0 °C – rt, 12 h, 80% over two steps. (iv) LiOH·H_2O, MeOH/THF/H_2O, rt, 3 h. (v) H-Gly-OBn, HOBt·H_2O, EDC·HCl, *i*-Pr_2EtN, CH_2Cl_2, 0 °C – rt, 12 h, 74% over two steps. (vi) CF_3CO_2H, CH_2Cl_2, rt, 2 h, assumed quant. (vii) 24-Crown-8, Et_3N, toluene, rt, 36 h, 33%. (viii) Pd/C, H_2, MeOH, rt, 8 h. (ix) H-Phe-OMe, HOBt·H_2O, EDC·HCl, *i*-Pr_2EtN, CH_2Cl_2, 0 °C – rt, 12 h, 88% over two steps. (x) CF_3CO_2H, CH_2Cl_2, rt, 2 h. (xi) Boc-Leu-OH, HOBt·H_2O, EDC·HCl, *i*-Pr_2EtN, CH_2Cl_2, 0 °C – rt, 12 h, 81% over two steps.

Synthesis of **S11**

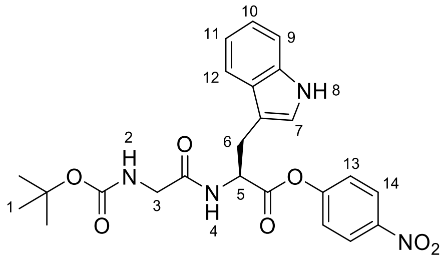

To a solution of **S9** (375 mg, 1.0 mmol, 1.0 equiv.) in 1,2-dichloroethane (10 mL) was added Me_3SnOH (900 mg, 5.0 mmol, 5 equiv.). The reaction mixture was stirred for 24 hours at 70 °C. The mixture was cooled to room temperature, 4-nitrophenyl chloroformate (201 mg, 1.0 mmol, 1.0 equiv.) and Et_3N (0.14 mL, 1.0 mmol, 1.0 equiv.) were added to the reaction mixture and stirred for 30 minutes at 0 °C. DMAP (12 mg, 0.1 mmol, 0.1 equiv.) was added to the reaction mixture and stirred for additional 1.5 hours at 0 °C. The solvent was removed under reduced pressure. Flash column chromatography of the crude residue (SiO_2, EtOAc/hexane 1:5) afforded **S11** (246 mg, 0.51 mmol, 51%) as a yellow oil. **^{1}H NMR** (500 MHz, CD_2Cl_2, 298 K) δ 8.67 (s, 1H, H_8), 8.13 (d, *J* = 9.1 Hz, 2H, H_{14}), 7.58 (d, *J* = 7.9 Hz, 1H, H_{12}), 7.41 (d, *J* = 8.1 Hz, 1H, H_9), 7.20 (t, *J* = 7.6 Hz, 1H, H_{10}), 7.14 (d, *J* = 2.4 Hz, 1H, H_7), 7.11 (t, *J* = 7.5 Hz, 1H, H_{11}), 7.01 (d, *J* = 9.1 Hz, 2H, H_{13}), 6.93 (s, 1H, H_4), 5.38 – 5.27 (m, 1H, H_2), 5.06 (q, *J* = 6.5 Hz, 1H, H_5), 3.79 (s, 2H, H_3), 3.49 – 3.38 (m, 2H, H_6), 1.42 (s, 9H, H_1). **^{13}C NMR** (125 MHz, CD_2Cl_2, 298 K) δ 170.36, 170.32, 156.69, 155.57, 145.99, 136.84, 127.82, 125.57, 123.96, 122.88, 122.86, 120.30, 118.91, 112.08, 109.71, 80.73, 44.76, 28.53, 28.01. **HRMS** (ESI$^+$) Calculated for $C_{24}H_{26}O_7N_4Na$ $[M+Na]^+$ = 505.1694, found 505.1685.

Synthesis of **S12**

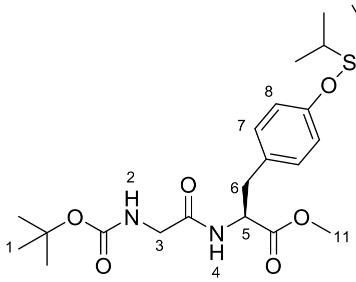

To a solution of **S1** (902 mg, 2 mmol, 1.0 equiv.) in dichloromethane (20 mL) was added CF_3CO_2H (5 mL). The solution was stirred at room temperature for 1 hour before toluene (10 mL) was added and the mixture was concentrated to dryness under reduced pressure. The crude salt was dissolved in dichloromethane (20 mL), Boc-Gly-OH (350 mg, 2 mmol, 1.0 equiv.), HOBt·H_2O (367 mg, 2.4 mmol, 1.2 equiv.) and EDC·HCl (576 mg, 3 mmol, 1.5 equiv.) was added and stirred for 30 minutes at 0 °C. *N*,*N*-Diisopropylethylamine (0.71 mL, 4 mmol, 2 equiv.) was added to the reaction mixture and stirred for another 12 hours at room temperature. The liquors were concentrated under reduced pressure and H_2O (20 mL) was added. The resulting solution was extracted with dichloromethane (3 × 20 mL). The combined organic extracts were washed with brine (20 mL), dried with $MgSO_4$ and concentrated under reduced pressure. Flash column chromatography of the crude residue (SiO_2, acetone/hexane 1:3) afforded **S12** (812 mg, 1.6 mmol, 80%) as colorless oil. **^{1}H NMR** (400 MHz, CD_2Cl_2, 298 K) δ 7.06 – 6.89 (m, 2H, H_7), 6.89 – 6.75 (m, 2H, H_8), 6.59 (d, *J* = 7.7 Hz, 1H, H_4), 5.22 (s, 1H, H_2), 4.77 (dt, *J* = 7.9, 6.0 Hz, 1H, H_5), 3.80 – 3.68 (m, 2H, H_3), 3.66 (s, 3H, H_{11}), 3.15 – 2.90 (m, 2H, H_6), 1.44 (s, 9H, H_1), 1.31 – 1.19 (m, 3H, H_9), 1.09 (d, *J* = 7.2 Hz, 18H, H_{10}). **^{13}C NMR** (101 MHz, CD_2Cl_2, 298 K) δ 172.38, 169.58, 155.80, 130.80, 129.00, 120.59, 80.51, 53.94, 53.46, 52.69, 44.82, 37.79, 28.62, 18.26, 13.24. **HRMS** (ESI$^+$) Calculated for $C_{26}H_{44}O_6N_2NaSi$ [M+Na]$^+$ = 531.2861, found 531.2845.

Synthesis of **S13**

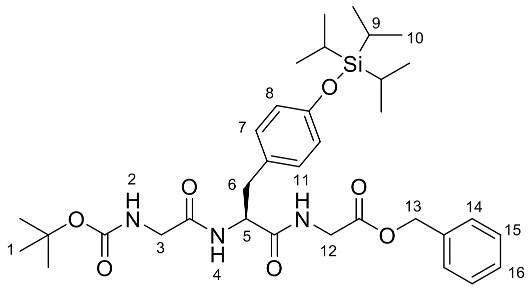

To a solution of **S12** (2.54 g, 5.0 mmol, 1.0 equiv.) in MeOH/THF/H_2O (2:2:1, 25 mL) was added LiOH·H_2O (315 mg, 7.5 mmol, 1.5 equiv.). The reaction mixture was stirred for 3 hours at room temperature. NH_4Cl (405 mg, 7.5 mmol, 1.5 equiv.) was added to quench the reaction. The solvent was removed under reduced pressure and the crude acid was used without further purification (assumed quant., 5.0 mmol). The crude acid was dissolved in dichloromethane (30 mL), to which H-Gly-OBn (1.15 g, 5 mmol, 1.0 equiv.), HOBt·H_2O (918 mg, 6 mmol, 1.2 equiv.) and EDC·HCl (1.44 g, 7.5 mmol, 1.5 equiv.) were added and stirred for 30 minutes at 0 °C. *N*,*N*-Diisopropylethylamine (1.76 mL, 10 mmol, 2 equiv.) was added to the reaction mixture and stirred for another 12 hours at room temperature. The liquors were concentrated under reduced pressure and H_2O (30 mL) was added. The resulting solution was extracted with dichloromethane (3 × 30 mL). The combined organic extracts were washed with brine (30 mL), dried with $MgSO_4$ and concentrated under reduced pressure. Flash column chromatography of the crude residue (SiO_2, acetone/hexane 1:1) afforded **S13** (2.37 g, 3.7 mmol, 74%) as white solid. **^{1}H NMR** (400 MHz, $CDCl_3$, 298 K) δ 7.38 – 7.28 (m, 5H, $H_{14,15,16}$), 7.07 – 7.00 (m, 2H, H_7), 6.92 – 6.83 (m, 2H, $H_{4,11}$), 6.82 – 6.74 (m, 2H, H_8), 5.27 (t, *J* = 5.7 Hz, 1H, H_2), 5.13 (s, 2H, H_{13}), 4.71 (dt, *J* = 8.2, 6.9 Hz, 1H, H_5), 4.05 (dd, *J* = 18.1, 5.8 Hz, 1H, H_{12a}), 3.87 (dd, *J* = 18.1, 5.2 Hz, 1H, H_{12b}), 3.80 – 3.63 (m, 2H, H_3), 3.01 (d, *J* = 6.9 Hz, 2H, H_6), 1.41 (s, 9H, H_1), 1.33 – 1.15 (m, 3H, H_9), 1.07 (d, *J* = 7.3 Hz, 18H, H_{10}). **^{13}C NMR** (100 MHz, $CDCl_3$, 298 K) δ 171.33, 169.70, 169.37, 156.24, 155.16, 135.32, 130.35, 128.80, 128.75, 128.72, 128.59, 128.45, 120.21, 80.42, 77.36, 67.20, 54.39, 44.42, 41.41, 37.39, 28.37, 18.00, 12.71. **HRMS** (ESI$^+$) Calculated for $C_{34}H_{52}O_7N_3Si$ $[M+H]^+$ = 642.3569, found 642.3552.

Synthesis of **24C8⊂20**

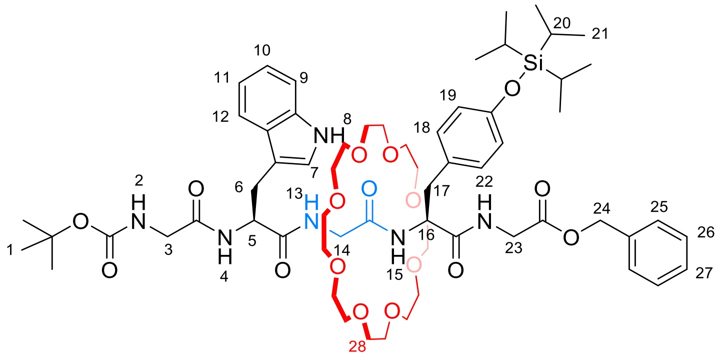

To a solution of **S13** (128 mg, 0.2 mmol, 1.0 equiv.) in dichloromethane (4 mL) was added CF_3CO_2H (1 mL). The solution was stirred at room temperature for 1 hour before toluene (5 mL) was added and the mixture was concentrated to dryness under reduced pressure. The crude amine salt **S14** (assumed 0.2 mmol) was dissolved in toluene (3 mL). Et_3N (0.07 mL, 0.5 mmol, 2.5 equiv.) was added followed by **S11** (96 mg, 0.2 mmol, 1.0 equiv.) and 24-crown-8 ether (106 mg, 0.3 mmol, 1.5 equiv.). The reaction mixture was stirred for 36 hours at room temperature. The solvent was removed under reduced pressure. Flash column chromatography of the crude residue (SiO_2, MeOH/EtOAc 1:20) afforded **24C8⊂20** (82 mg, 0.066 mmol, 33%) as a colorless oil. **^{1}H NMR** (500 MHz, DMSO-*d*$_6$, 298 K) δ 10.71 (s, 1H, H_8), 8.03 (t, *J* = 5.9 Hz, 1H, H_{22}), 7.72 (s, 1H, H_{13}), 7.60 – 7.54 (m, 1H, H_{12}), 7.53 – 7.46 (m, 2H, $H_{4,15}$), 7.40 – 7.31 (m, 5H, $H_{25,26,27}$), 7.29 (d, *J* = 8.1 Hz, 1H, H_9), 7.10 (d, *J* = 2.3 Hz, 1H, H_7), 7.07 (d, *J* = 8.2 Hz, 2H, H_{18}), 7.04 – 6.98 (m, 1H, H_{10}), 6.96 – 6.87 (m, 2H, $H_{2,11}$), 6.73 (d, *J* = 8.6 Hz, 2H, H_{19}), 5.15 – 5.08 (m, 2H, H_{24}), 4.88 – 4.76 (m, 1H, H_5), 4.57 – 4.46 (m, 1H, H_{16}), 4.30 – 4.20 (m, 1H, H_{14a}), 4.07 – 3.98 (m, 1H, H_{14b}), 3.93 (dd, *J* = 17.2, 6.2 Hz, 1H, H_{23a}), 3.72 (dt, *J* = 17.1, 5.1 Hz, 1H, H_{32b}), 3.44 (m, 34H, $H_{3,28}$), 3.28 – 3.13 (m, 1H, H_{6a}), 2.99 (dd, *J* = 14.6, 5.9 Hz, 1H, H_{17a}), 2.94 – 2.77 (m, 2H, H_{6b}, H_{27b}), 1.36 (s, 9H, H_1), 1.30 – 1.14 (m, 3H, H_{20}), 1.05 (d, *J* = 7.4 Hz, 18H, H_{21}). **^{13}C NMR** (125 MHz, DMSO-*d*$_6$, 298 K) δ 171.61, 171.46, 170.43, 169.44, 169.40, 169.38, 169.35, 169.20, 168.17, 155.63, 153.73, 135.98, 135.91, 130.35, 129.98, 129.88, 129.80, 128.39, 128.38, 128.05, 127.99, 127.96, 127.94, 127.88, 127.86, 123.20, 120.55, 119.12, 118.49, 118.04, 114.96, 111.04, 110.50, 110.43, 77.99, 69.82, 69.79, 69.72, 69.69, 65.84, 65.83, 53.89, 53.62, 52.44, 52.30, 43.38, 42.67, 42.59, 40.83, 36.58, 28.16, 17.84, 17.74, 12.08, 12.05. **HRMS** (ESI$^+$) Calculated for $C_{63}H_{97}O_{17}N_6Si$ $[M+H]^+$ = 1237.6674, found 1237.6653.

Synthesis of **24C8⊂S15**

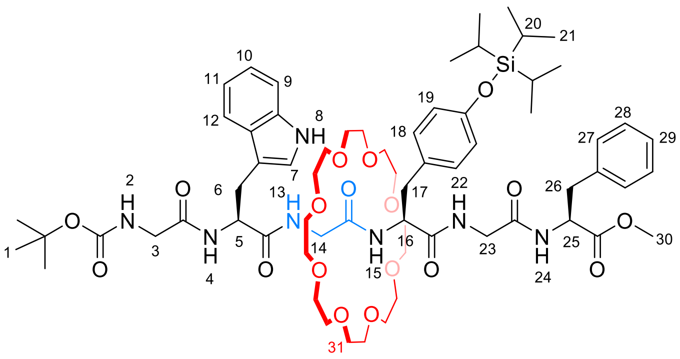

To a sealed solution of **24C8⊂20** (62 mg, 0.050 mmol, 1.0 eq.) in MeOH (5 mL) was added 10% Pd/C (6 mg, 1.0 eq.) and then evacuated and backfilled with 1 atm H_2 (3 ×) and stirred under a balloon of H_2 for 8 hours at room temperature. The mixture was filtered over celite and concentrated under reduced pressure to afford the crude acid, which was used without further purification.

The crude acid (assumed 0.050 mmol) was dissolved in dichloromethane (5 mL). H-Phe-OMe·HCl (11 mg, 0.05 mmol, 1.0 equiv.), HOBt·H_2O (9.2 mg, 0.06 mmol, 1.2 equiv.) and EDC·HCl (14.4 mg, 0.075 mmol, 1.5 equiv.) were added and the reaction mixture stirred for 30 minutes at 0 °C. *N*,*N*-Diisopropylethylamine (17.6 µL, 0.1 mmol, 2 equiv.) was added to the reaction mixture and stirred for 12 hours at room temperature. The liquors were concentrated under reduced pressure and H_2O (5 mL) was added. The resulting solution was extracted with dichloromethane (3 × 5 mL). The combined organic extracts were washed with brine (5 mL), dried with $MgSO_4$ and concentrated under reduced pressure. Flash column chromatography of the crude residue (SiO_2, acetone/hexane 1:1) afforded **24C8⊂S15** (58 mg, 0.044 mmol, 88%) as a white oil. **^{1}H NMR** (500 MHz, DMSO-*d*$_6$, 298 K) δ 10.71 (s, 1H, H_8), 8.28 (d, *J* = 7.9 Hz, 1H, H_{24}), 7.69 (t, *J* = 5.7 Hz, 1H, H_{13}), 7.61 – 7.46 (m, 4H, $H_{4,9,15,22}$), 7.30 – 7.15 (m, 6H, $H_{12,27,28,29}$), 7.11 – 6.98 (m, 4H, $H_{7,10,18}$), 6.98 – 6.92 (m, 1H, H_{11}), 6.89 (t, *J* = 6.2 Hz, 1H, H_2), 6.73 (d, *J* = 8.0 Hz, 2H, H_{19}), 4.86 – 4.75 (m, 1H, H_5), 4.55 – 4.36 (m, 2H, $H_{16,25}$), 4.31 – 4.21 (m, 1H, H_{14a}), 4.04 – 3.89 (m, 1H, H_{14b}), 3.83 (dt, *J* = 16.6, 6.9 Hz, 1H, H_{23a}), 3.60 – 3.56 (m, 3H, H_{30}), 3.53 – 3.34 (m, 35H, $H_{3,23b,31}$), 3.26 – 3.17 (m, 1H, H_{6a}), 3.06 – 2.78 (m, 5H, $H_{6b,17,34}$), 1.36 (s, 9H, H_1), 1.27 – 1.17 (m, 3H, H_{20}), 1.04 (d, *J* = 7.4 Hz, 18H, H_{21}). **^{13}C NMR** (125 MHz, DMSO-*d*$_6$, 298 K) δ 171.85, 171.81, 171.22, 171.19, 170.50, 170.38, 169.73, 169.25, 168.56, 168.51, 168.15, 155.61, 153.72, 153.69, 137.20, 137.07, 135.97, 130.42, 130.33, 130.26, 130.01, 129.89, 129.85, 129.09, 129.06, 128.26, 128.21, 128.17, 127.86, 126.54, 126.49, 123.18, 120.57, 119.15, 119.12, 118.50, 118.44, 118.03, 111.05, 110.47, 110.38, 77.98, 69.76, 69.67, 54.69, 54.00, 53.58, 53.56, 52.40, 52.27, 51.86, 51.83, 43.35, 42.71, 41.41, 41.29, 36.89, 36.81, 36.46, 28.16, 17.73, 12.03. **HRMS** (ESI$^+$) Calculated for $C_{66}H_{102}O_{18}N_7Si$ $[M+H]^+$ = 1308.7045, found 1308.7023.

Synthesis of *0:1:0*-**24C8⊂16**

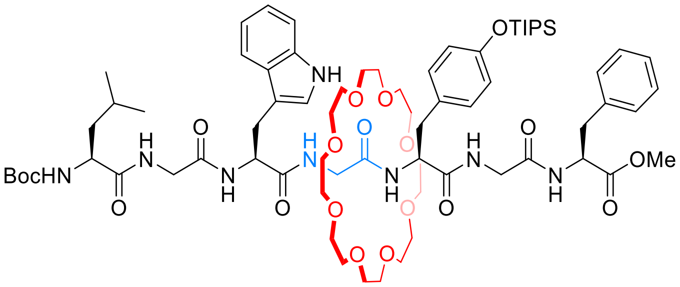

To a solution of **24C8⊂S15** (52 mg, 0.04 mmol, 1.0 equiv.) in dichloromethane (2 mL) was added CF_3CO_2H (0.5 mL). The solution was stirred at room temperature for 1 hour before toluene (5 mL) was added and the mixture was concentrated to dryness under reduced pressure. The crude salt (assumed 0.04 mmol) was dissolved in dichloromethane (5 mL). Boc-Leu-OH (11.5 mg, 0.05 mmol, 1.0 equiv.), HOBt·H_2O (9.2 mg, 0.06 mmol, 1.2 equiv.) and EDC·HCl (14.4 mg, 0.075 mmol, 1.5 equiv.) were added and stirred for 30 minutes at 0 °C. *N*,*N*-Diisopropylethylamine (17.6 μL, 0.1 mmol, 2.5 equiv.) was added to the reaction mixture and stirred for another 12 hours at room temperature. The liquors were concentrated under reduced pressure and H_2O (5 mL) was added. The resulting solution was extracted with dichloromethane (3 × 5 mL). The combined organic extracts were washed with brine (5 mL), dried with $MgSO_4$ and concentrated under reduced pressure. Flash column chromatography of the crude residue (SiO_2, acetone/hexane 1:1) afforded *0:1:0*-**24C8⊂16** (46 mg, 0.032 mmol, 81%) as a white solid. Data is consistent with that of *0:1:0*-**24C8⊂16** prepared in section 3.1.

4. ^{1}H NMR analysis of 24C8⊂5

The incorporation of the crown ether macrocycle leads to an increase in the diastereotopic H_b splitting observed for H_c (3.21 and 2.59 ppm for **24C8⊂5** compared to 3.08 and 2.98 ppm for **5**), which coupled with a large difference in the observed $^{3}J_{HaHb}$ values between H_b and H_c (4.4 and 10.9 Hz in **24C8⊂5** compared to 8.8 and 9.4 Hz in **3**), indicates a significant restriction in rotation of the tyrosine sidechain on the ^{1}H NMR timescale (Figure S1). This increase in restriction also leads to the appearance of amide rotamers (labeled with a* in Figure S1). Small peaks in a 1:4 ratio to the parent peaks are observed, which coalesce at 343 K when analyzed by variable temperature (VT)-NMR (see Figure S2).

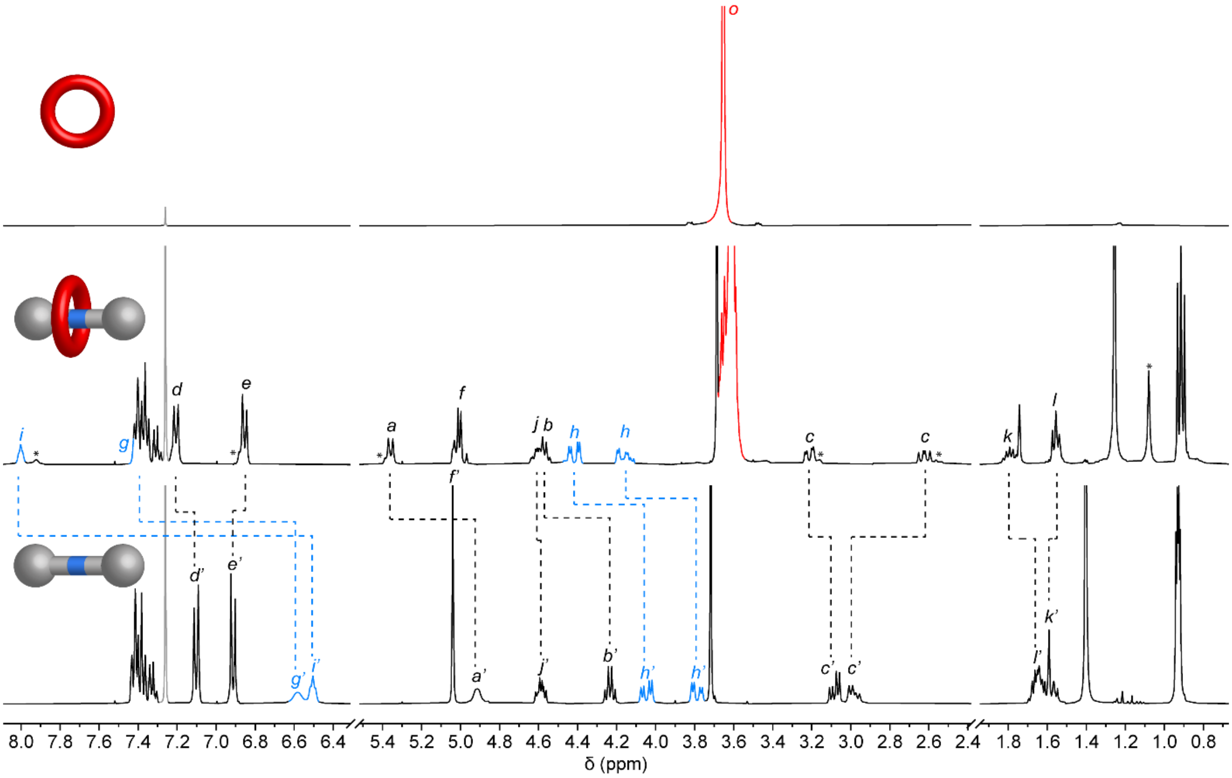

Figure S1. Partial ^{1}H NMR spectra (400 MHz, $CDCl_3$, 298K) of **24C8** (top), **24C8⊂5** (middle), and **5** (bottom). The region of 6.1 – 8.1 ppm is scaled vertically 1.5× compared to the region of 0.7 – 5.5 ppm. Peaks in grey correspond to residual $CHCl_3$. Smaller peaks suffixed with * are amide rotamers of **24C8⊂5**.

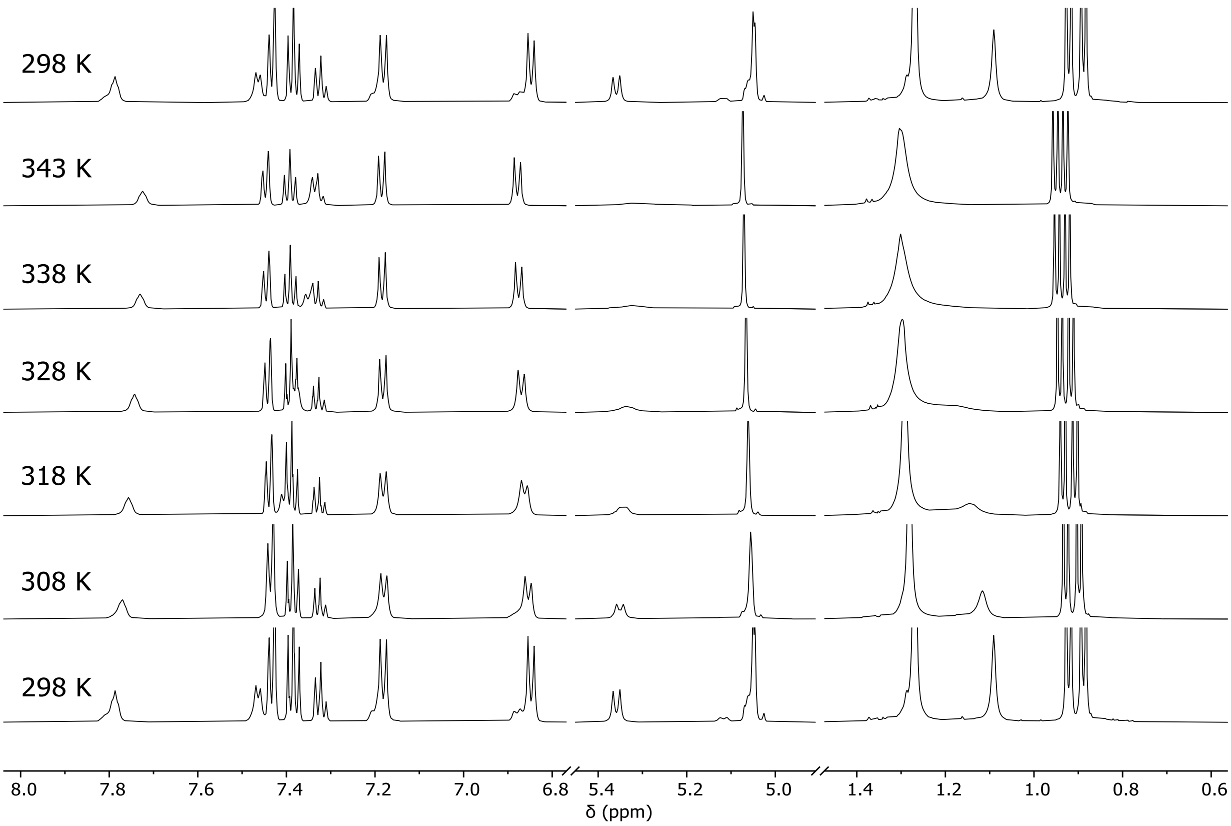

Figure S2. Variable temperature ^{1}H NMR analysis of **24C8⊂5** (600 MHz, CD_3CN). Peak coalescence occurs upon warming the sample to 338 K. Cooling the sample back to 298 K leads to re-emergence of rotamer peaks.

5. Limitations

5.1. Direct formation on substituted amino acids.

Reaction of H-Phe-OMe with Boc-Phe-ONp in the presence of **24C8** does not yield the desired rotaxane, instead the dipeptide is formed preferentially.

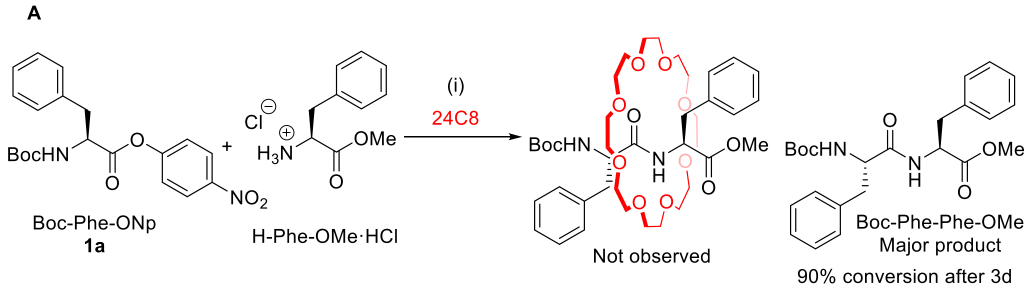

Scheme S8. Attempted peptide-rotaxane formation with branched amino acid nucleophile H-Phe-OMe. Reagents and conditions: (i) **24C8** (1 eq.), Et_3N (2.5 eq.), toluene, rt, 72 h.

5.2. Reaction with bulky activated esters.

Reaction of H-Gly-Phe-OtBu with Boc-Val-ONp in the presence of **24C8** does not yield the desired rotaxane, instead the tripeptide is formed preferentially. The crown ether acts as a moderate catalyst in this instance with a slight increase in the formation of the tripeptide when compared to the background reaction.

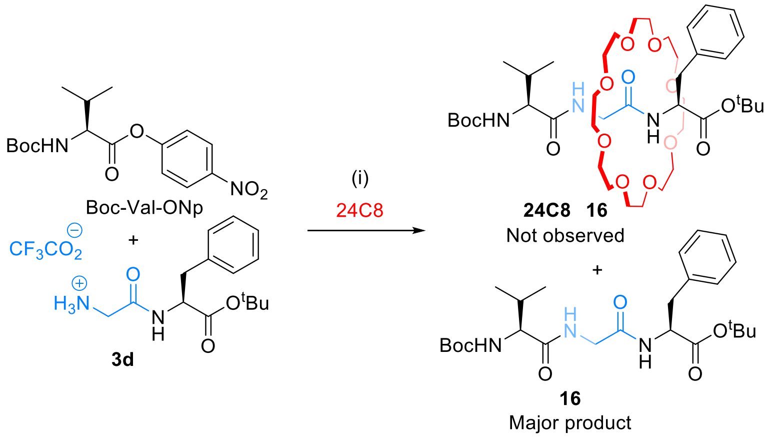

Scheme S9. Attempted peptide-rotaxane formation with valine activated ester **1i**. Reagents and conditions: (i) **24C8** (1 eq.), Et_3N (1 eq.), toluene, rt, 72 h.

6. CD and UV spectra of *1:0:0*-24C8⊂16, *0:1:0*-24C8⊂16, *0:0:1*-24C8⊂16 and 16

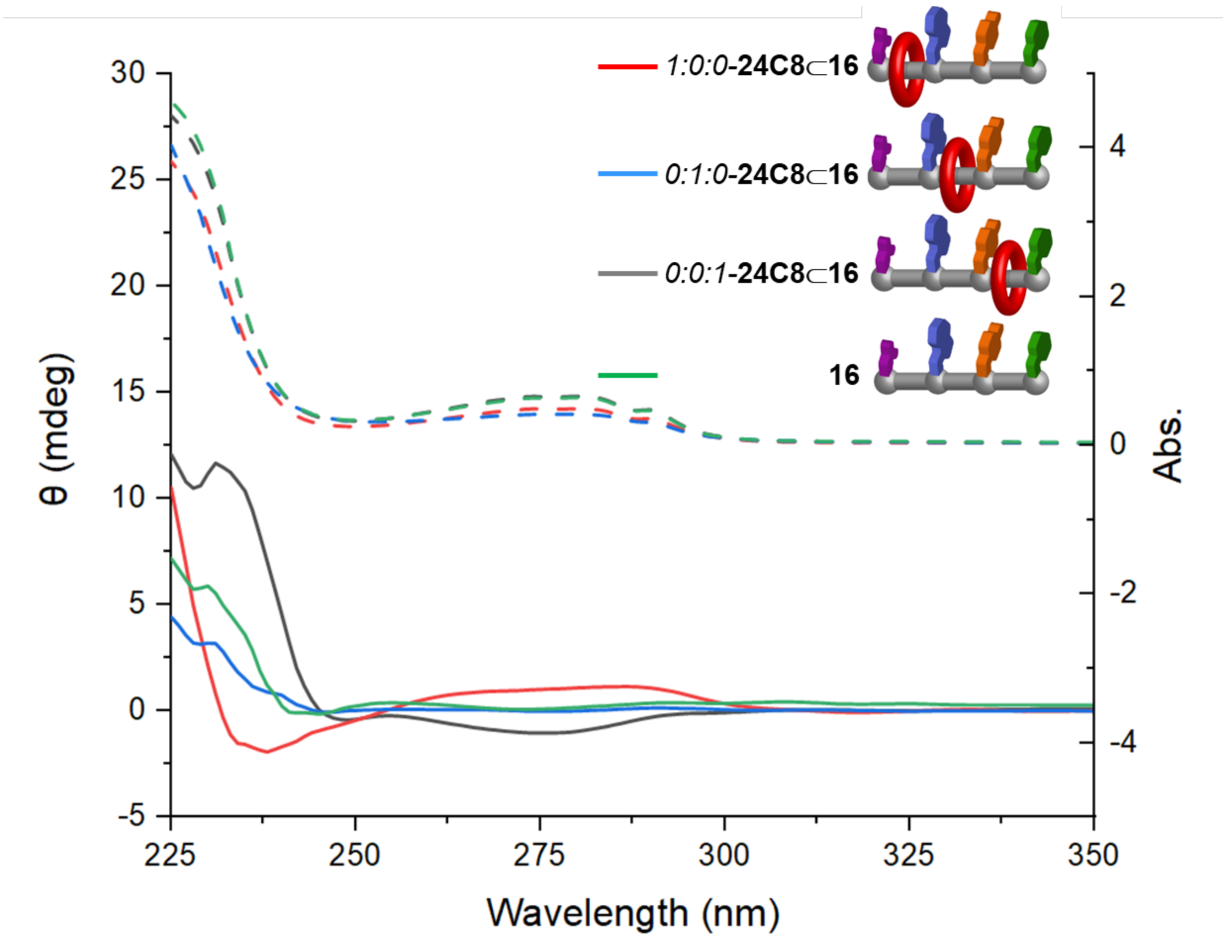

Figure S3. Overlay of the UV−vis (dashed lines, 5 × 10^{-4} M, CH_2Cl_2, 298 K) and CD spectra (solid lines, 5 × 10^{-4} M, CH_2Cl_2, 298 K) for isomers *1,0,0*-**24C8⊂16** (red line), *0,1,0*-**24C8⊂16** (blue line), *0,0,1*-**24C8⊂16** (grey line) and heptapeptide thread **16** (green line).

7. Pronase hydrolysis of peptide rotaxanes

A solution of 1 mg of peptide was diluted with 2 mL of a buffer (Phosphate buffer saline, pH 7.4). To this solution was added Pronase dissolved in the same buffer (1.0 mg/mL, 100 µL). The mixture was incubated at 37 °C for 24 h and analyzed at selected time points: 0.2, 0.6, 1.5, 2.5, 3.5, 4.5, 6, 8 and 24 hours for peptide rotaxane isomers. For analysis, an aliquot (30 µL) was taken from the reaction mixture, diluted with H_2O/MeOH (1:1, 30 µL, 0.1% TFA) and injected to analytical RP-HPLC. The peak area of the starting peptide (At) was determined by integration (Figure S1). The percentage of the remaining peptide at each time point was calculated as follows: Peptide remaining (%) = (A_t /A_0) × 100. Each reaction was further analyzed crude by LRMS. 0.1 mL aliquots of the crude reaction mixture were diluted with MeOH (1 mL) then analyzed by LRMS (ESI^+) (Figures S2-S4).

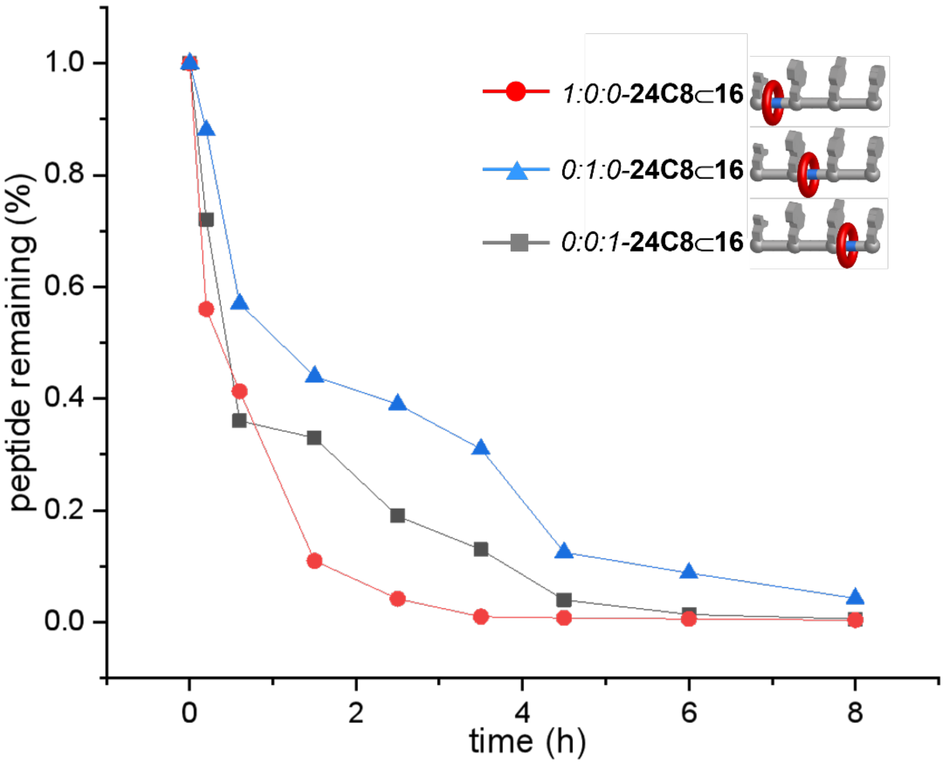

Figure S4. Plots of time vs conversion (Pronase assay).

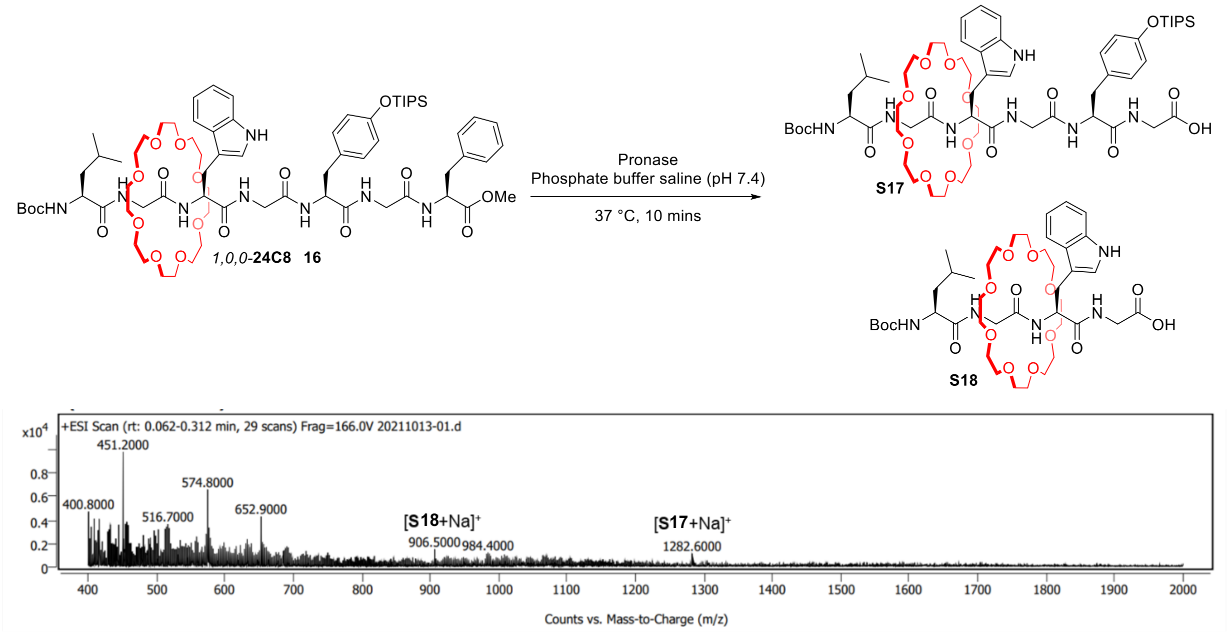

Figure S5. MS (ESI$^+$) of the crude reaction mixture of the Pronase mediated hydrolysis of *1:0:0*-**24C8⊂16** (aliquot taken after 10 minutes).

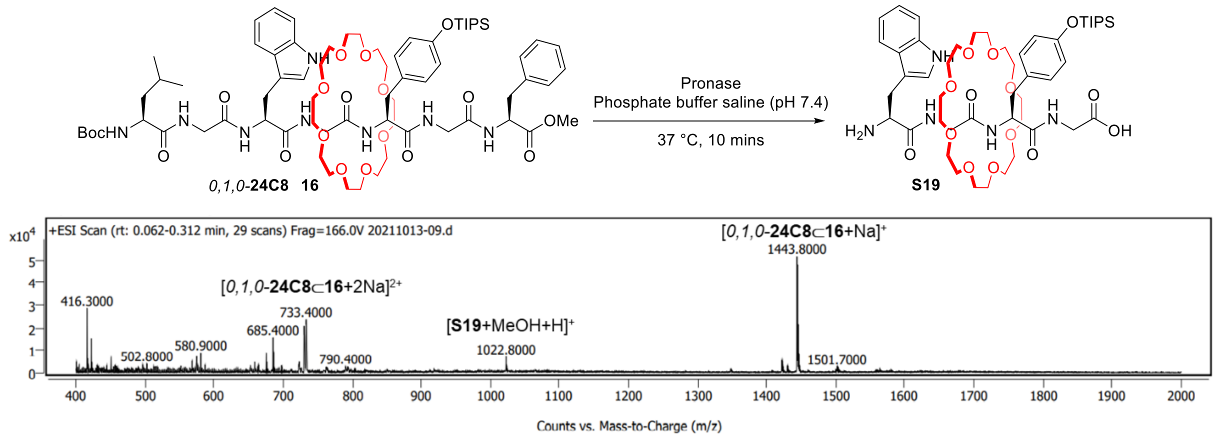

Figure S6. MS (ESI$^+$) of the crude reaction mixture of the Pronase mediated hydrolysis of *0:1:0*-**24C8⊂16** (aliquot taken after 1 hour).

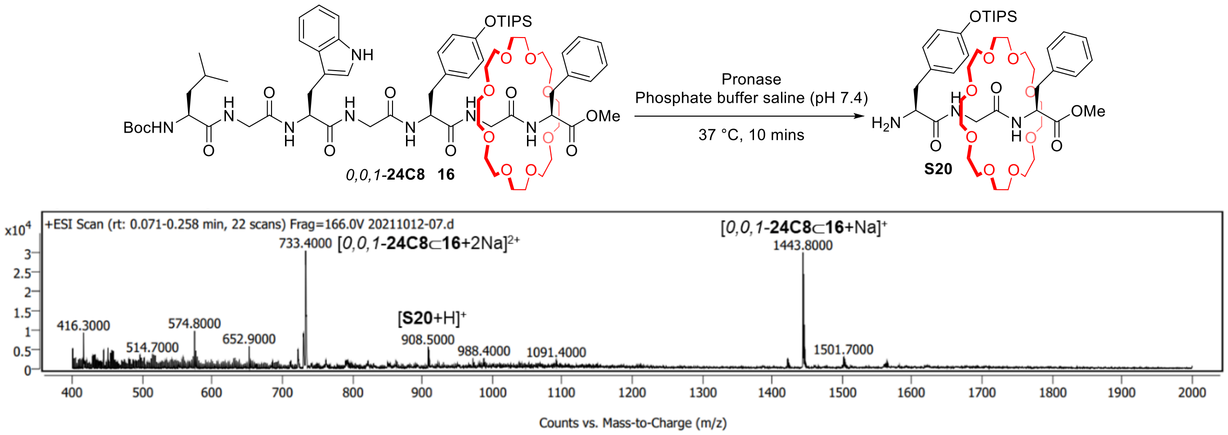

Figure S7. MS (ESI$^+$) of the crude reaction mixture of the Pronase mediated hydrolysis of *0:0:1*-**24C8⊂16** (aliquot taken after 10 minutes).

8. Tandem MS/MS analysis of 24C8⊂4, 16, *1:0:0*-24C8⊂16, *0:1:0*-24C8⊂16 and *0:0:1*-24C8⊂16.

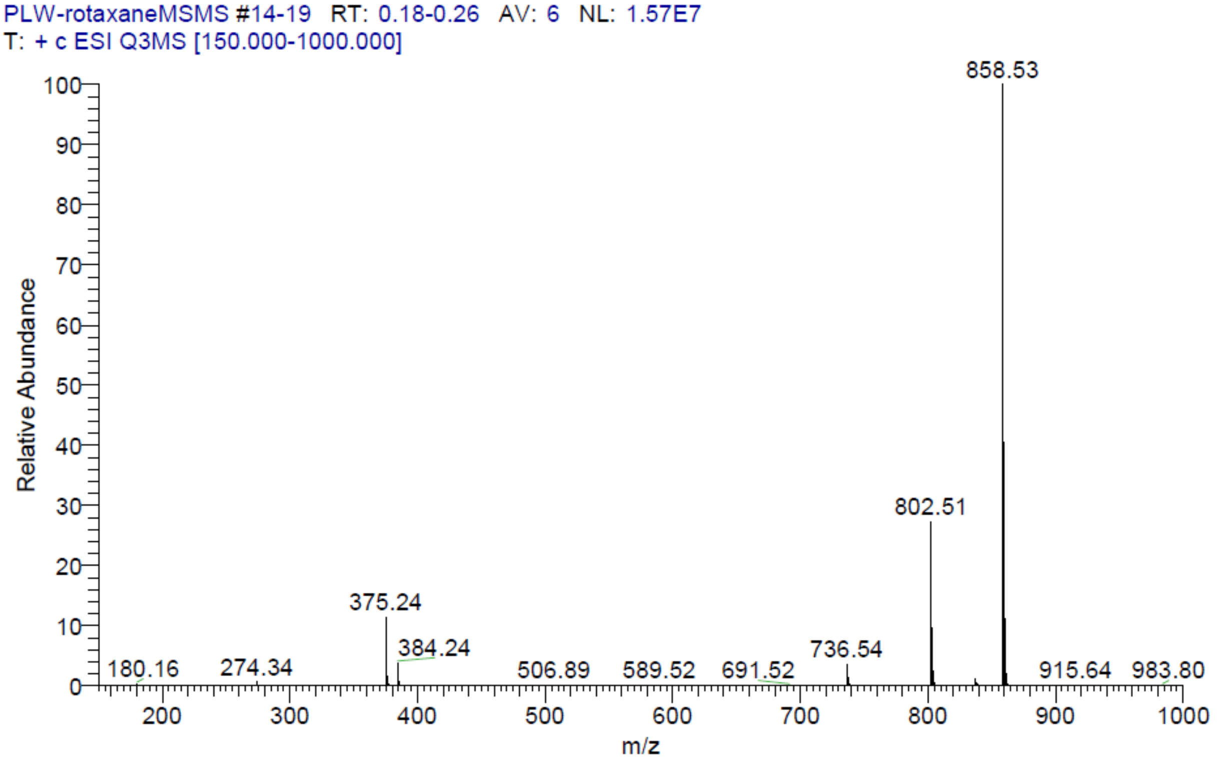

Figure S8. ESI-MS spectrum of **24C8⊂4** shows the m/z 858 precursor ion [M+Na]$^+$.

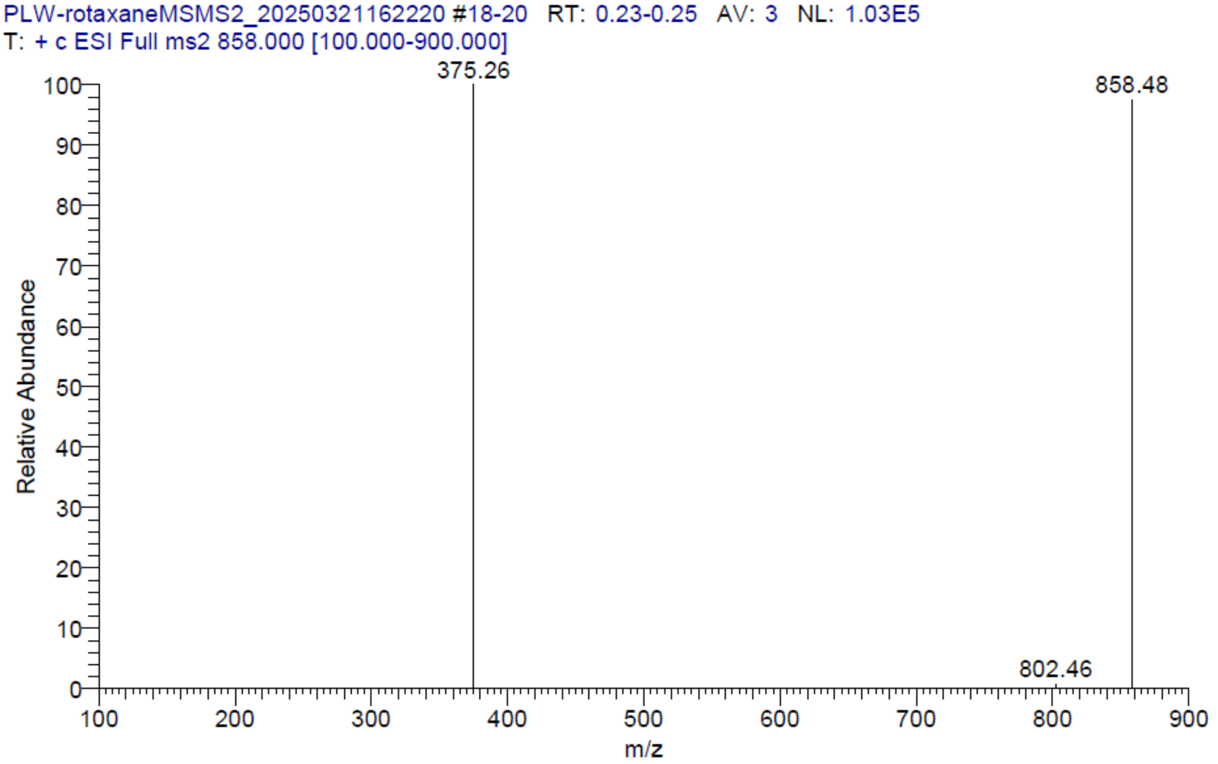

Figure S9. ESI-MS/MS spectrum of **24C8⊂4** after collisional activation of the m/z 858 precursor ion [M+Na]$^+$, the signal (m/z 375) corresponds to [24C8+Na]$^+$.

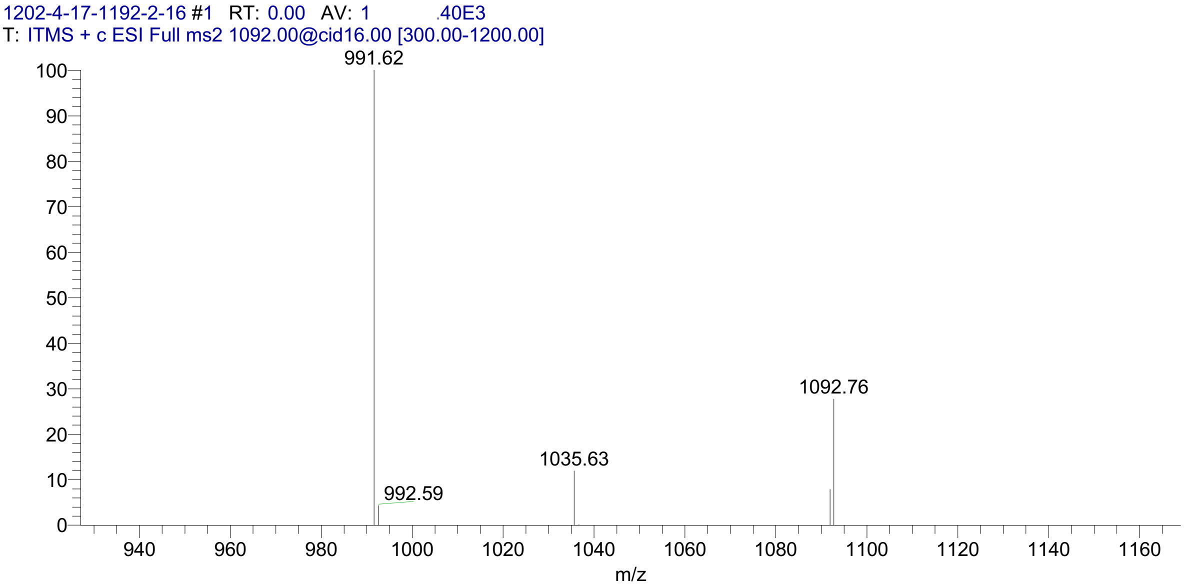

Figure S10. ESI-MS/MS spectrum of thread **16** after collisional activation of the m/z 1092 precursor ion $[M+Na]^+$.

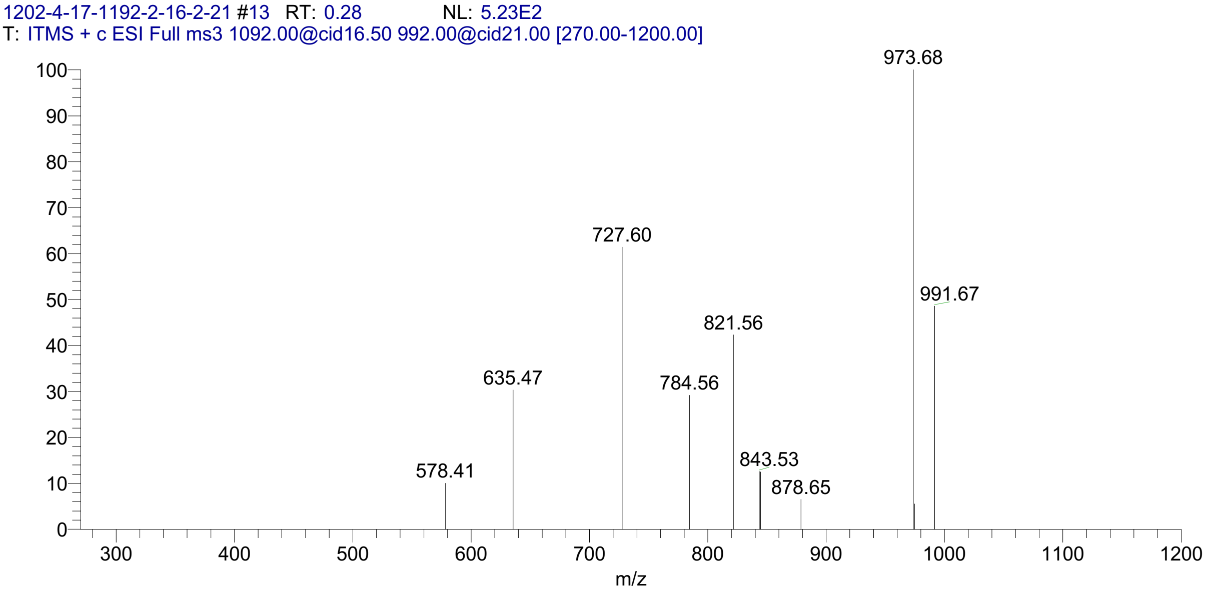

Figure S11. ESI-MS/MS spectrum of thread **16** after collisional activation of the peak at m/z 992.

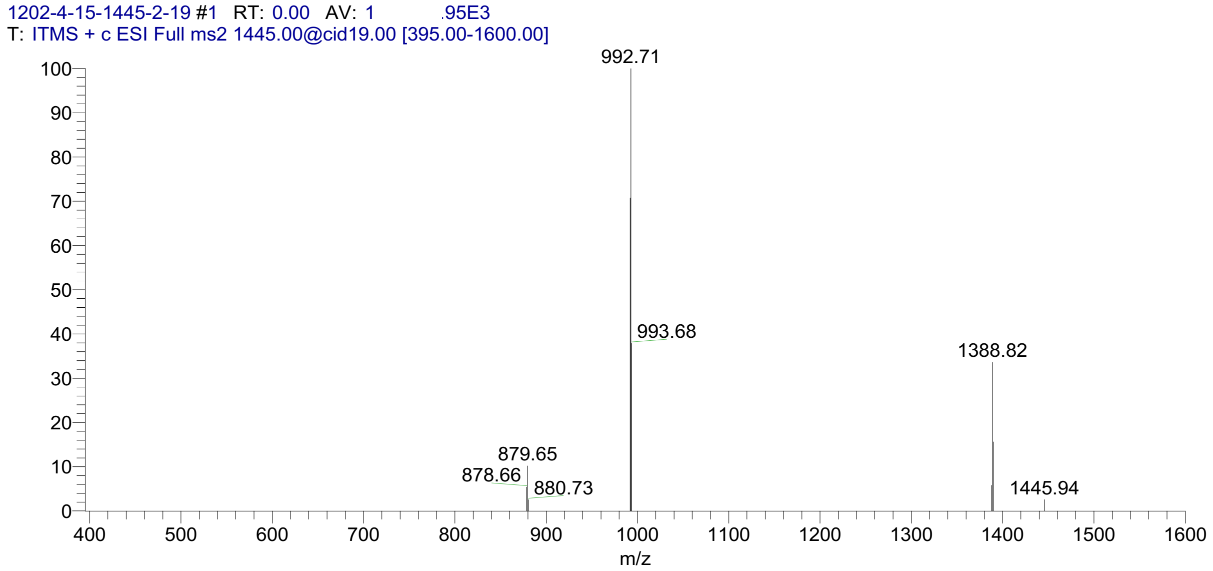

Figure S12. ESI-MS/MS spectrum of *1:0:0*-**24C8⊂16** after collisional activation of the m/z 1445 precursor ion [M+Na]$^+$.

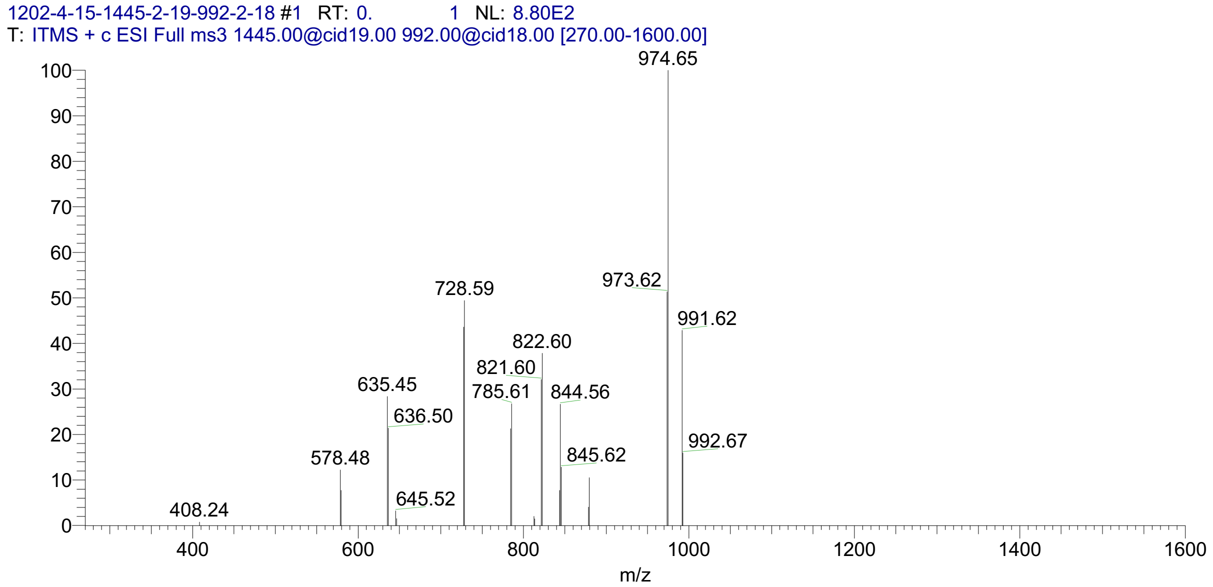

Figure S13. ESI-MS/MS spectrum of *1:0:0*-**24C8⊂16** after collisional activation of the peak at m/z 992.

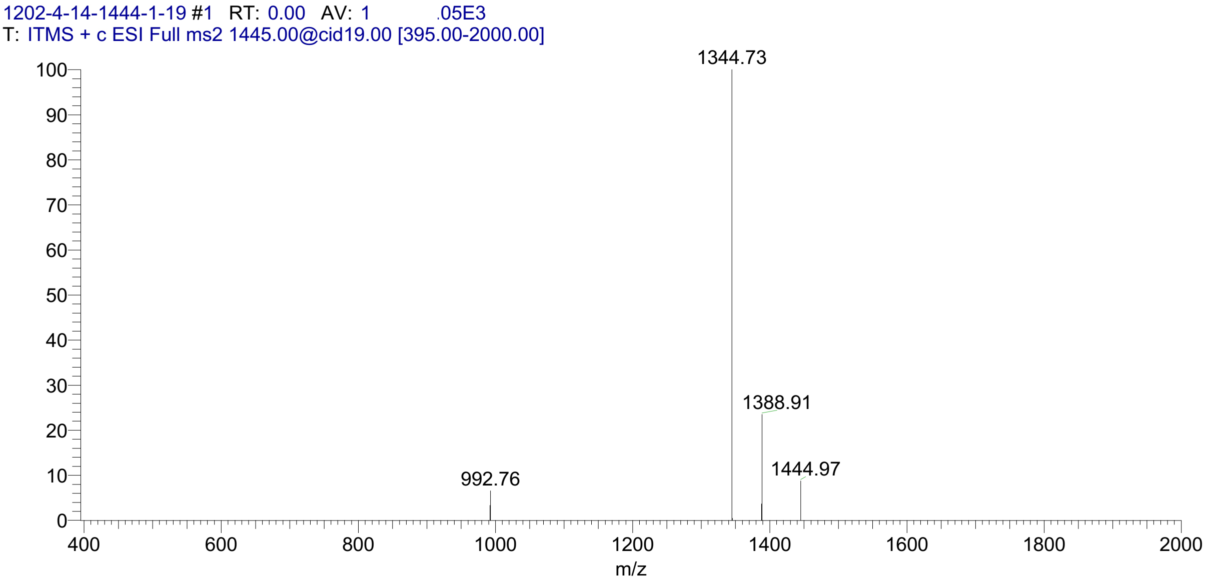

Figure S14. ESI-MS/MS spectrum of *0:1:0*-**24C8⊂16** after collisional activation of the m/z 1445 precursor ion [M+Na]$^+$.

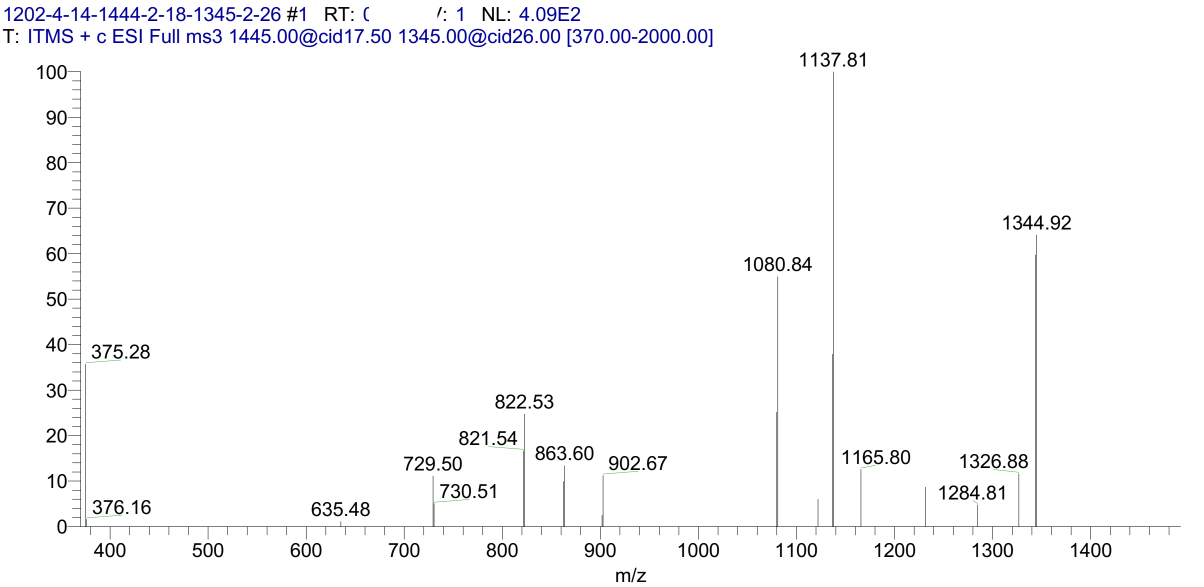

Figure S15. ESI-MS/MS spectrum of *0:1:0*-**24C8⊂16** after collisional activation of the peak at m/z 1345.

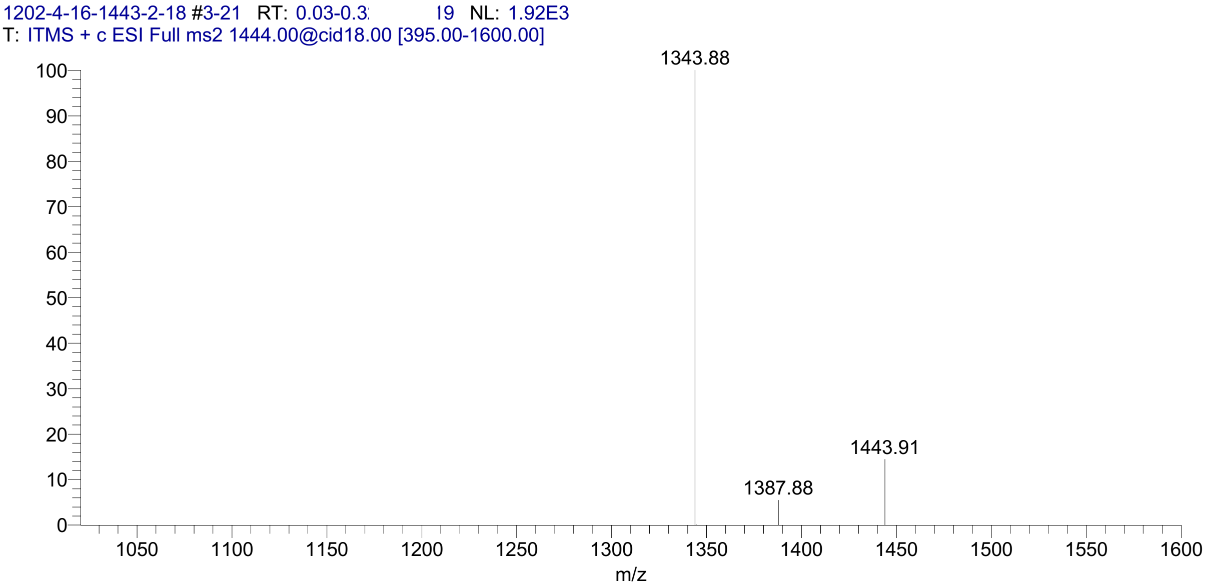

Figure S16. ESI-MS/MS spectrum of *0:0:1*-**24C8⊂16** after collisional activation of the m/z 1444 precursor ion [M+Na]$^+$.

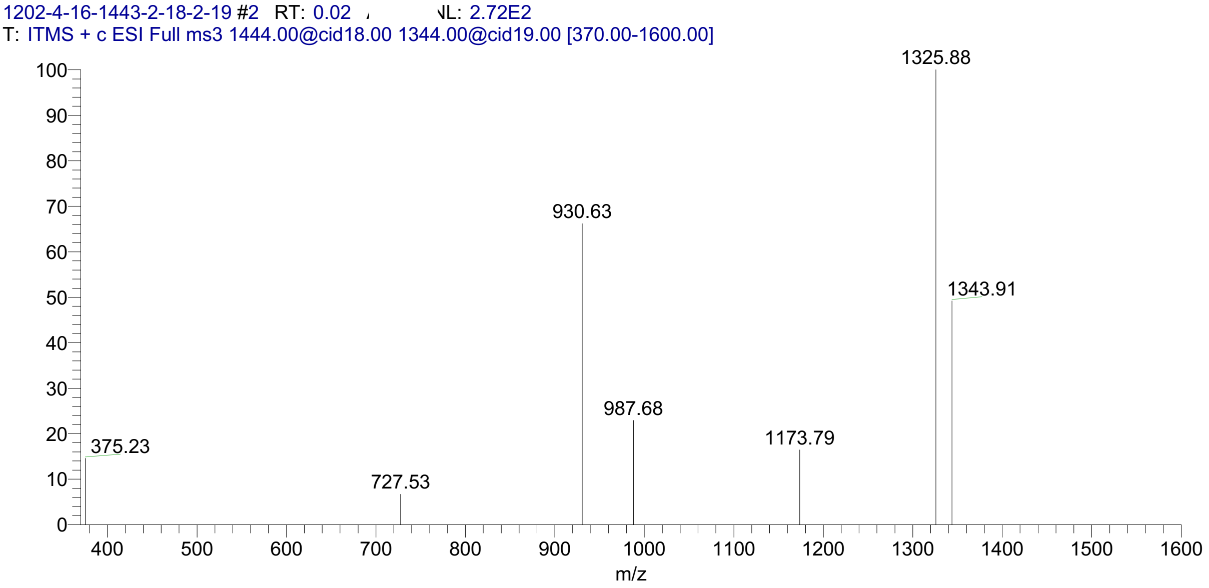

Figure S17. ESI-MS/MS spectrum of *0:0:1*-**24C8⊂16** after collisional activation of the peak at m/z 1344.

9. Molecular modelling of 24C8⊂4

Computational studies were performed on the rotaxane **24C8⊂4** using the Gaussian 16 software package at the Computational Shared Facility 3 (CSF3) of the University of Manchester. Geometry optimizations were carried out at DFT level using hybrid B3LYP functional with a 6-31G basis set. Empirical dispersion correction (gd3bj) was introduced to account for the H-bonding between the thread and the macrocycle and chloroform was included as the solvent using the Polarization Continuum Model (PCM). Frequency calculations were performed at the same level to evaluate the zero-point vibrational energy and thermal corrections at 298 K (Table S1).

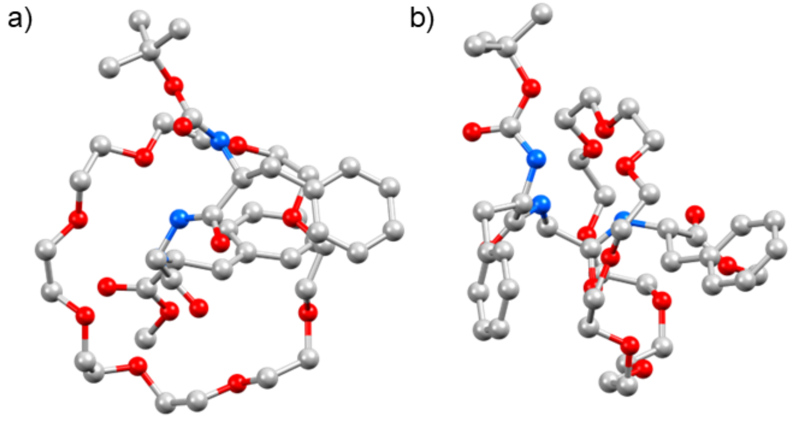

Figure S18: Energy optimized structure (DFT; hybrid B3LYP functional with 6-31G basis set) of the rotaxane **24C8⊂4** in $CHCl_3$. a) Top view; b) Side-on view. Hydrogen atoms are omitted for clarity (Element color: nitrogen-blue, oxygen-red, and carbon-grey).

Table S1: Calculated energy values for the rotaxane **24C8⊂4.**

**Parameters**	**Energy (Hartree/Particle)**
Zero-point correction	1.072336(Hartree/Particle)
Thermal correction to Energy	1.134034
Thermal correction to Enthalpy	1.134978
Thermal correction to Gibbs Free Energy	0.974496
Sum of electronic and zero-point Energies	-2855.36
Sum of electronic and thermal Energies	-2855.3
Sum of electronic and thermal Enthalpies	-2855.3
Sum of electronic and thermal Free Energies	-2855.46

References:

S1) Gaussian 16, Revision C.01, M. J. Frisch, G. W. Trucks, H. B. Schlegel, G. E. Scuseria, M. A. Robb, J. R. Cheeseman, G. Scalmani, V. Barone, G. A. Petersson, H. Nakatsuji, X. Li, M. Caricato, A. V. Marenich, J. Bloino, B. G. Janesko, R. Gomperts, B. Mennucci, H. P. Hratchian, J. V. Ortiz, A. F. Izmaylov, J. L. Sonnenberg, D. Williams-Young, F. Ding, F. Lipparini, F. Egidi, J. Goings, B. Peng, A. Petrone, T. Henderson, D. Ranasinghe, V. G. Zakrzewski, J. Gao, N. Rega, G. Zheng, W. Liang, M. Hada, M. Ehara, K. Toyota, R. Fukuda, J. Hasegawa, M. Ishida, T. Nakajima, Y. Honda, O. Kitao, H. Nakai, T. Vreven, K. Throssell, J. A. Montgomery, Jr., J. E. Peralta, F. Ogliaro, M. J. Bearpark, J. J. Heyd, E. N. Brothers, K. N. Kudin, V. N. Staroverov, T. A. Keith, R. Kobayashi, J. Normand, K. Raghavachari, A. P. Rendell, J. C. Burant, S. S. Iyengar, J. Tomasi, M. Cossi, J. M. Millam, M. Klene, C. Adamo, R. Cammi, J. W. Ochterski, R. L. Martin, K. Morokuma, O. Farkas, J. B. Foresman, and D. J. Fox, Gaussian, Inc., Wallingford CT, 2016.

10. NMR spectra

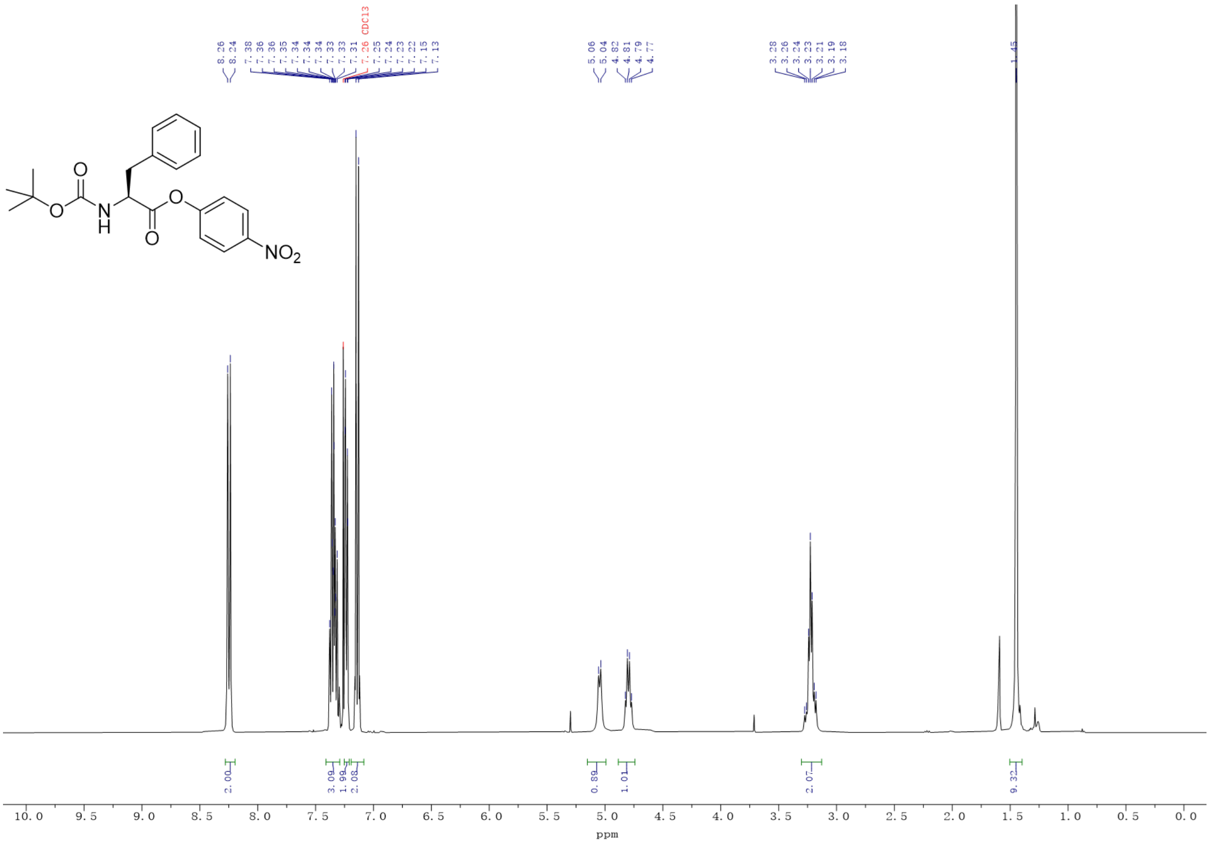

Spectrum S1: ^{1}H NMR (400 MHz, $CDCl_3$) spectrum of **1a**.

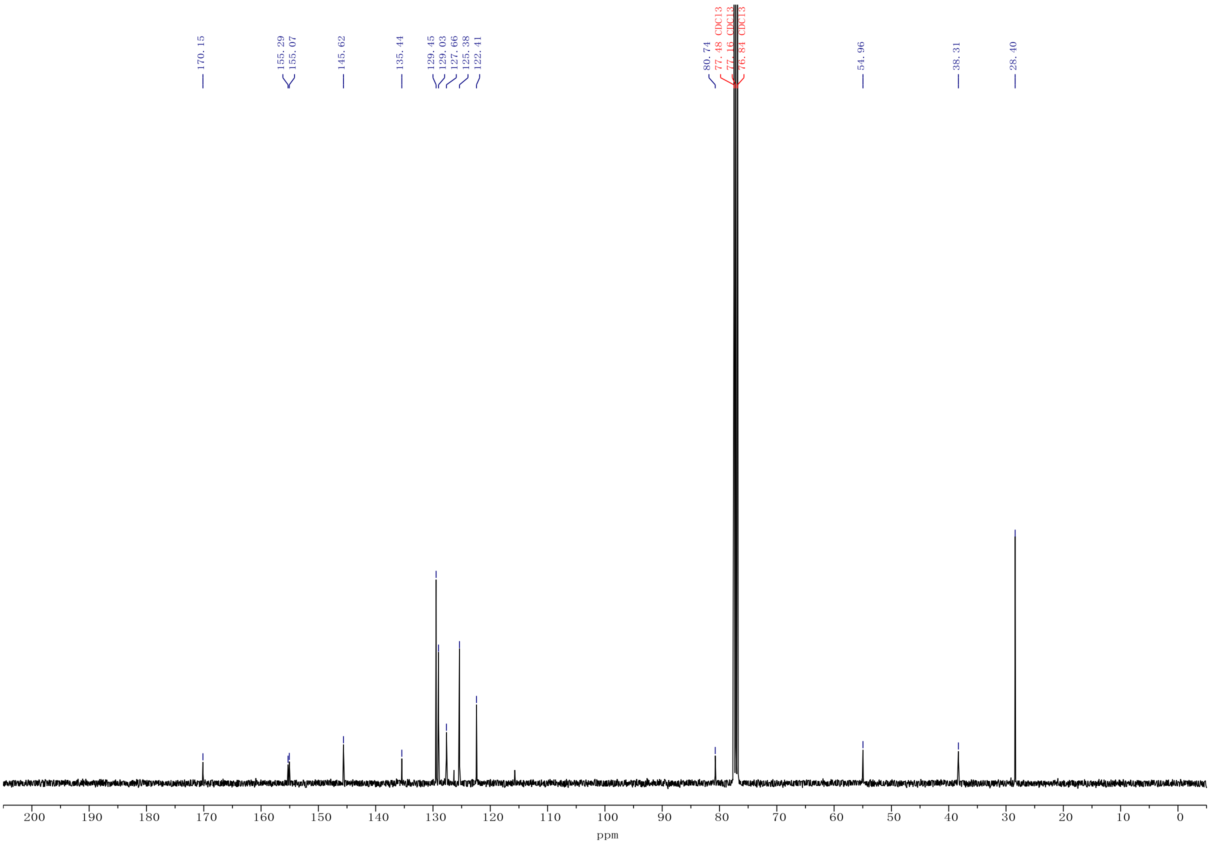

Spectrum S2: ^{13}C NMR (101 MHz, $CDCl_3$) spectrum of **1a**.

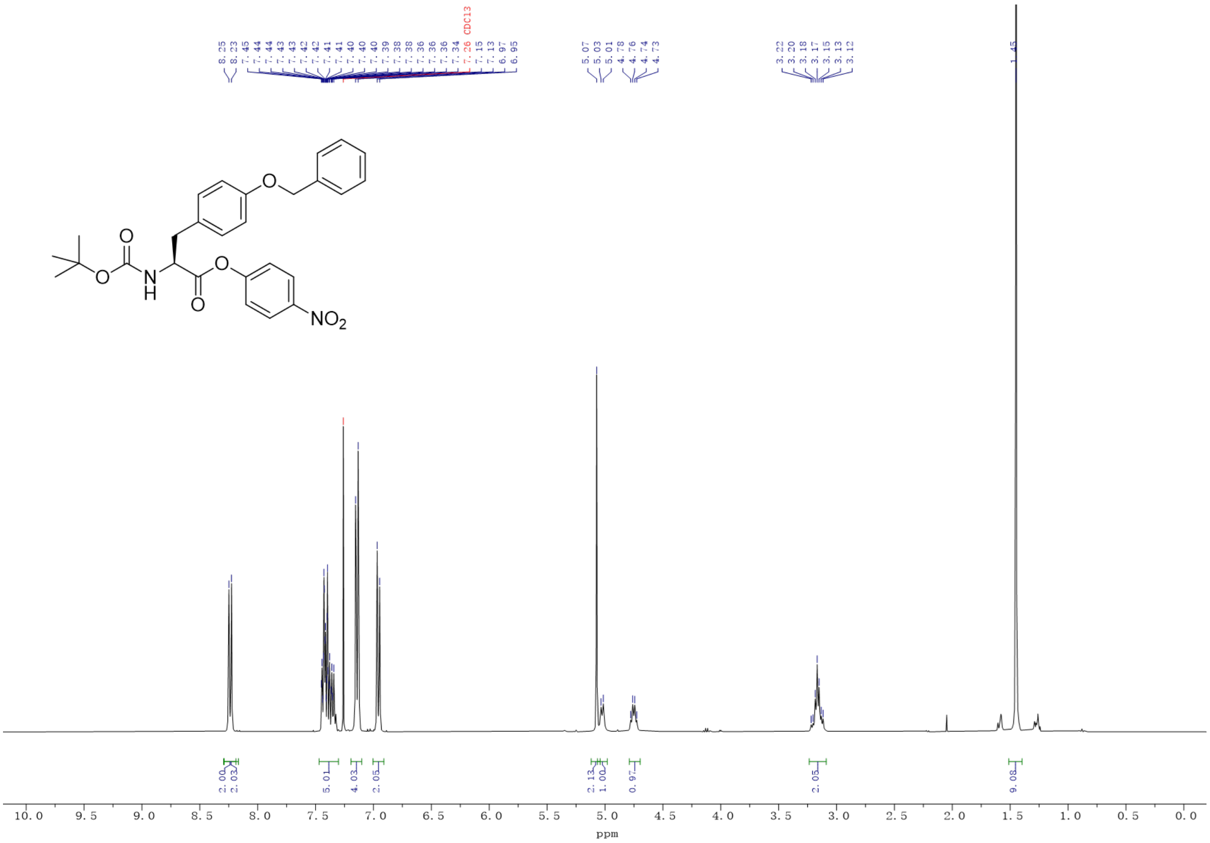

Spectrum S3: ^{1}H NMR (400 MHz, $CDCl_3$) spectrum of **1b**.

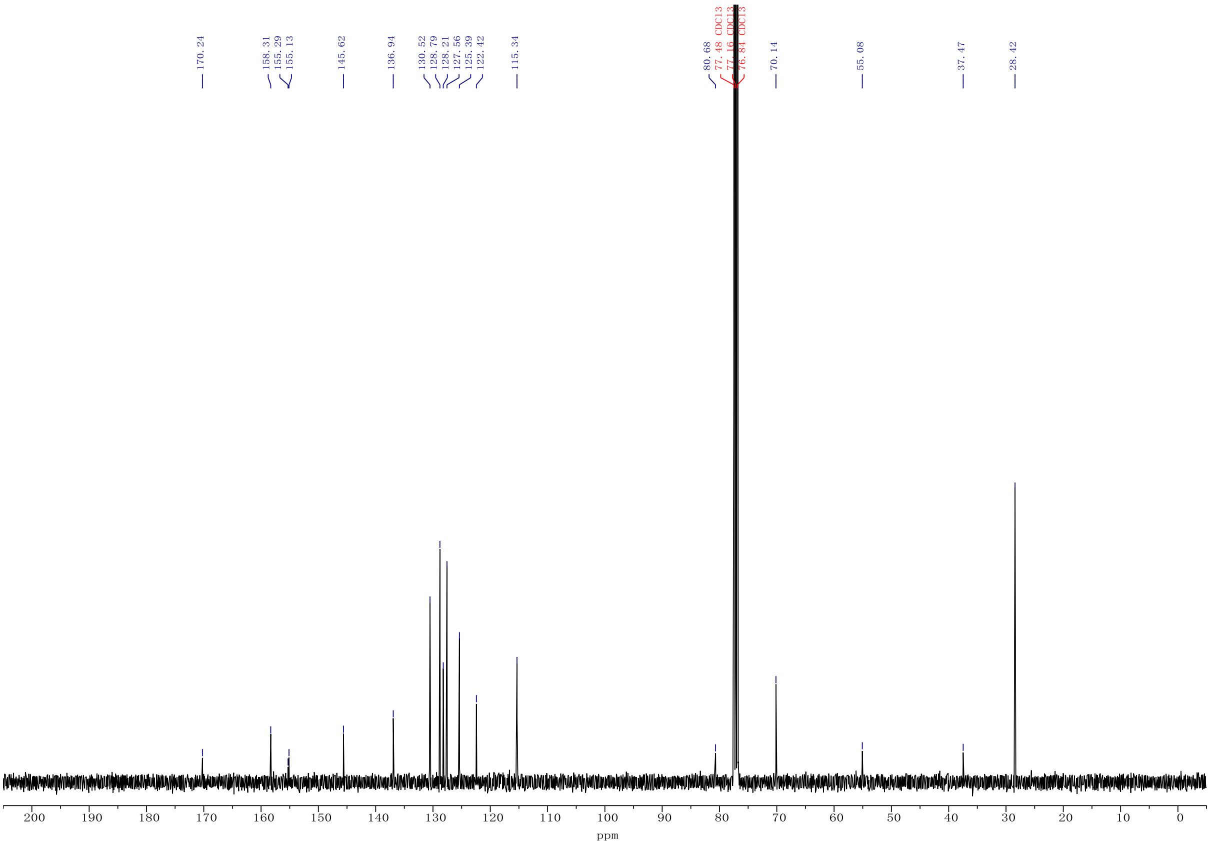

Spectrum S4: ^{13}C NMR (101 MHz, $CDCl_3$) spectrum of **1b**.

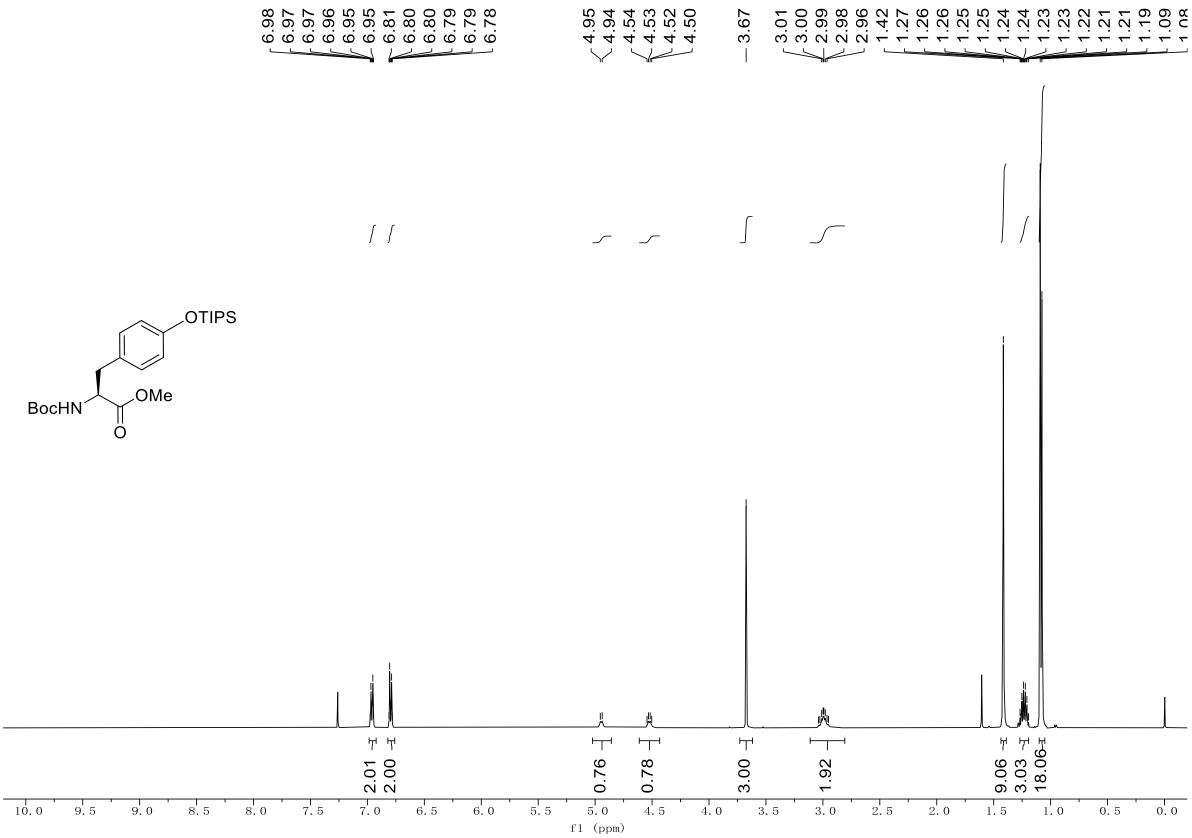

Spectrum S5: ^{1}H NMR (500 MHz, $CDCl_3$) spectrum of **S1**.

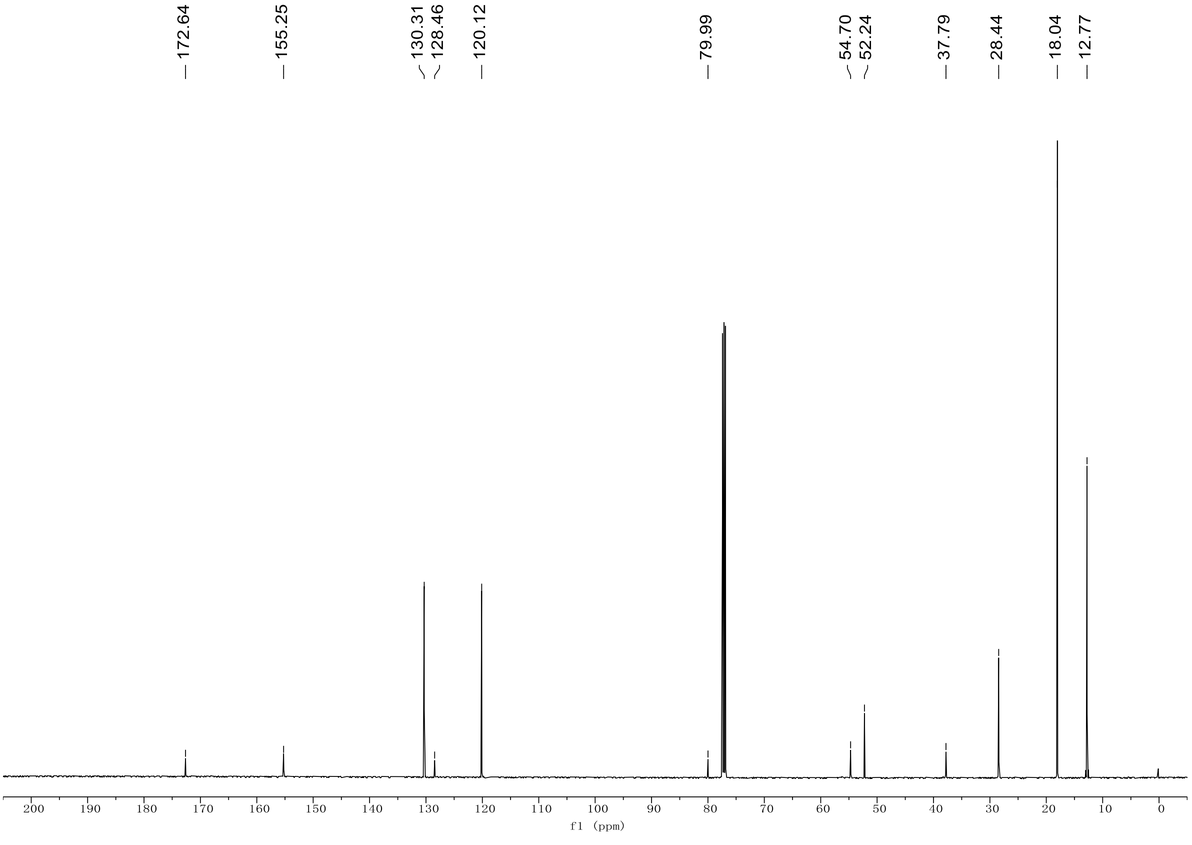

Spectrum S6: ^{13}C NMR (125 MHz, $CDCl_3$) spectrum of **S1**.

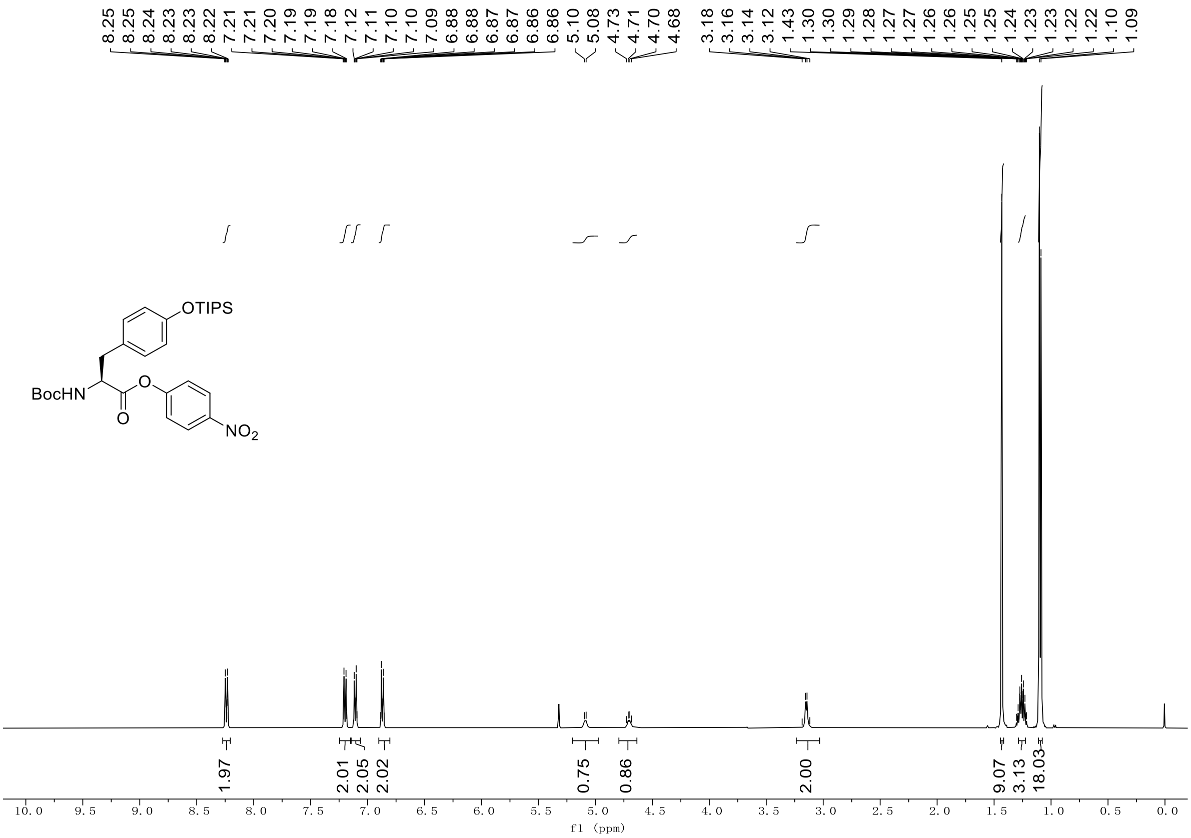

Spectrum S7: ^{1}H NMR (500 MHz, CD_2Cl_2) spectrum of **1c**.

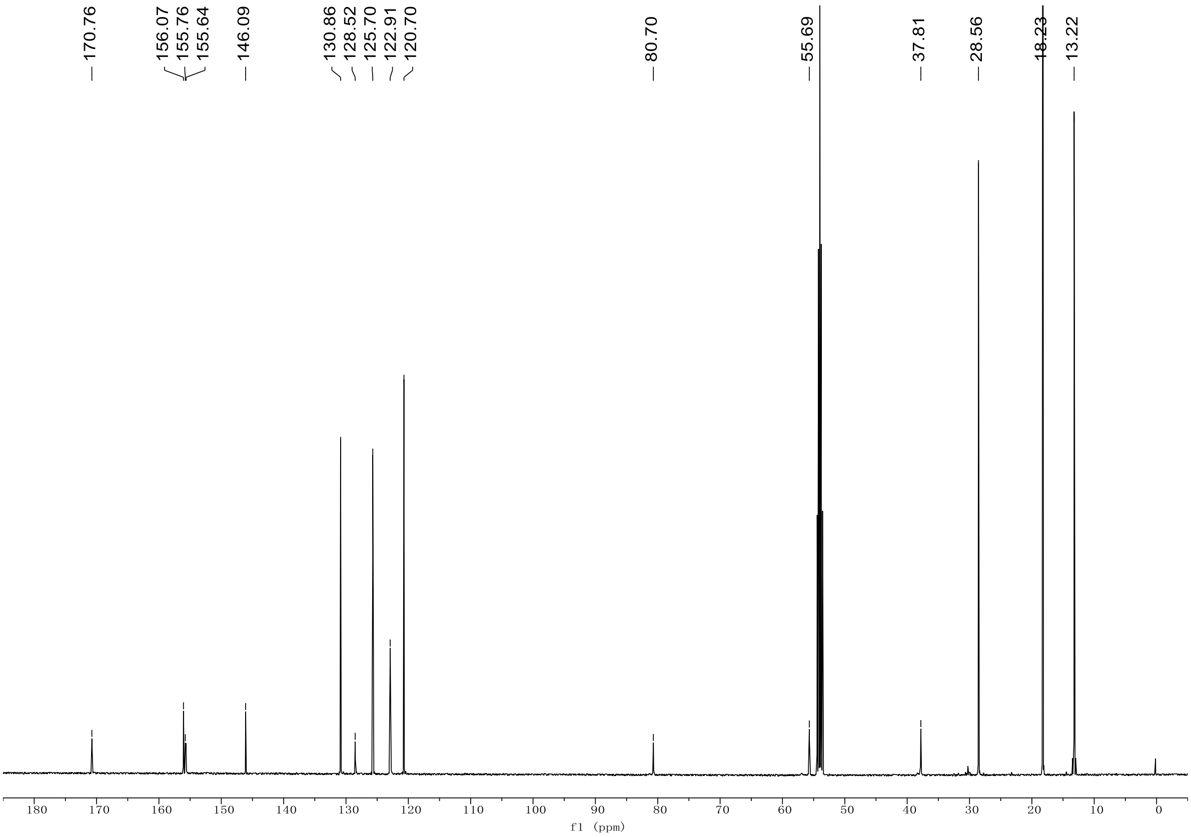

Spectrum S8: ^{13}C NMR (125 MHz, CD_2Cl_2) spectrum of **1c**.

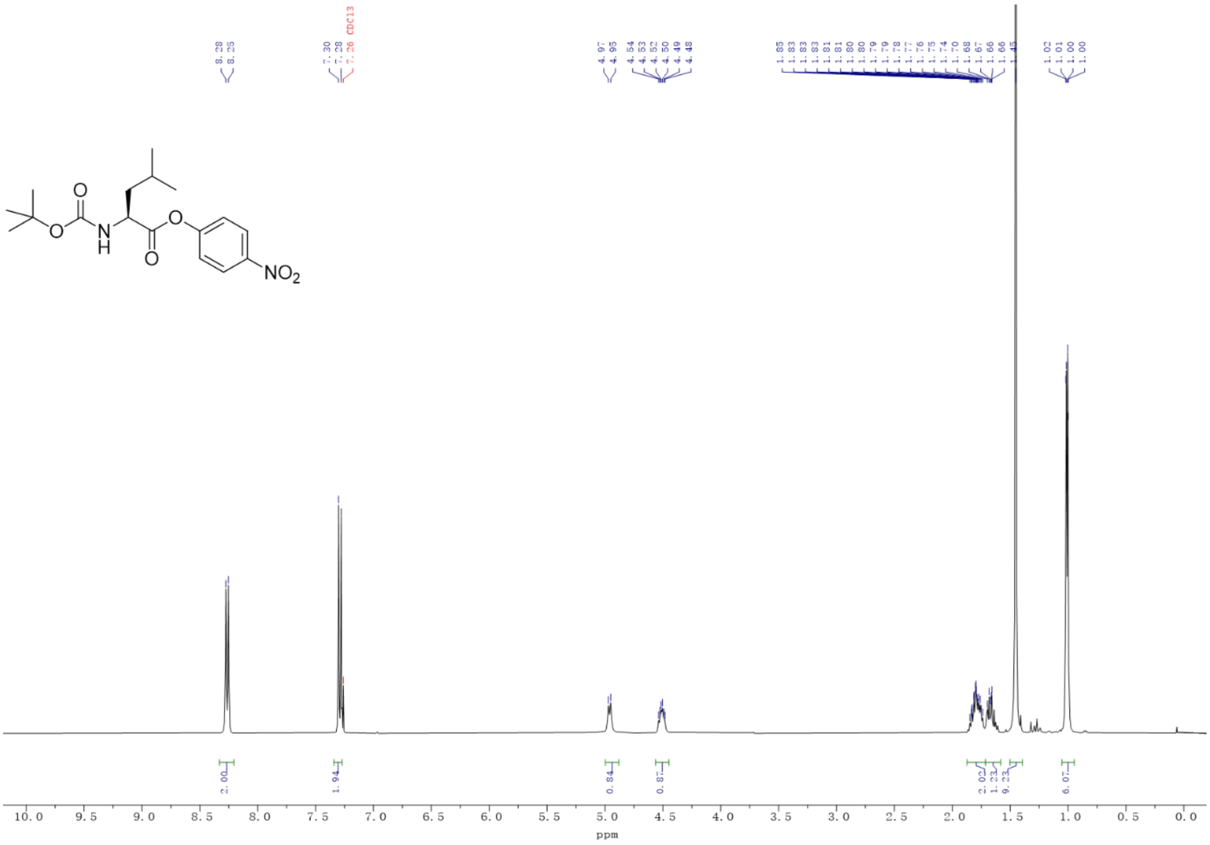

Spectrum S9: ^{1}H NMR (400 MHz, $CDCl_3$) spectrum of **1d**.

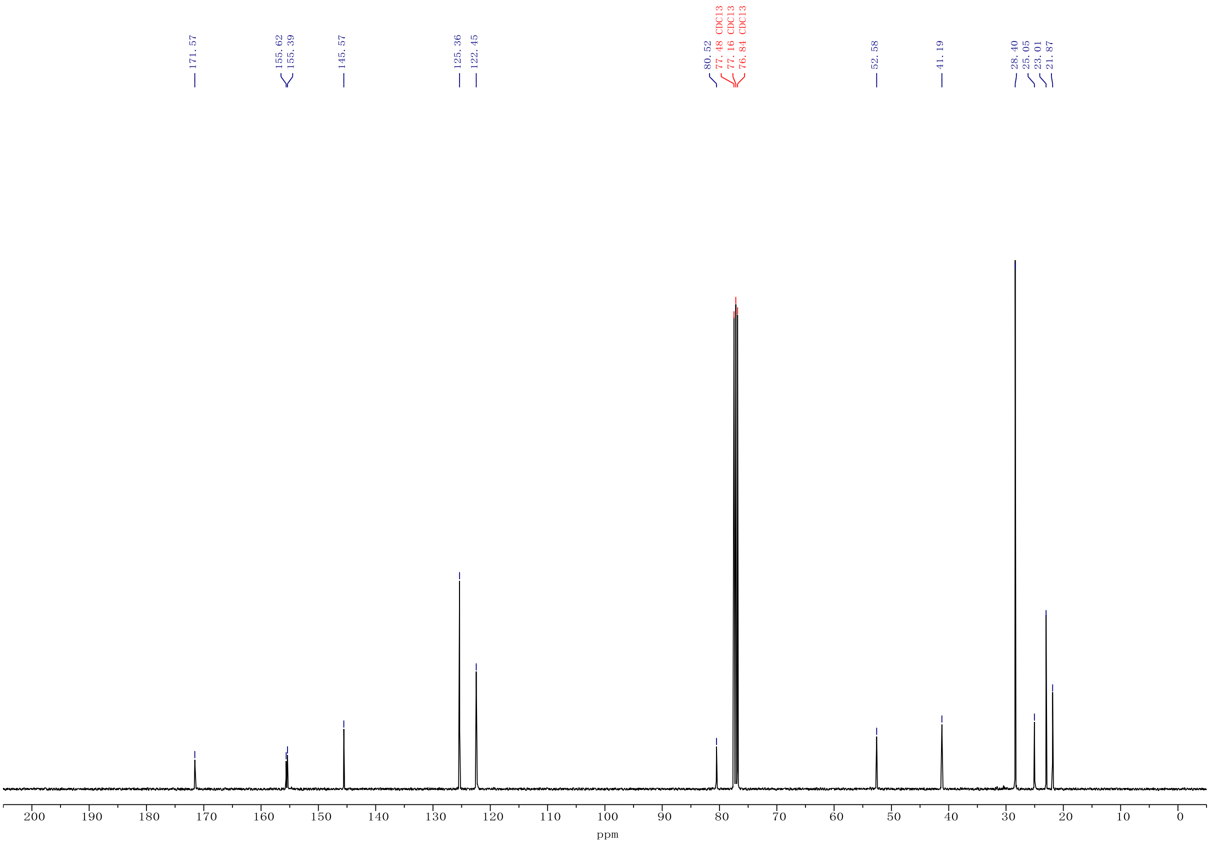

Spectrum S10: ^{13}C NMR (101 MHz, $CDCl_3$) spectrum of **1d**.

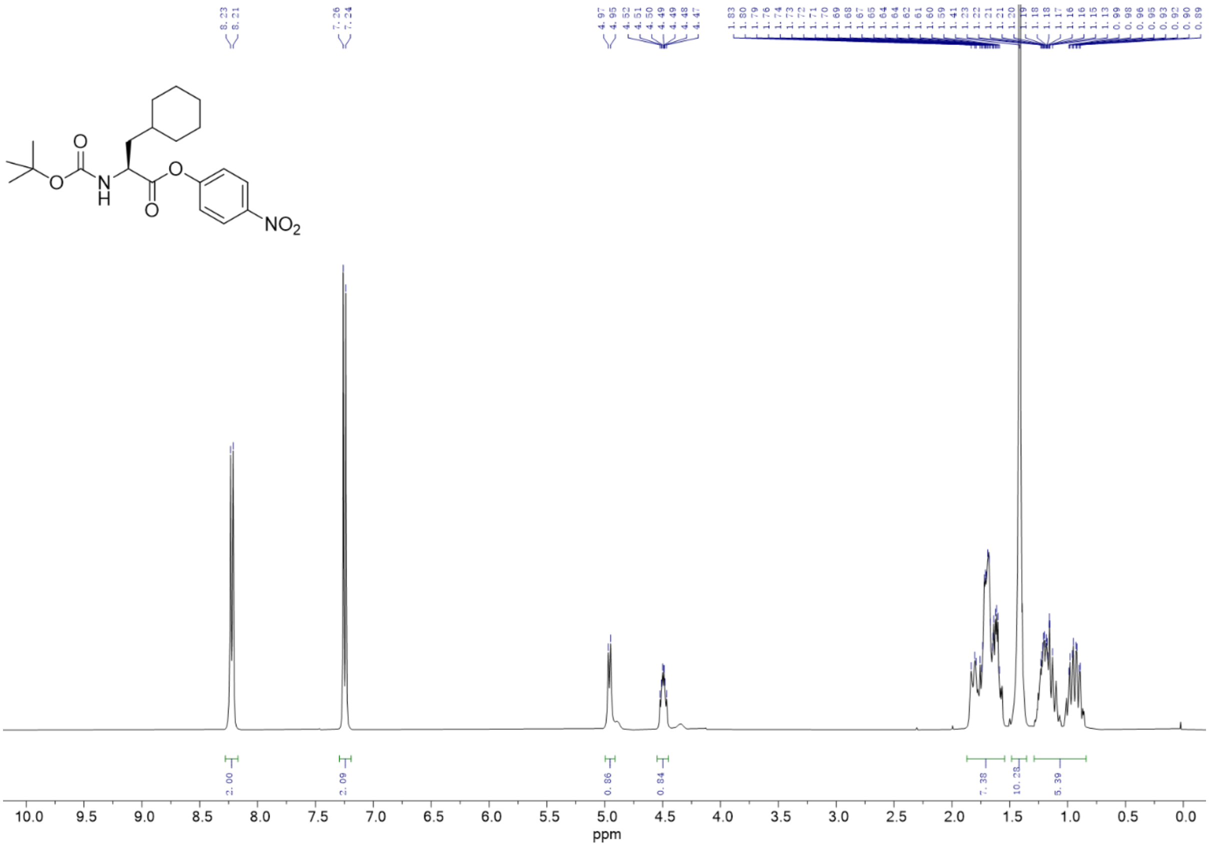

Spectrum S11: ^{1}H NMR (400 MHz, $CDCl_3$) spectrum of **1e**.

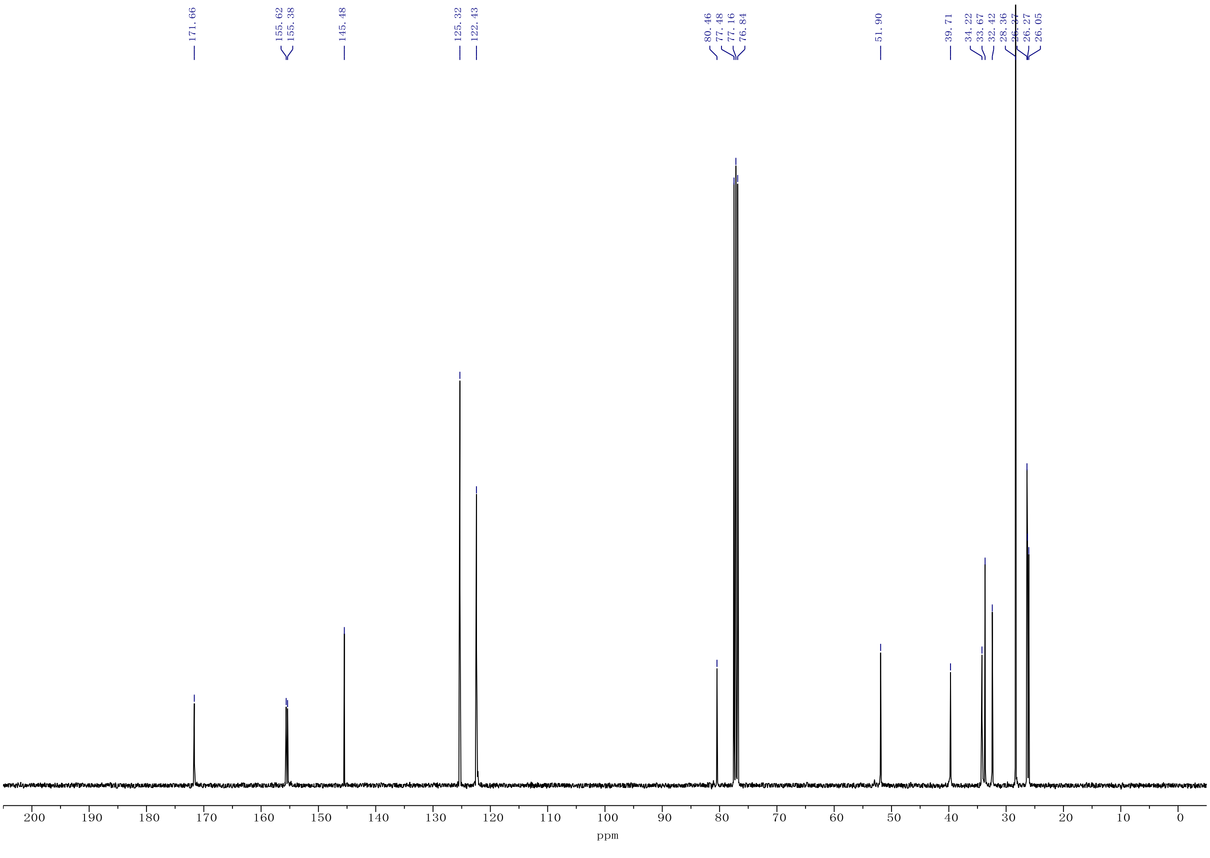

Spectrum S12: ^{13}C NMR (101 MHz, $CDCl_3$) spectrum of **1e**.

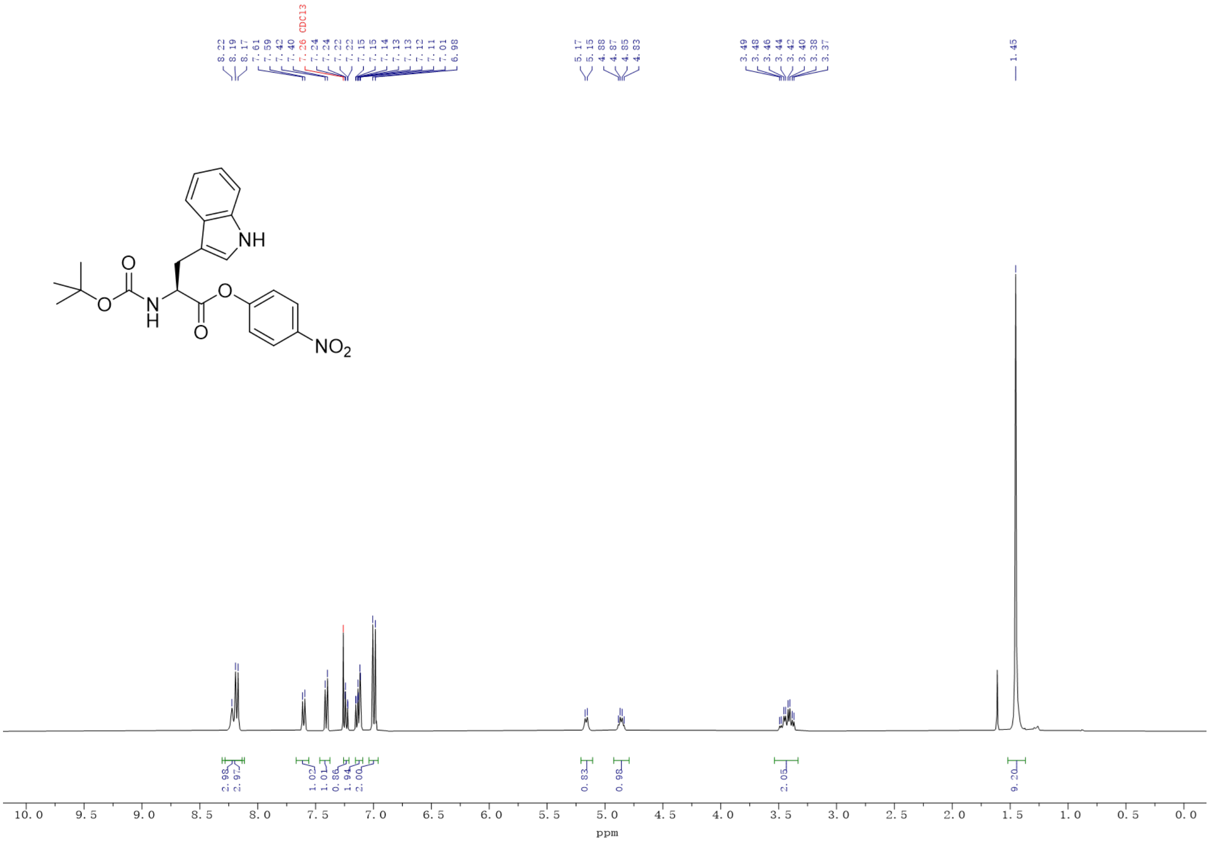

Spectrum S13: ^{1}H NMR (400 MHz, $CDCl_3$) spectrum of **1f**.

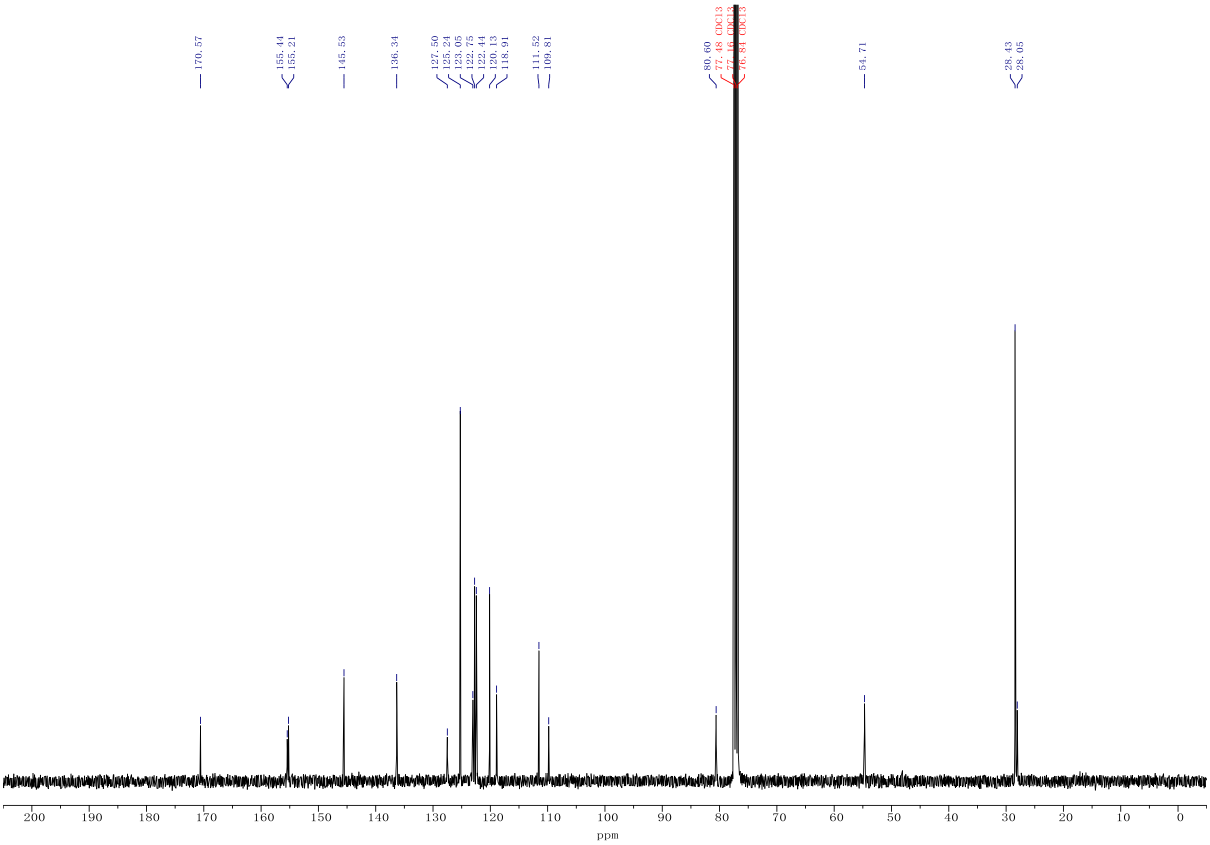

Spectrum S14: ^{13}C NMR (101 MHz, $CDCl_3$) spectrum of **1f**.

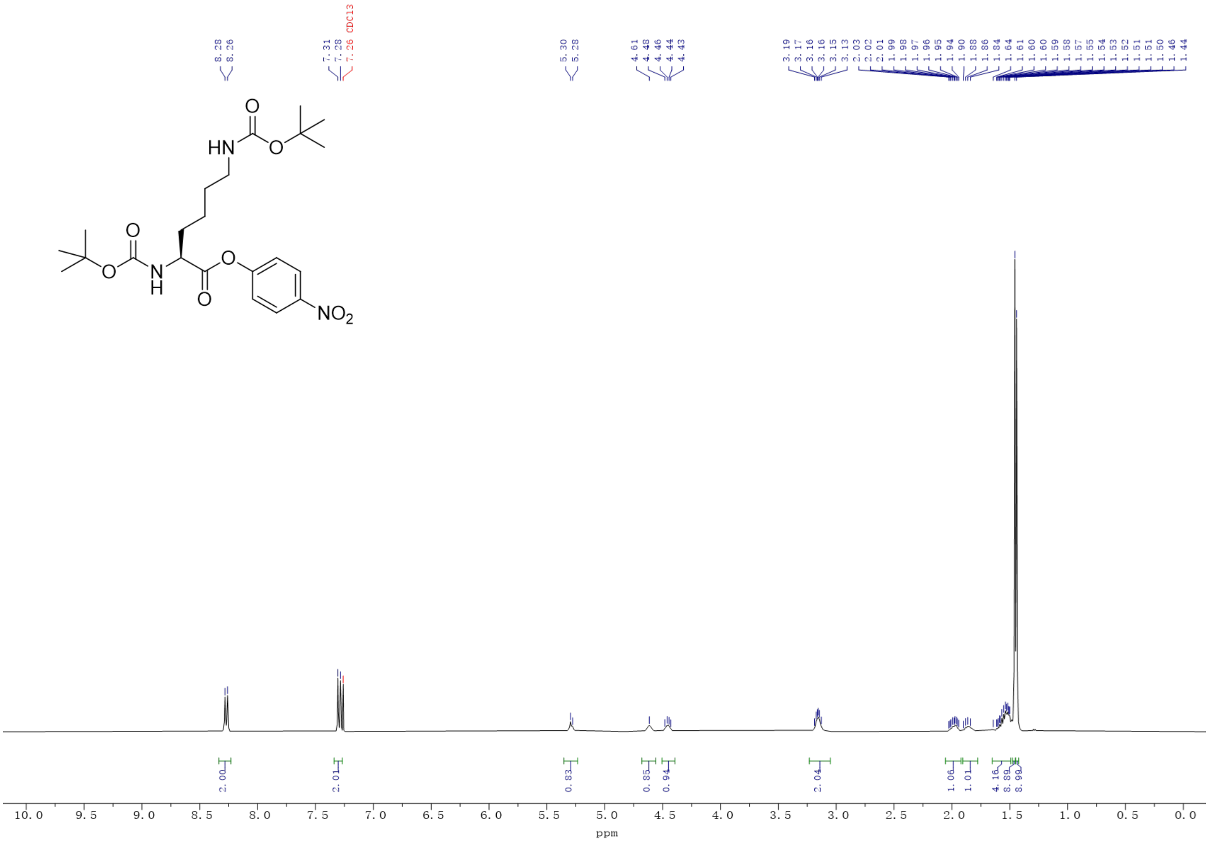

Spectrum S15: ^{1}H NMR (400 MHz, $CDCl_3$) spectrum of **1g**.

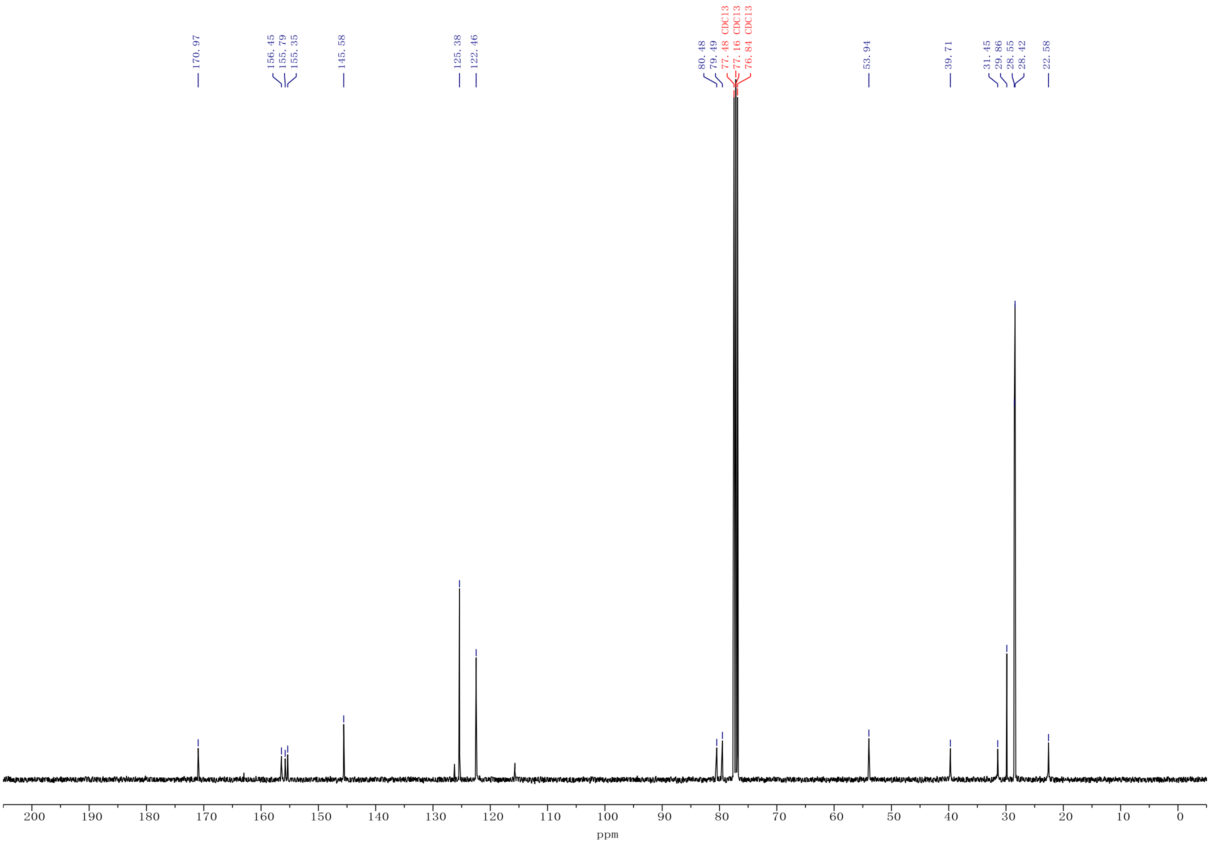

Spectrum S16: ^{13}C NMR (101 MHz, $CDCl_3$) spectrum of **1g**.

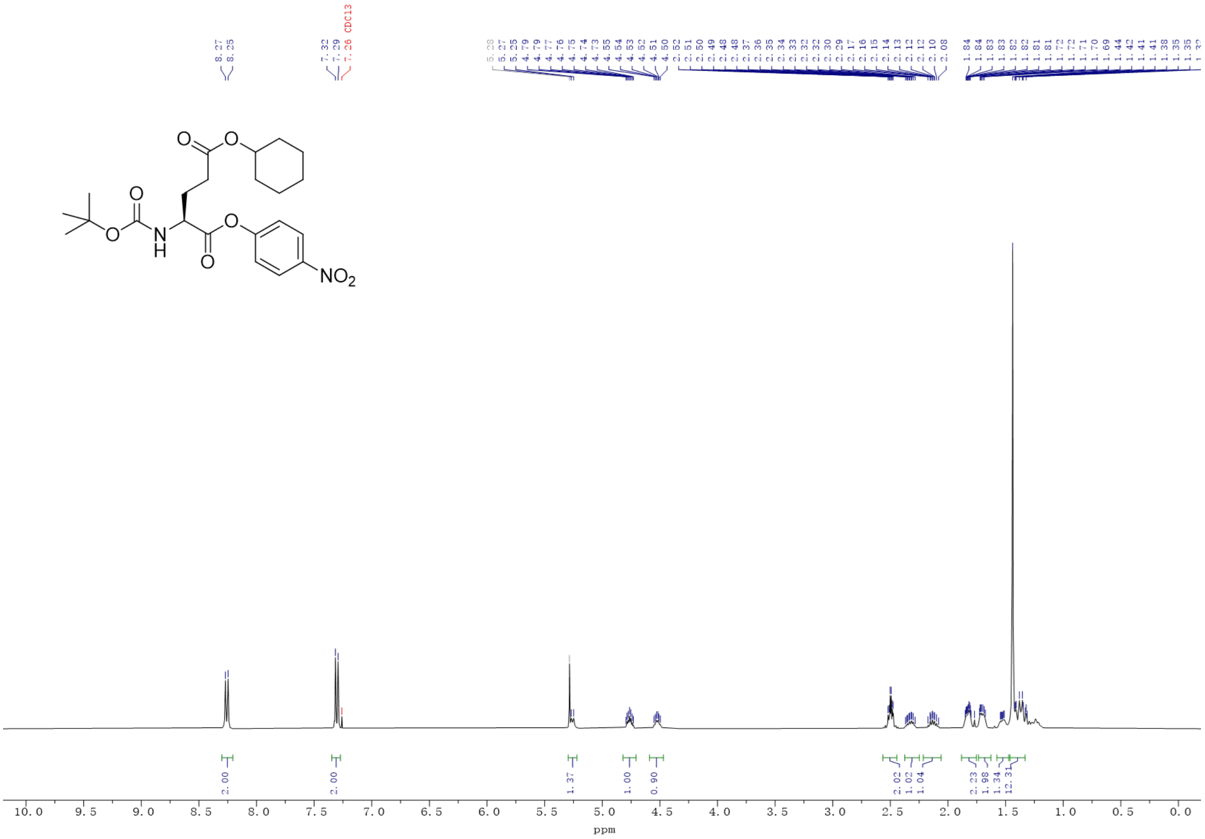

Spectrum S17: ^{1}H NMR (400 MHz, $CDCl_3$) spectrum of **1h**.

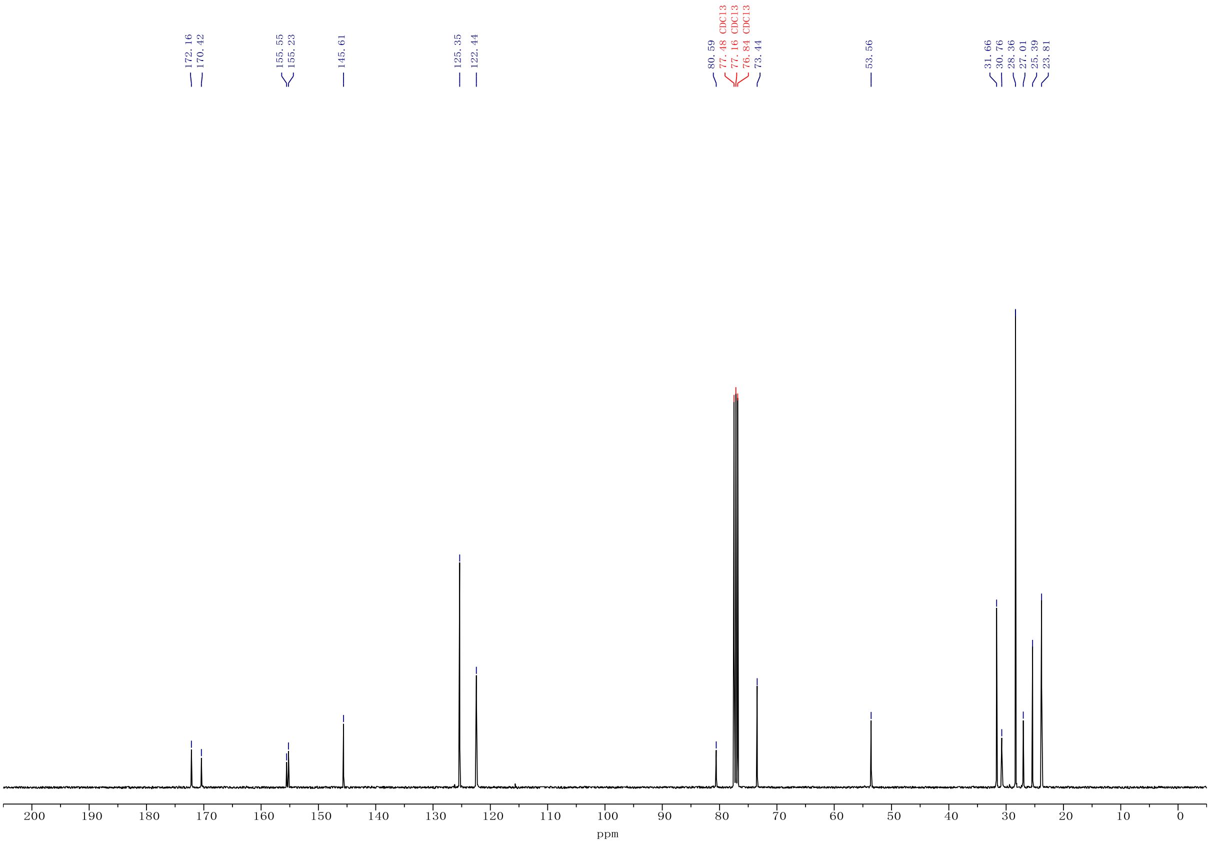

Spectrum S18: ^{13}C NMR (101 MHz, $CDCl_3$) spectrum of **1h**.

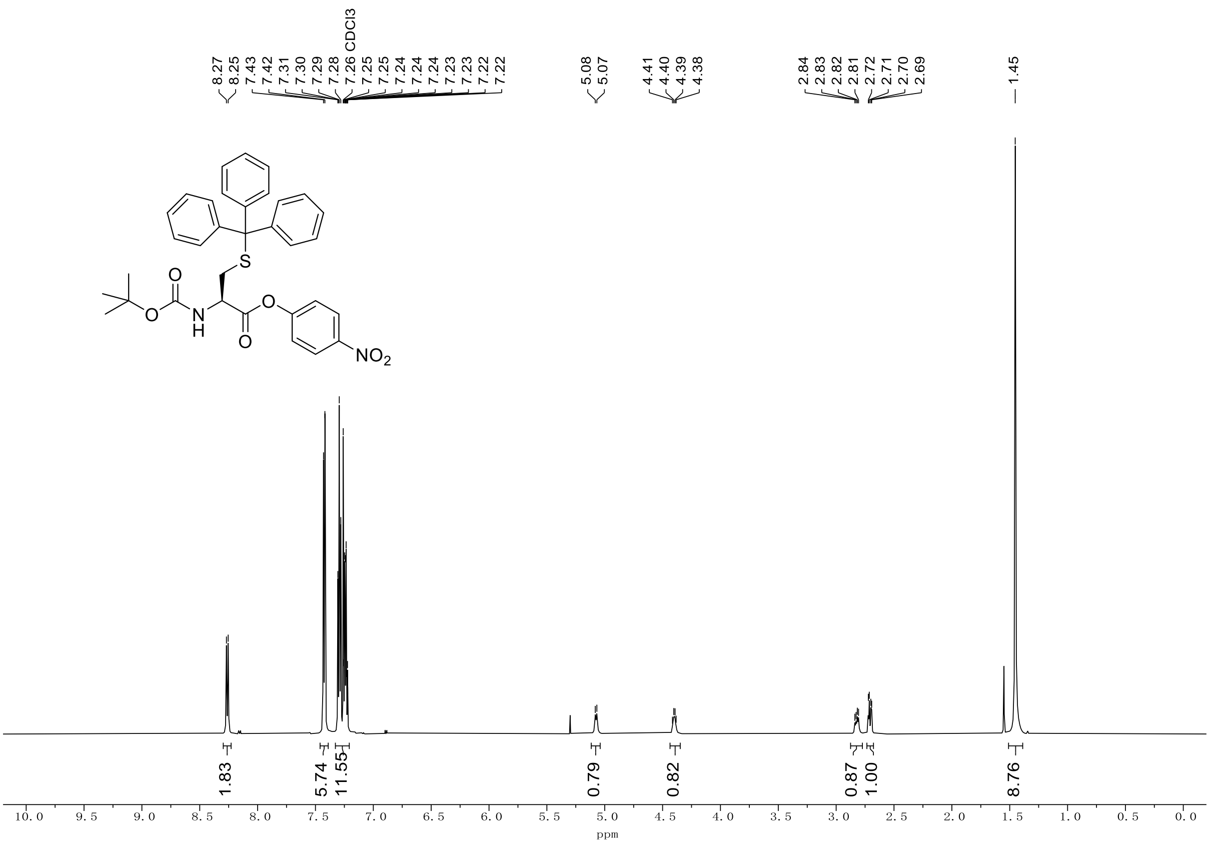

Spectrum S19: ^{1}H NMR (600 MHz, $CDCl_3$) spectrum of **1i**.

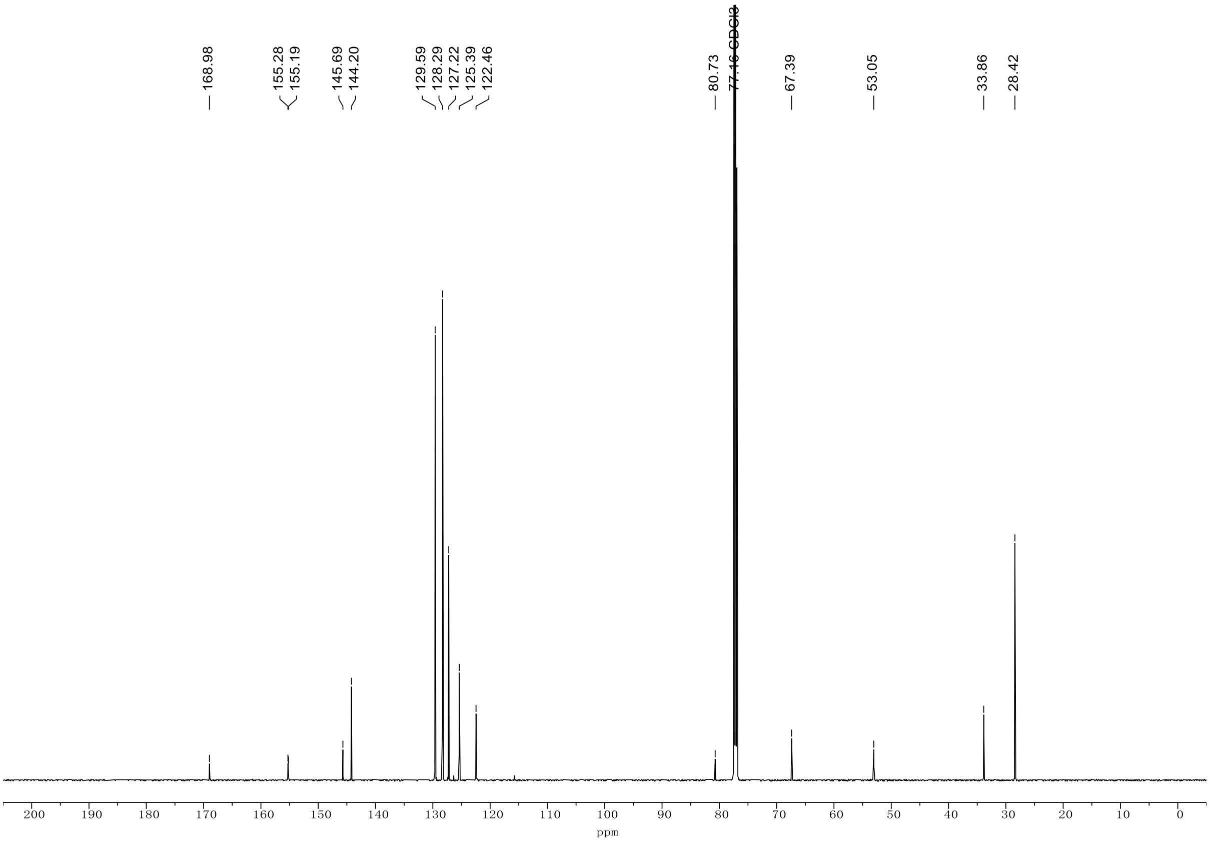

Spectrum S20: ^{13}C NMR (151 MHz, $CDCl_3$) spectrum of **1i**.

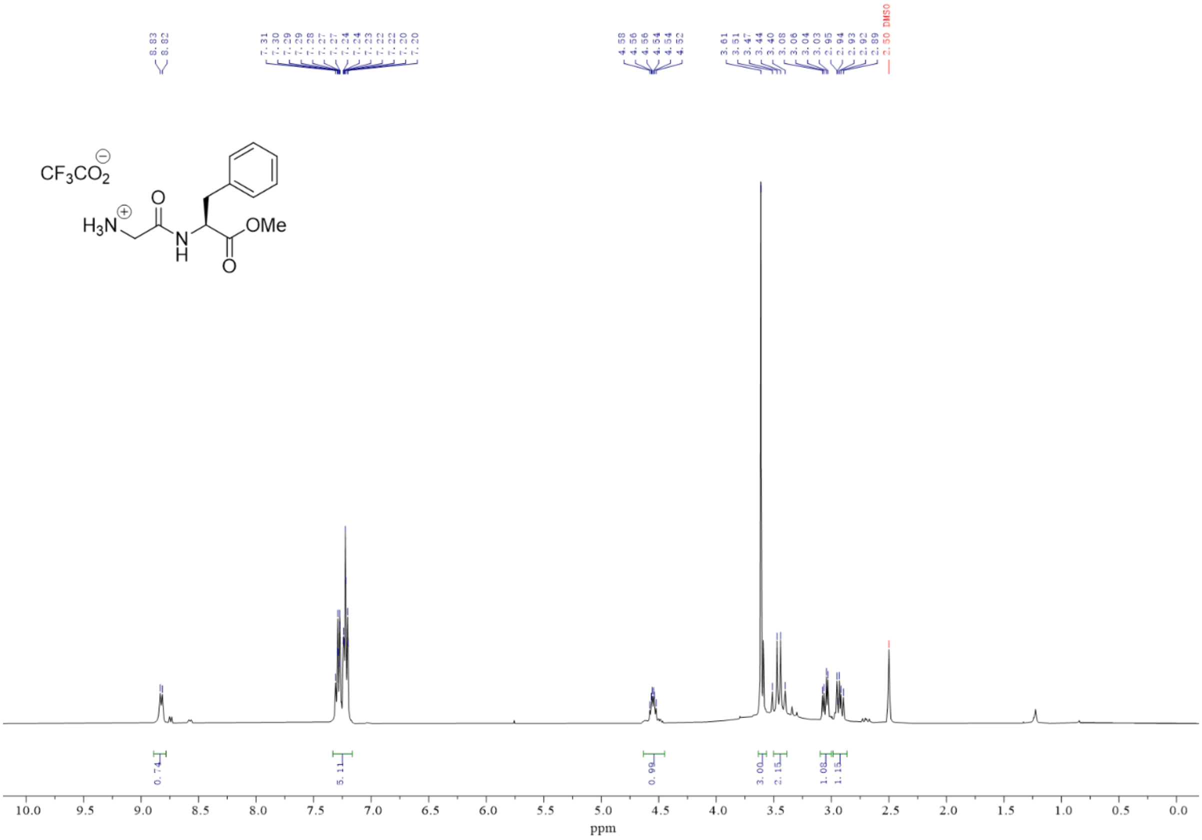

Spectrum S21: ^{1}H NMR (400 MHz, DMSO-*d*6) spectrum of **3a**·CF_3CO_2H.

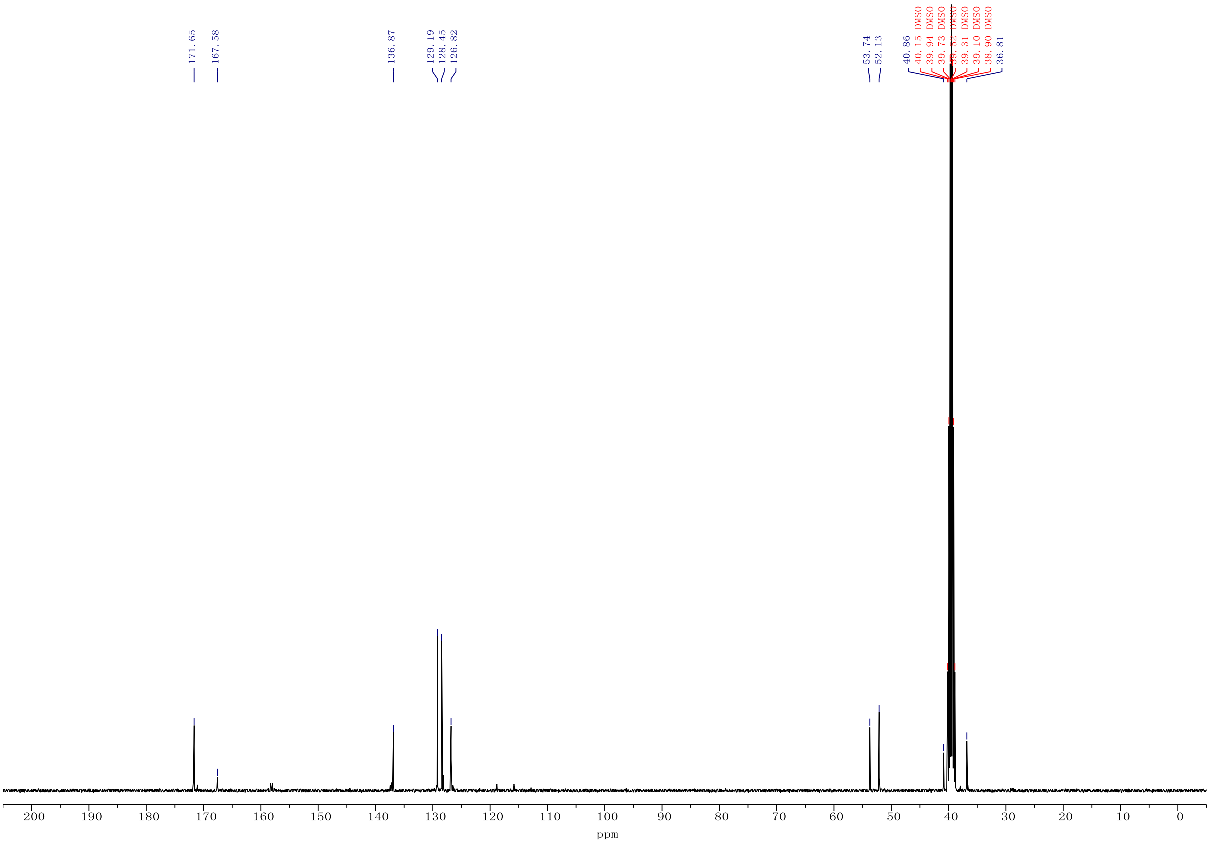

Spectrum S22: ^{13}C NMR (101 MHz, DMSO-*d*6) spectrum of **3a**·CF_3CO_2H.

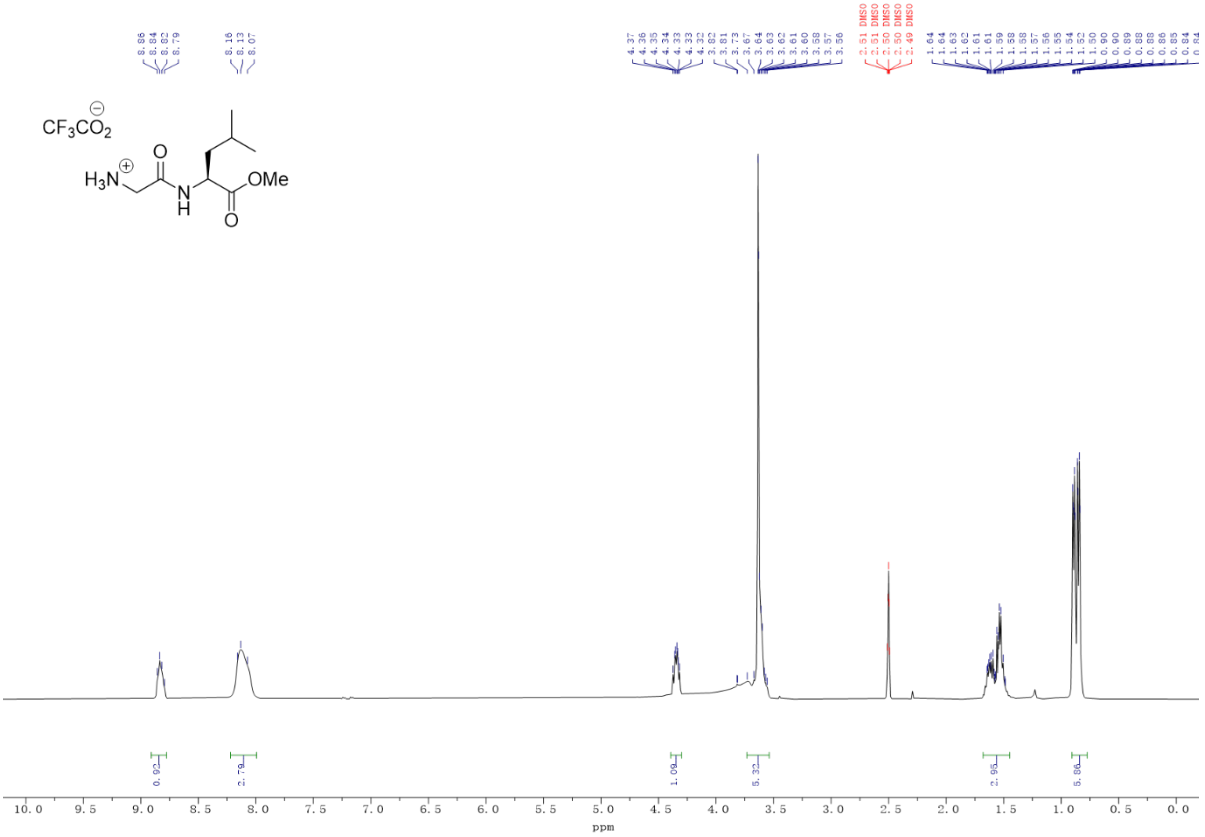

Spectrum S23: ^{1}H NMR (400 MHz, DMSO-d_6) spectrum of **3b**·CF_3CO_2H.

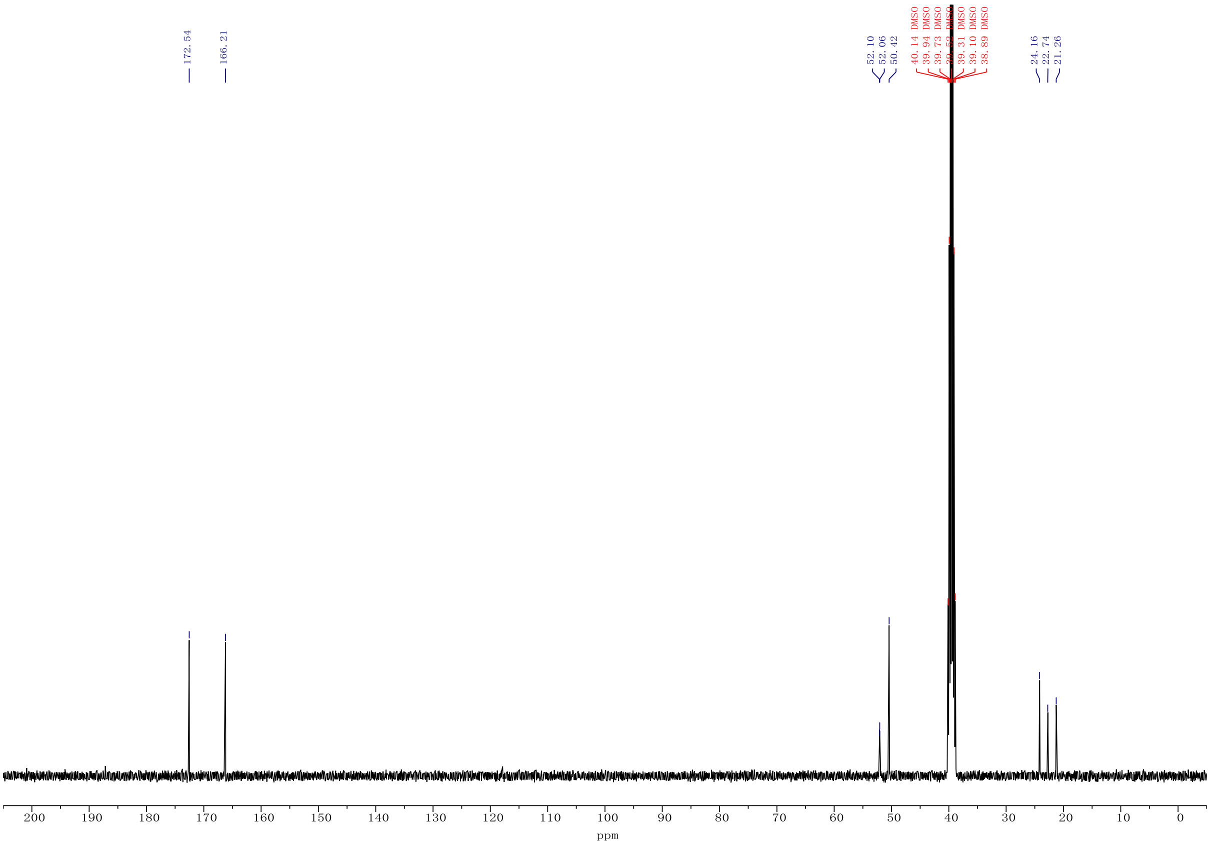

Spectrum S24: ^{13}C NMR (101 MHz, DMSO-d_6) spectrum of **3b**·CF_3CO_2H.

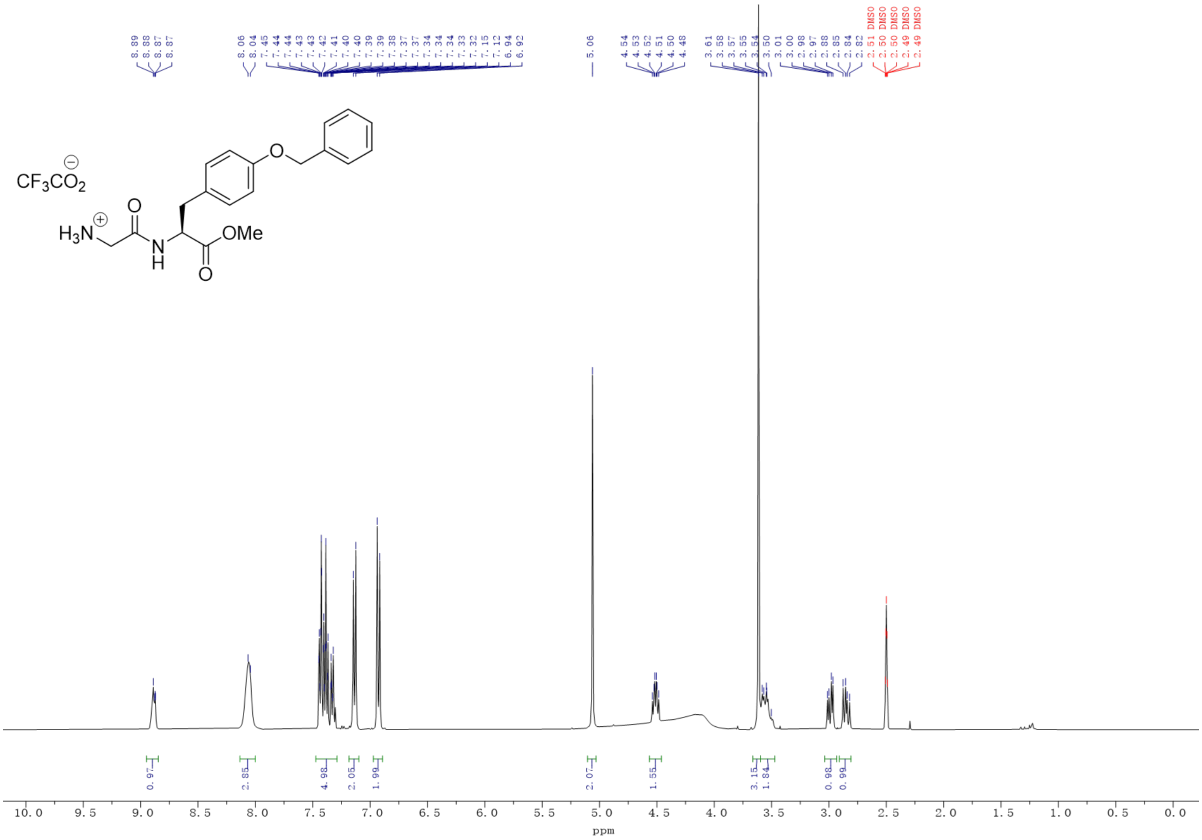

Spectrum S25: ^{1}H NMR (400 MHz, DMSO-*d6*) spectrum of **3c**·CF_3CO_2H.

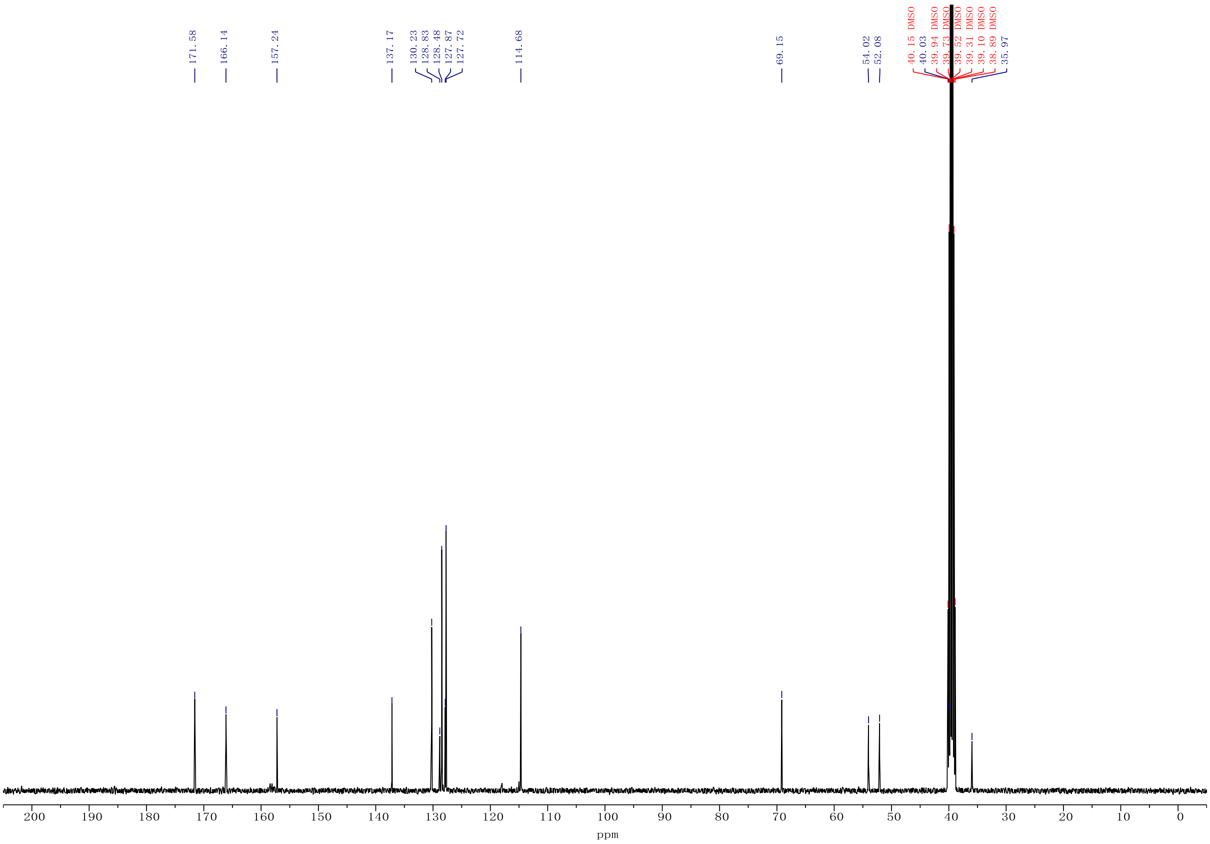

Spectrum S26: ^{13}C NMR (101 MHz, DMSO-*d6*) spectrum of **3c**·CF_3CO_2H.

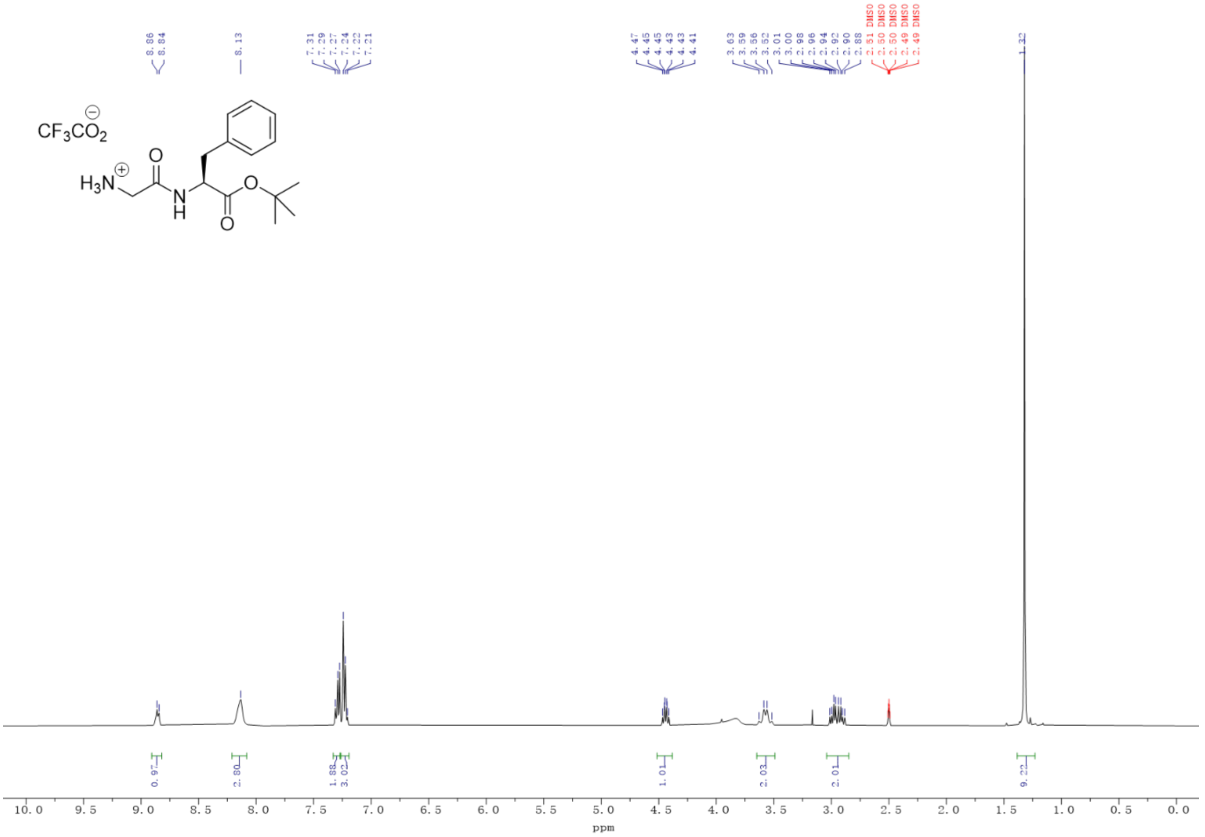

Spectrum S27: ^{1}H NMR (400 MHz, DMSO-d_6) spectrum of **3d**·CF_3CO_2H.

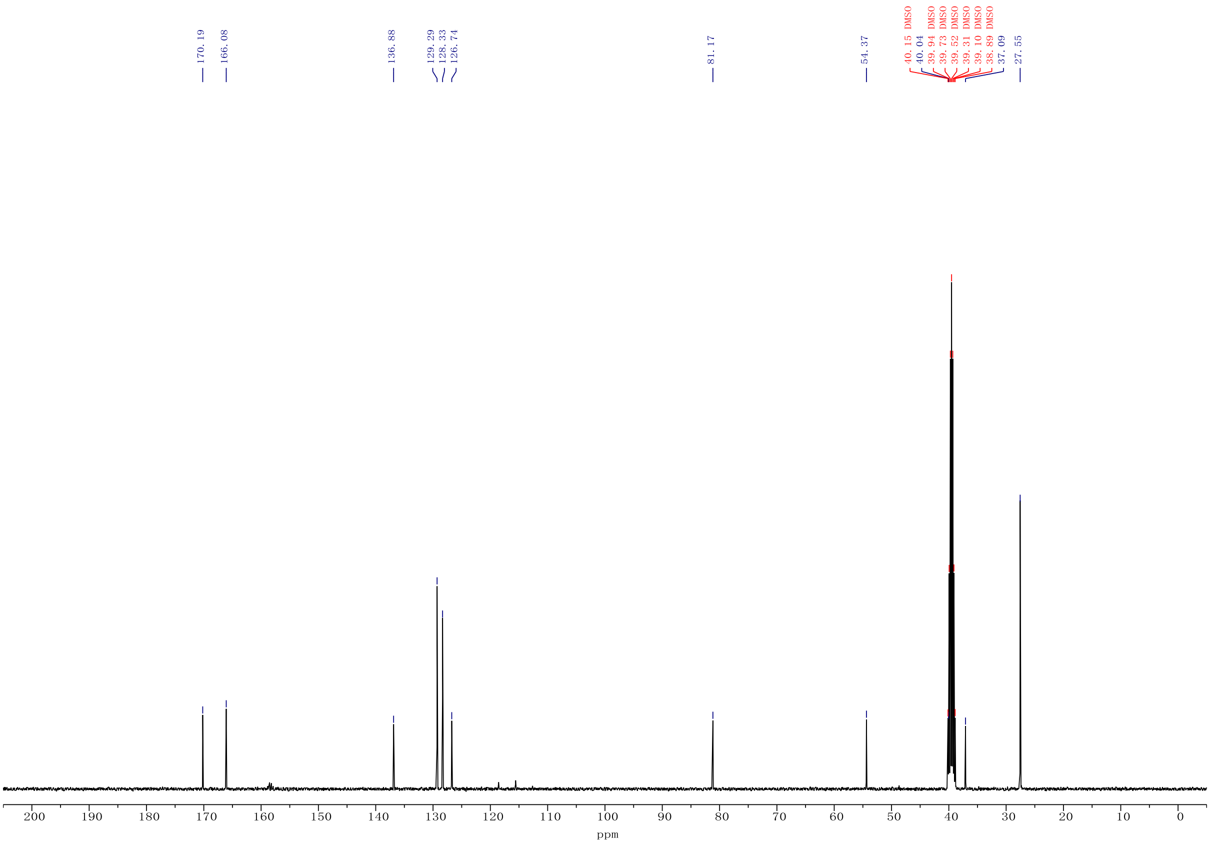

Spectrum S28: ^{13}C NMR (101 MHz, DMSO-d_6) spectrum of **3d**·CF_3CO_2H.

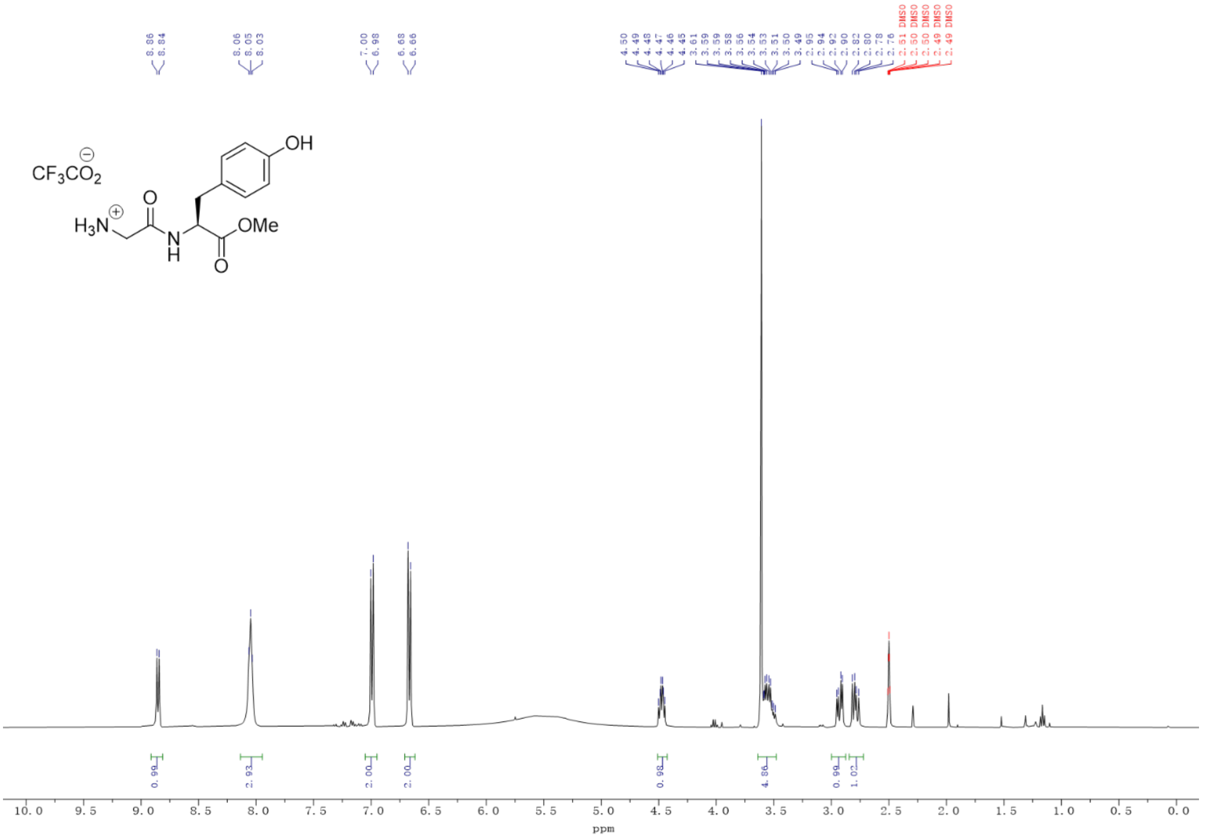

Spectrum S29: ^{1}H NMR (400 MHz, DMSO-d_6) spectrum of **3e**·CF_3CO_2H.

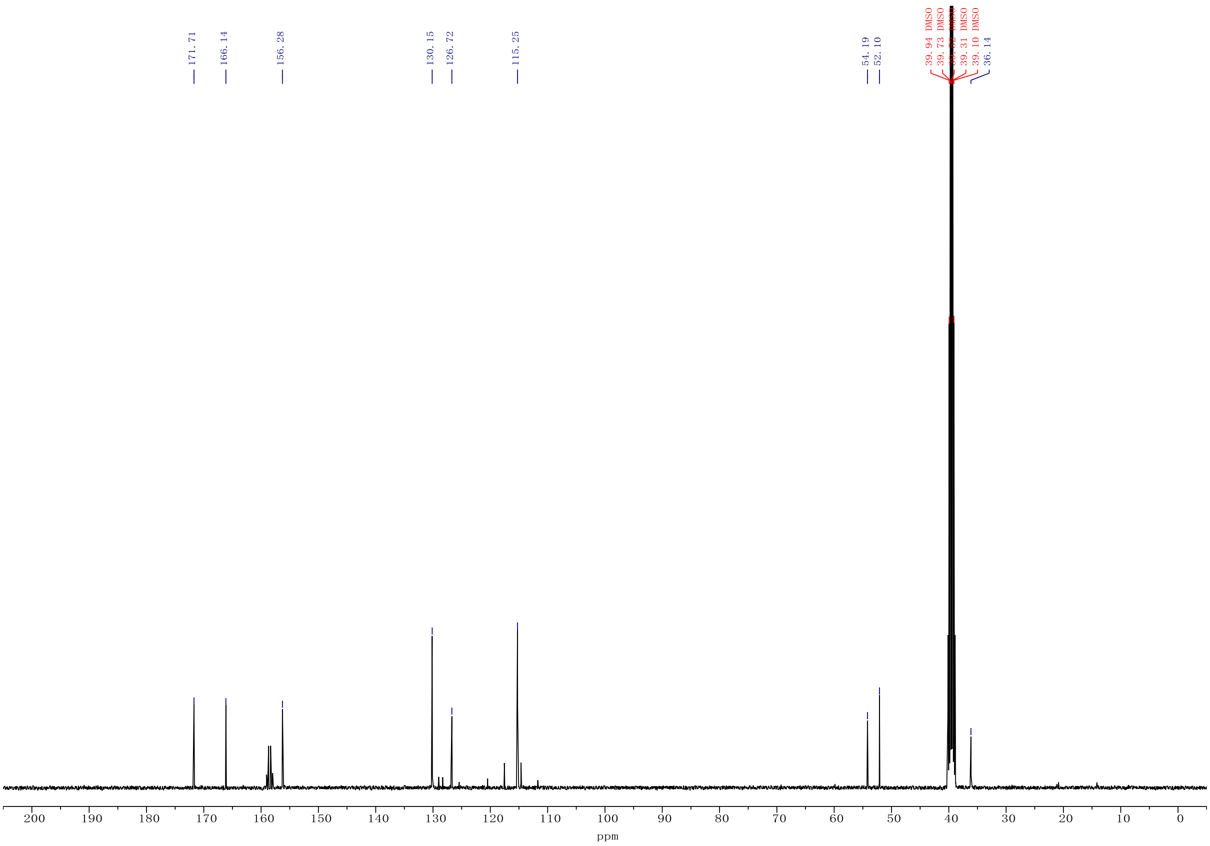

Spectrum S30: ^{13}C NMR (101 MHz, DMSO-d_6) spectrum of **3e**·CF_3CO_2H.

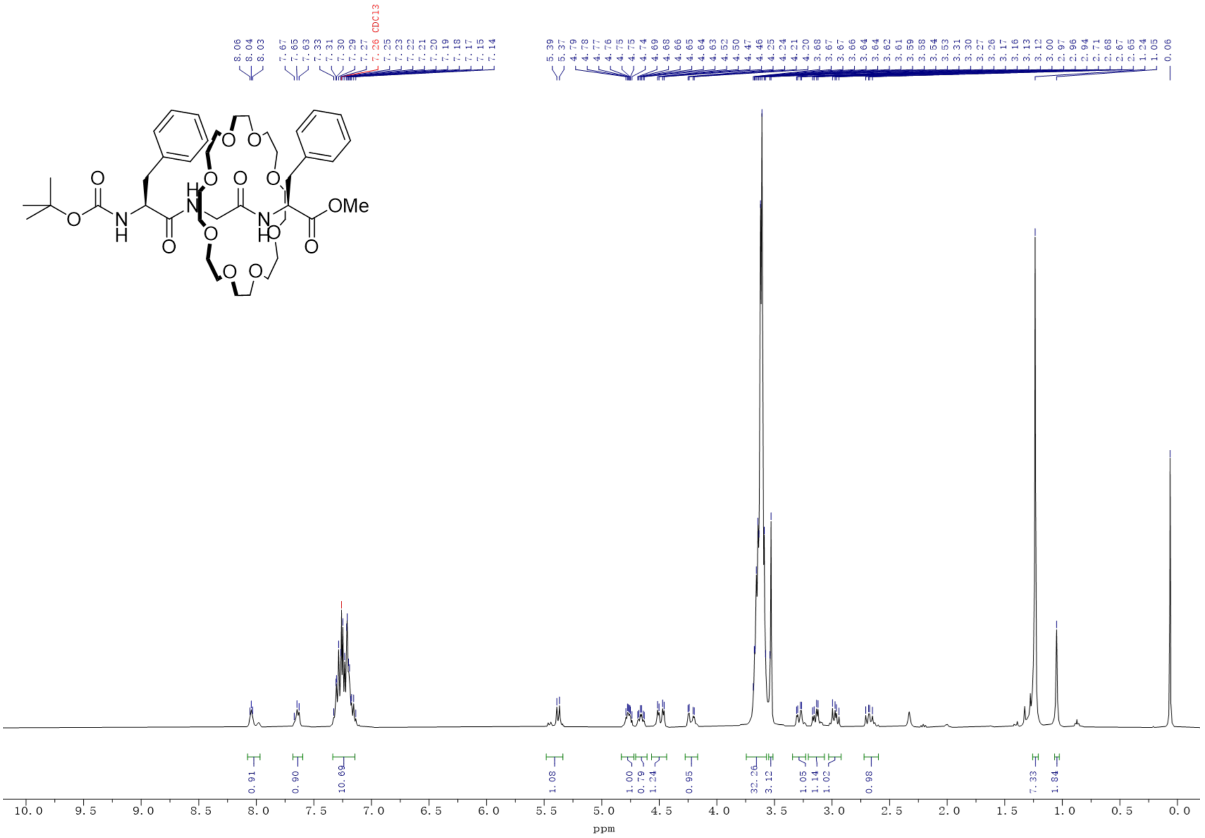

Spectrum S31: ^{1}H NMR (400 MHz, $CDCl_3$) spectrum of **24C8⊂4**.

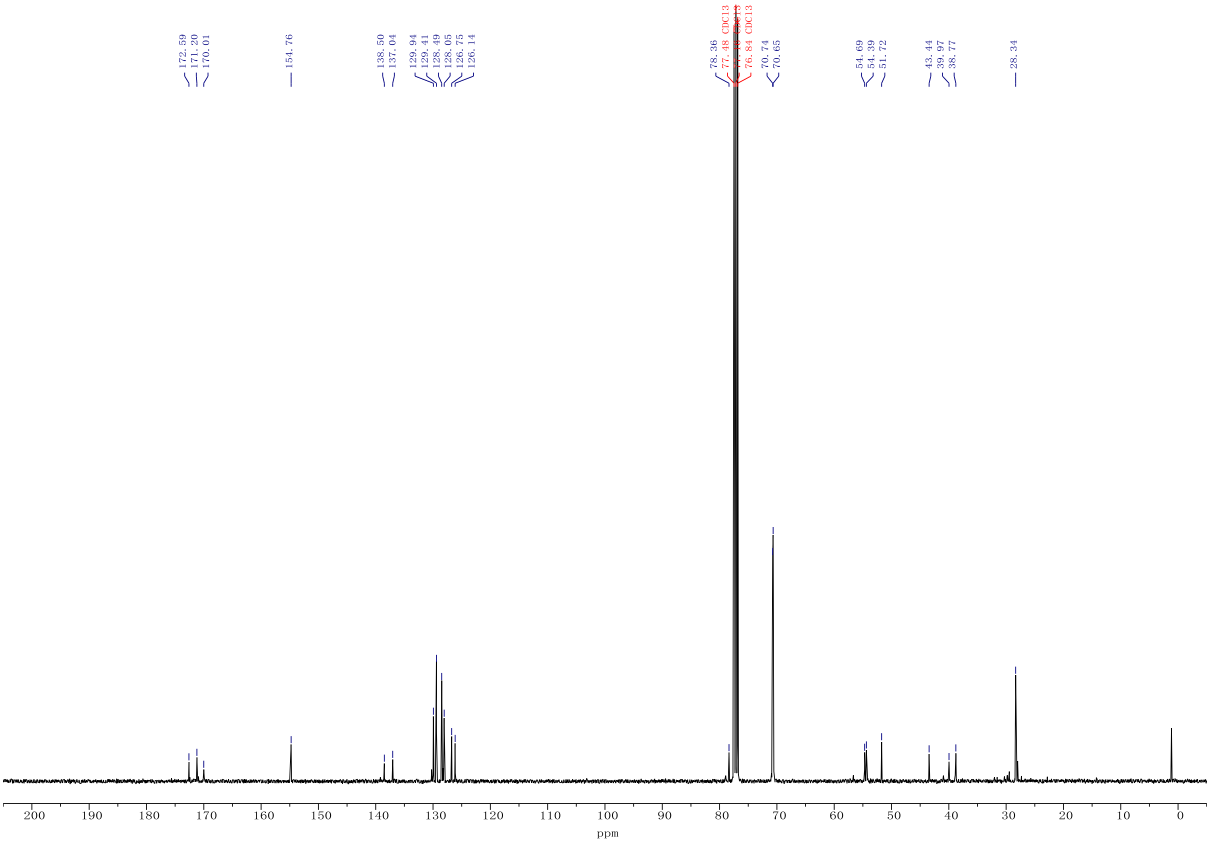

Spectrum S32: ^{13}C NMR (101 MHz, $CDCl_3$) spectrum of **24C8⊂4**.

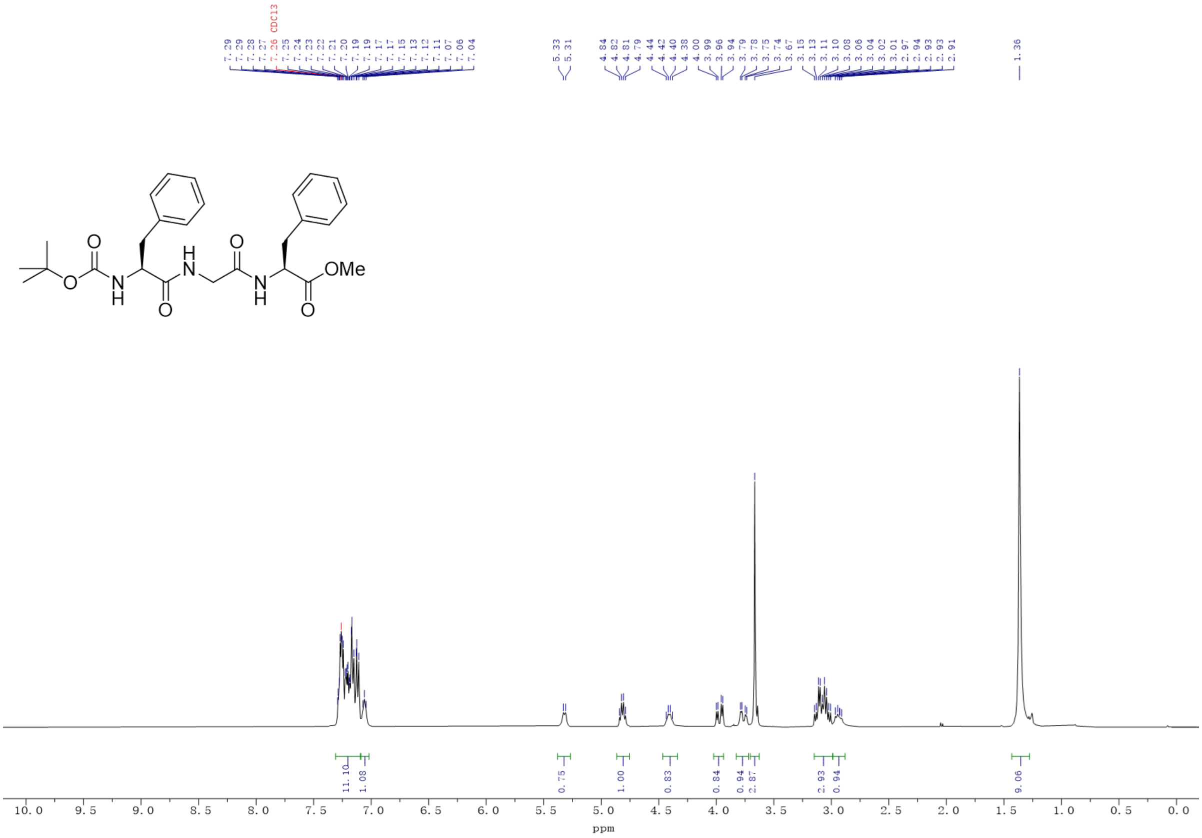

Spectrum S33: ^{1}H NMR (400 MHz, $CDCl_3$) spectrum of **4**.

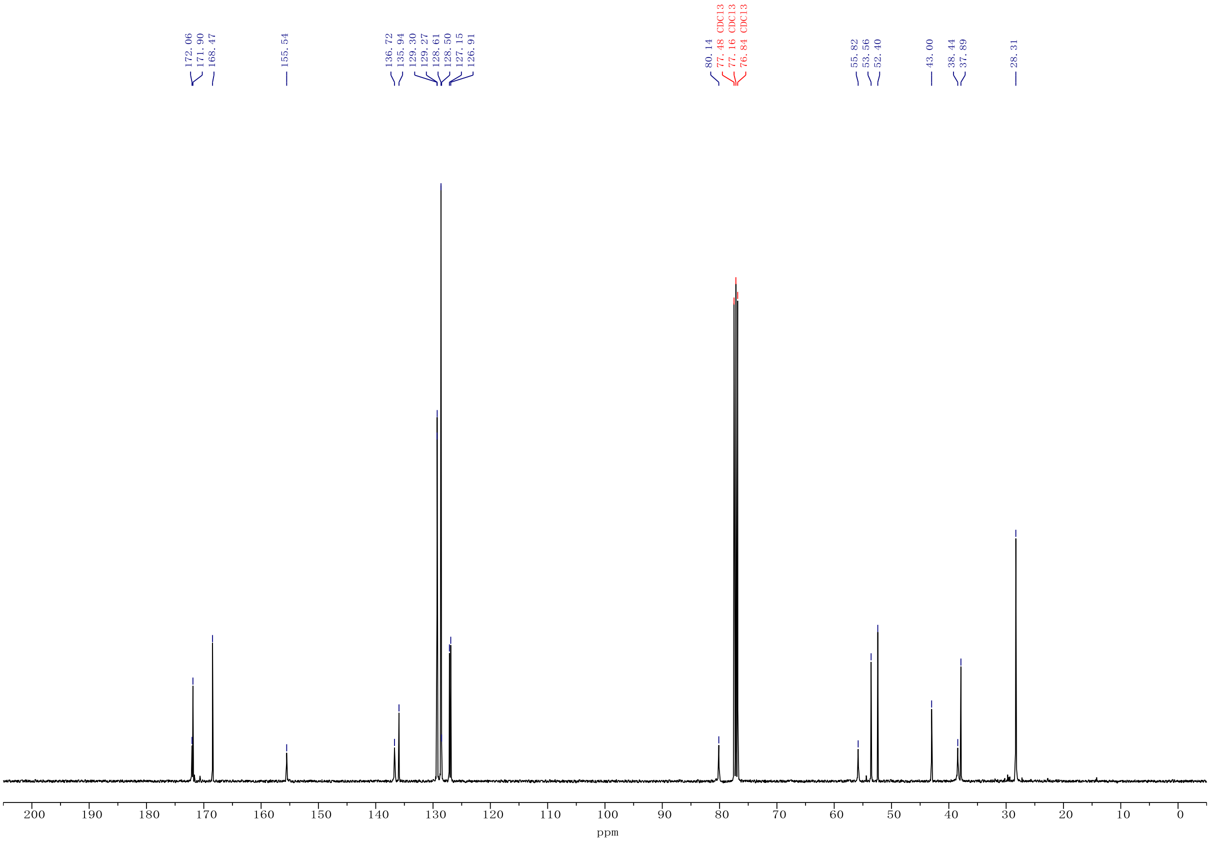

Spectrum S34: ^{13}C NMR (101 MHz, $CDCl_3$) spectrum of **4**.

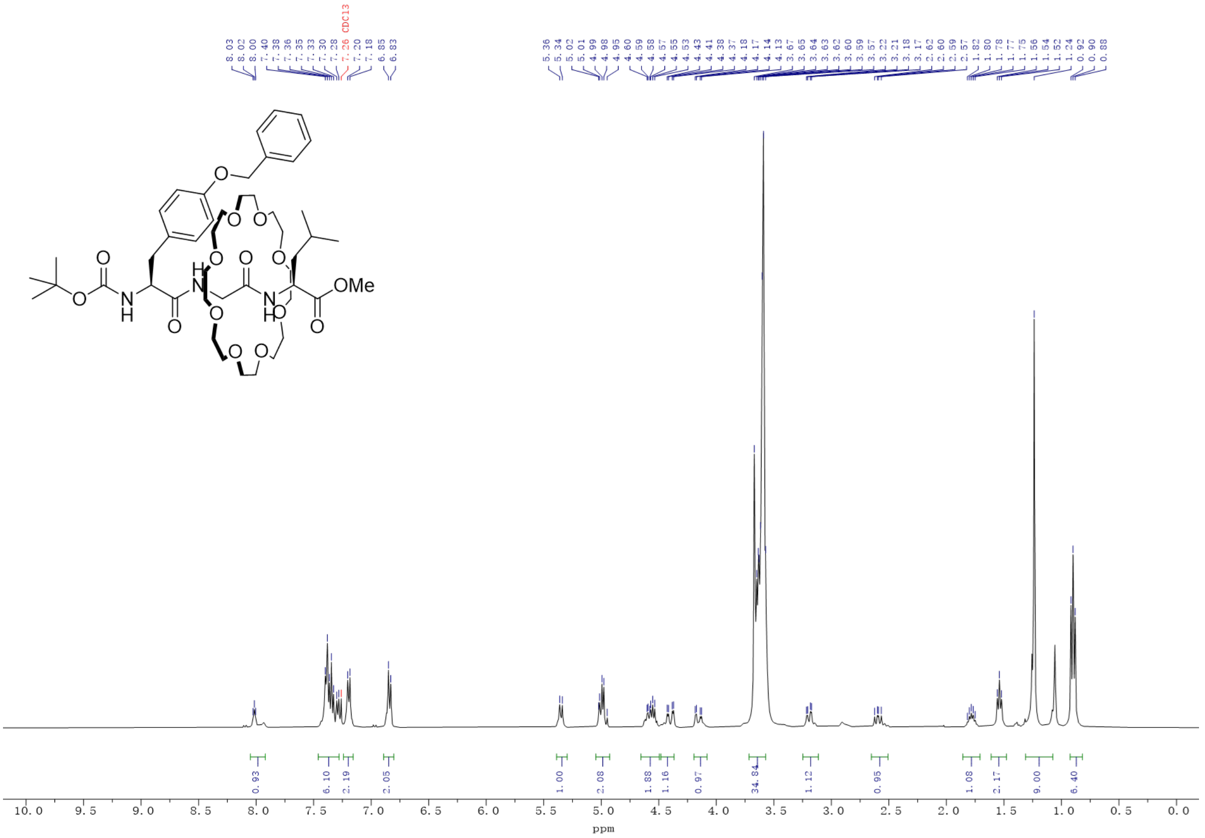

Spectrum S35: ^{1}H NMR (400 MHz, $CDCl_3$) spectrum of **24C8⊂5**.

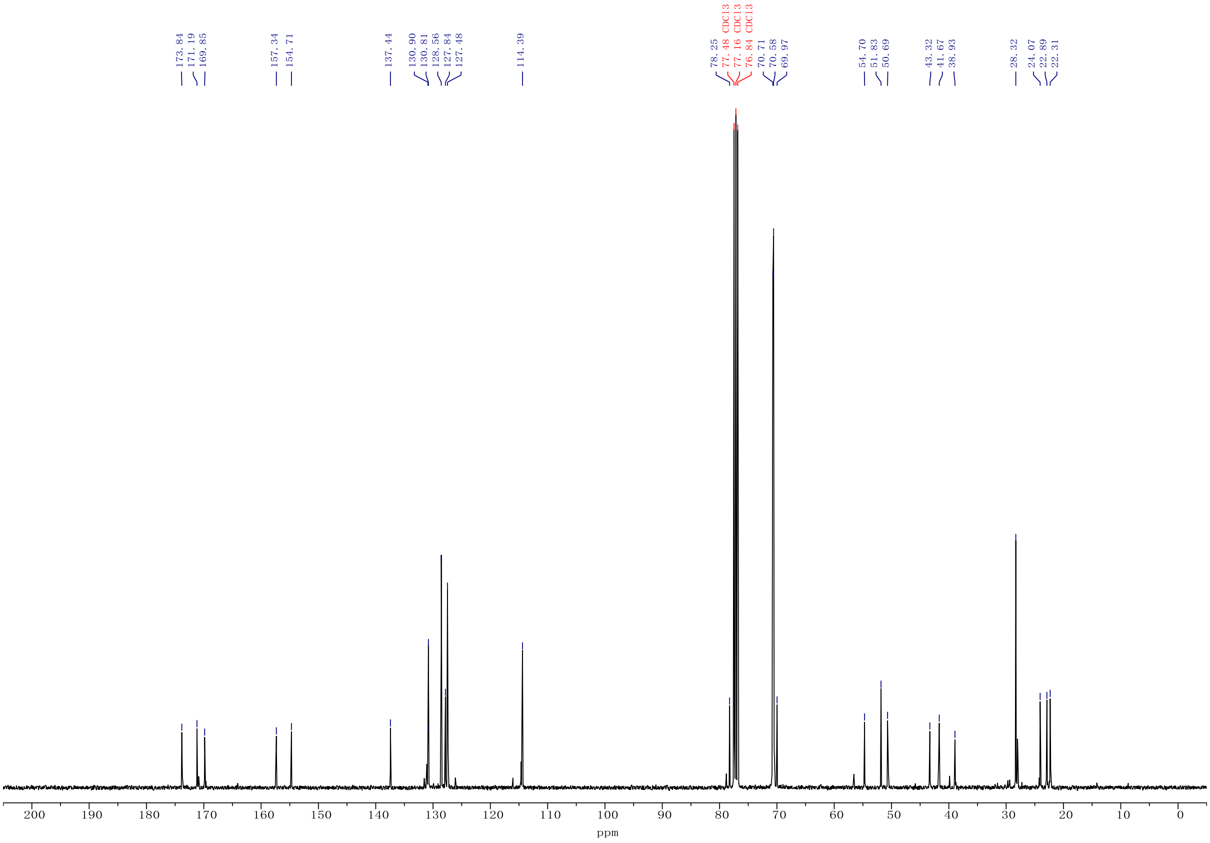

Spectrum S36: ^{13}C NMR (101 MHz, $CDCl_3$) spectrum of **24C8⊂5**.

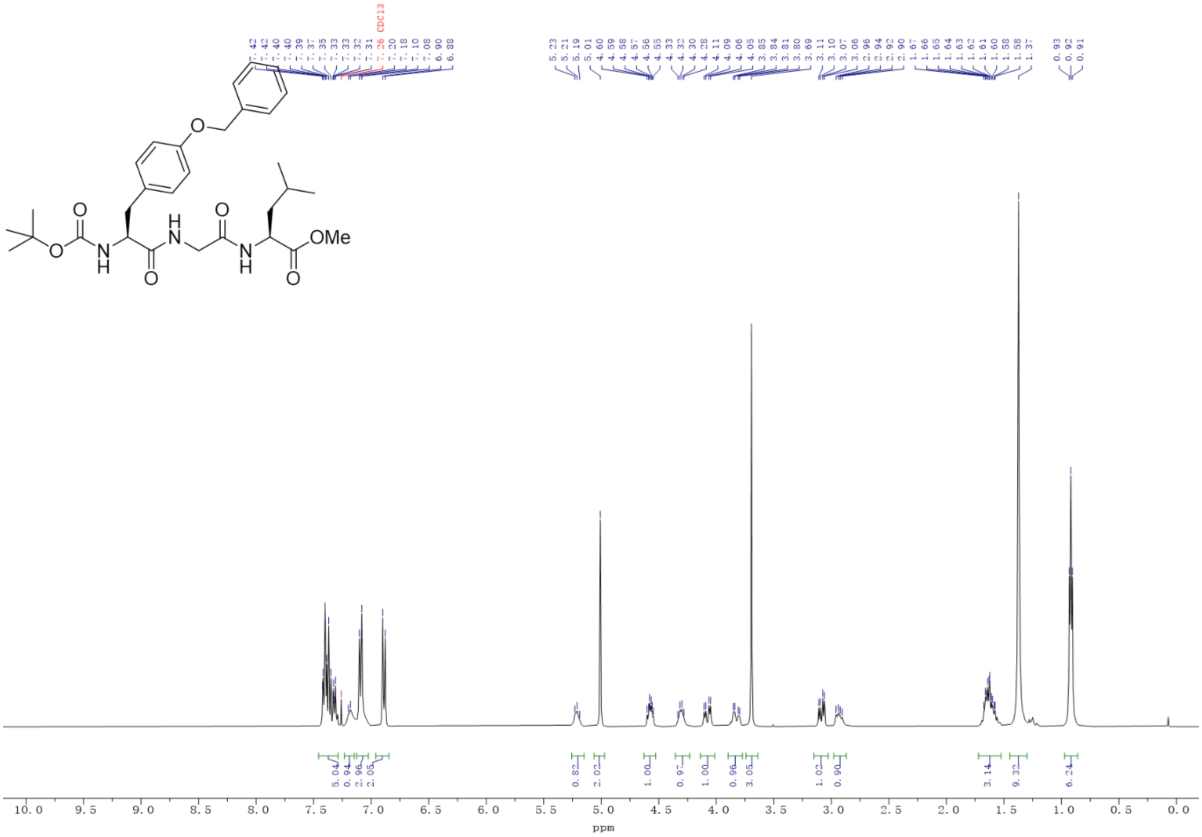

Spectrum S37: ^{1}H NMR (400 MHz, $CDCl_3$) spectrum of **5**.

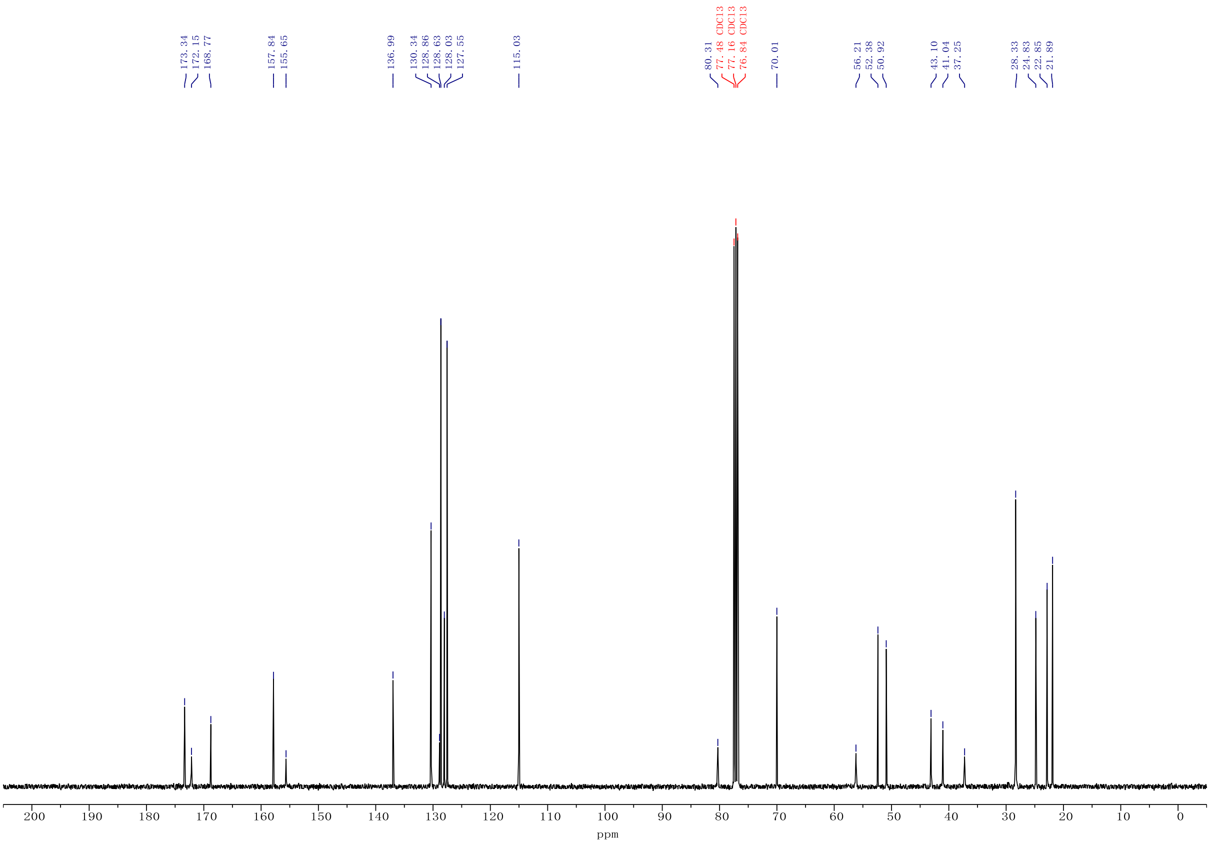

Spectrum S38: ^{13}C NMR (101 MHz, $CDCl_3$) spectrum of **5**.

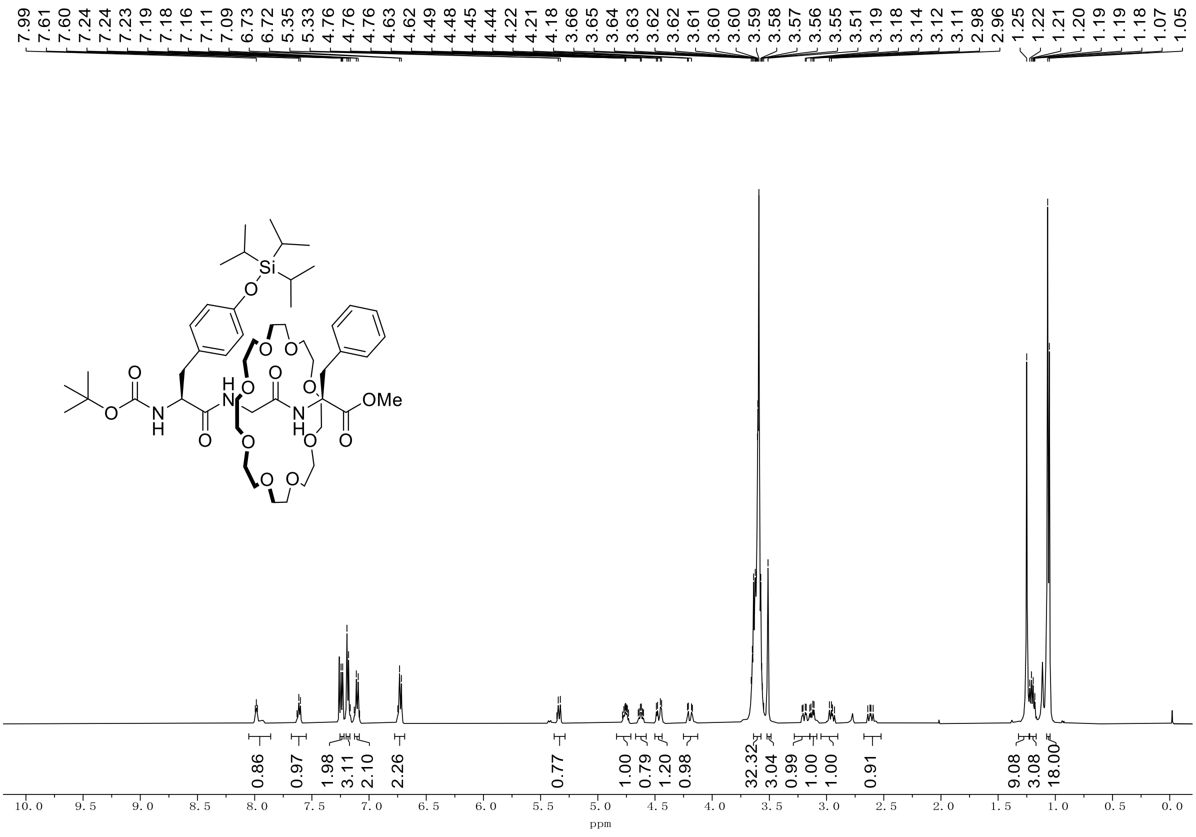

Spectrum S39: ^{1}H NMR (500 MHz, $CDCl_3$) spectrum of **24C8⊂6**.

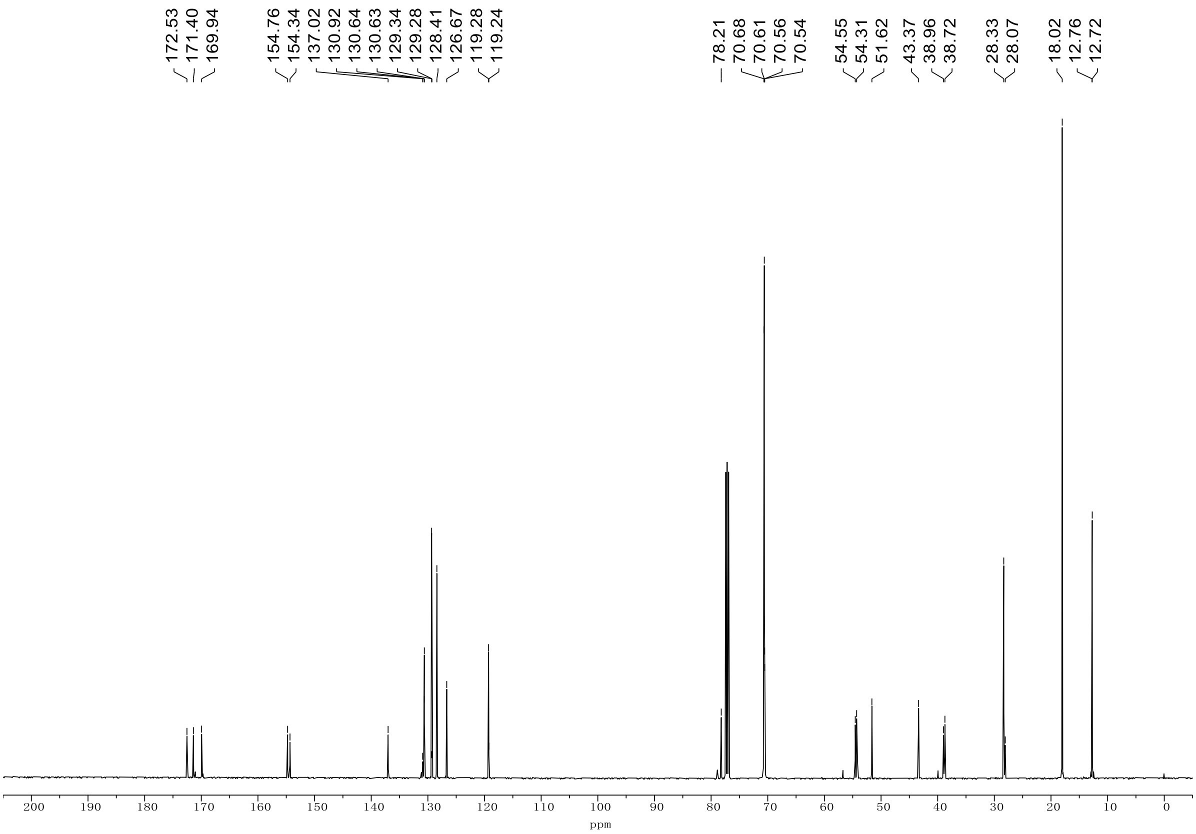

Spectrum S40: ^{13}C NMR (125 MHz, $CDCl_3$) spectrum of **24C8⊂6**.

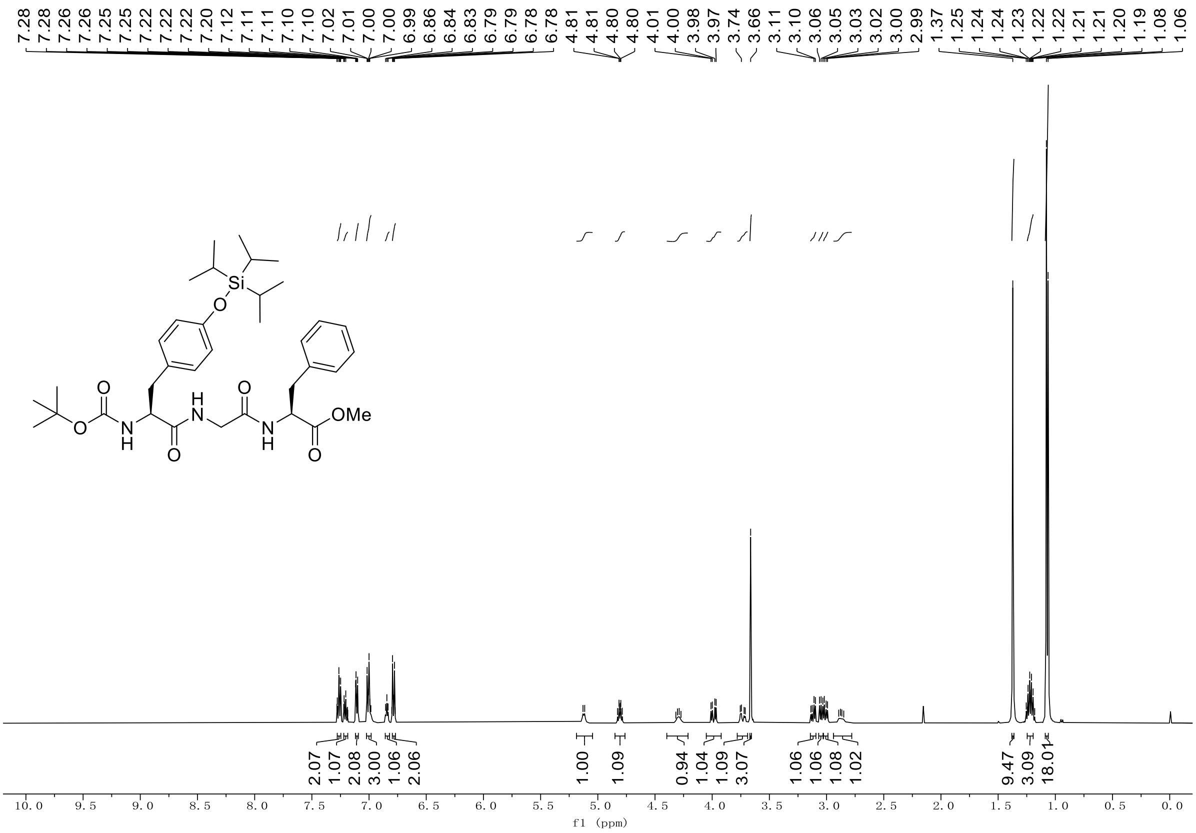

Spectrum S41: ^{1}H NMR (500 MHz, $CDCl_3$) spectrum of **6**.

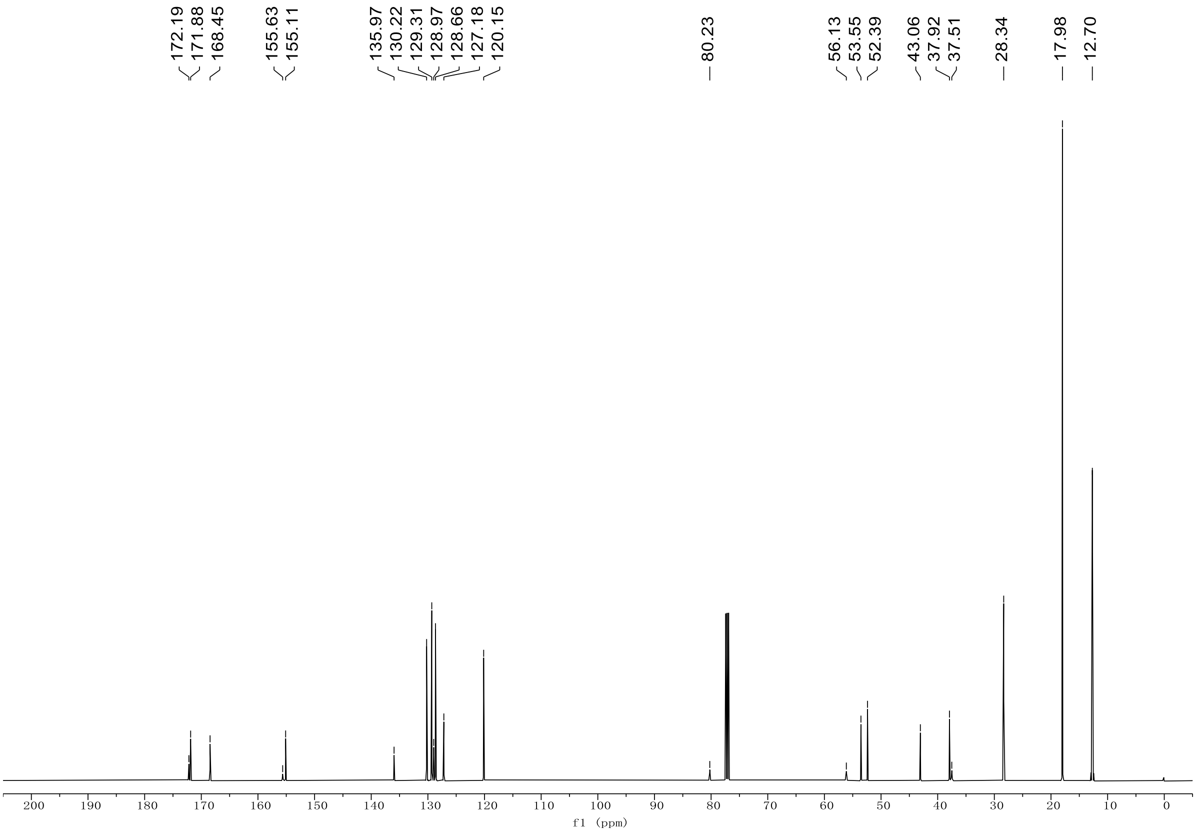

Spectrum S42: ^{13}C NMR (125 MHz, $CDCl_3$) spectrum of **6**.

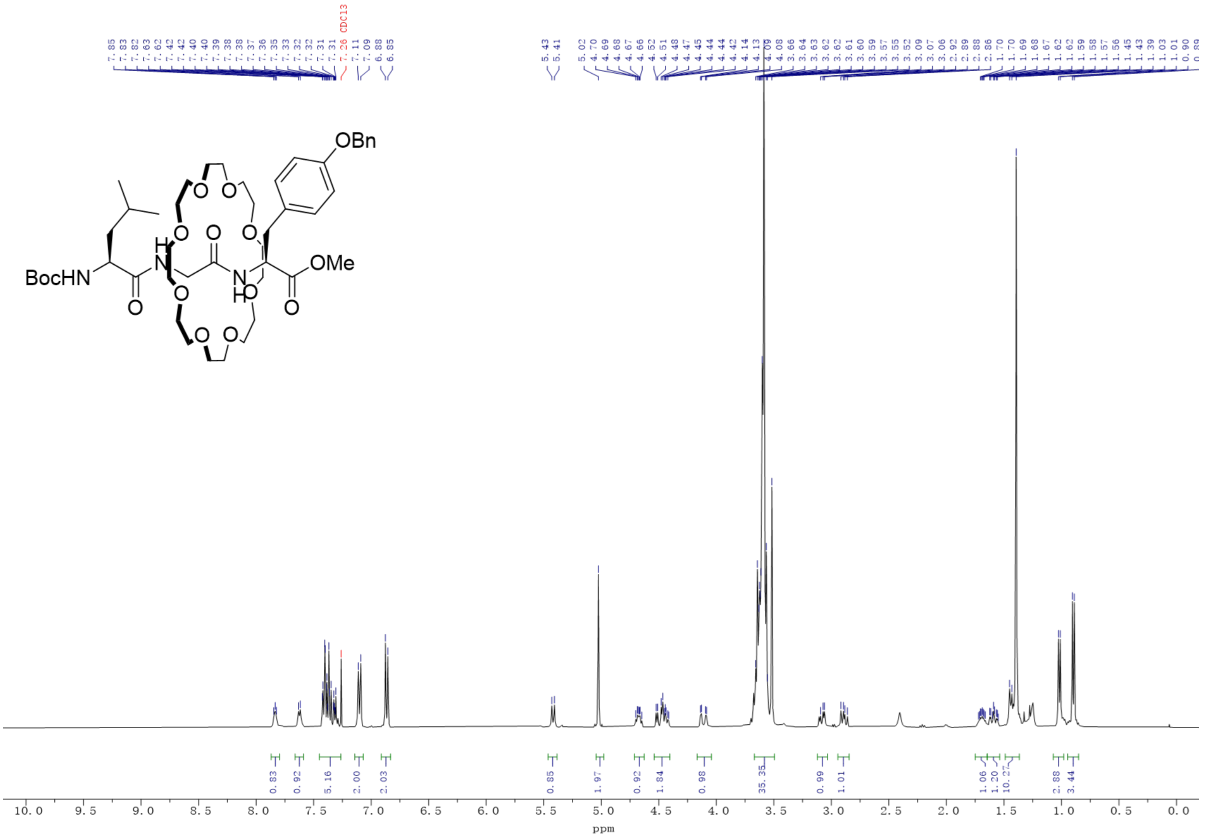

Spectrum S43: ^{1}H NMR (400 MHz, $CDCl_3$) spectrum of **24C8⊂7**.

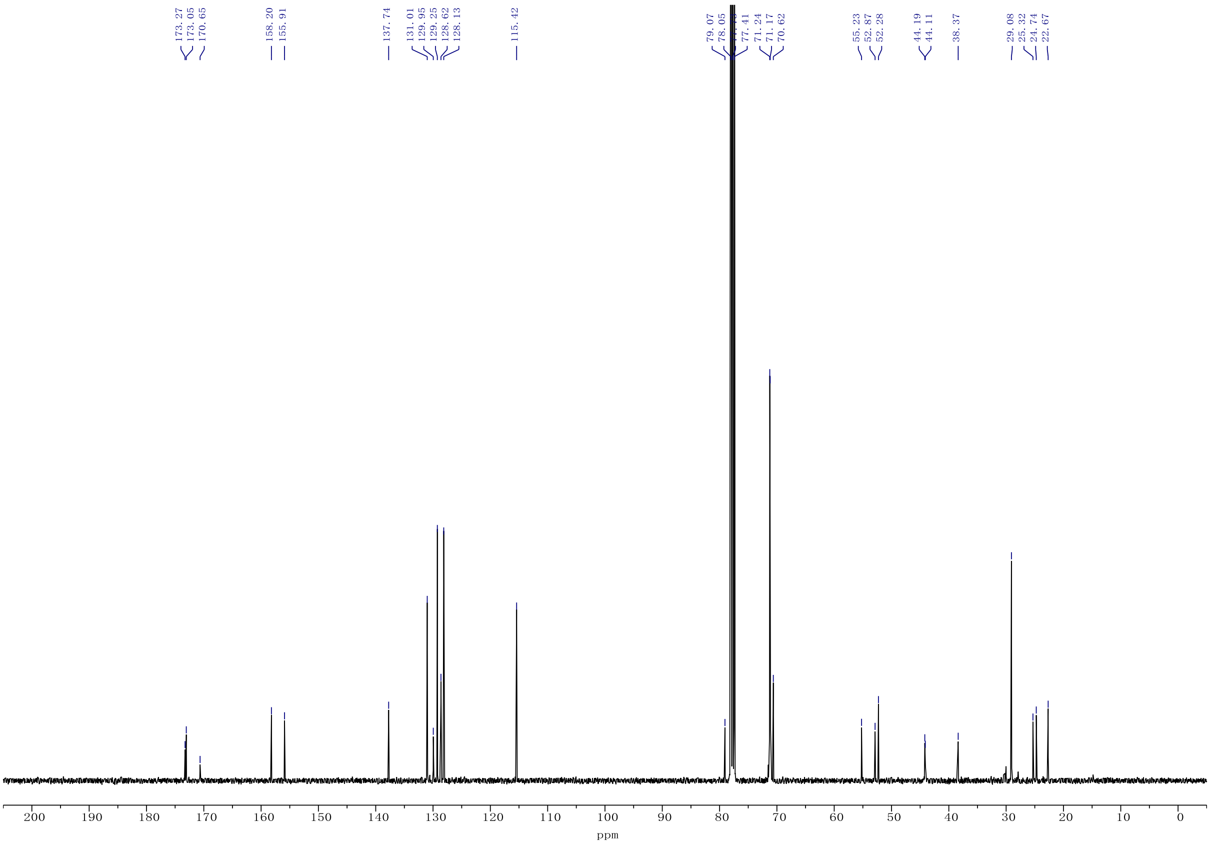

Spectrum S44: ^{13}C NMR (101 MHz, $CDCl_3$) spectrum of **24C8⊂7**.

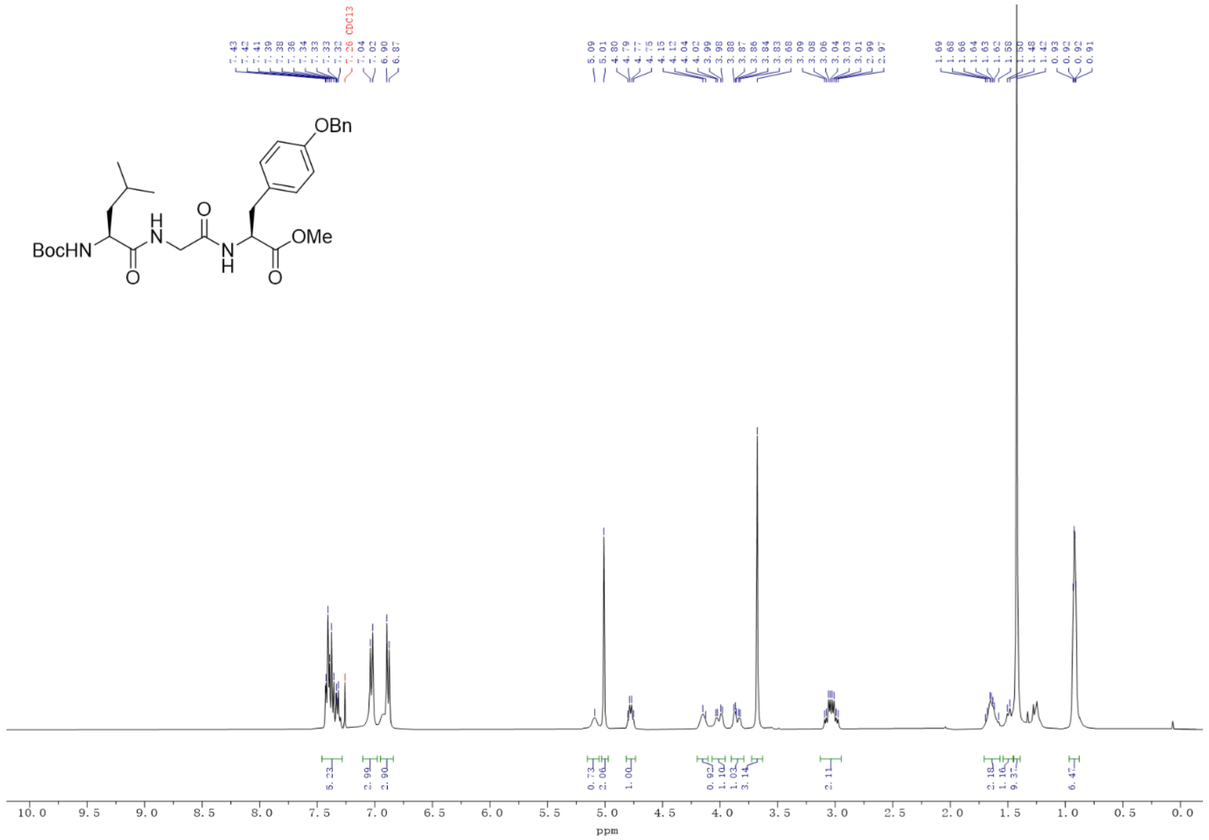

Spectrum S45: ^{1}H NMR (400 MHz, $CDCl_3$) spectrum of **7**.

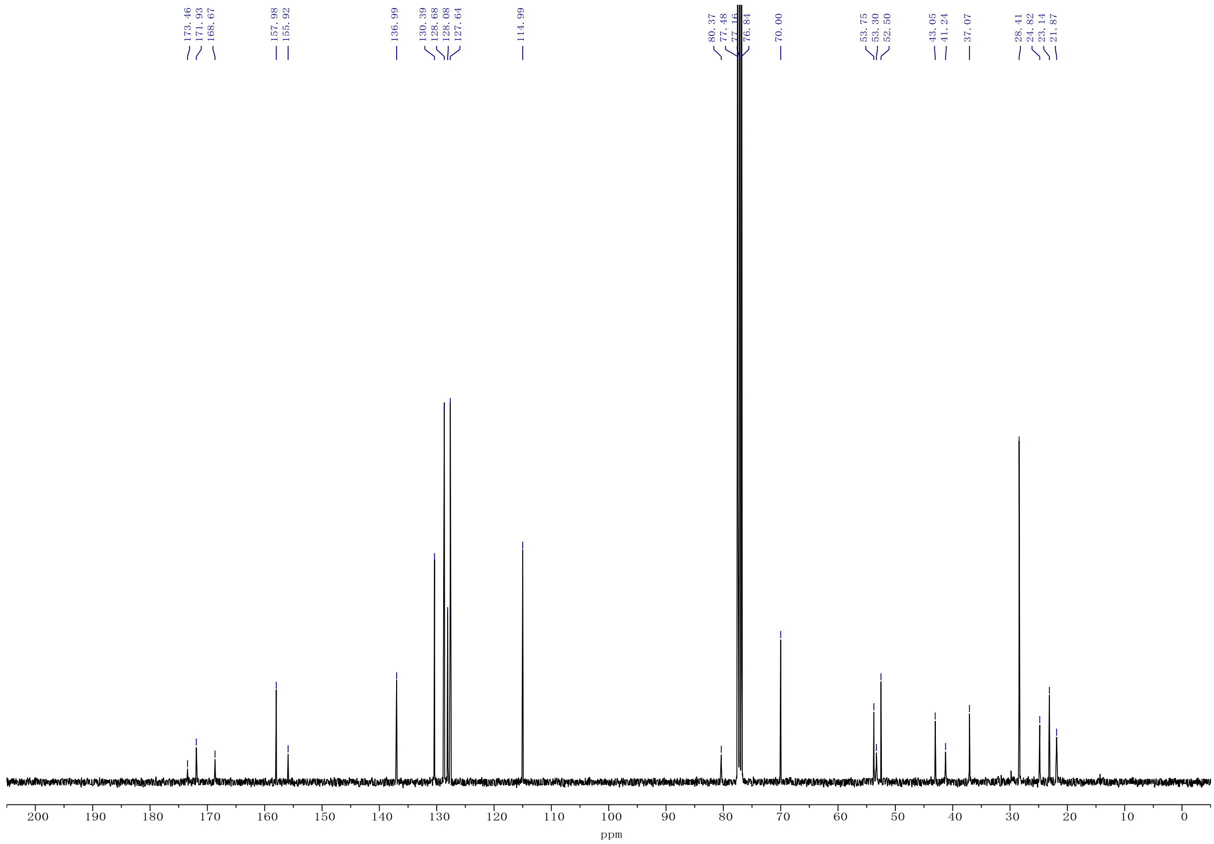

Spectrum S46: ^{13}C NMR (101 MHz, $CDCl_3$) spectrum of **7**.

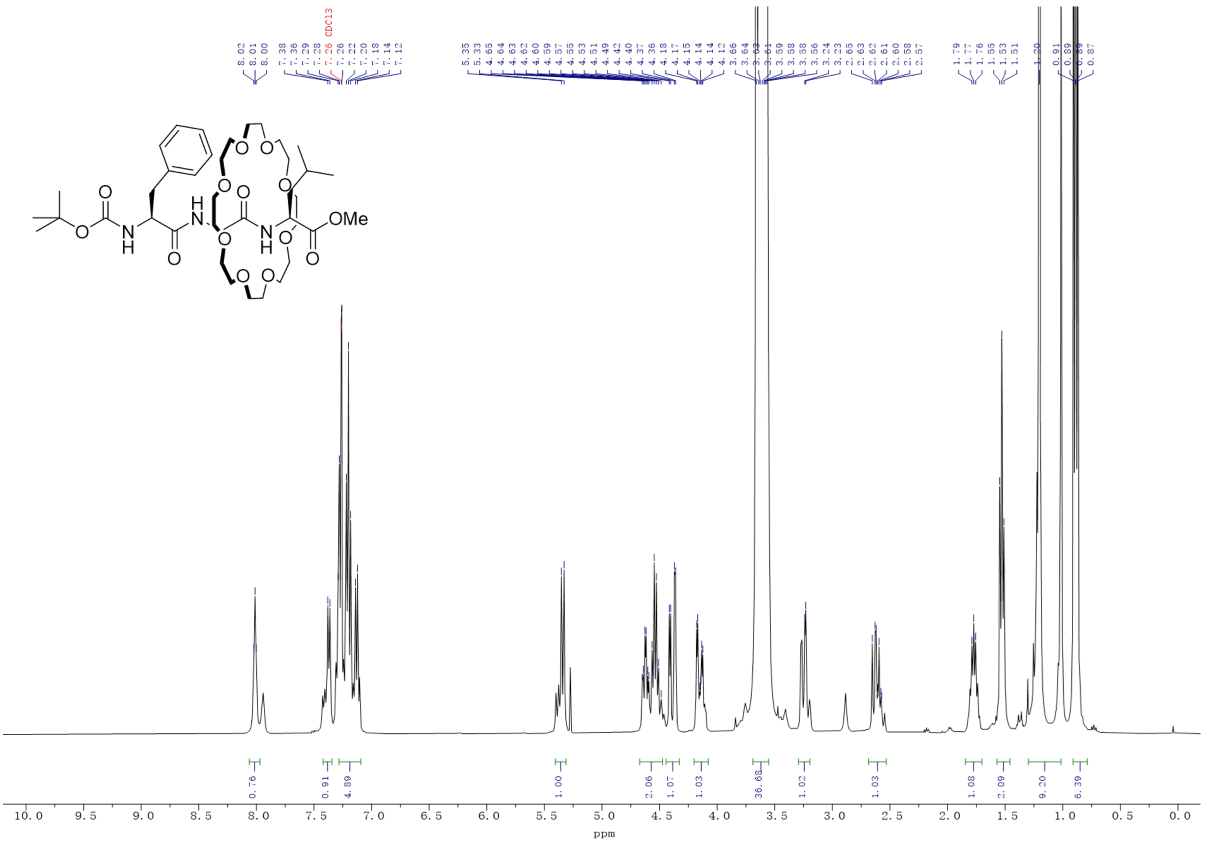

Spectrum S47: ^{1}H NMR (400 MHz, $CDCl_3$) spectrum of **24C8⊂8**.

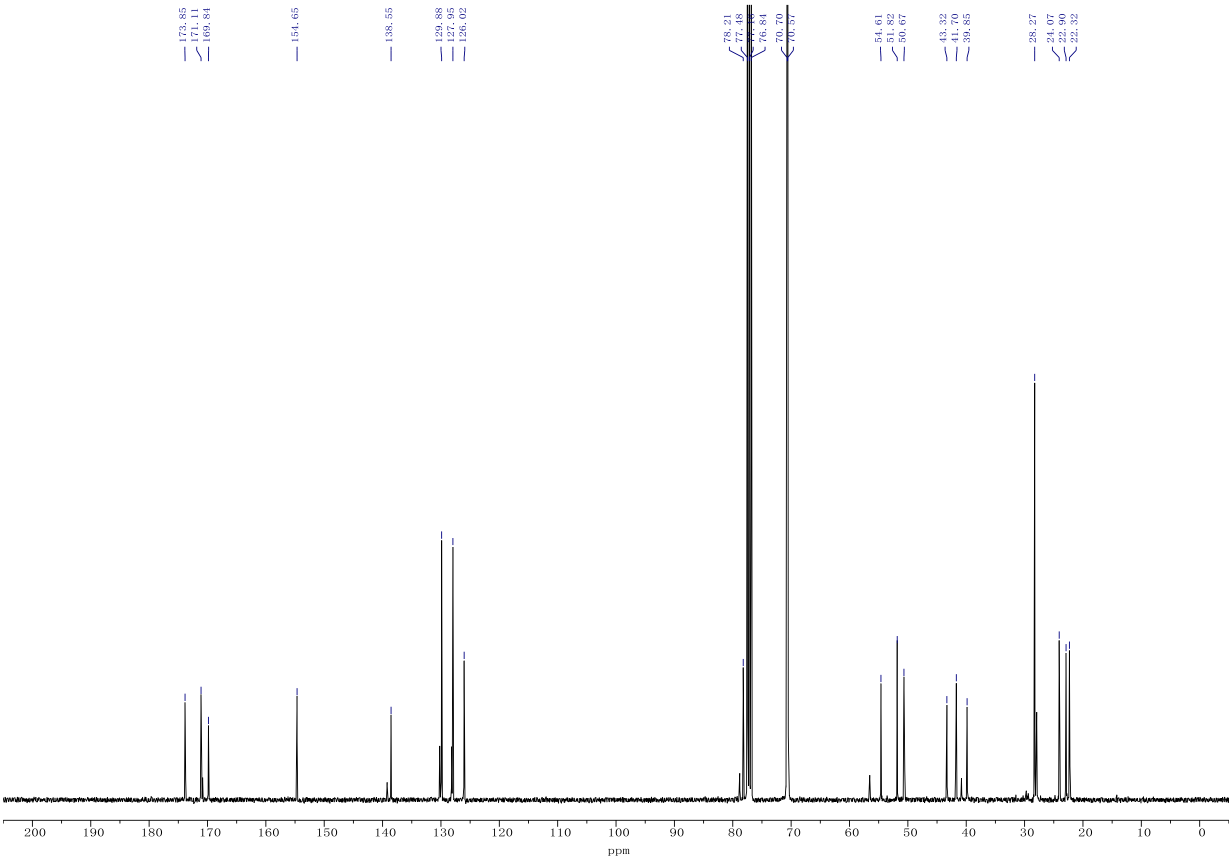

Spectrum S48: ^{13}C NMR (101 MHz, $CDCl_3$) spectrum of **24C8⊂8**.

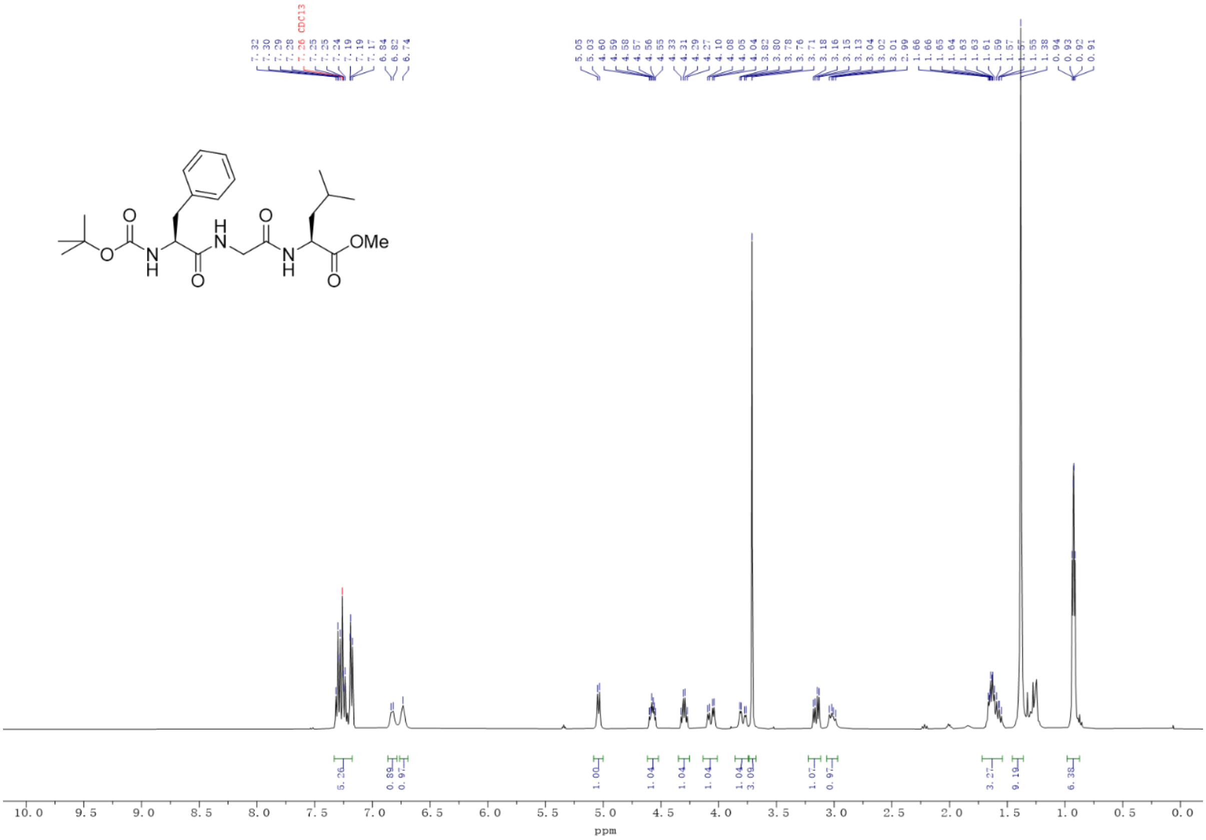

Spectrum S49: ^{1}H NMR (400 MHz, $CDCl_3$) spectrum of **8**.

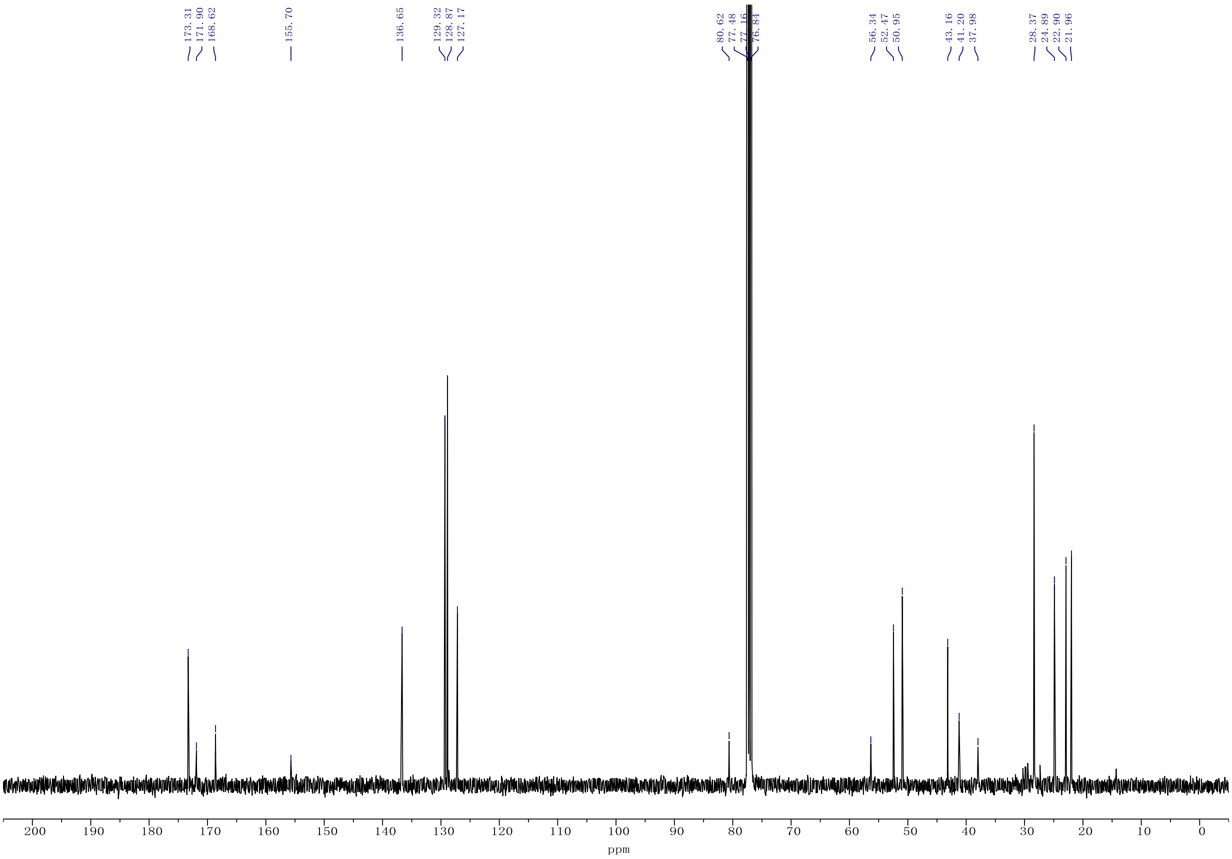

Spectrum S50: ^{13}C NMR (101 MHz, $CDCl_3$) spectrum of **8**.

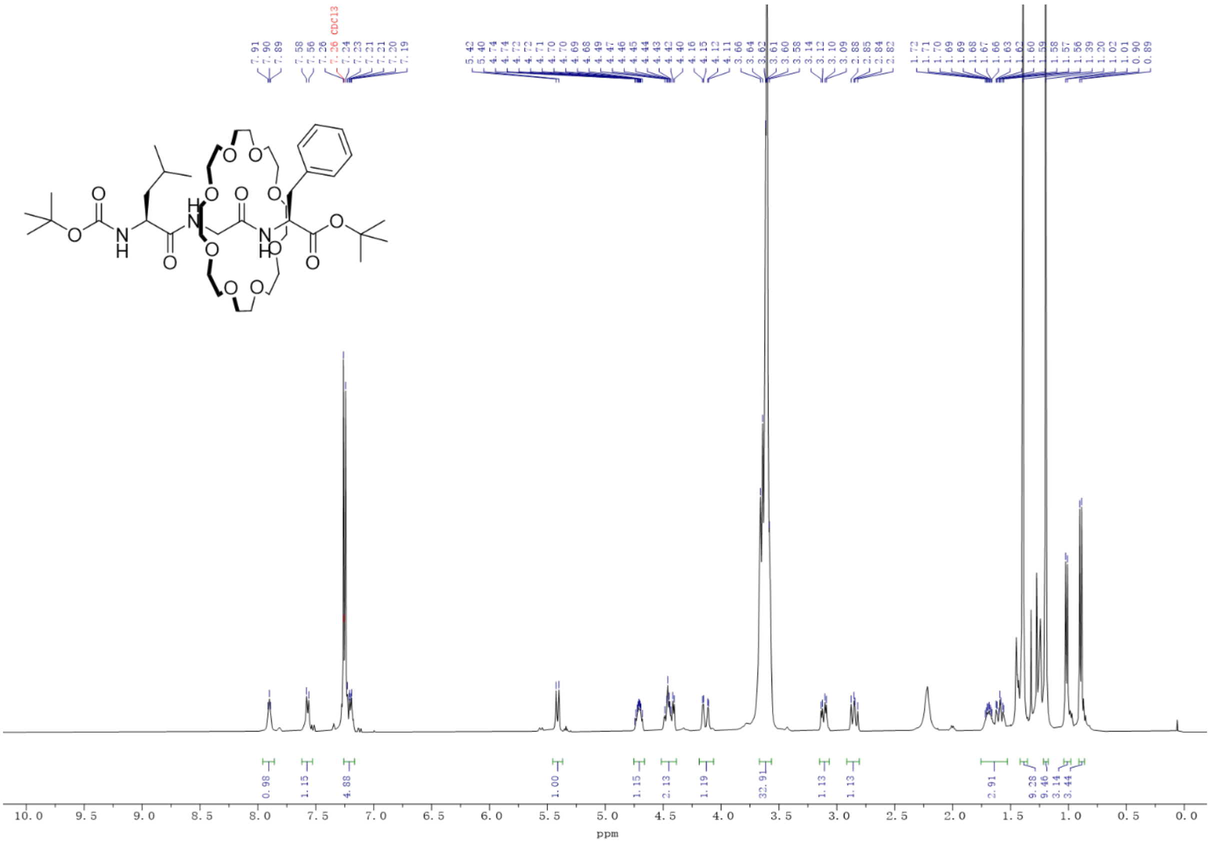

Spectrum S51: ^{1}H NMR (400 MHz, $CDCl_3$) spectrum of **24C8⊂9**.

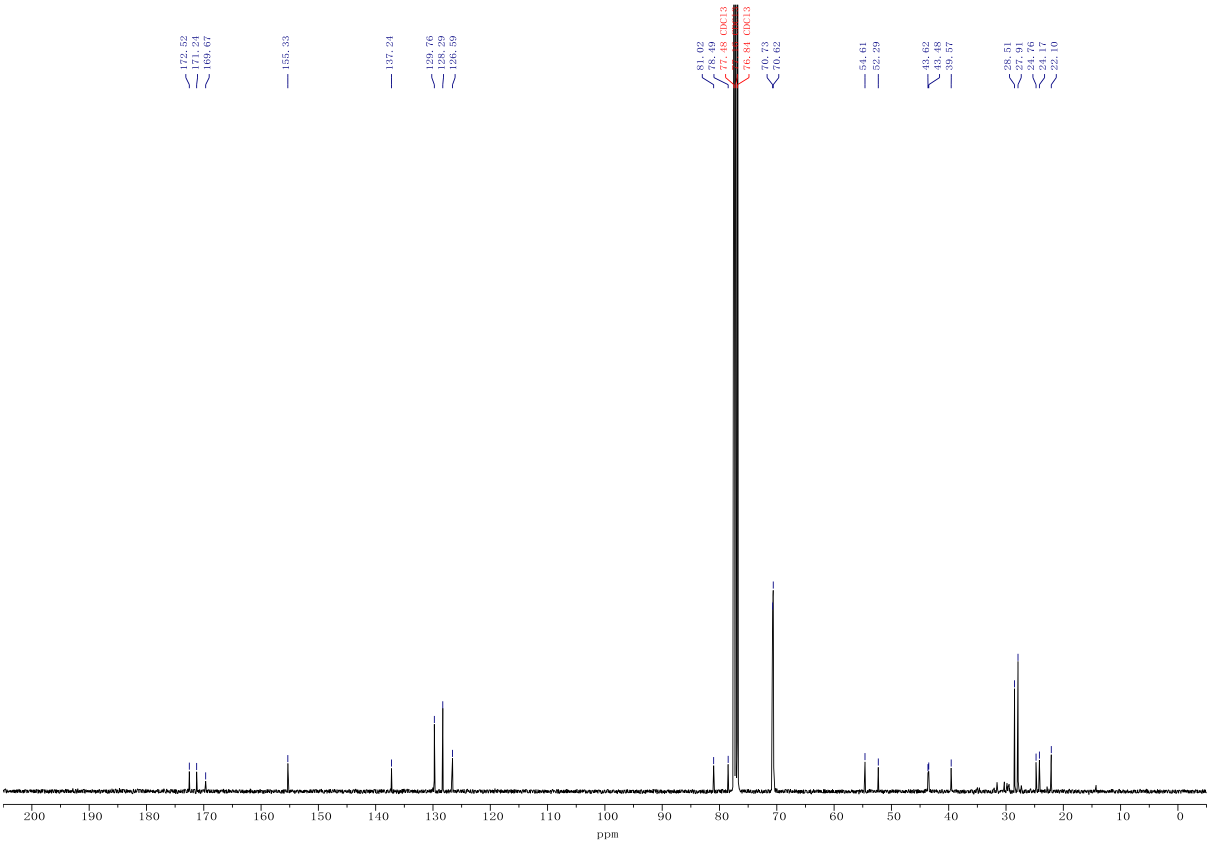

Spectrum S52: ^{13}C NMR (101 MHz, $CDCl_3$) spectrum of **24C8⊂9**.

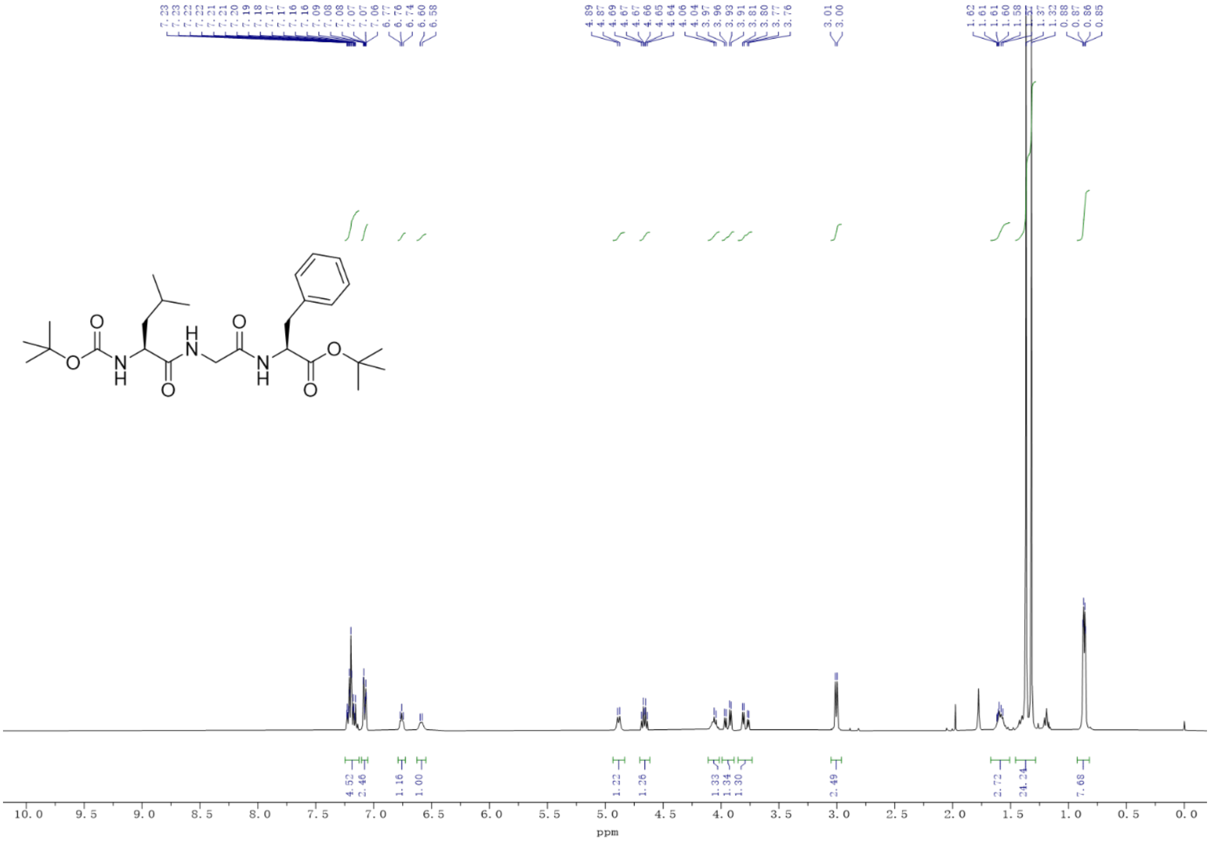

Spectrum S53: ^{1}H NMR (400 MHz, $CDCl_3$) spectrum of **9**.

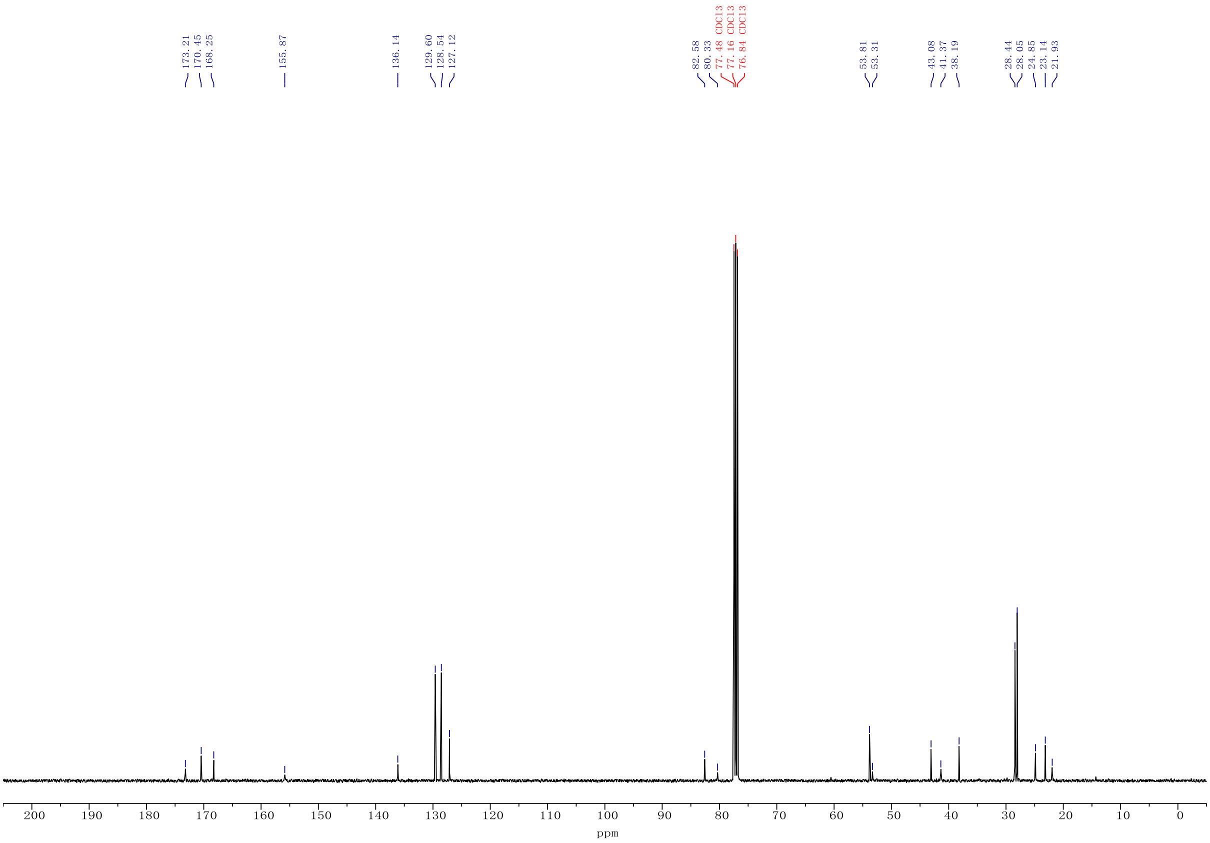

Spectrum S54: ^{13}C NMR (101 MHz, $CDCl_3$) spectrum of **9**.

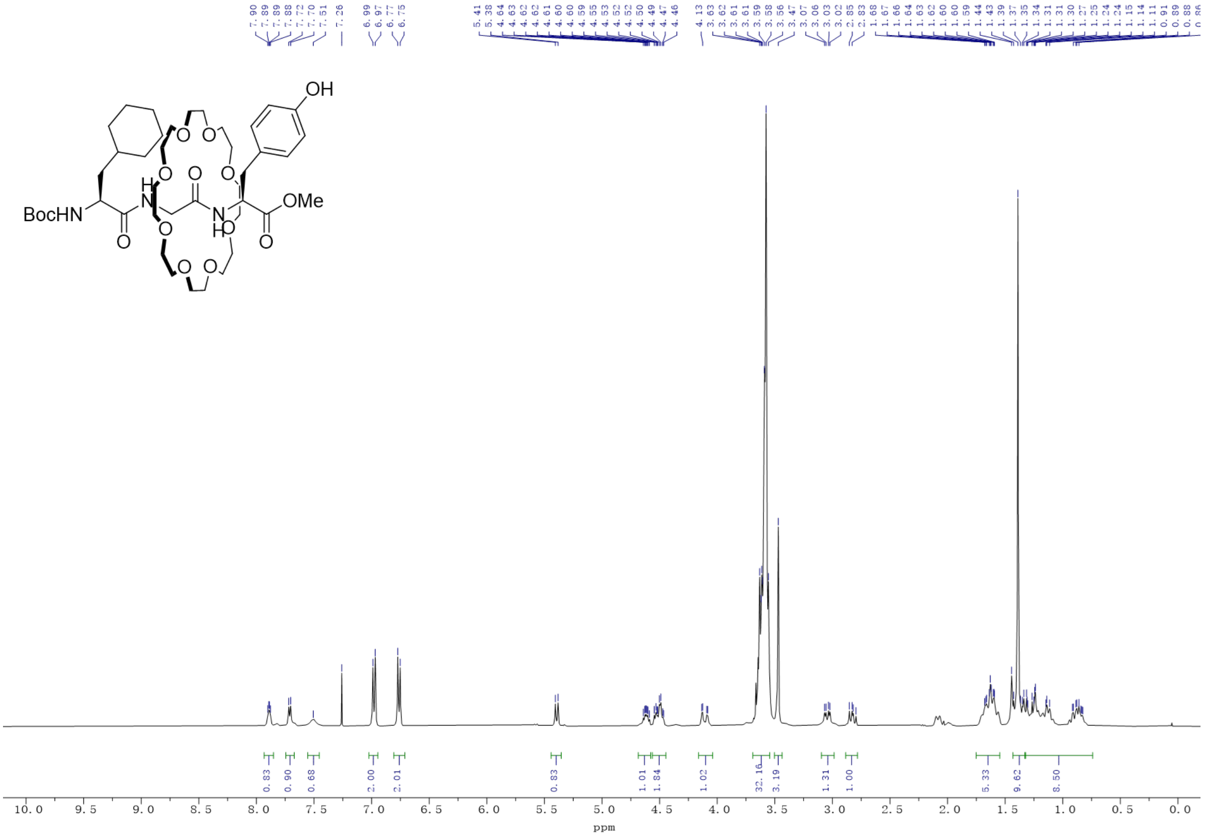

Spectrum S55: ^{1}H NMR (400 MHz, $CDCl_3$) spectrum of **24C8⊂10**.

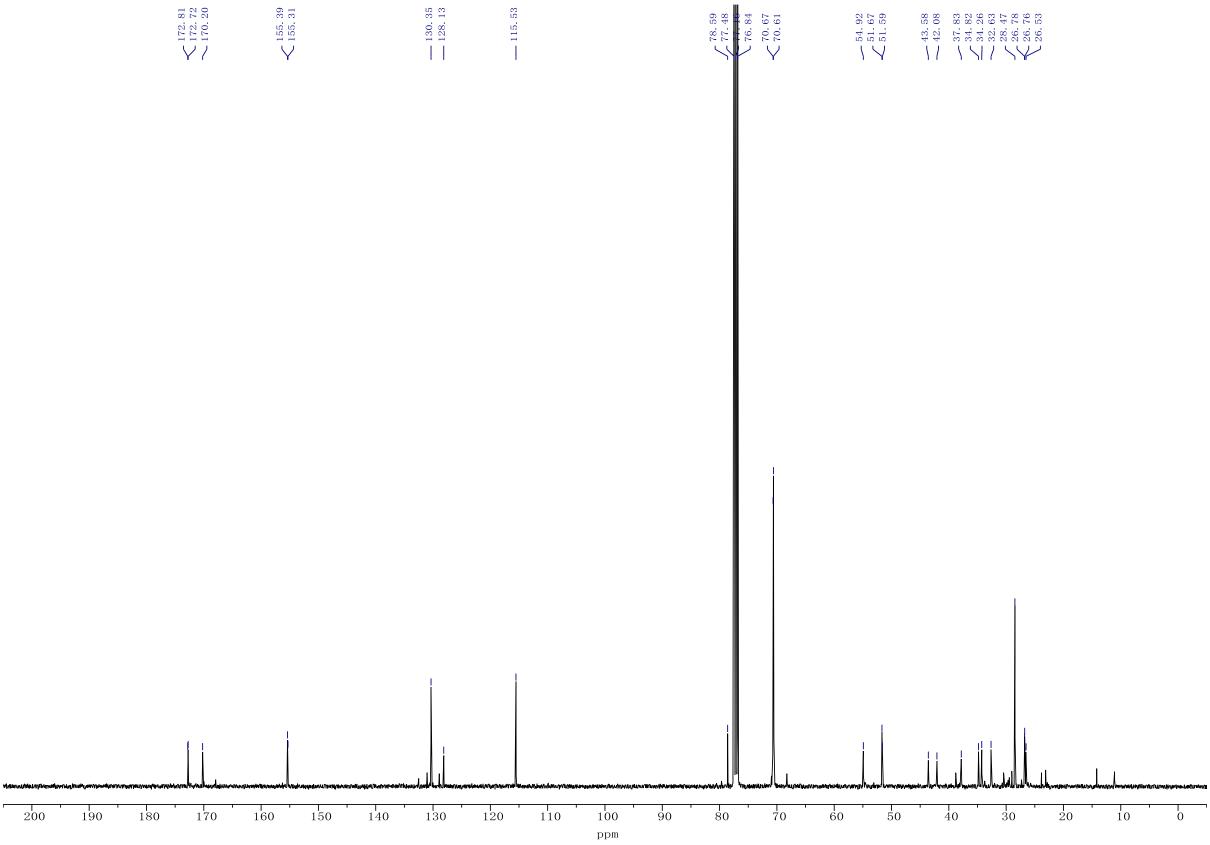

Spectrum S56: ^{13}C NMR (101 MHz, $CDCl_3$) spectrum of **24C8⊂10**.

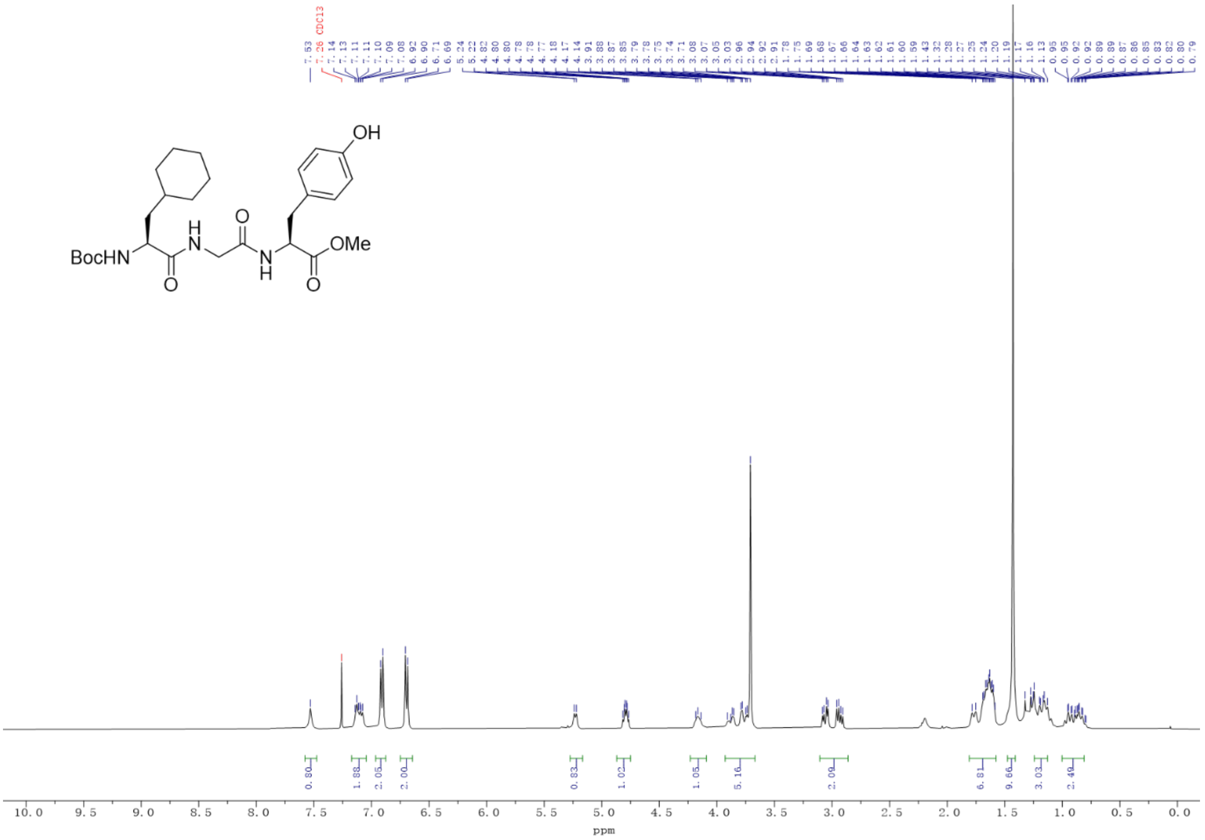

Spectrum S57: ^{1}H NMR (400 MHz, $CDCl_3$) spectrum of **10**.

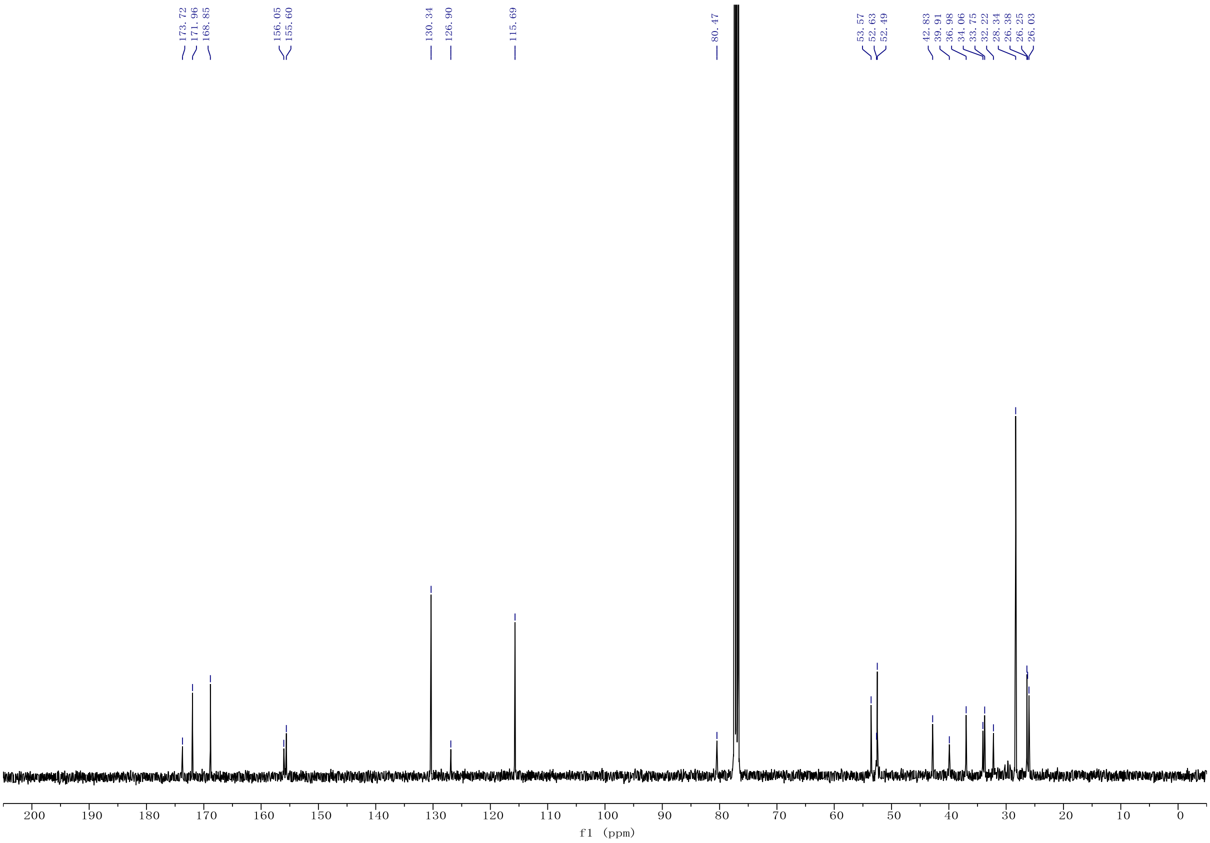

Spectrum S58: ^{13}C NMR (101 MHz, $CDCl_3$) spectrum of **10**.

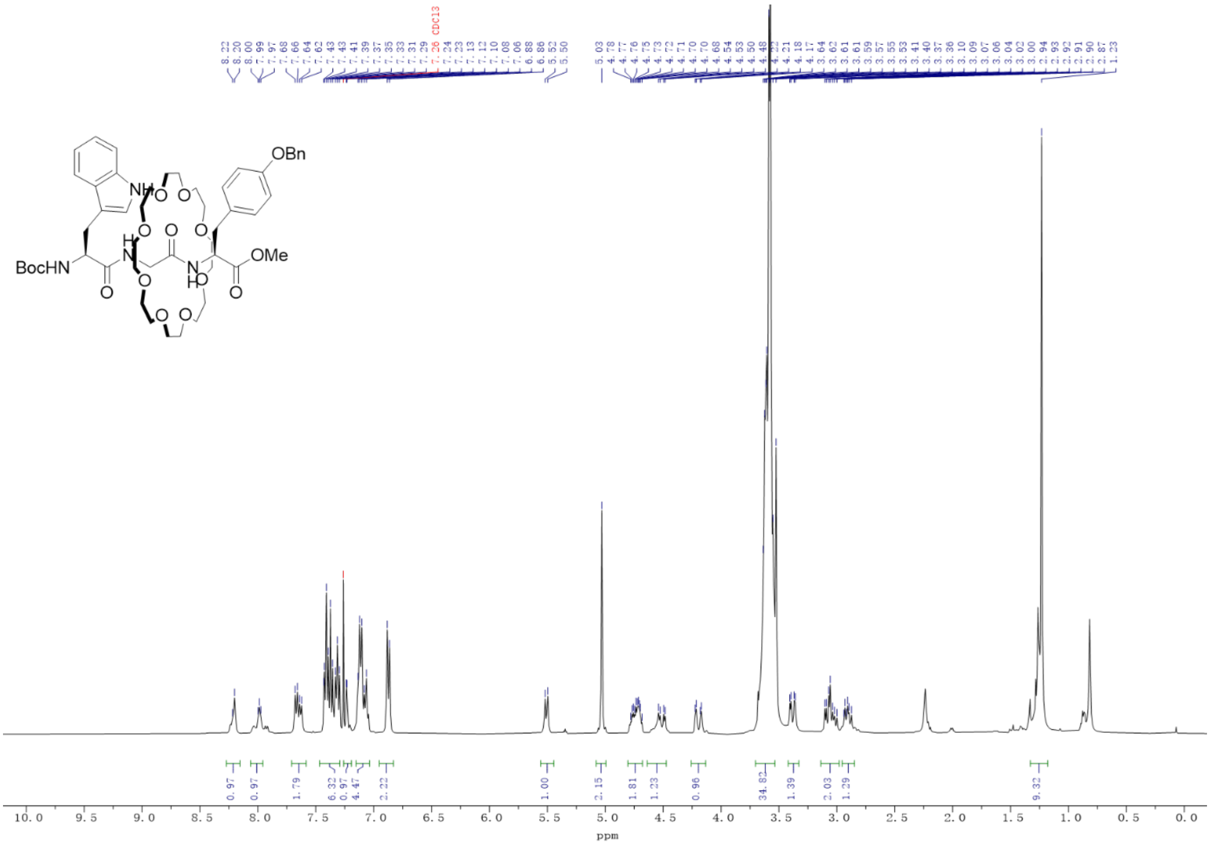

Spectrum S59: ^{1}H NMR (400 MHz, $CDCl_3$) spectrum of **24C8⊂11**.

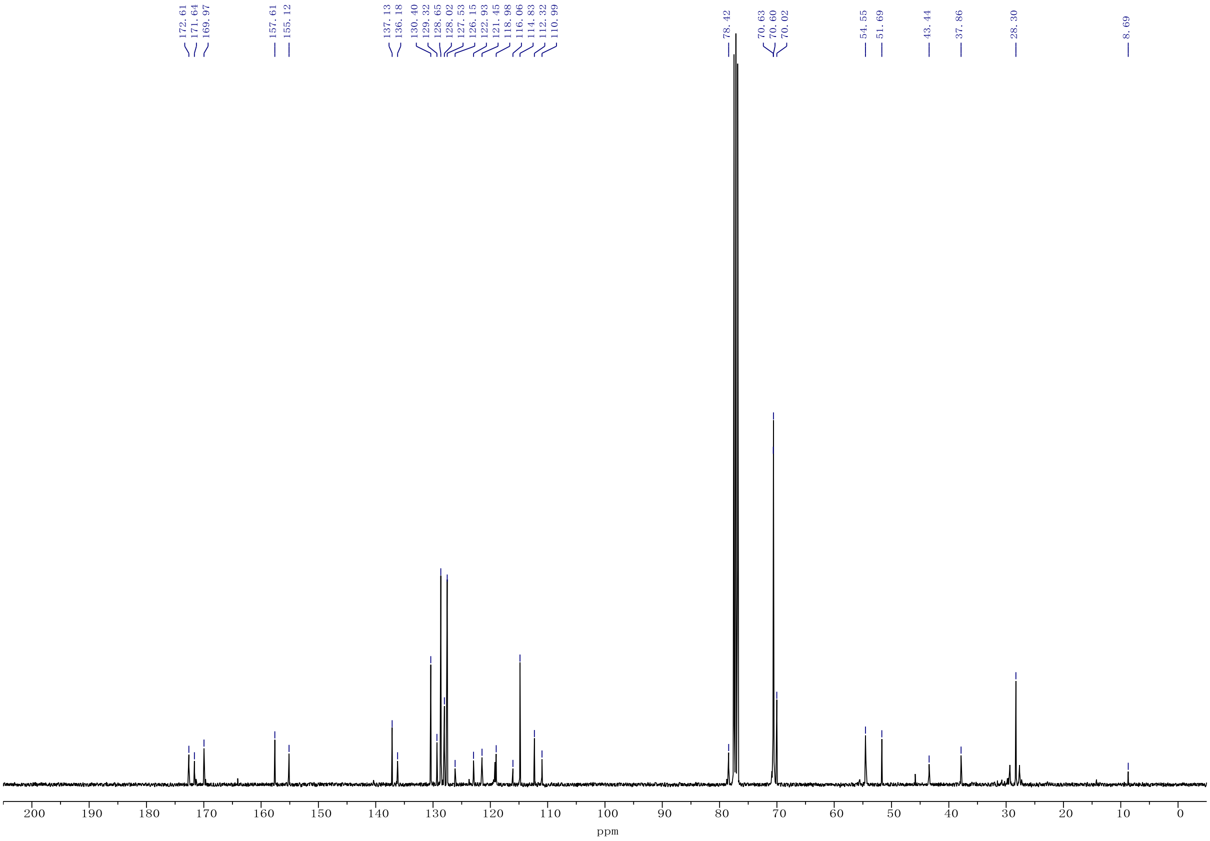

Spectrum S60: ^{13}C NMR (101 MHz, $CDCl_3$) spectrum of **24C8⊂11**.

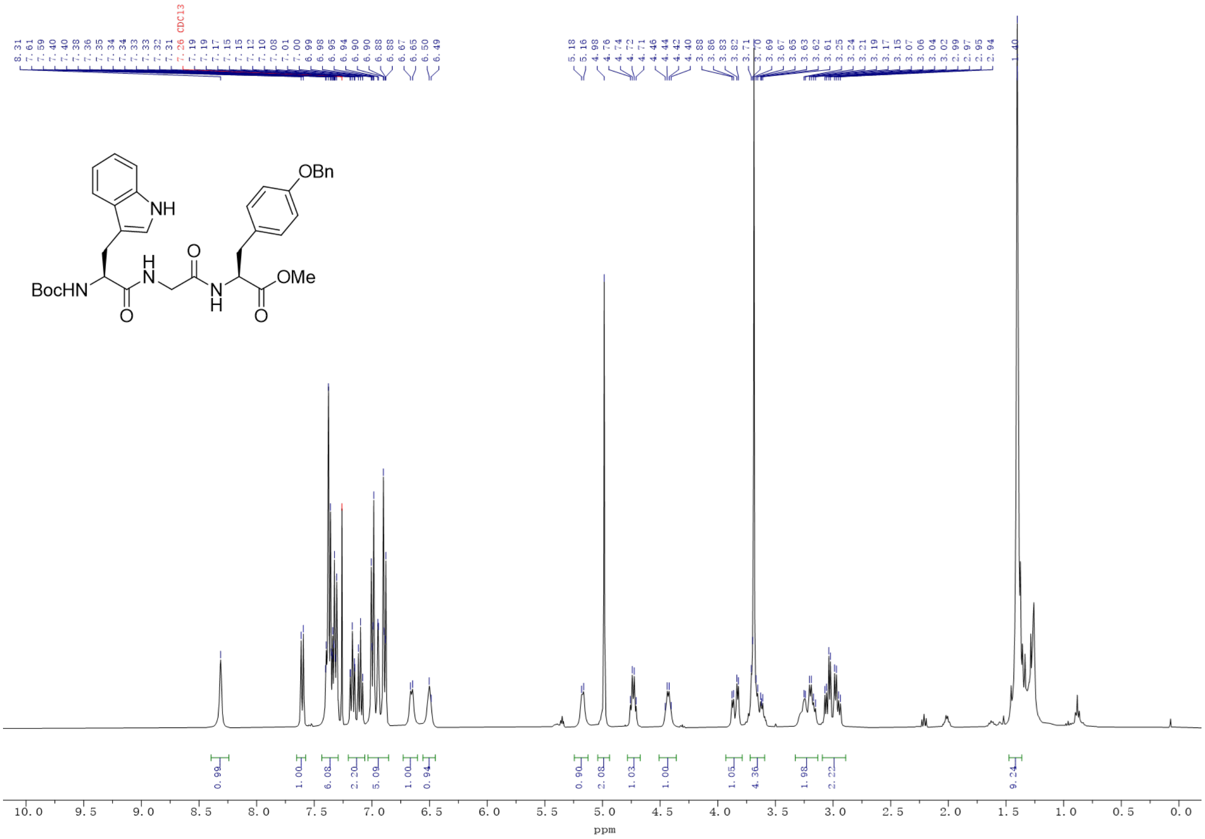

Spectrum S61: ^{1}H NMR (400 MHz, $CDCl_3$) spectrum of **11**.

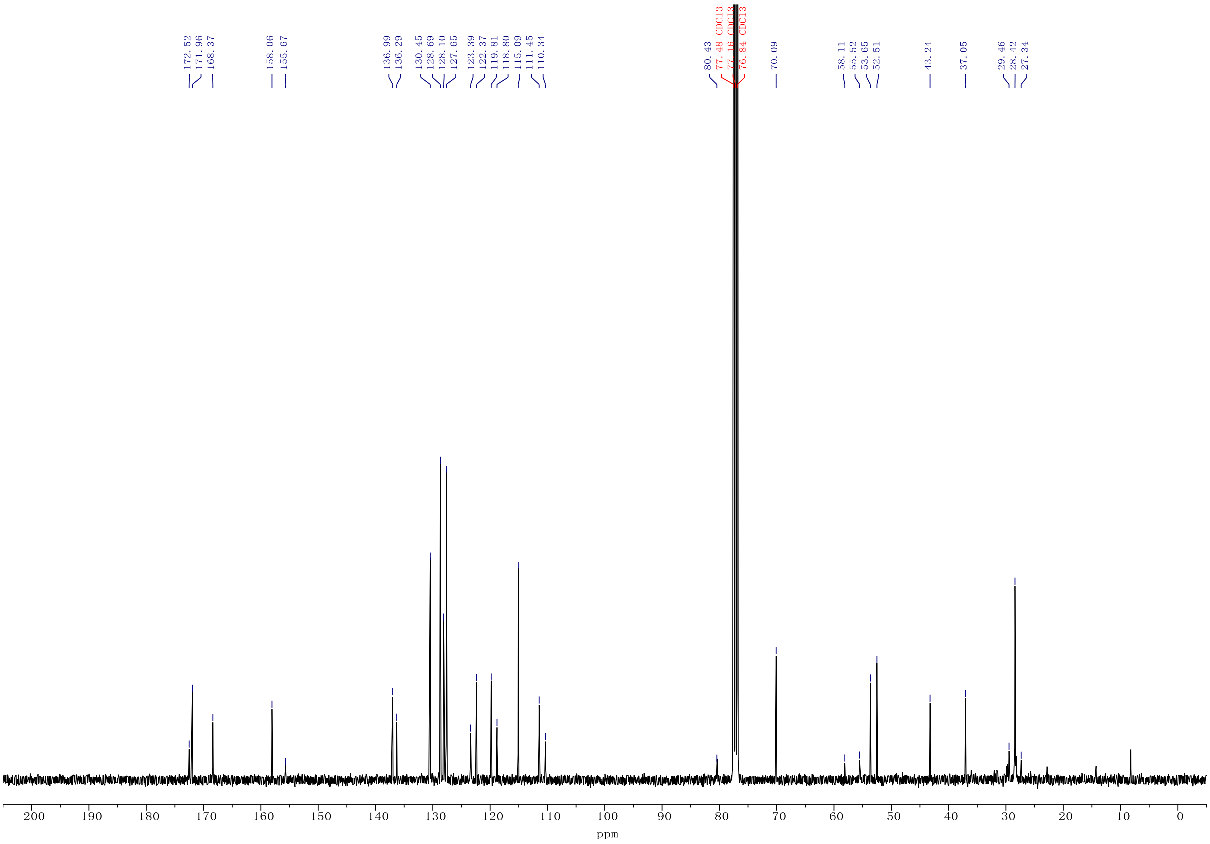

Spectrum S62: ^{13}C NMR (101 MHz, $CDCl_3$) spectrum of **11**.

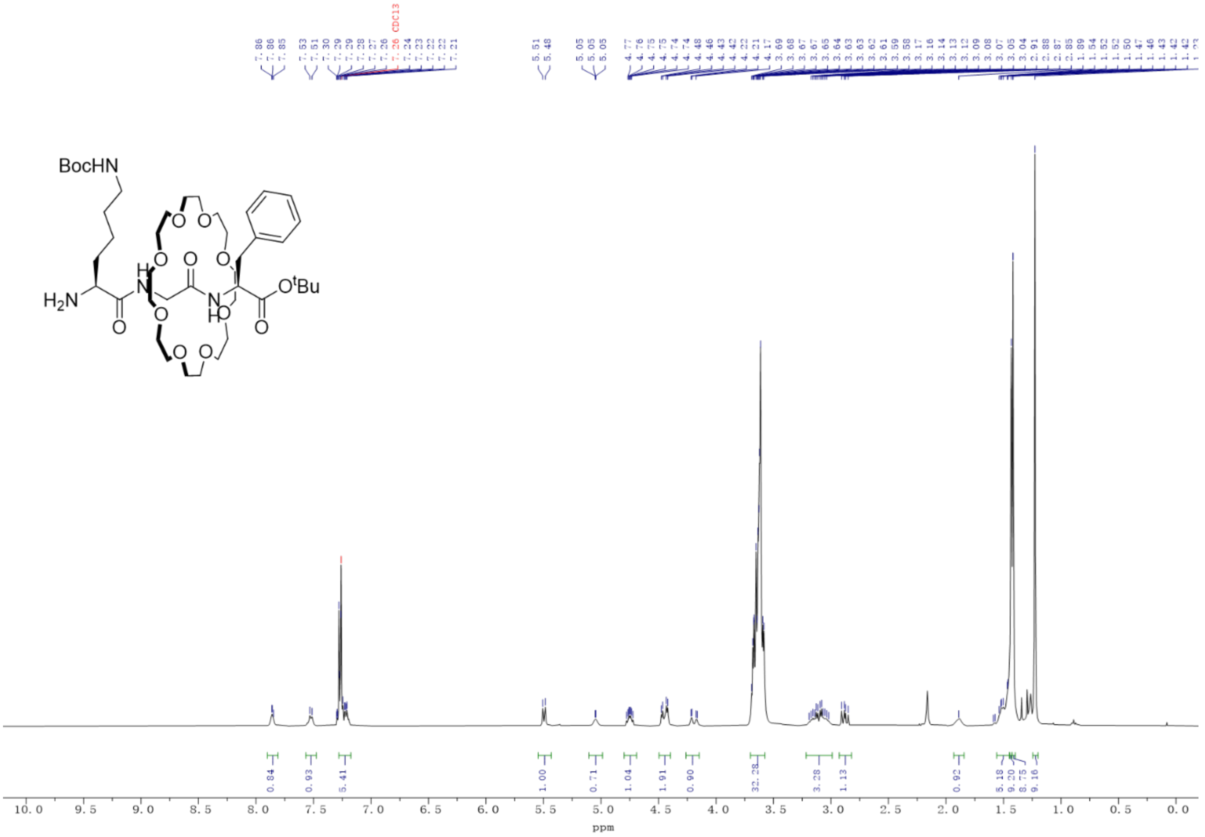

Spectrum S63: ^{1}H NMR (400 MHz, $CDCl_3$) spectrum of **24C8⊂12**.

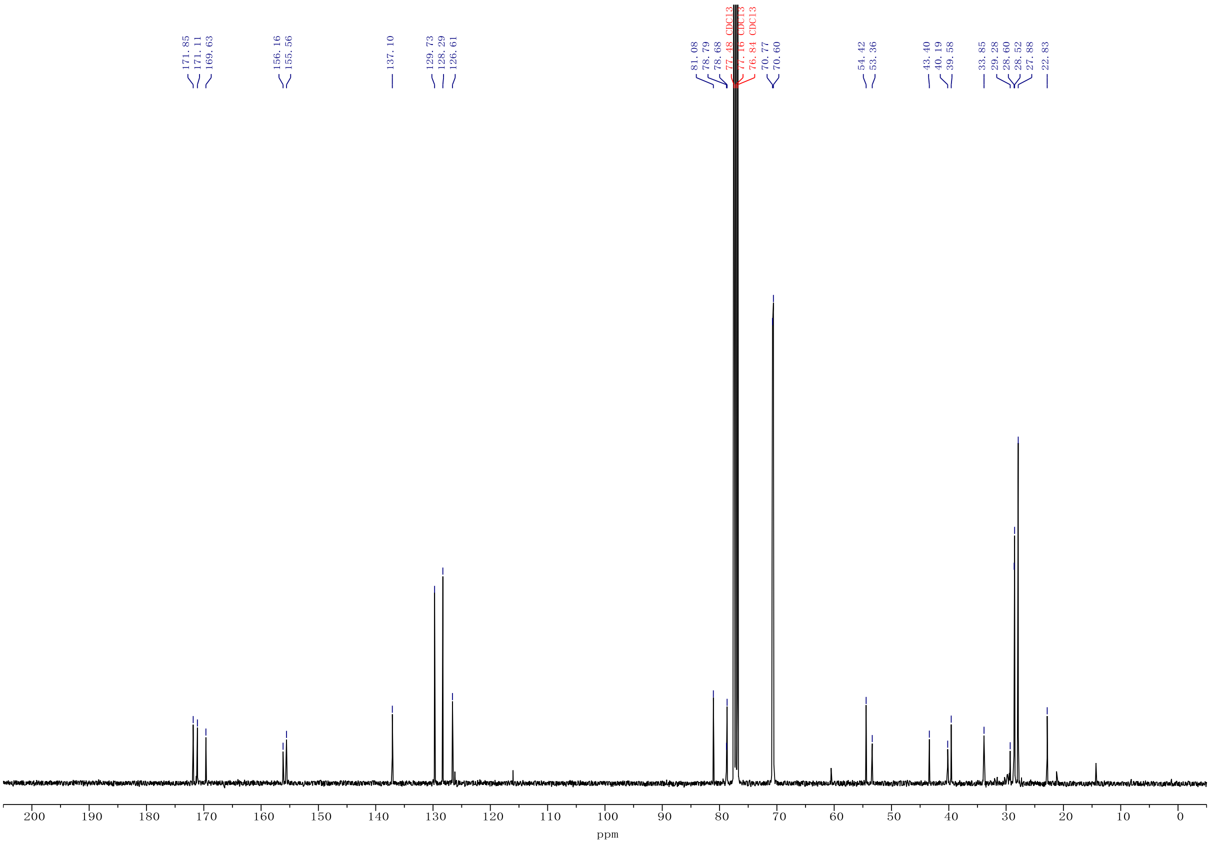

Spectrum S64: ^{13}C NMR (101 MHz, $CDCl_3$) spectrum of **24C8⊂12**.

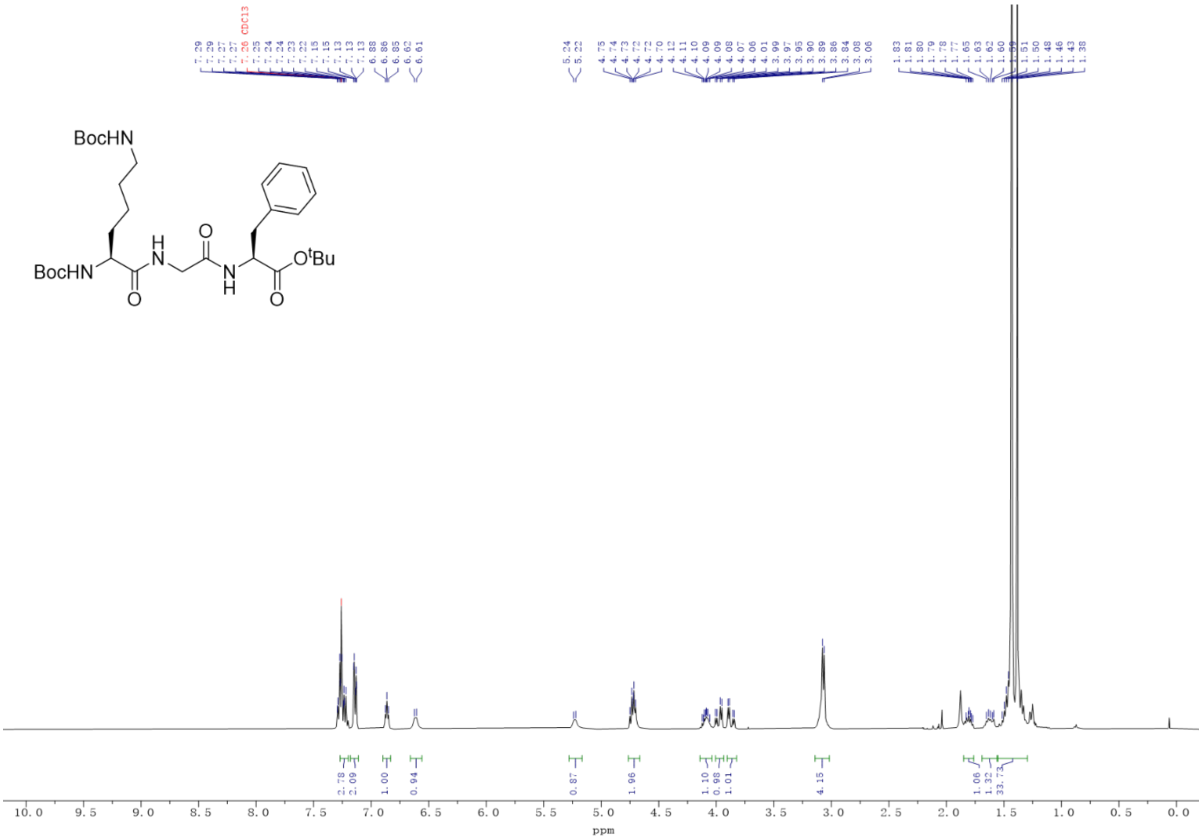

Spectrum S65: ^{1}H NMR (400 MHz, $CDCl_3$) spectrum of **12**.

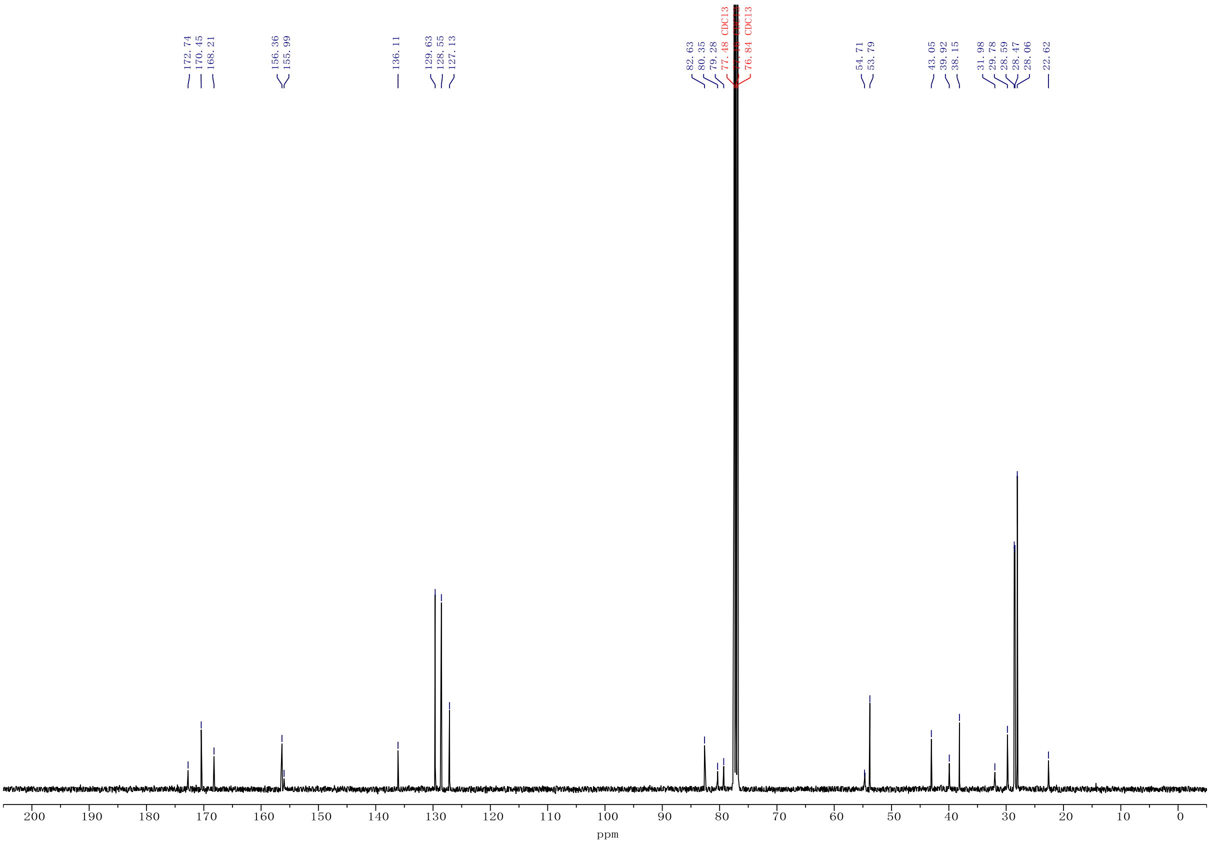

Spectrum S66: ^{13}C NMR (101 MHz, $CDCl_3$) spectrum of **12**.

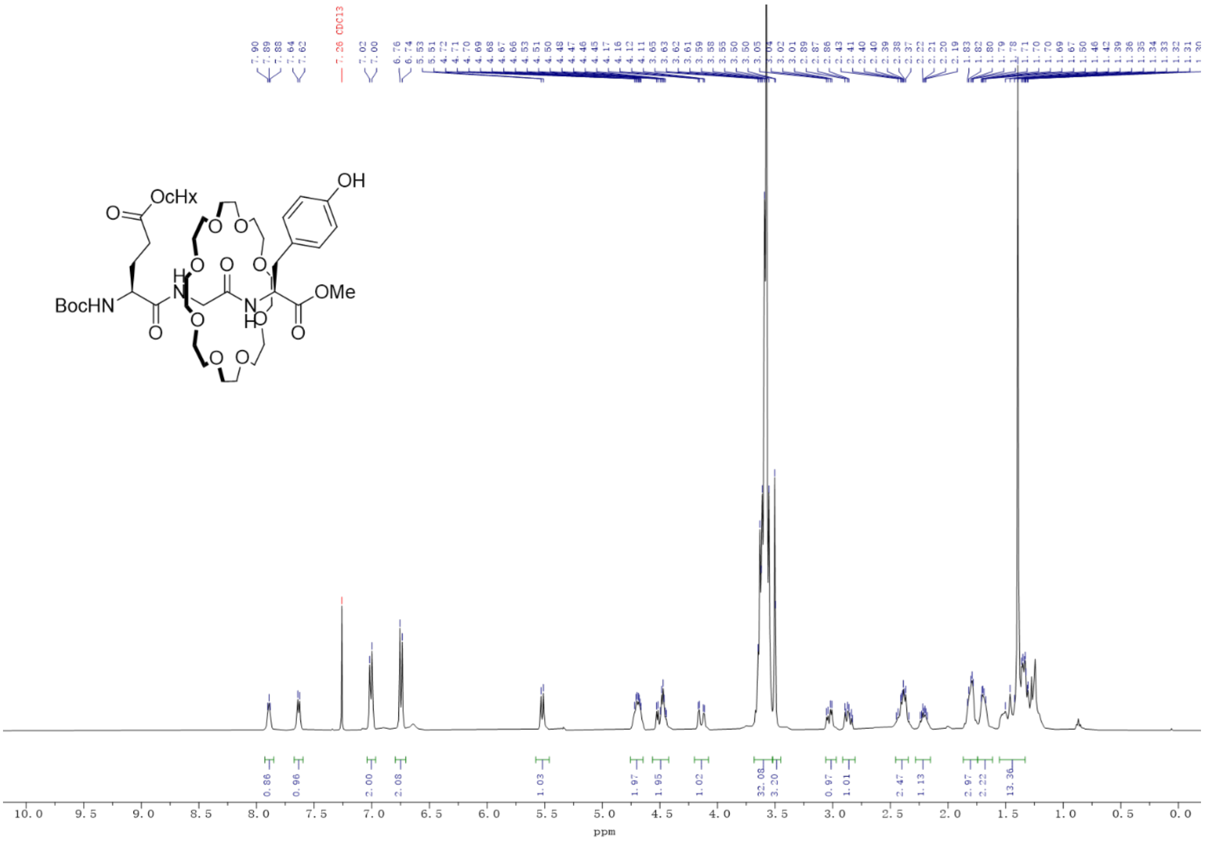

Spectrum S67: ^{1}H NMR (400 MHz, $CDCl_3$) spectrum of **24C8⊂13**.

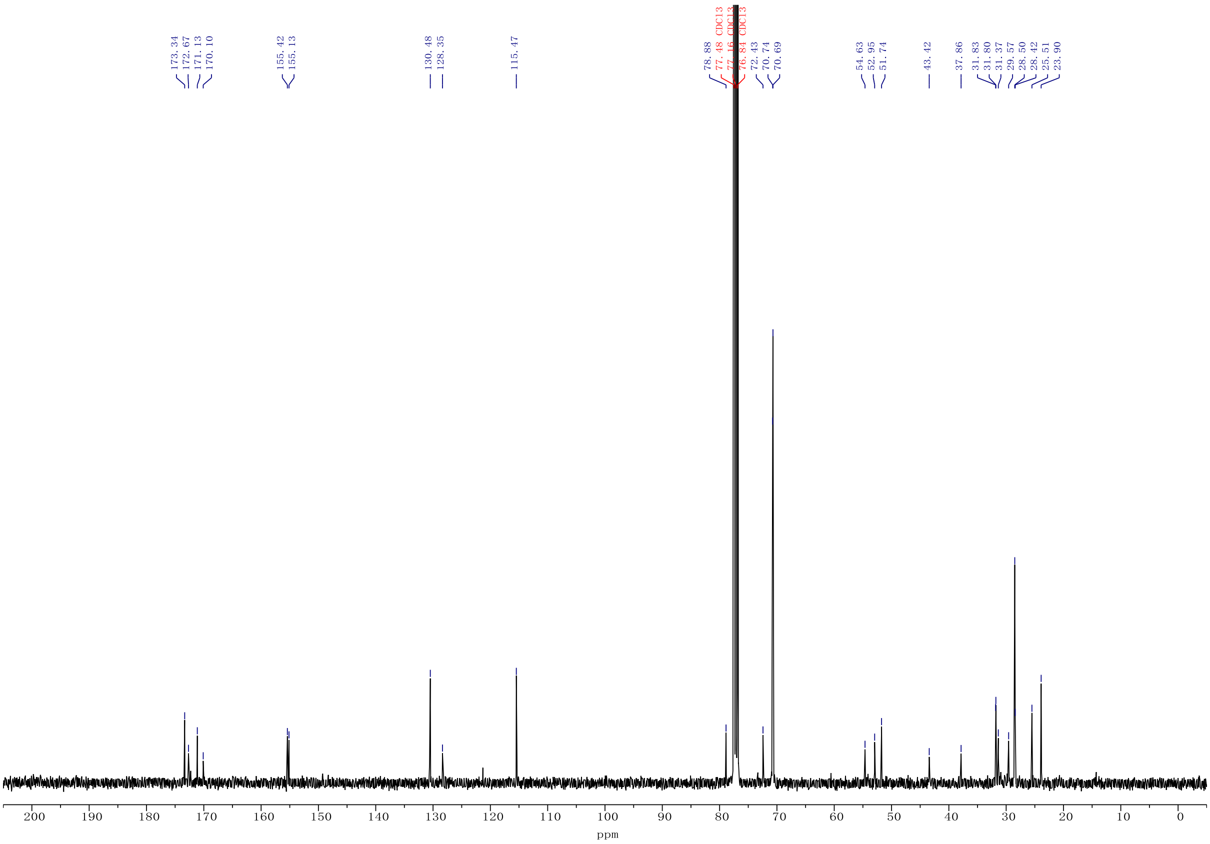

Spectrum S68: ^{13}C NMR (101 MHz, $CDCl_3$) spectrum of **24C8⊂13**.

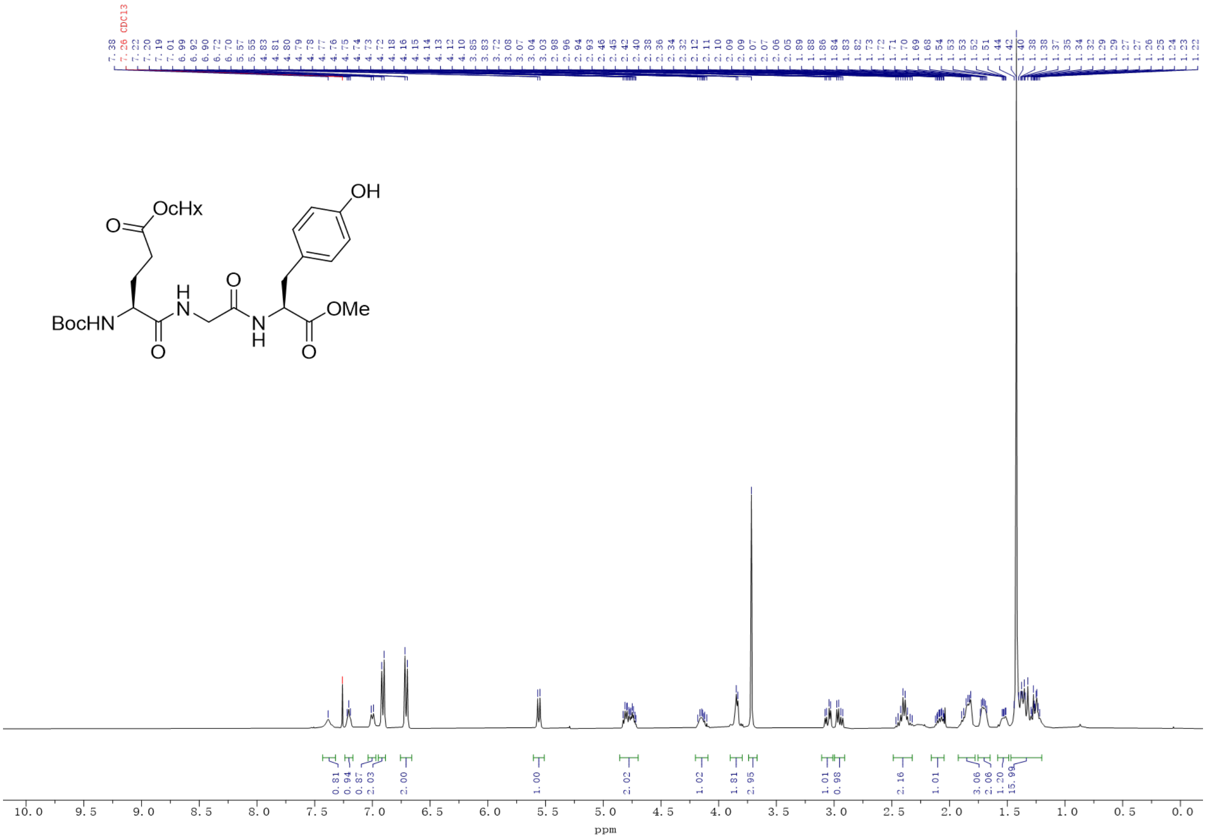

Spectrum S69: ^{1}H NMR (400 MHz, $CDCl_3$) spectrum of **13**.

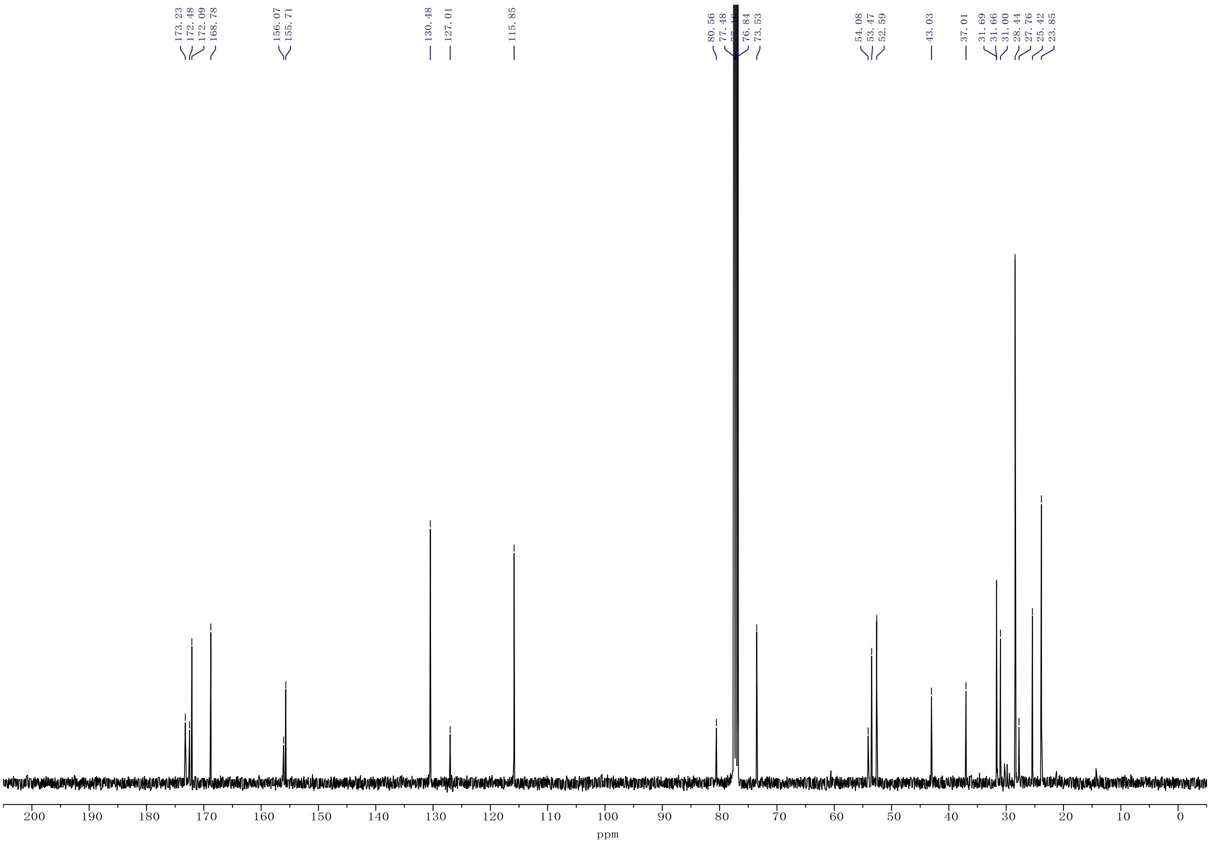

Spectrum S70: ^{13}C NMR (101 MHz, $CDCl_3$) spectrum of **13**.

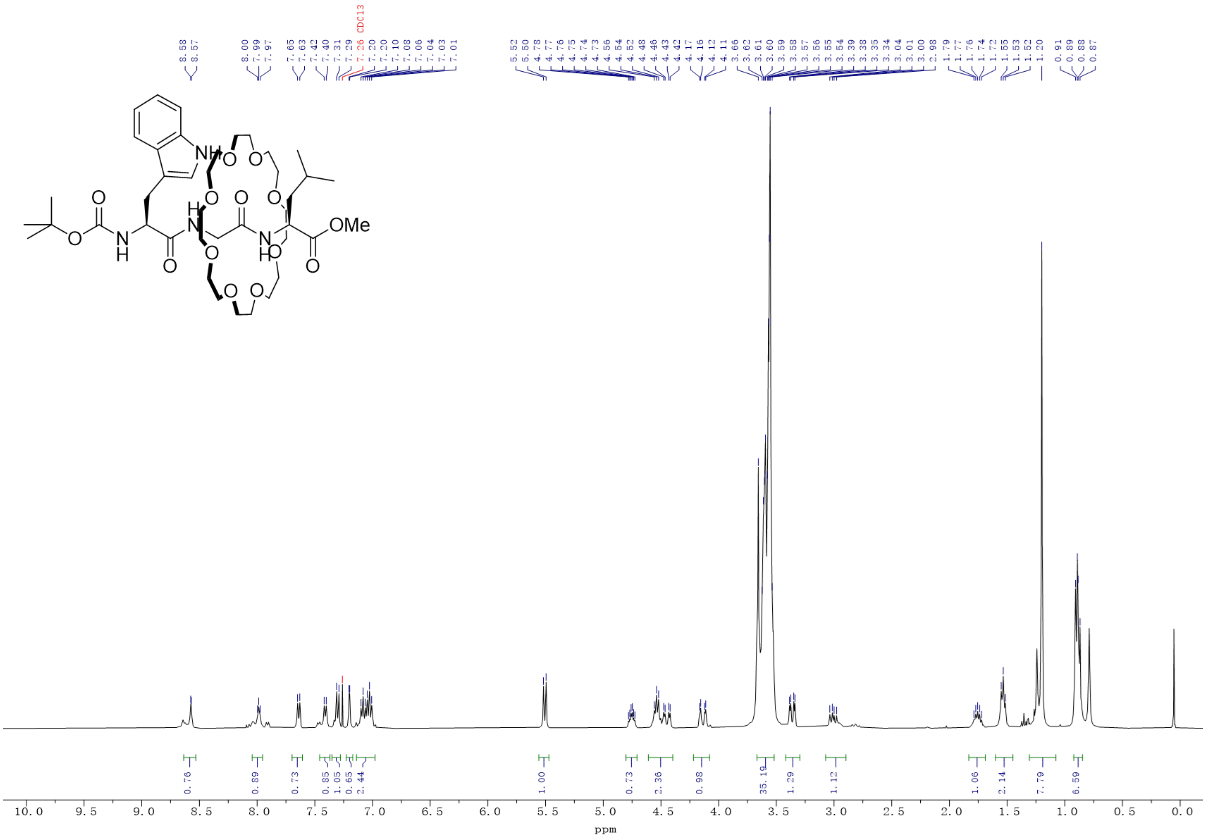

Spectrum S71: ^{1}H NMR (400 MHz, $CDCl_3$) spectrum of **24C8⊂14**.

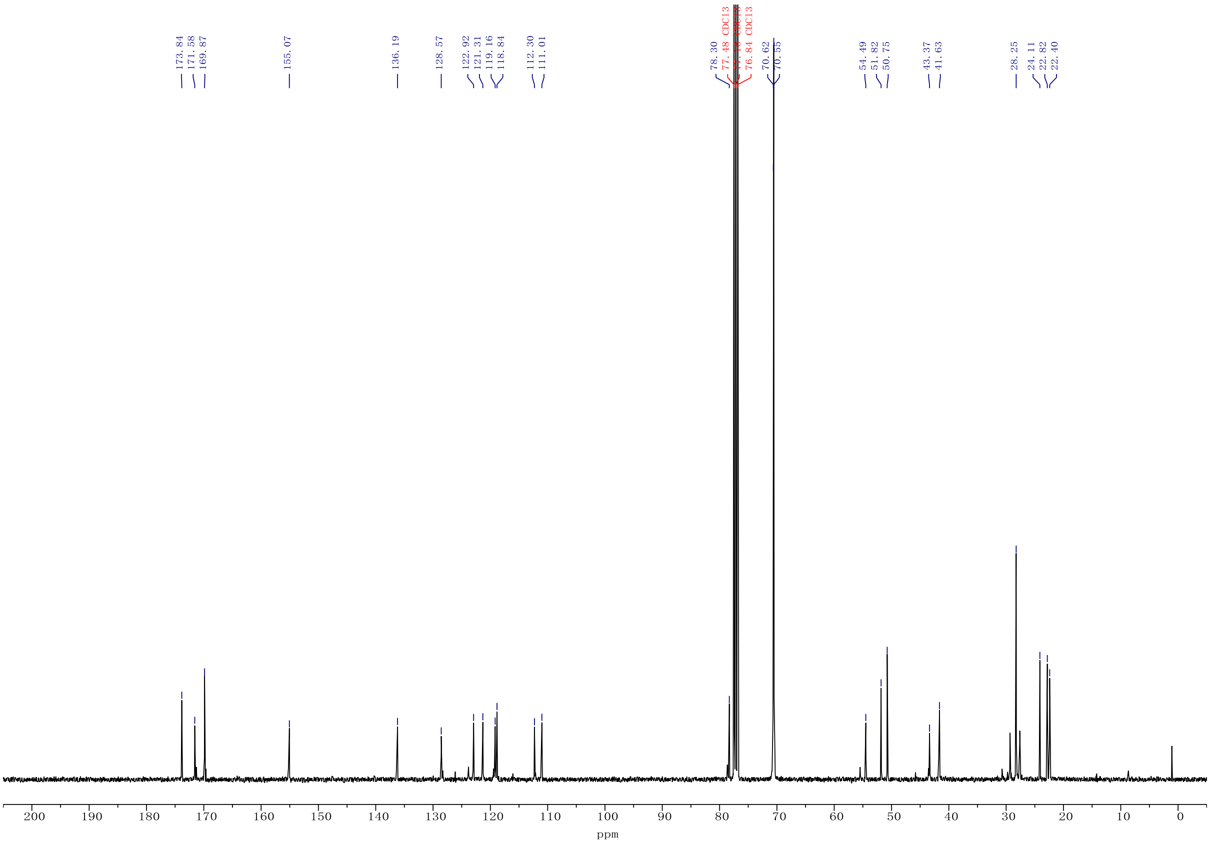

Spectrum S72: ^{13}C NMR (101 MHz, $CDCl_3$) spectrum of **24C8⊂14**.

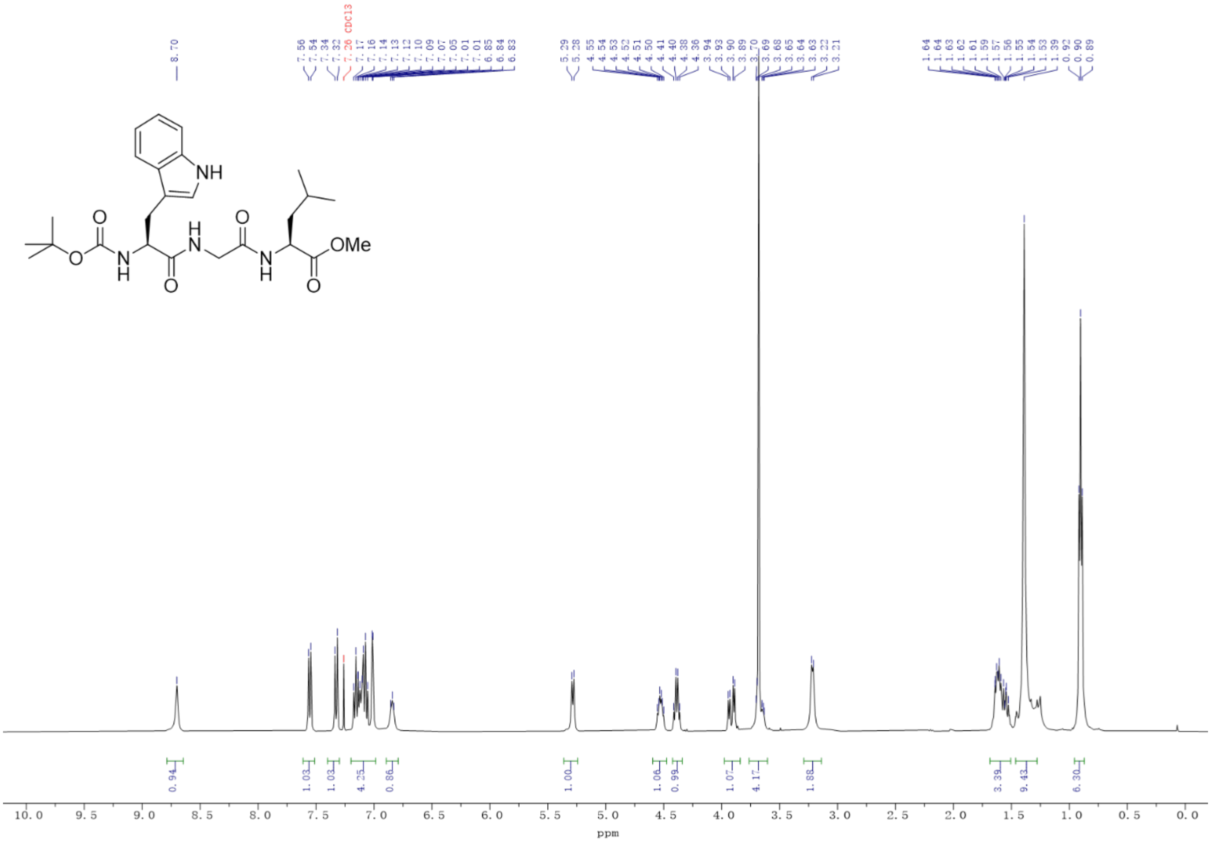

Spectrum S73: ^{1}H NMR (400 MHz, $CDCl_3$) spectrum of **14**.

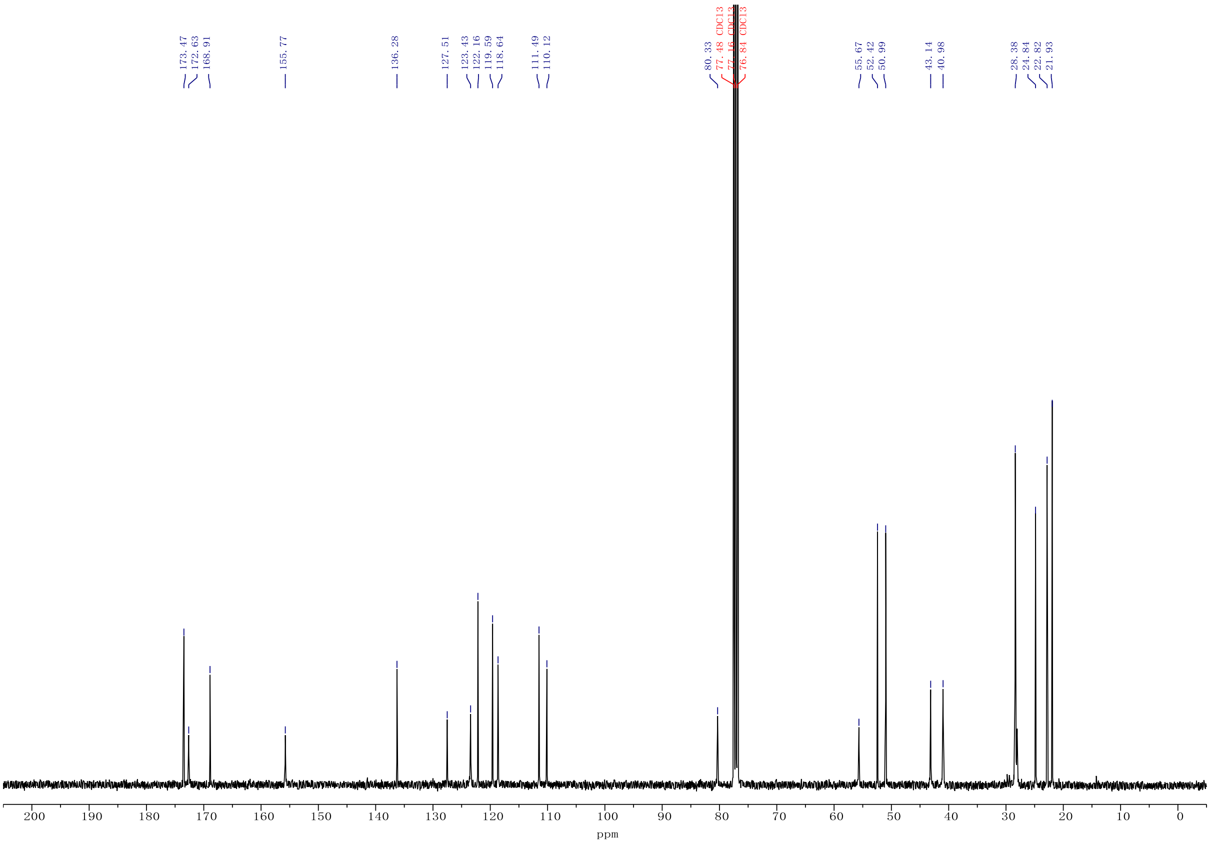

Spectrum S74: ^{13}C NMR (101 MHz, $CDCl_3$) spectrum of **14**.

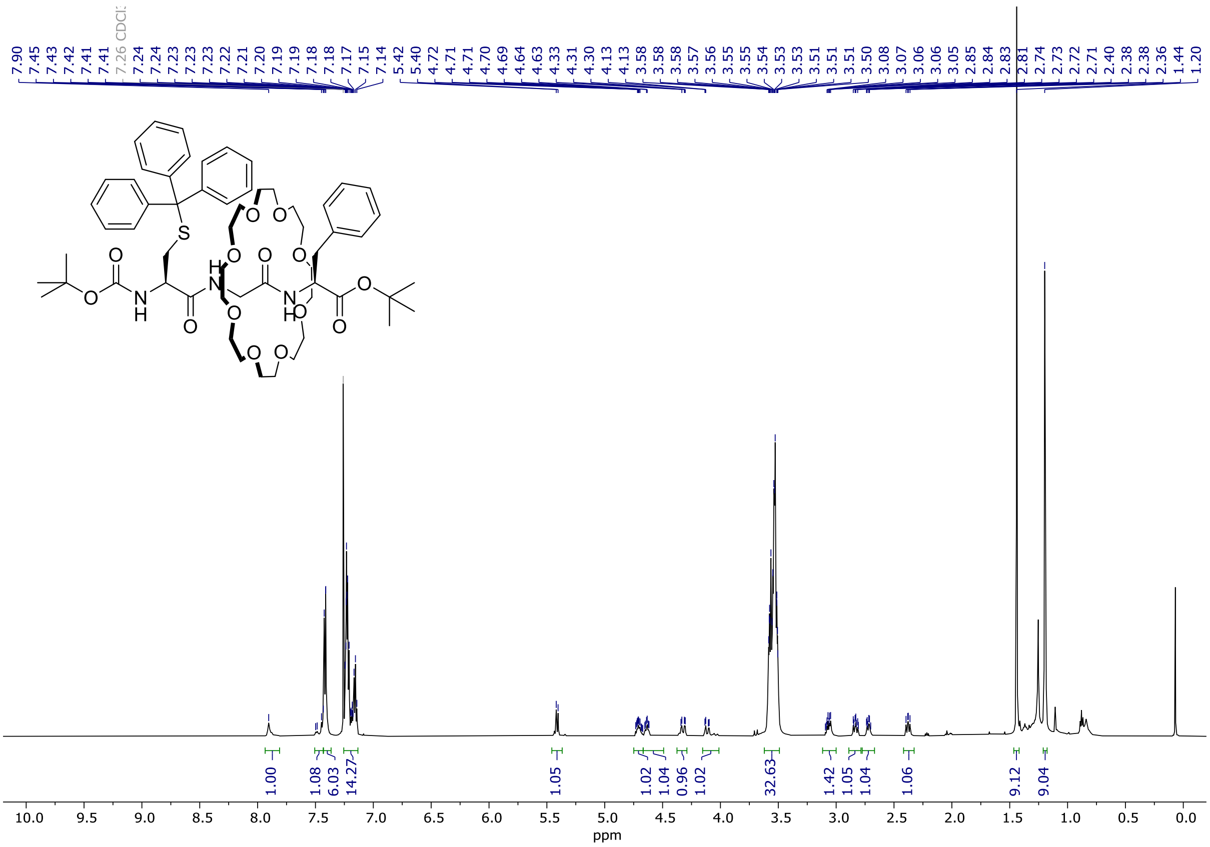

Spectrum S75: ^{1}H NMR (600 MHz, $CDCl_3$) spectrum of **24C8⊂15**.

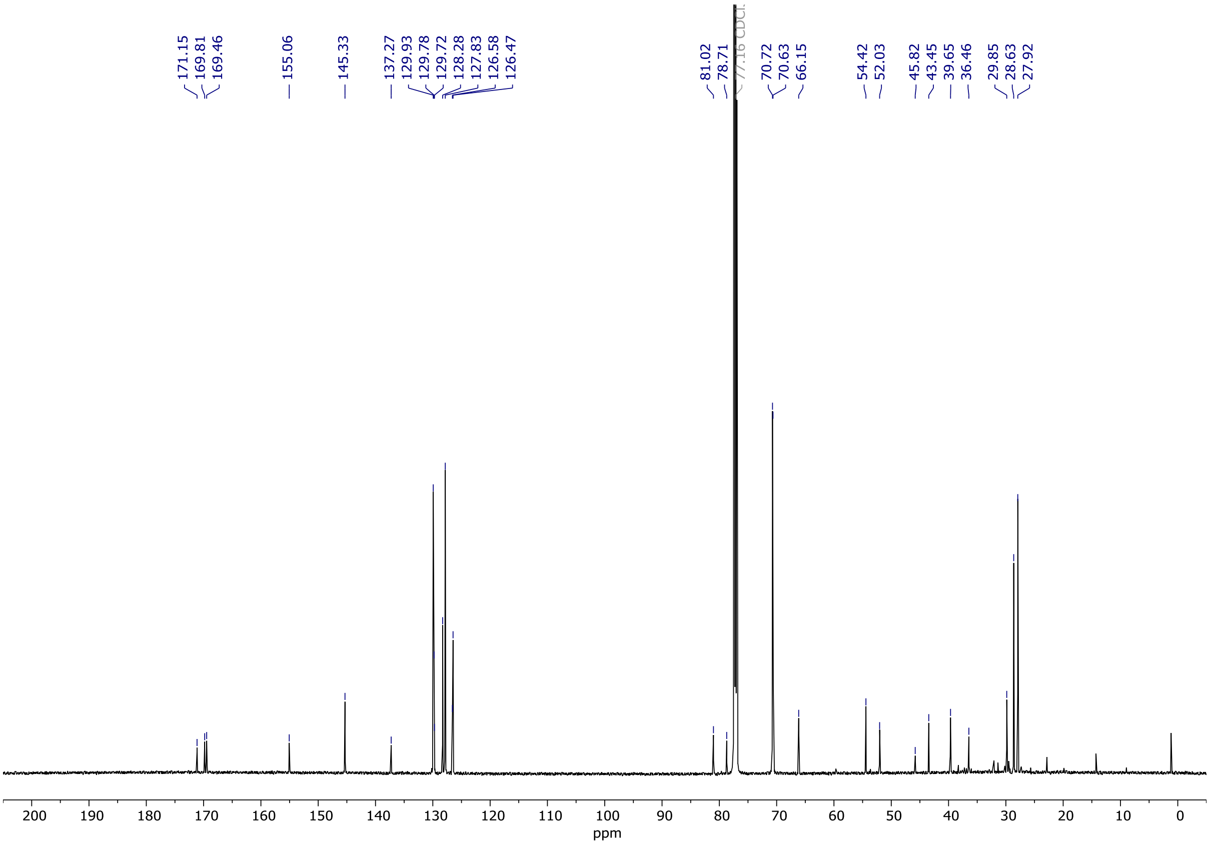

Spectrum S76: ^{13}C NMR (151 MHz, $CDCl_3$) spectrum of **24C8⊂15**.

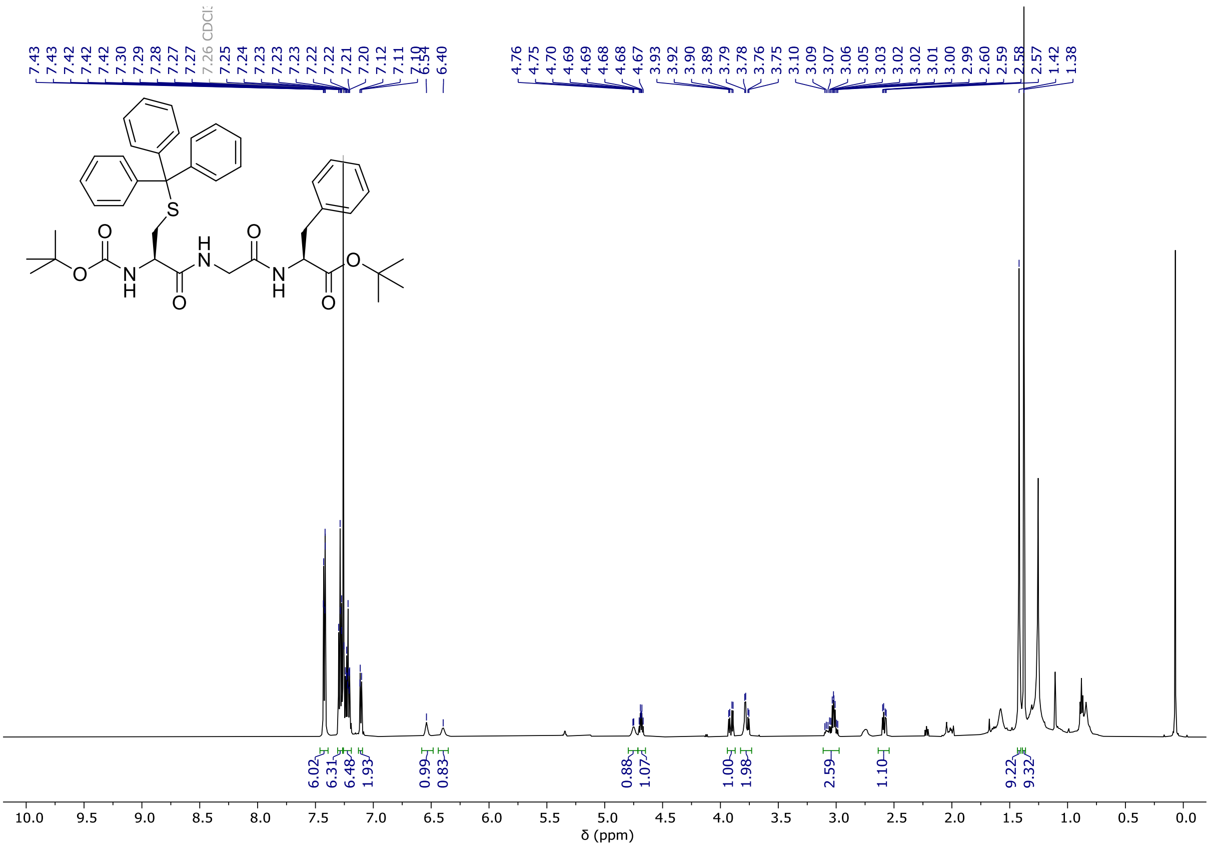

Spectrum S77: ^{1}H NMR (600 MHz, $CDCl_3$) spectrum of **15**.

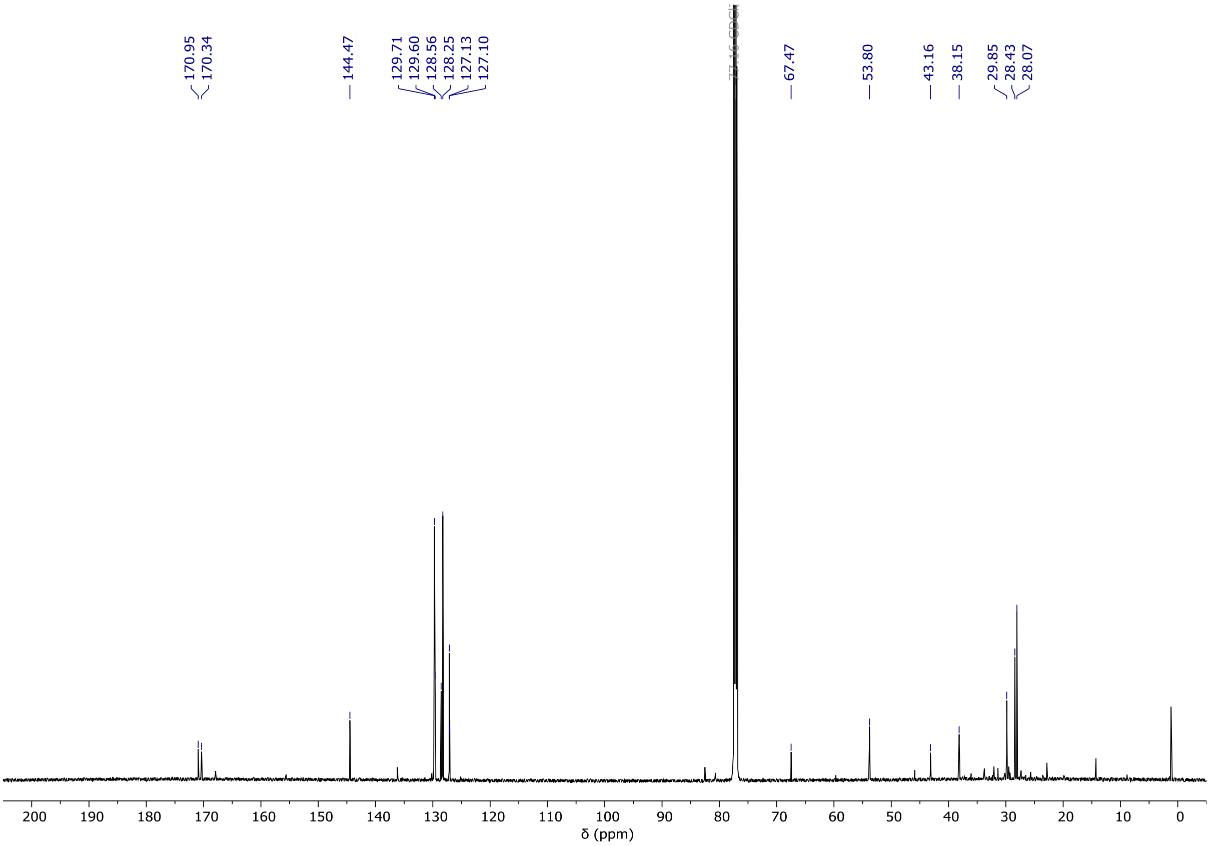

Spectrum S78: ^{13}C NMR (151 MHz, $CDCl_3$) spectrum of **15**.

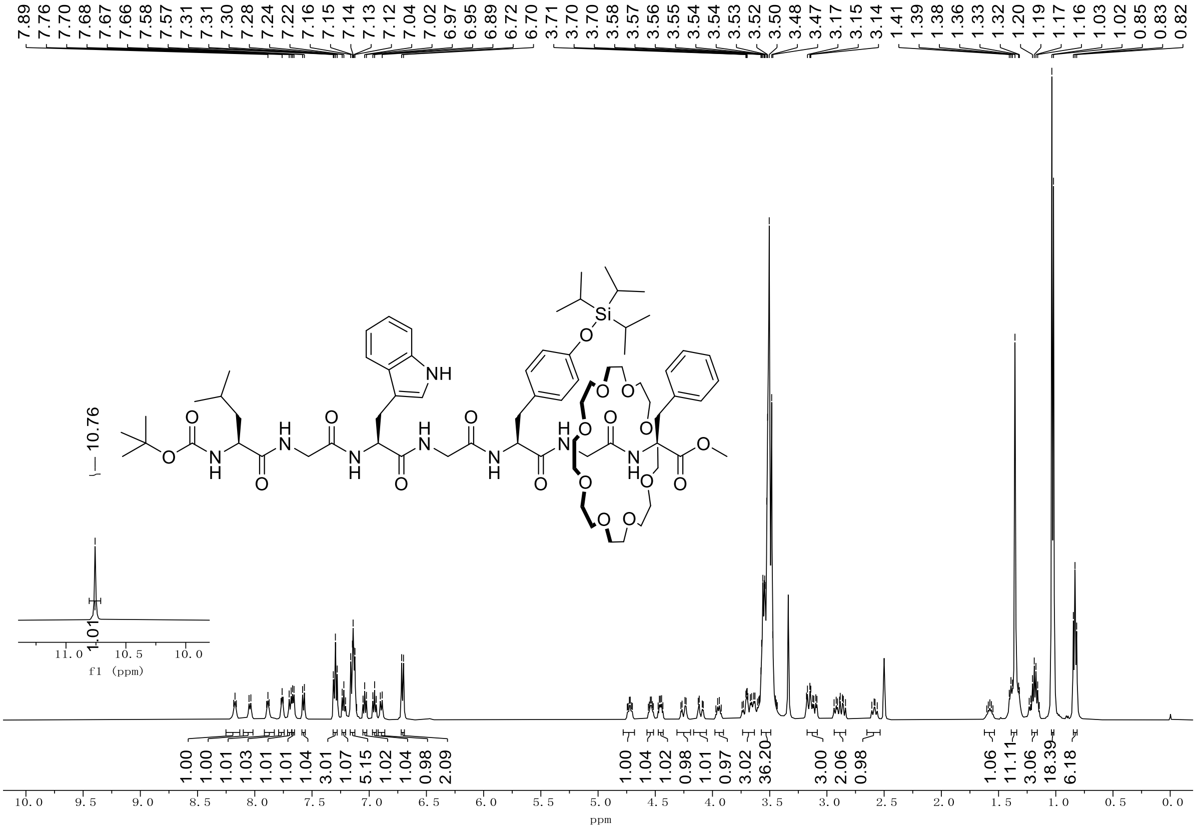

Spectrum S79: ^{1}H NMR (500 MHz, DMSO-d_6) spectrum of *0:0:1*-**24C8⊂16**.

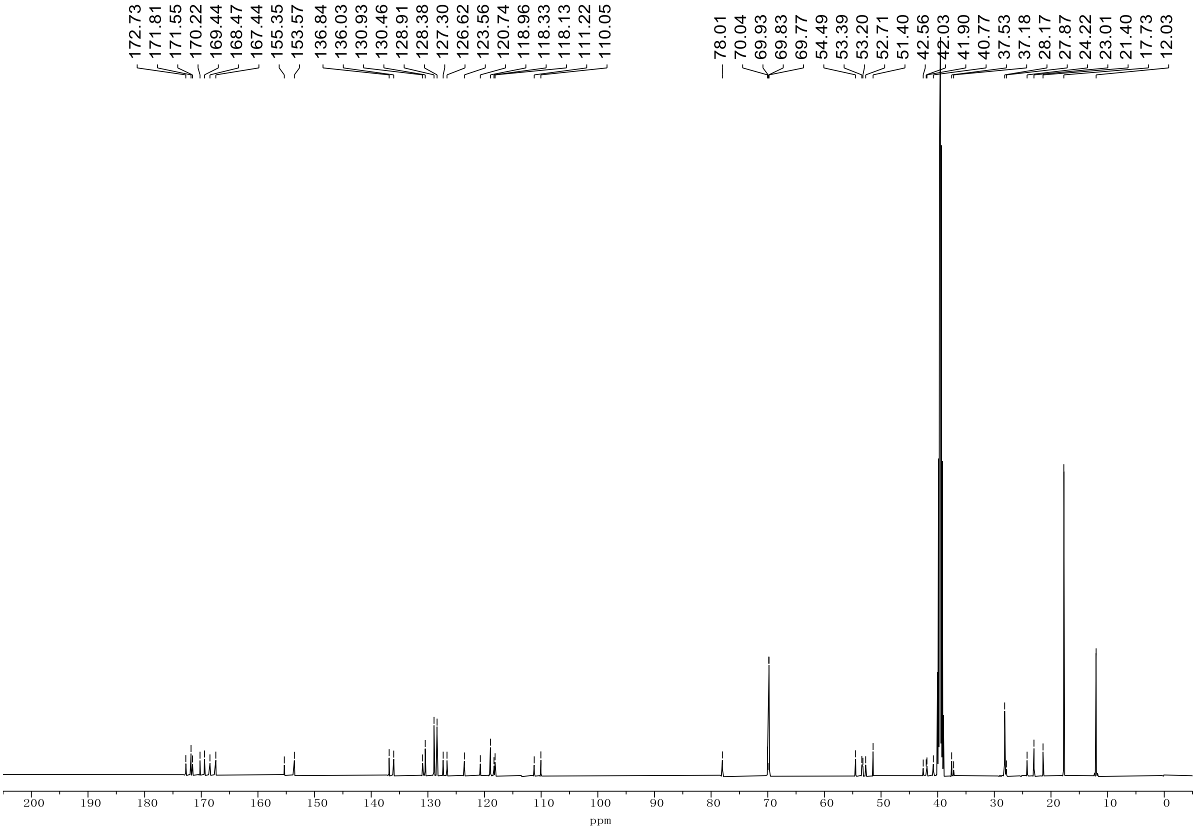

Spectrum S80: ^{13}C NMR (125 MHz, DMSO-d_6) spectrum of *0:0:1*-**24C8⊂16**.

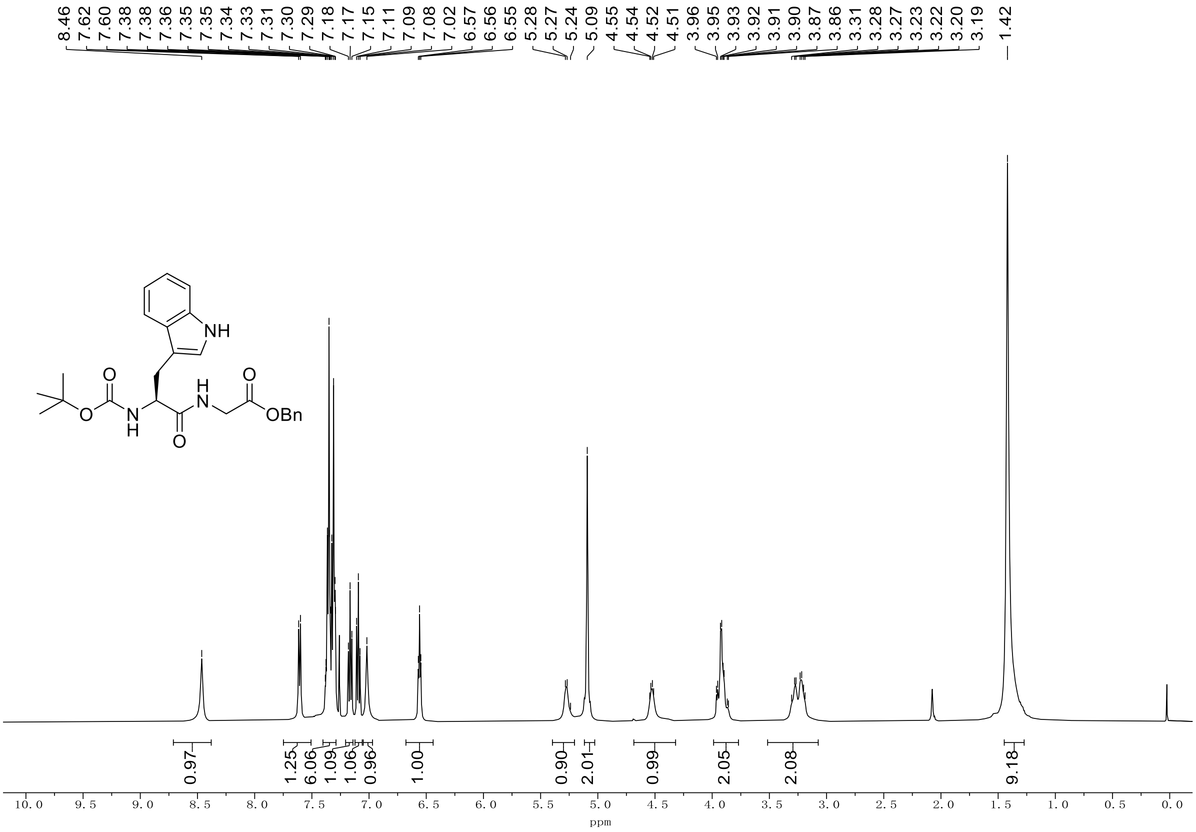

Spectrum S81: ^{1}H NMR (500 MHz, $CDCl_3$) spectrum of **S5**.

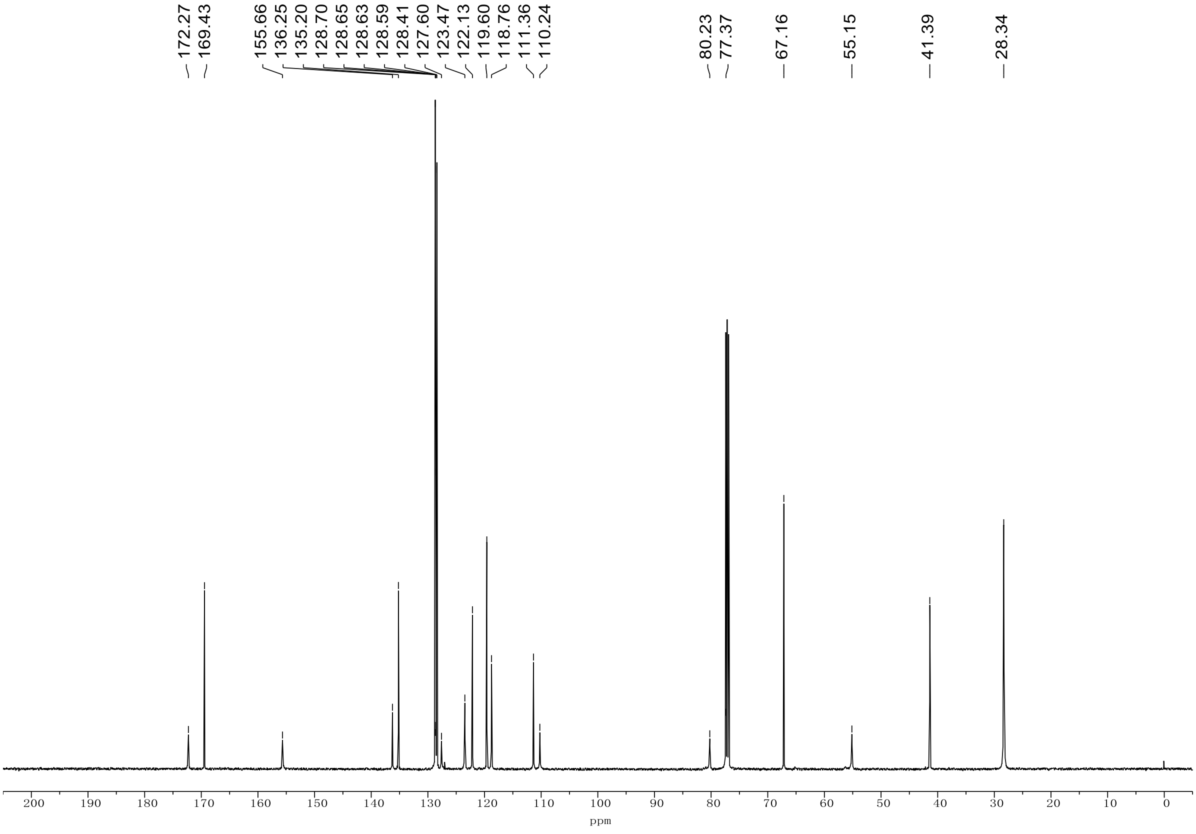

Spectrum S82: ^{13}C NMR (125 MHz, $CDCl_3$) spectrum of **S5**.

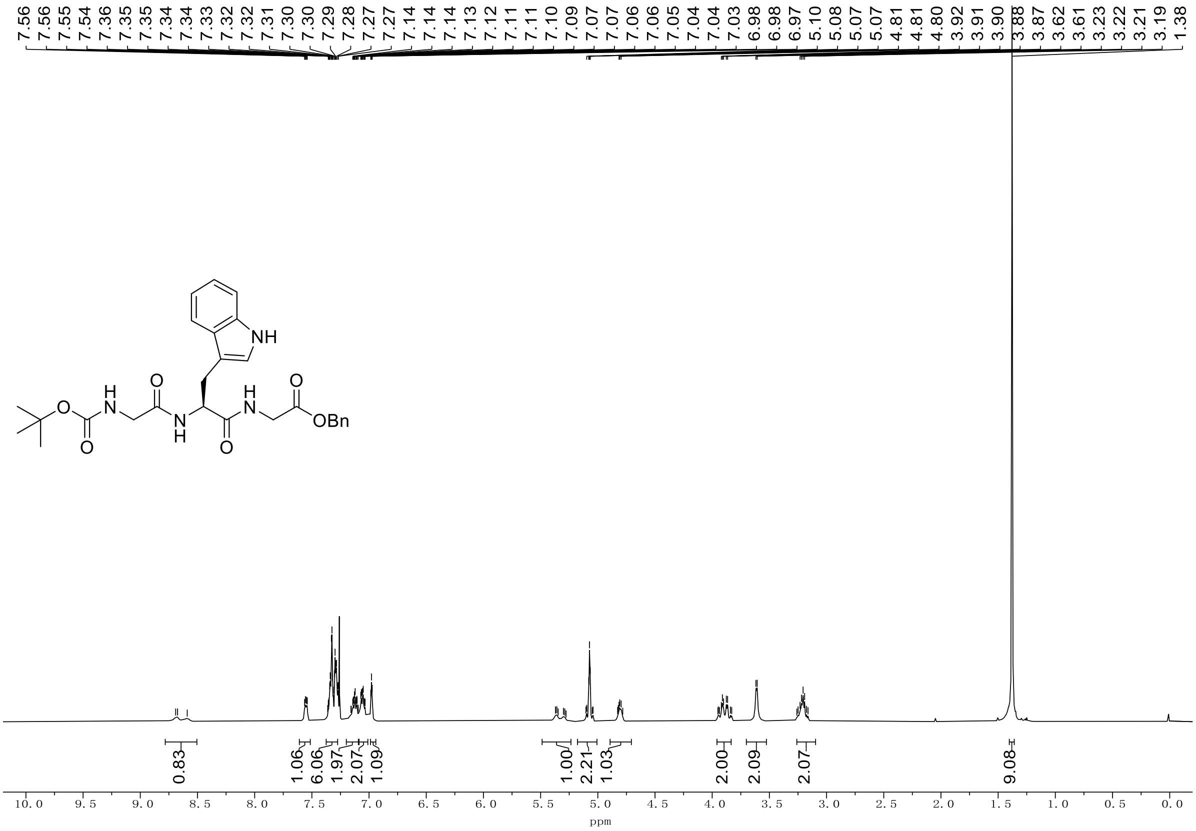

Spectrum S83: ^{1}H NMR (500 MHz, $CDCl_3$) spectrum of **S6**.

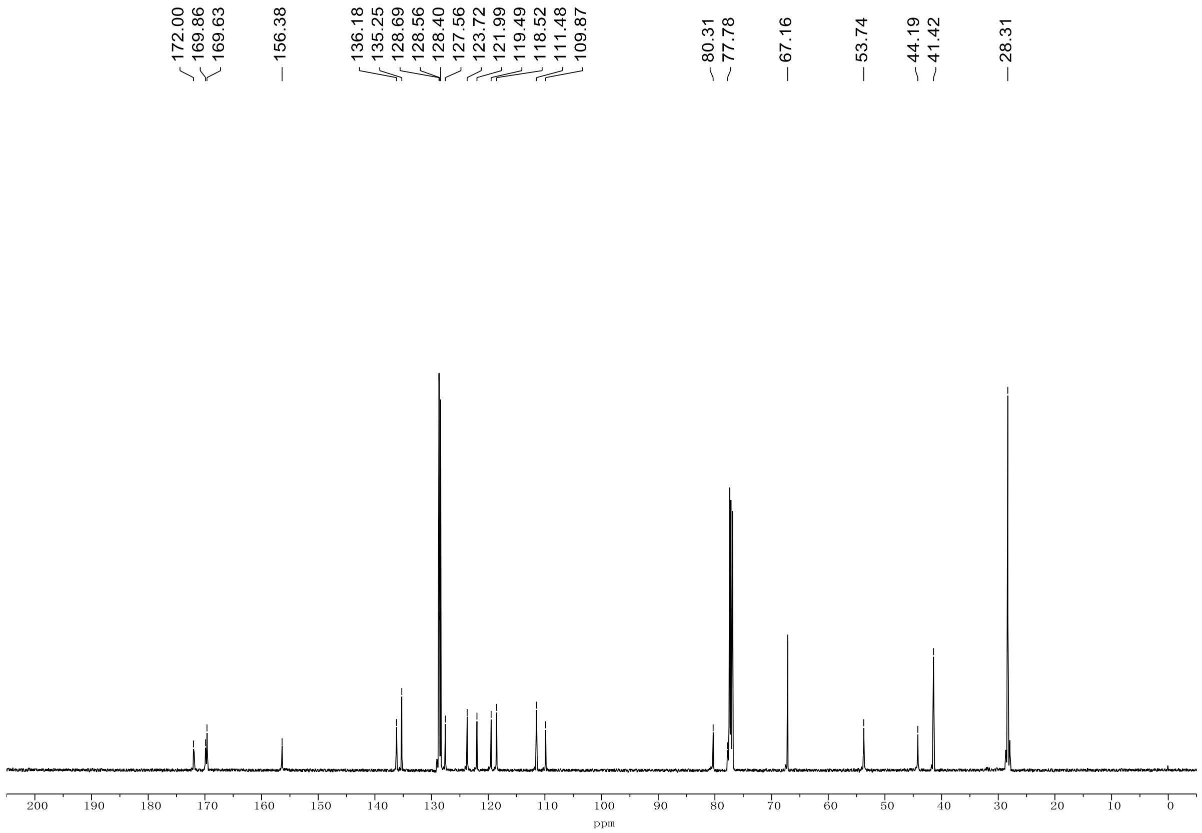

Spectrum S84: ^{13}C NMR (125 MHz, $CDCl_3$) spectrum of **S6**.

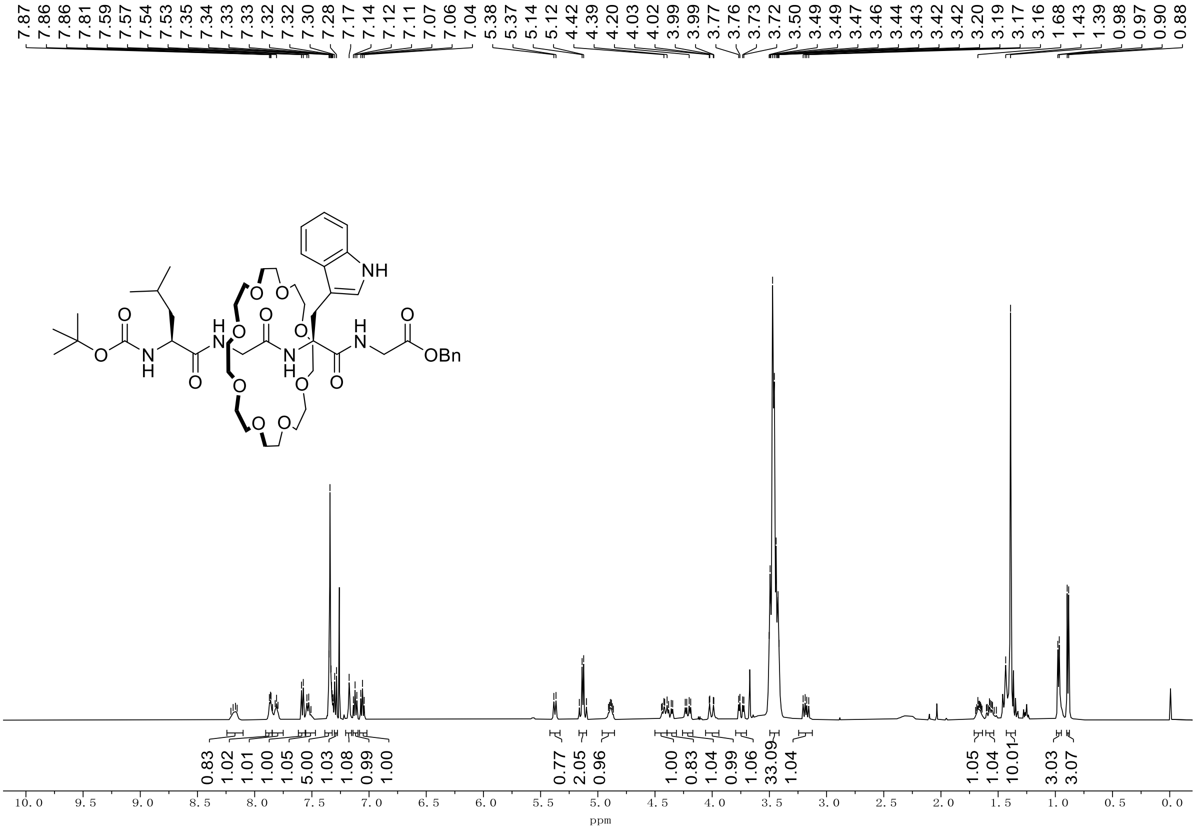

Spectrum S85: ^{1}H NMR (500 MHz, $CDCl_3$) spectrum of **24C8⊂17**.

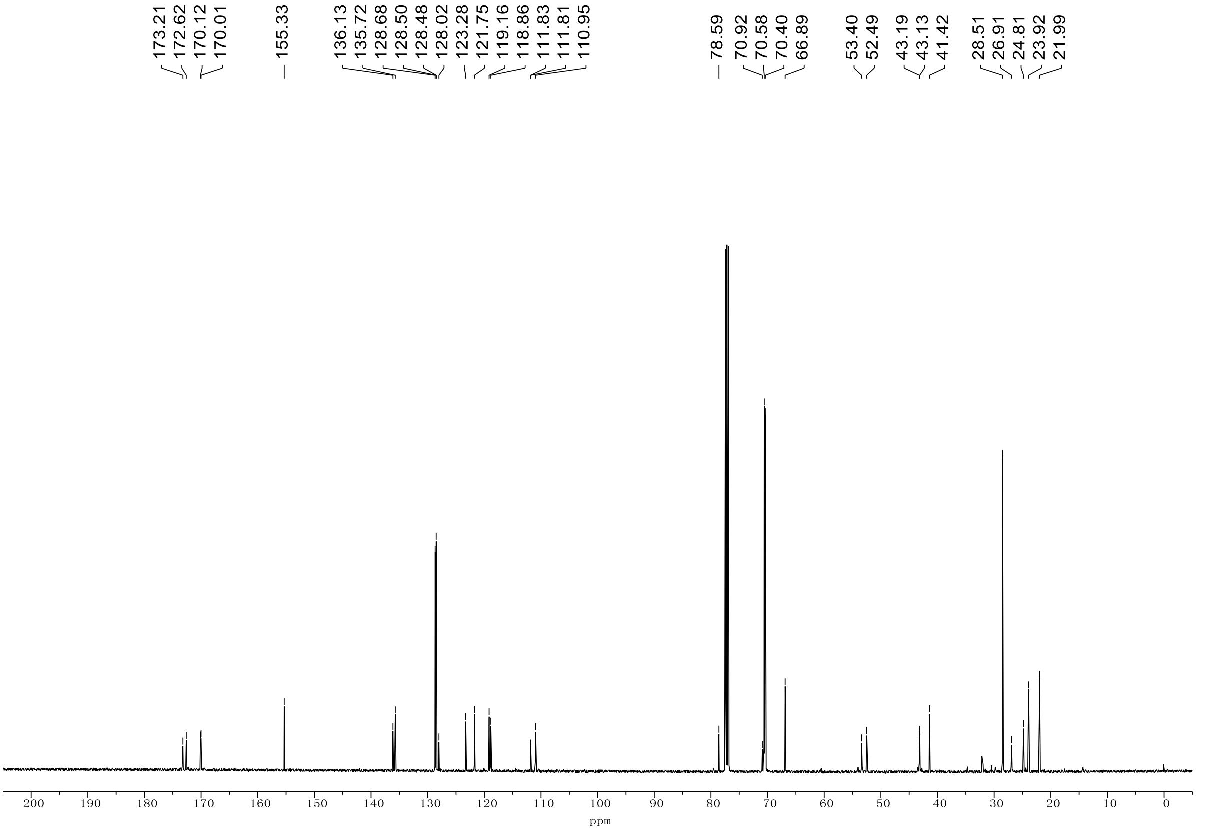

Spectrum S86: ^{13}C NMR (125 MHz, $CDCl_3$) spectrum of **24C8⊂17**.

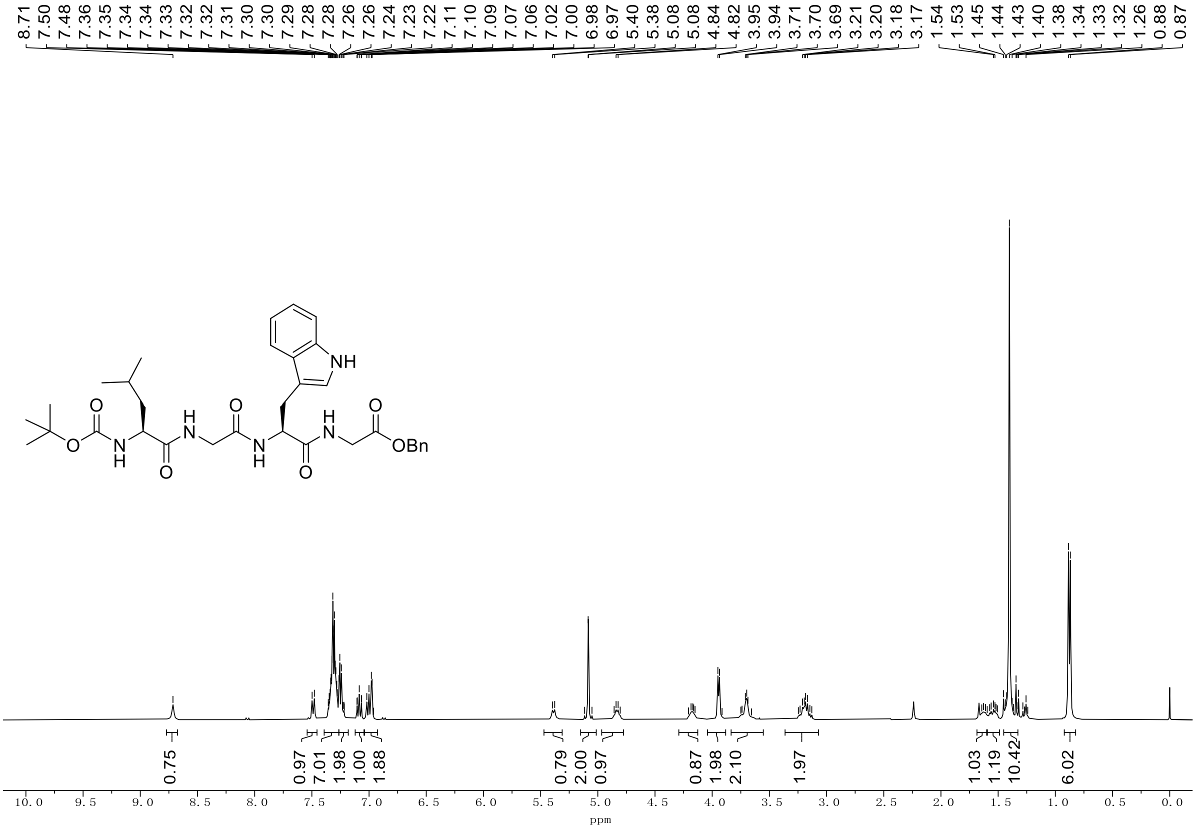

Spectrum S87: ^{1}H NMR (500 MHz, $CDCl_3$) spectrum of **17**.

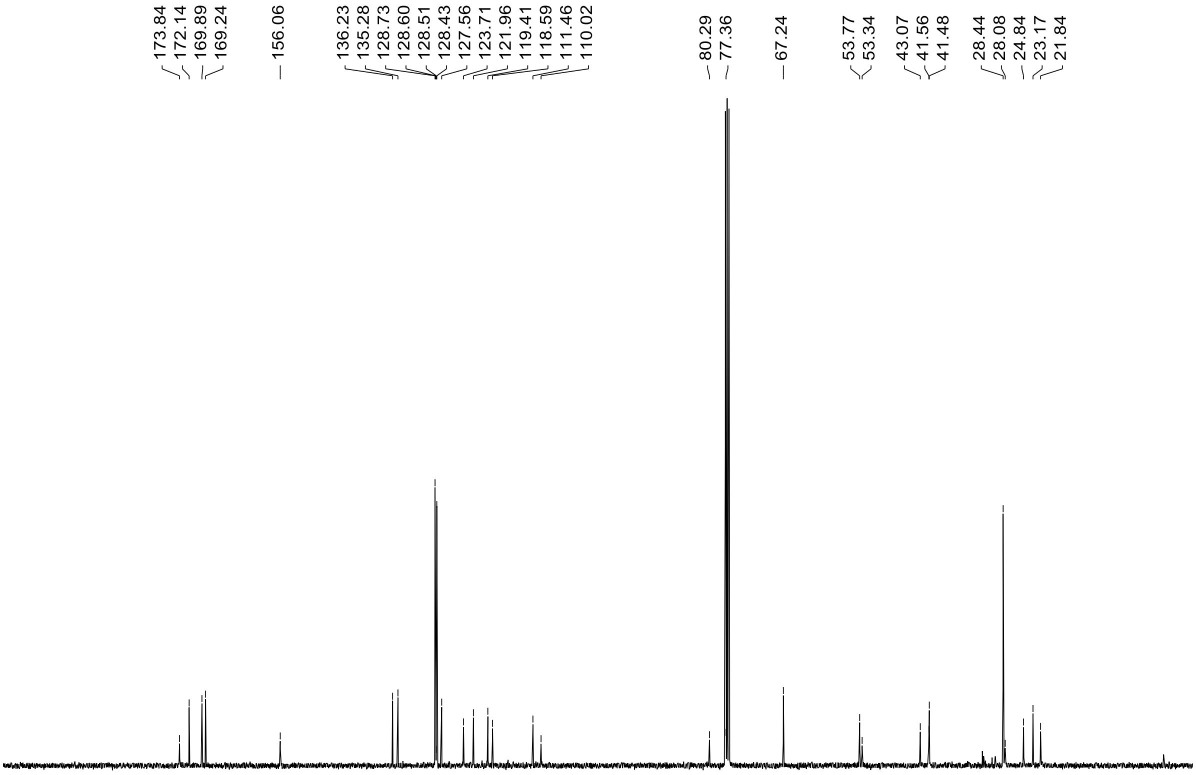

Spectrum S88: ^{13}C NMR (125 MHz, $CDCl_3$) spectrum of **17**.

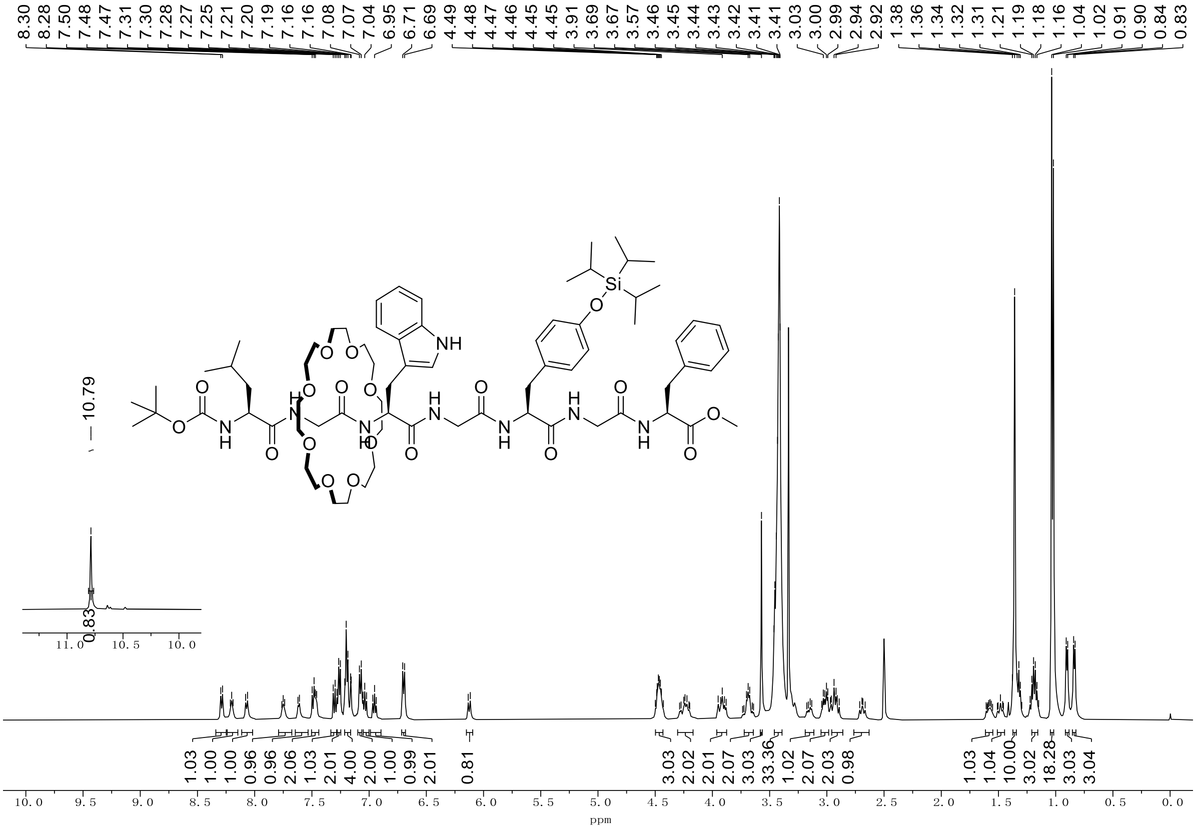

Spectrum S89: ^{1}H NMR (500 MHz, DMSO-d_6) spectrum of *1:0:0*-**24C8⊂16**.

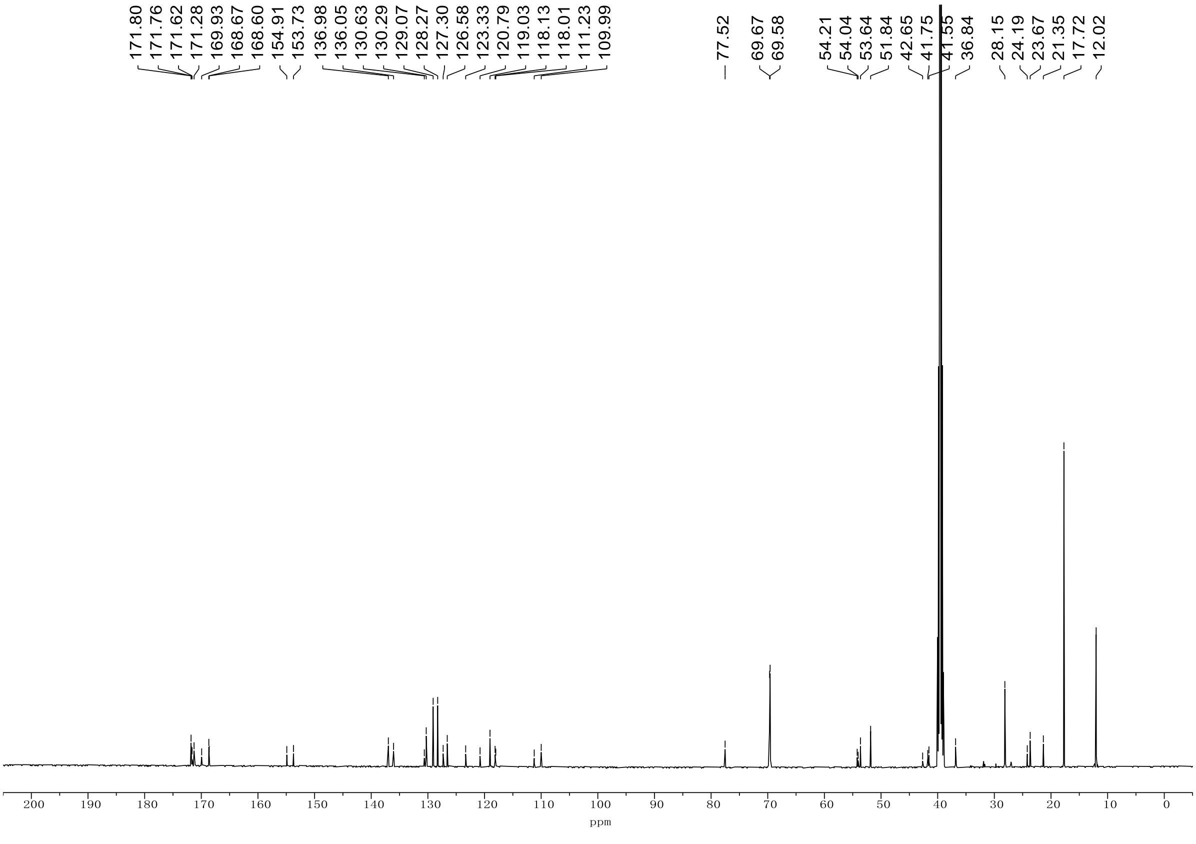

Spectrum S90: ^{13}C NMR (125 MHz, DMSO-d_6) spectrum of *1:0:0*-**24C8⊂16**.

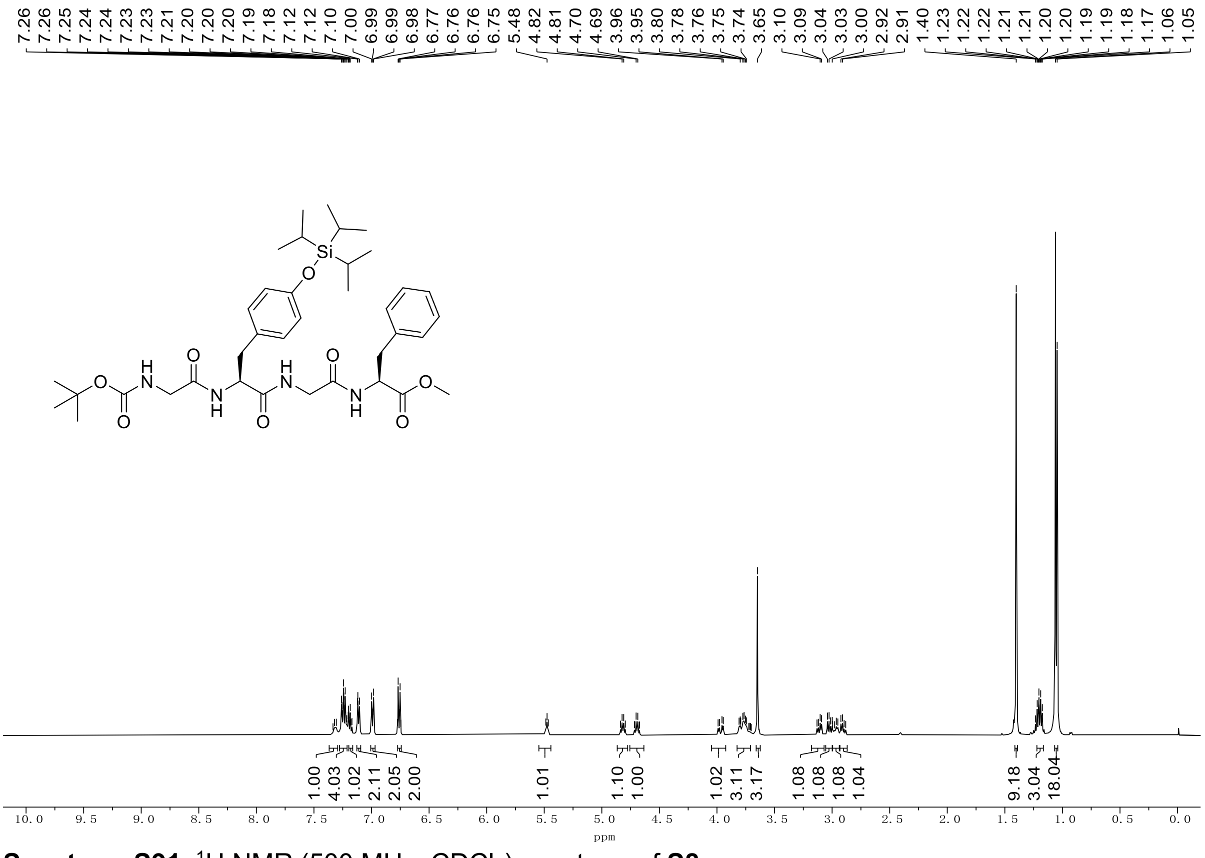

Spectrum S91: ^{1}H NMR (500 MHz, $CDCl_3$) spectrum of **S8**.

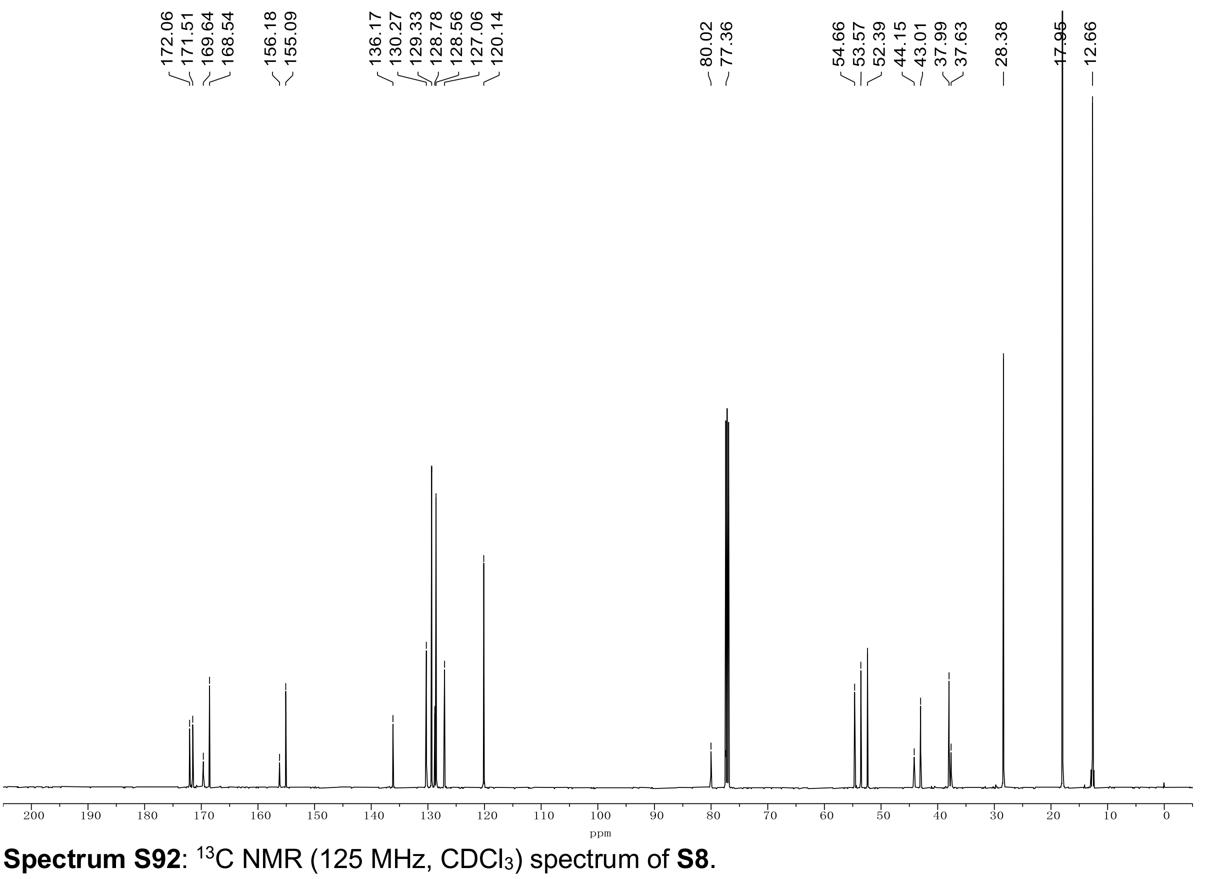

Spectrum S92: ^{13}C NMR (125 MHz, $CDCl_3$) spectrum of **S8**.

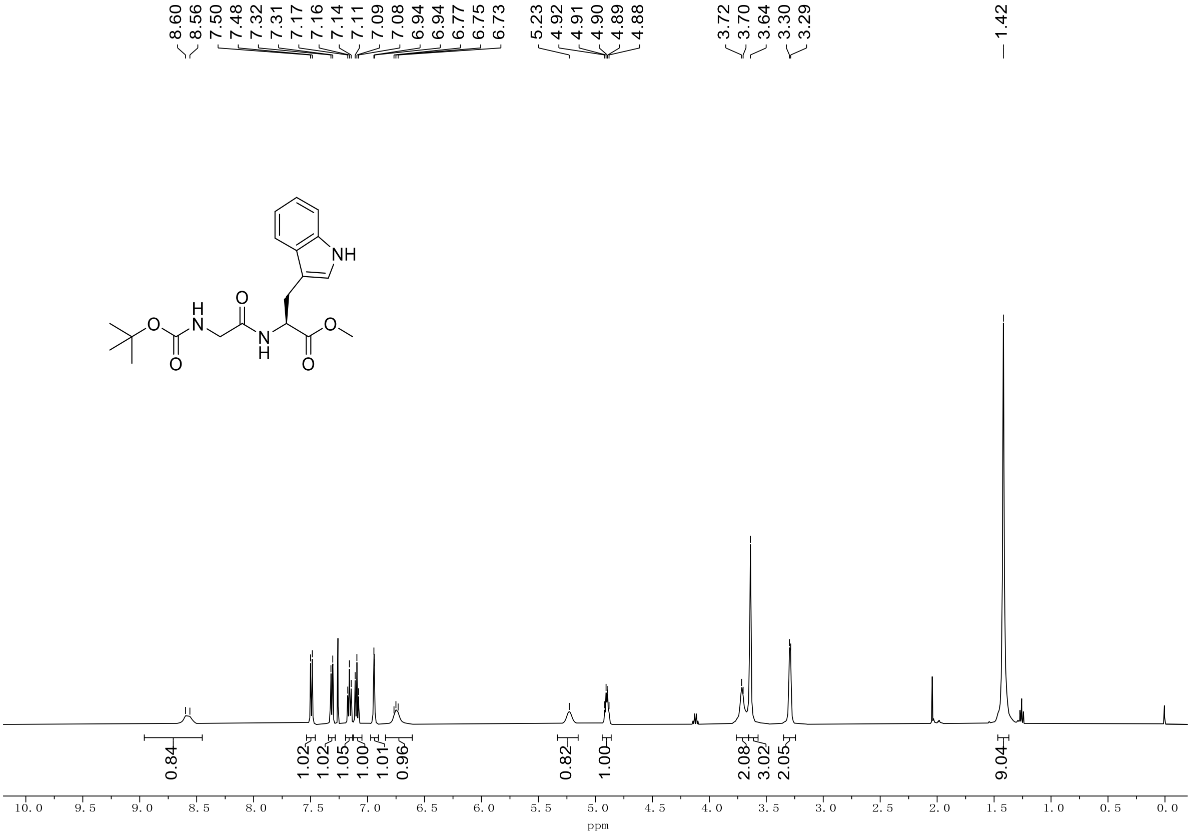

Spectrum S93: ^{1}H NMR (500 MHz, $CDCl_3$) spectrum of **S9**.

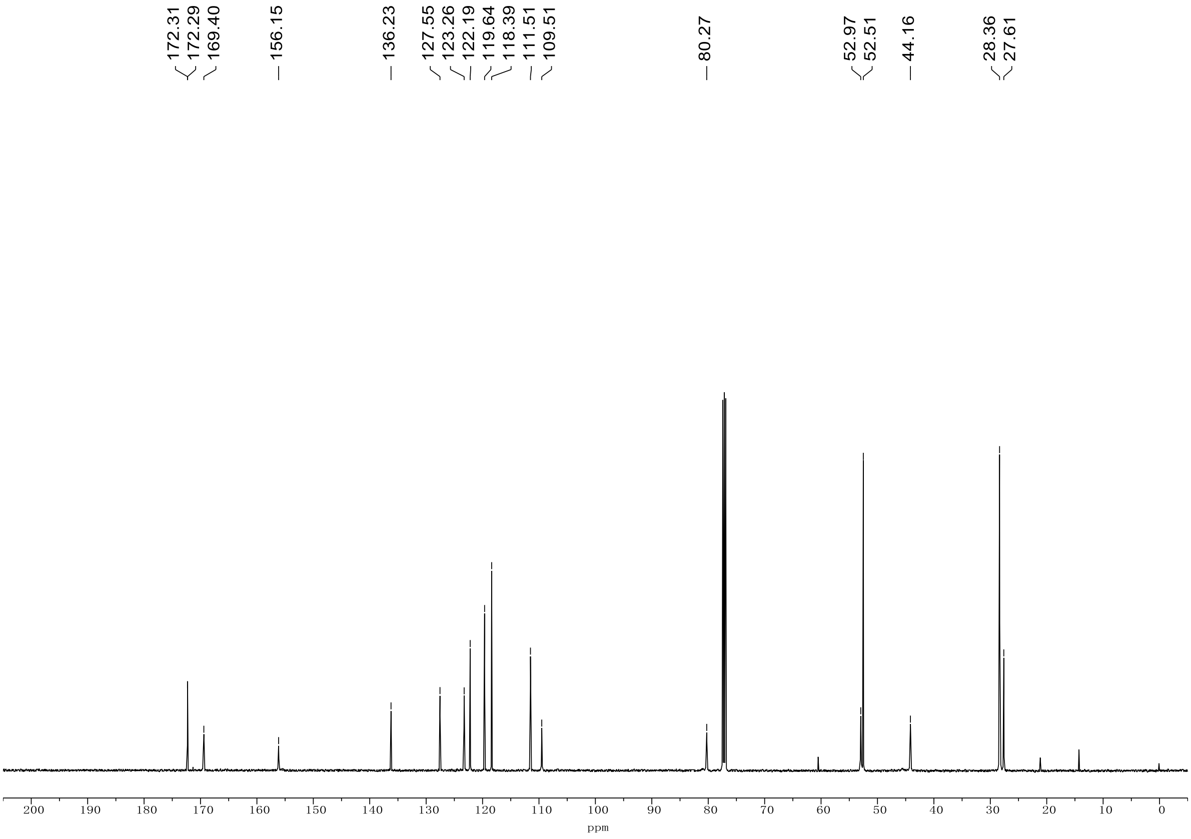

Spectrum S94: ^{13}C NMR (125 MHz, $CDCl_3$) spectrum of **S9**.

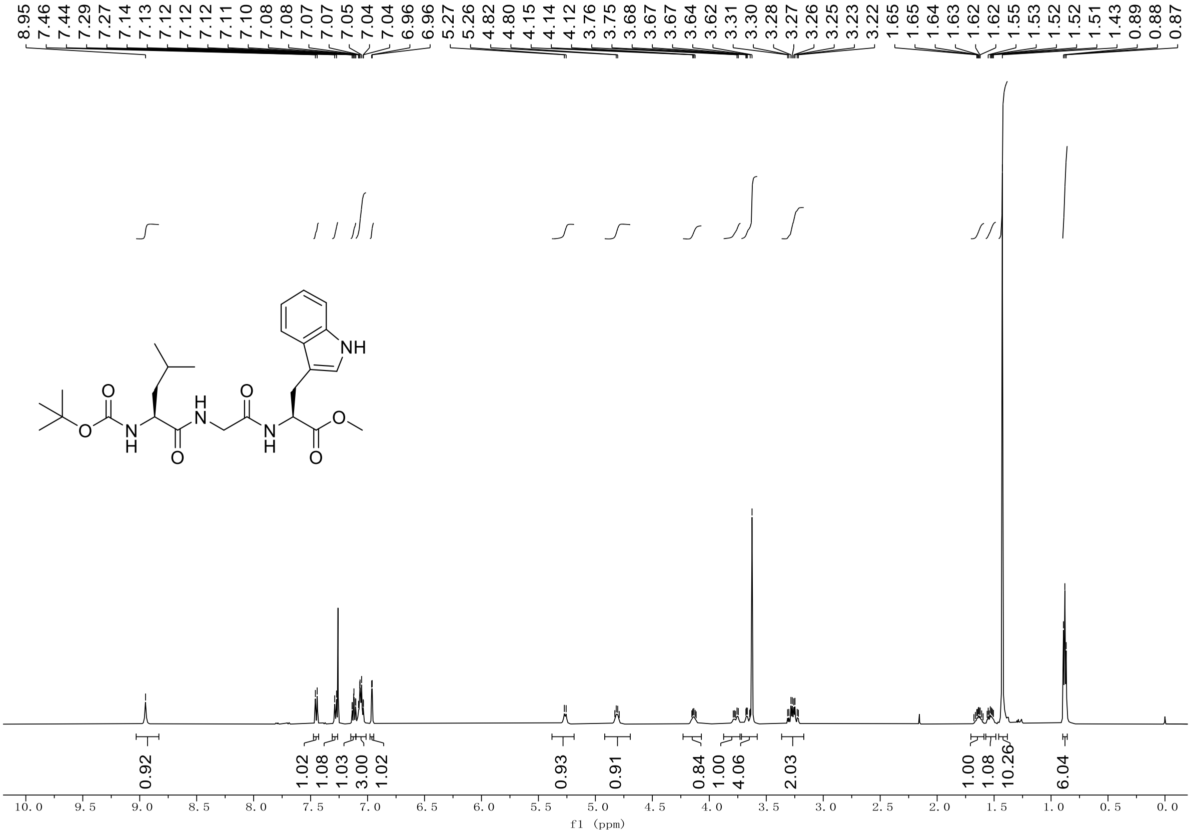

Spectrum S95: ^{1}H NMR (500 MHz, $CDCl_3$) spectrum of **S10**.

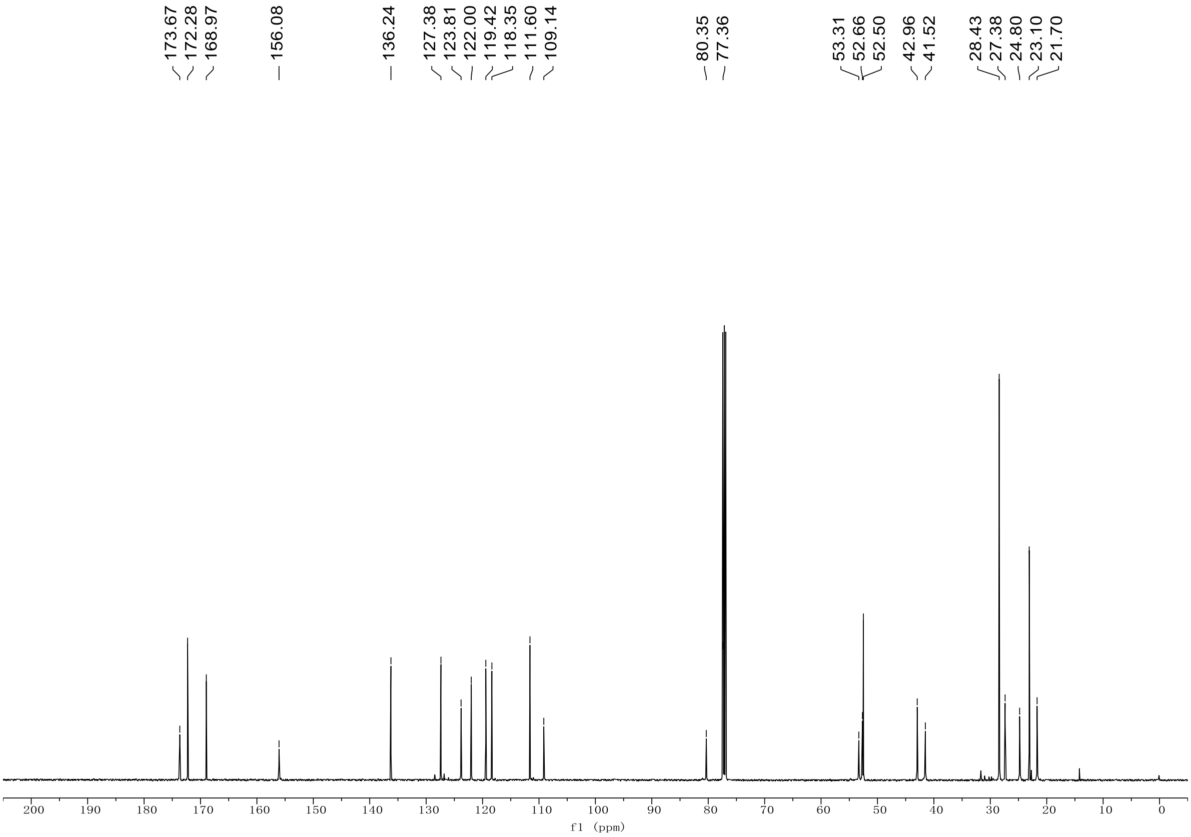

Spectrum S96: ^{13}C NMR (125 MHz, $CDCl_3$) spectrum of **S10**.

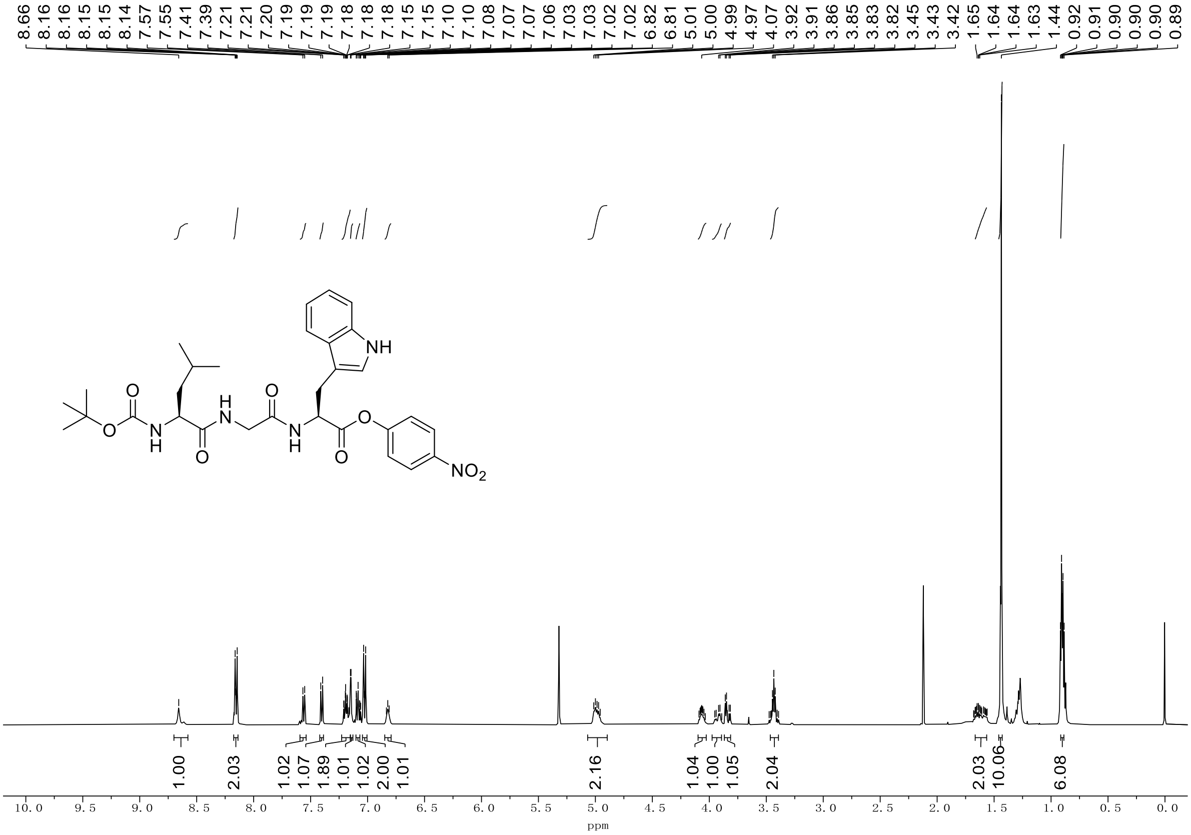

Spectrum S97: ^{1}H NMR (500 MHz, CD_2Cl_2) spectrum of **19**.

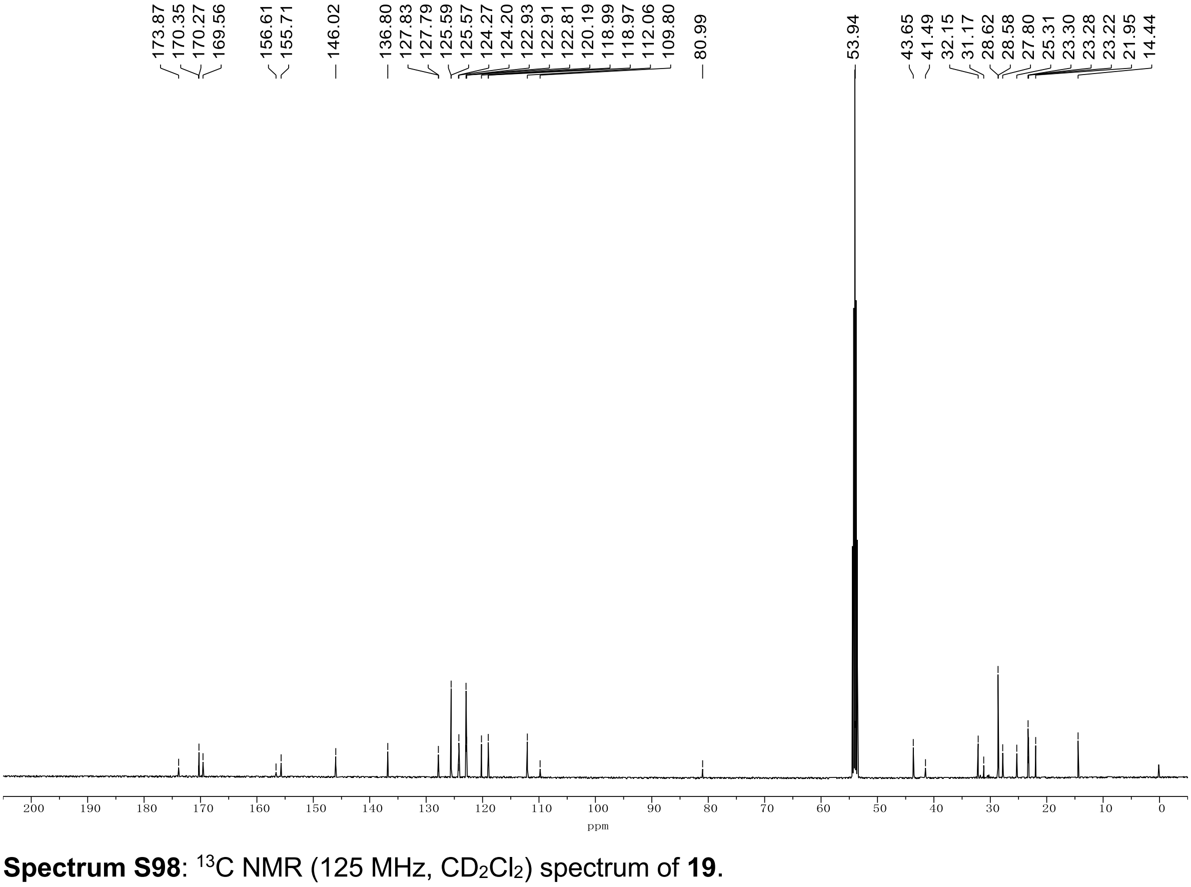

Spectrum S98: ^{13}C NMR (125 MHz, CD_2Cl_2) spectrum of **19**.

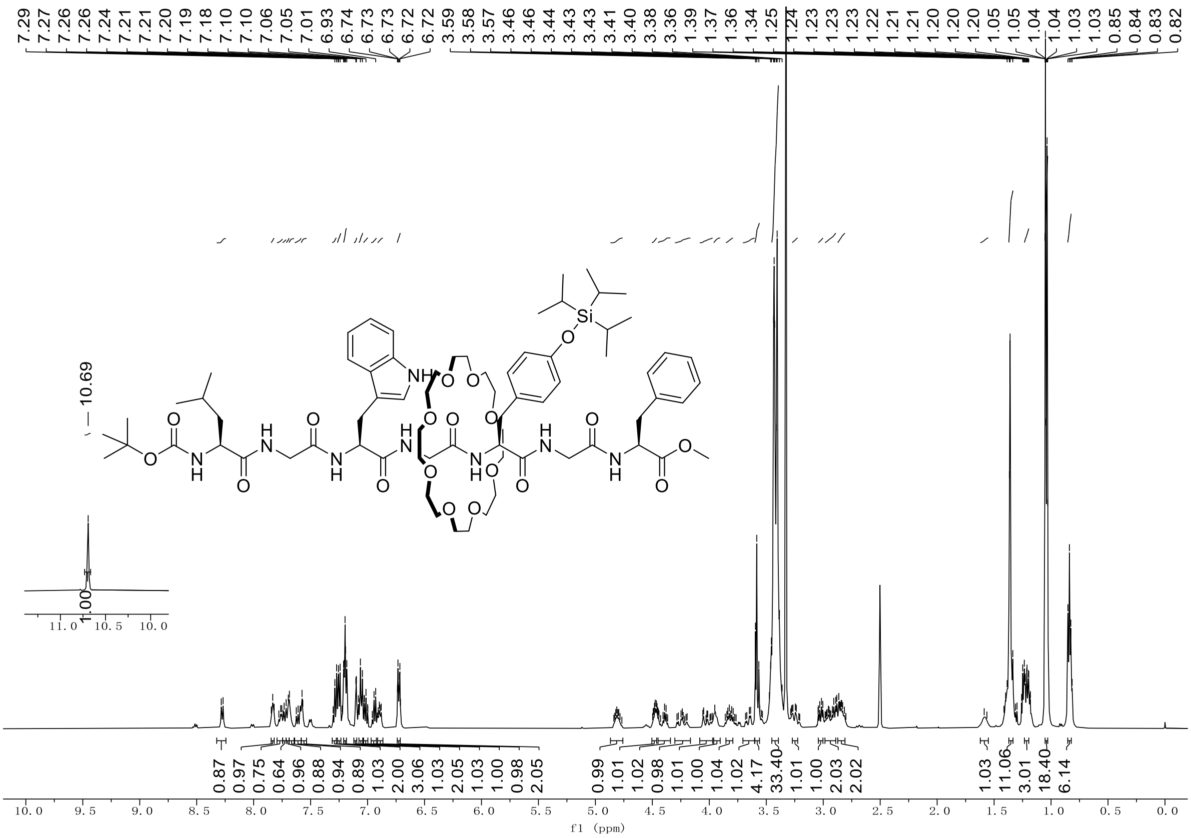

Spectrum S99: ^{1}H NMR (500 MHz, DMSO-d_6) spectrum of *0:1:0*-**24C8⊂16**.

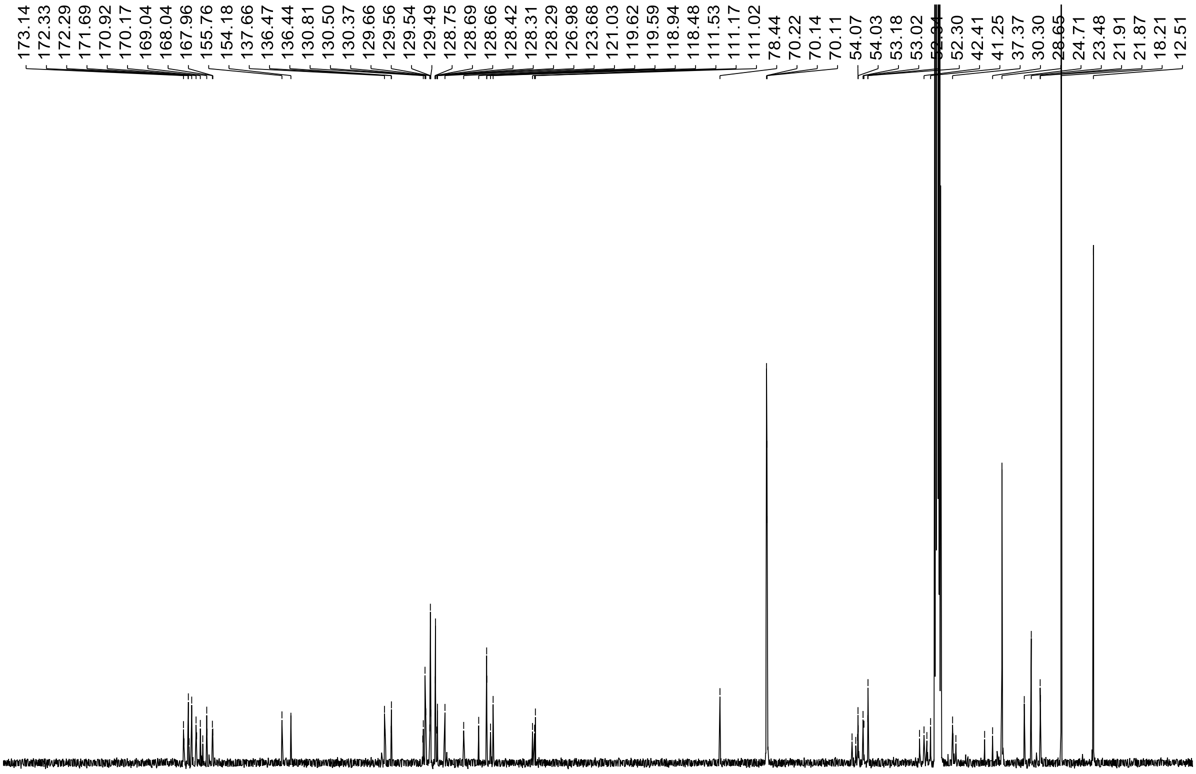

Spectrum S100: ^{13}C NMR (125 MHz, DMSO-d_6) spectrum of *0:1:0*-**24C8⊂16**.

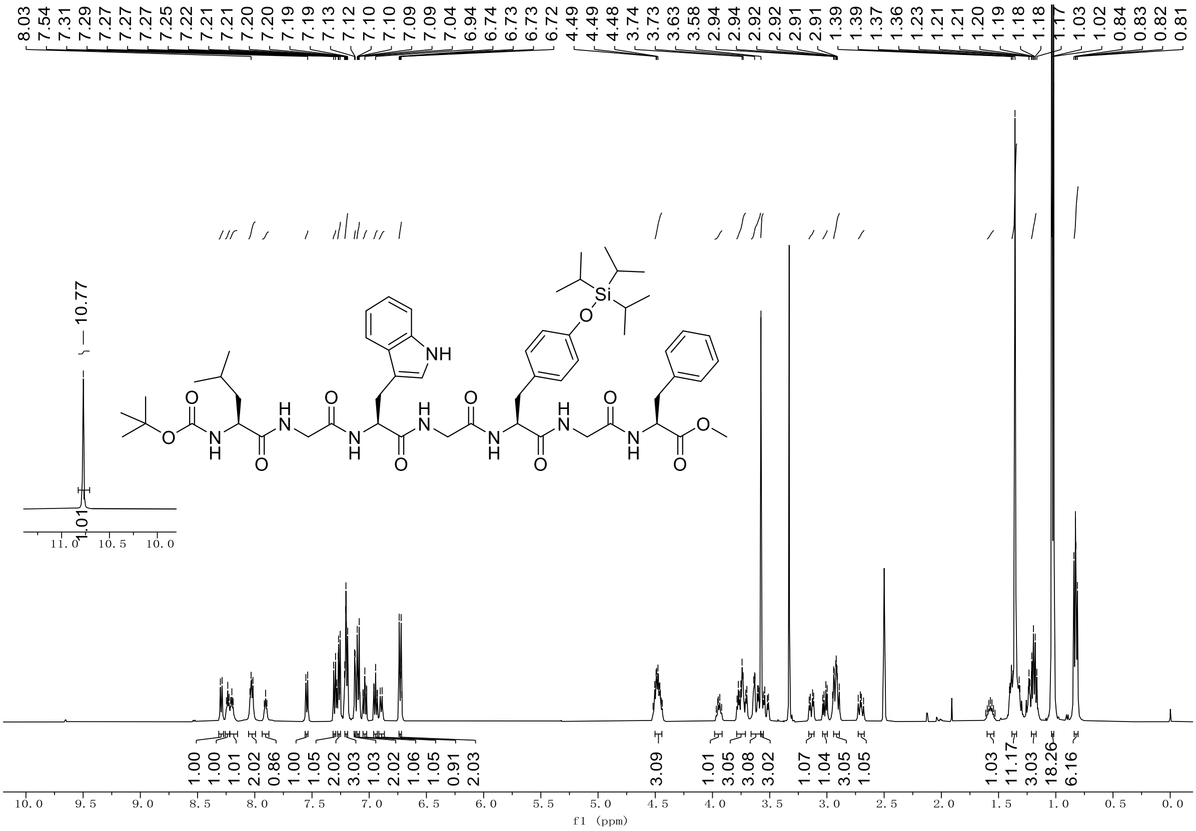

Spectrum S101: ^{1}H NMR (500 MHz, DMSO-d_6) spectrum of **16**.

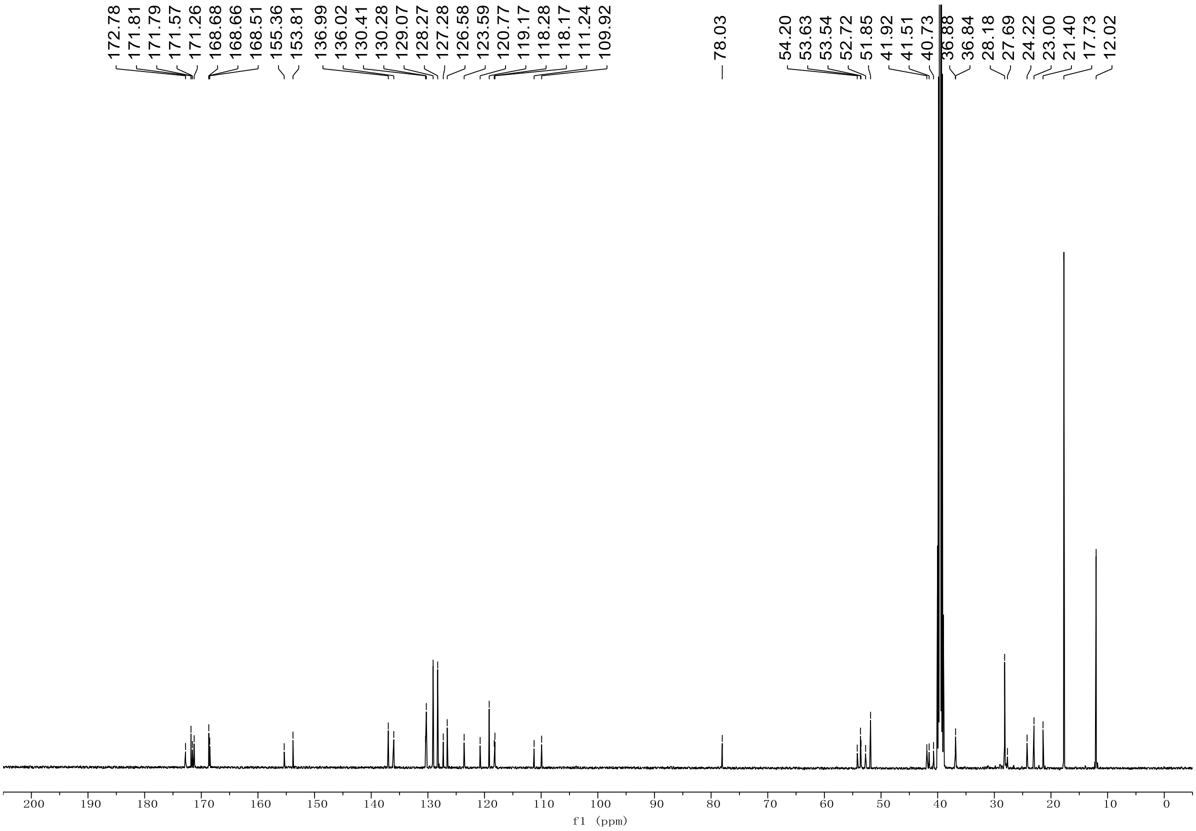

Spectrum S102: ^{13}C NMR (125 MHz, DMSO-d_6) spectrum of **16**.

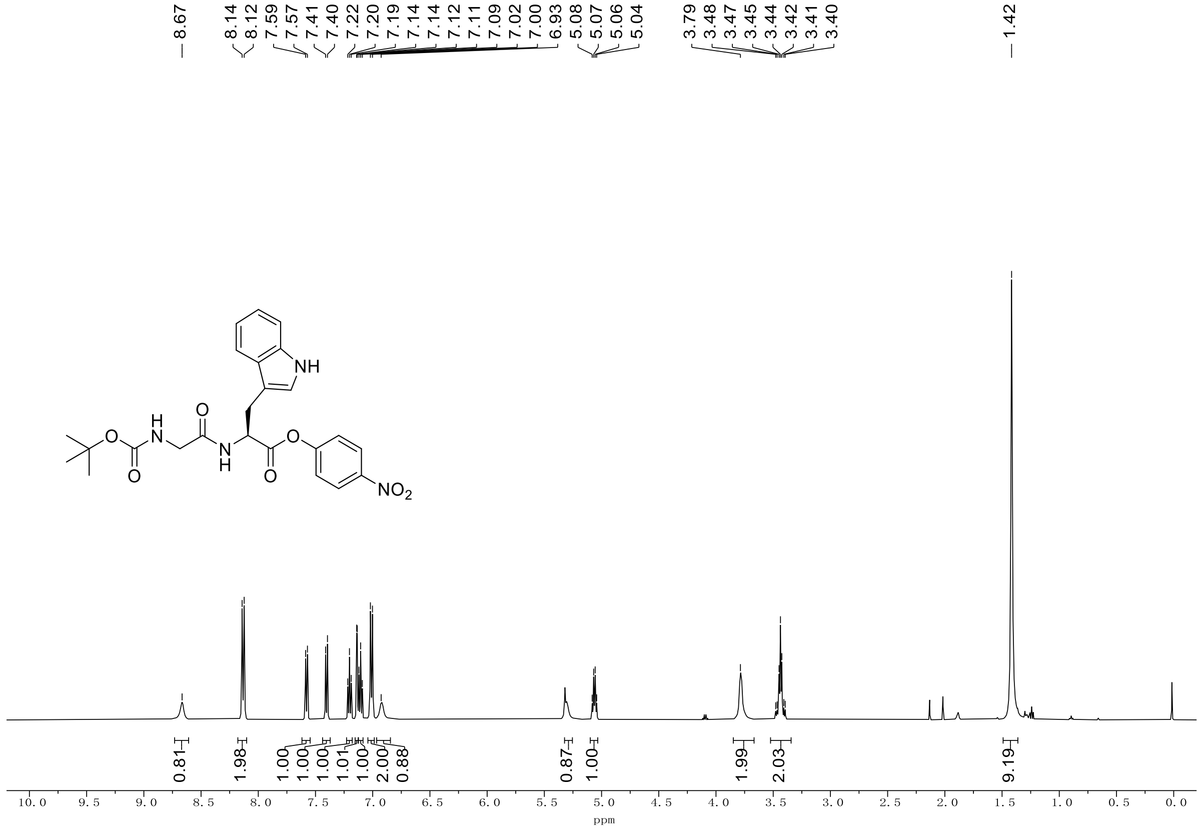

Spectrum S103: ^{1}H NMR (500 MHz, CD_2Cl_2) spectrum of **S11**.

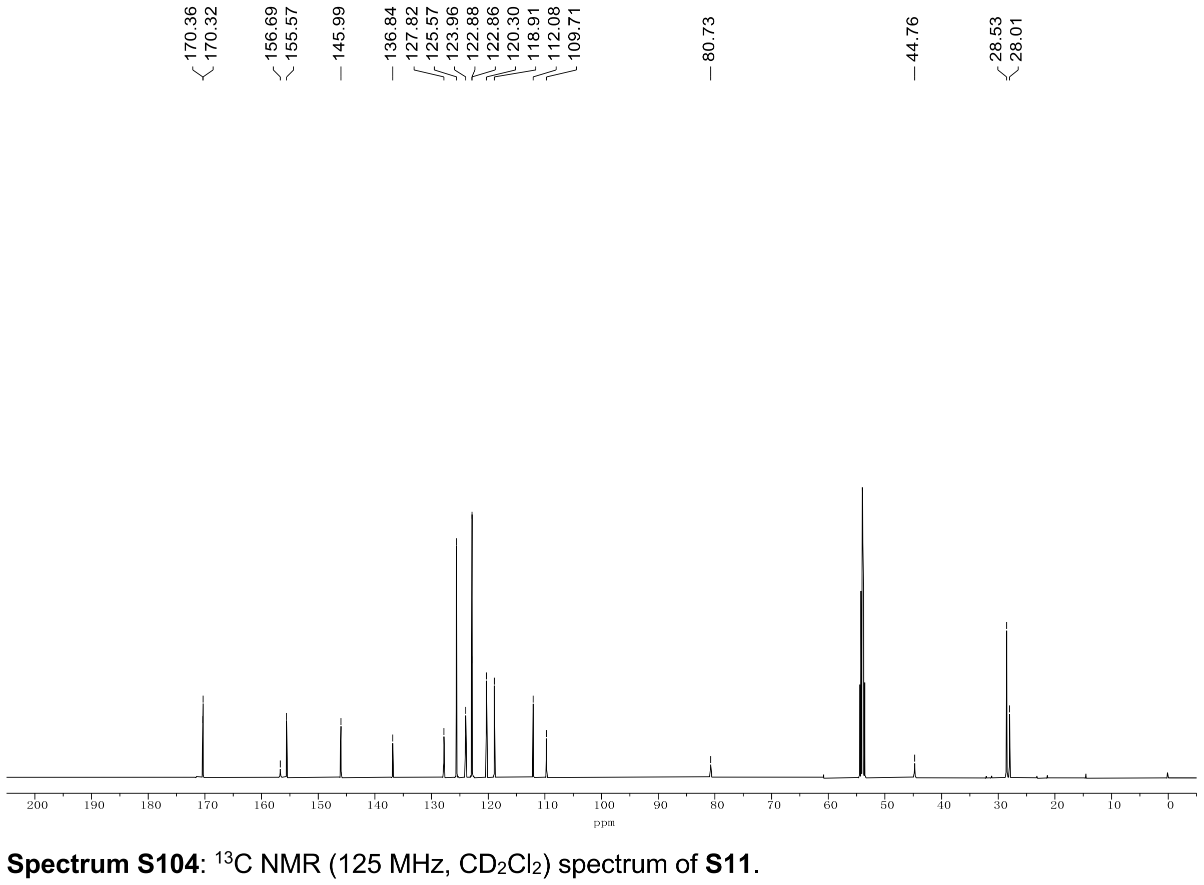

Spectrum S104: ^{13}C NMR (125 MHz, CD_2Cl_2) spectrum of **S11**.

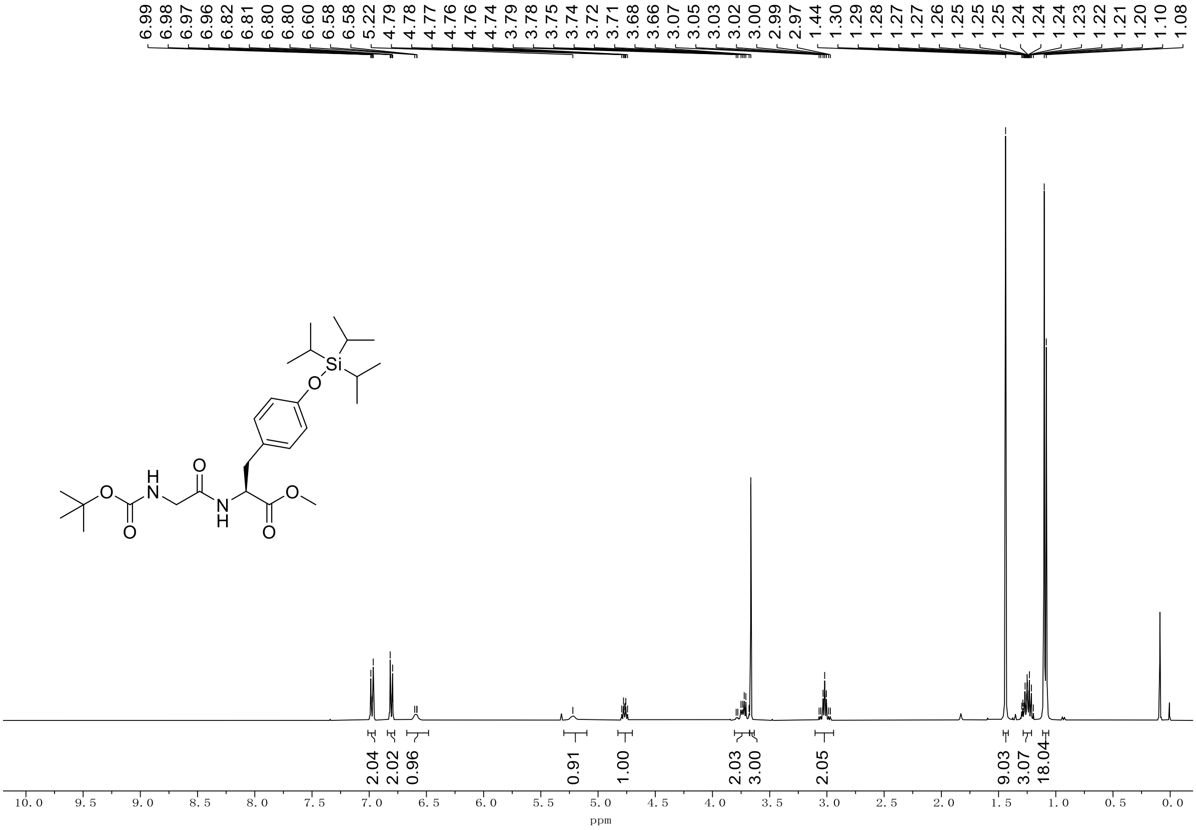

Spectrum S105: ^{1}H NMR (500 MHz, CD_2Cl_2) spectrum of **S12**.

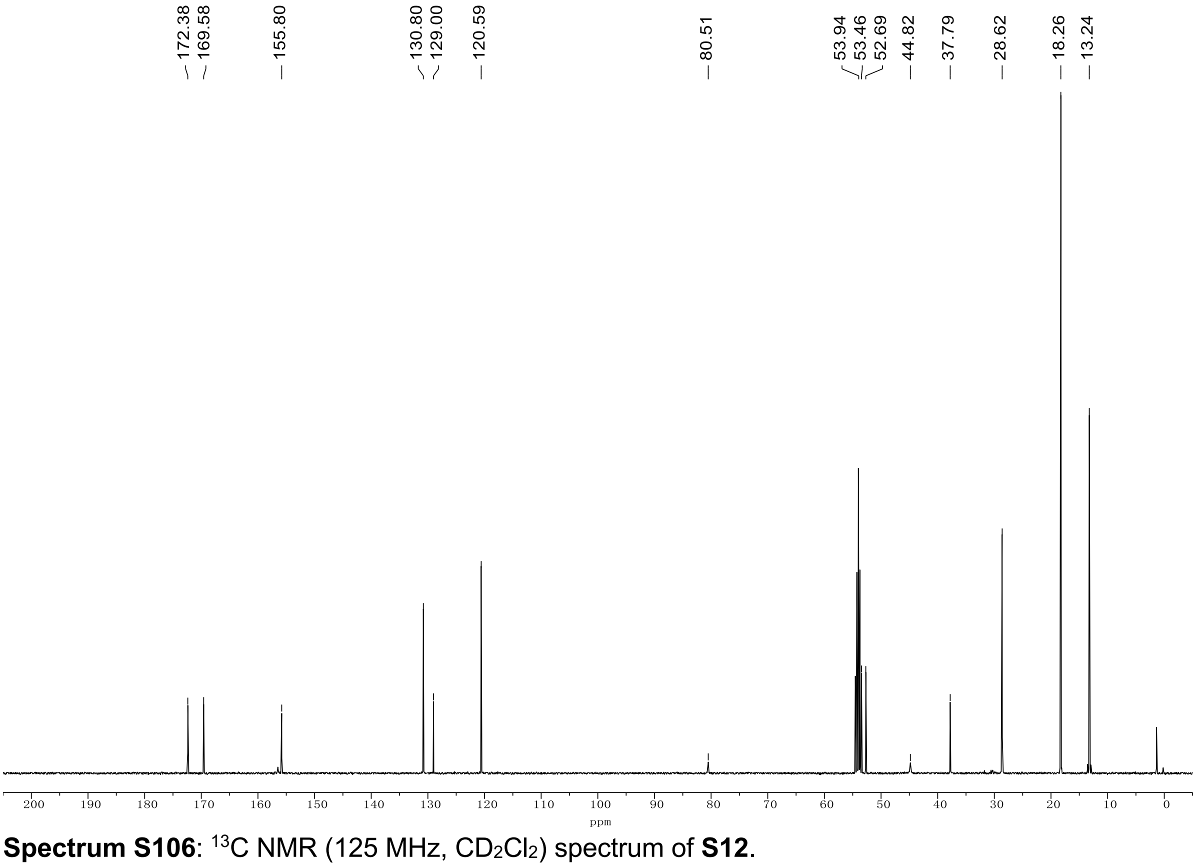

Spectrum S106: ^{13}C NMR (125 MHz, CD_2Cl_2) spectrum of **S12**.

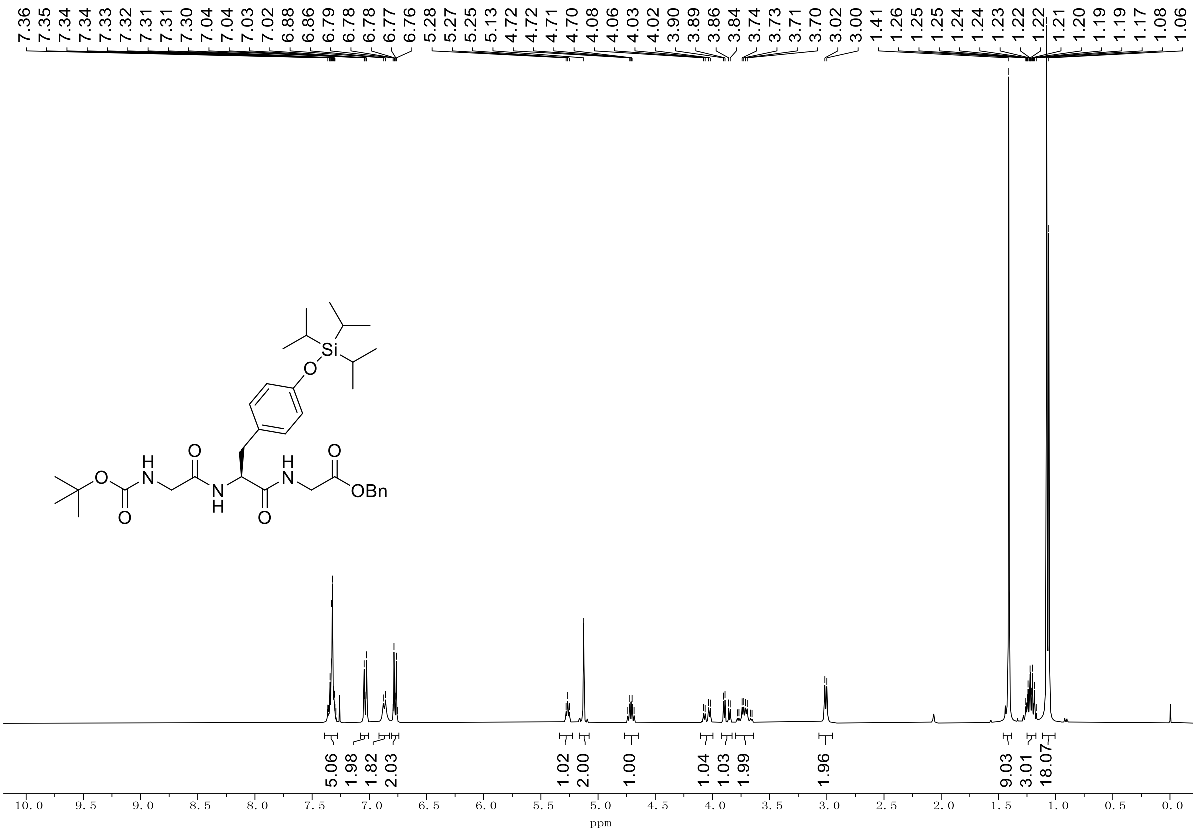

Spectrum S107: ^{1}H NMR (500 MHz, $CDCl_3$) spectrum of **S13**.

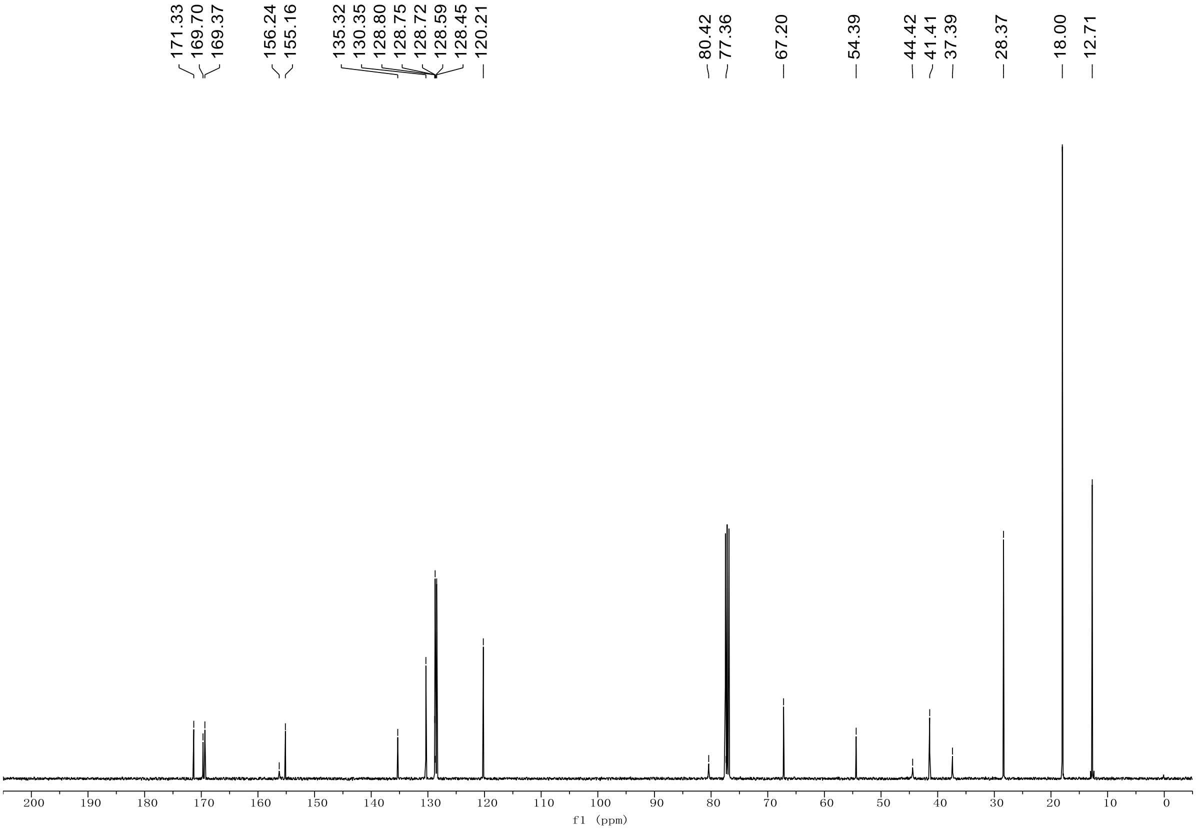

Spectrum S108: ^{13}C NMR (125 MHz, $CDCl_3$) spectrum of **S13**.

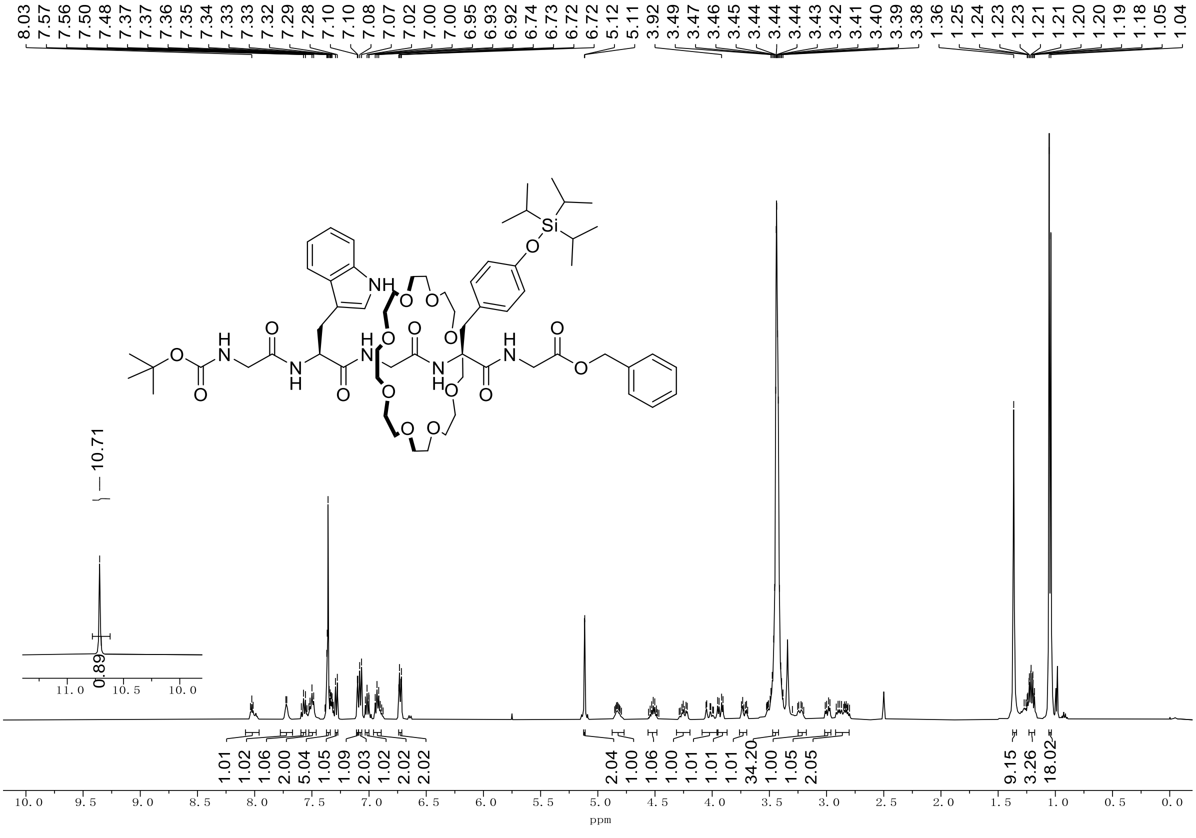

Spectrum S109: ^{1}H NMR (500 MHz, DMSO-*d*6) spectrum of **24C8⊂20**.

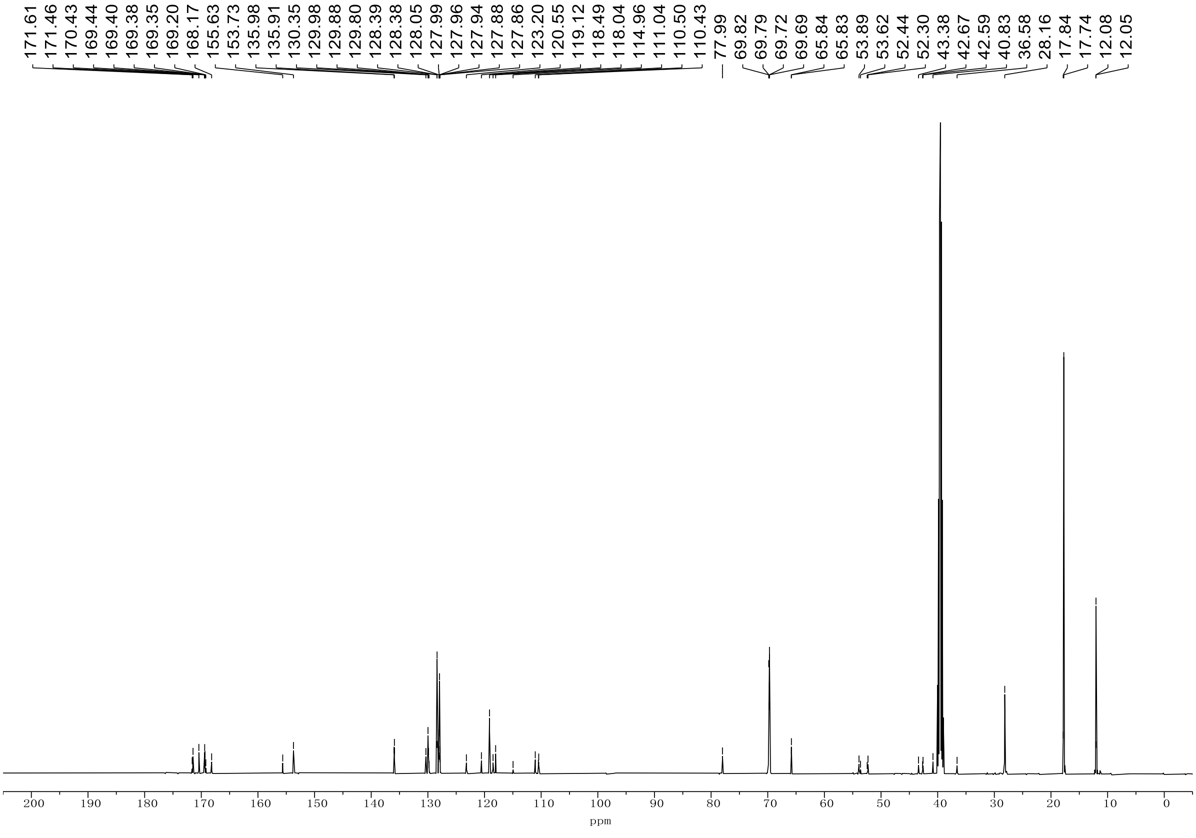

Spectrum S110: ^{13}C NMR (125 MHz, DMSO-*d*6) spectrum of **24C8⊂S20**.

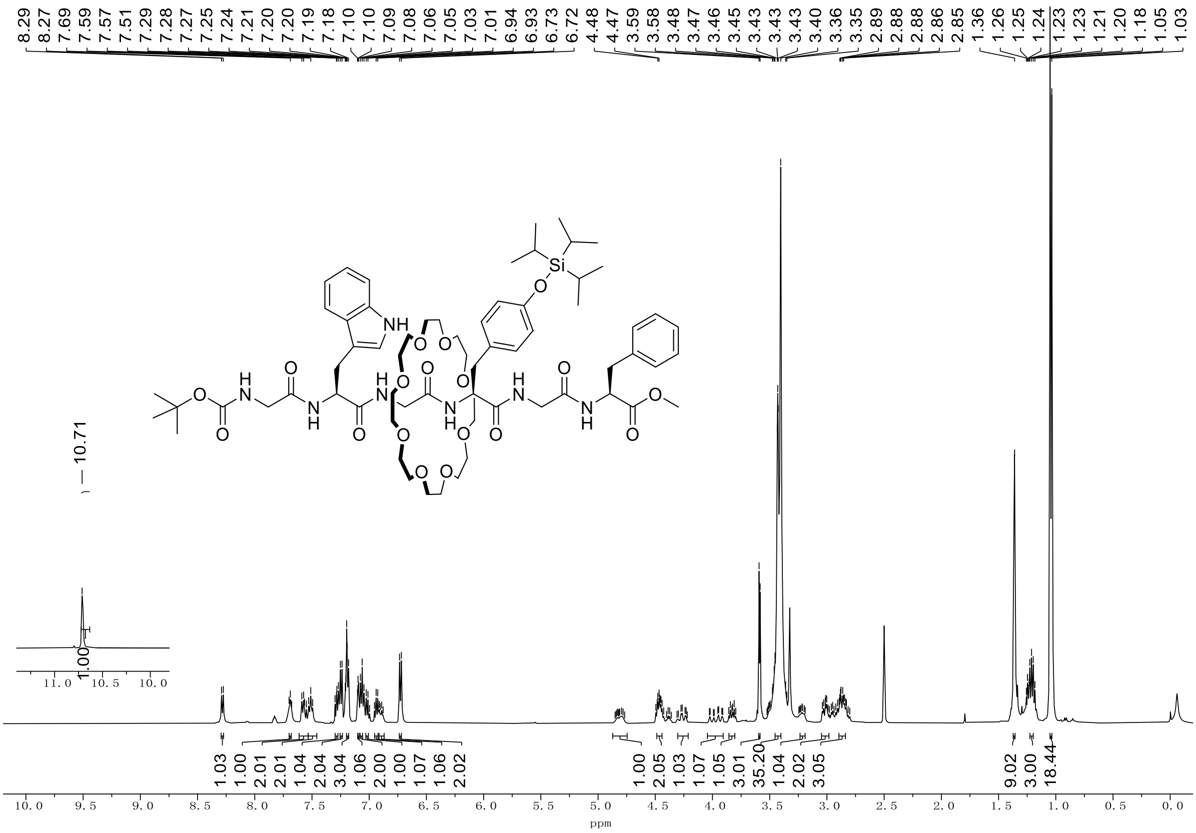

Spectrum S111: ^{1}H NMR (500 MHz, DMSO-d_6) spectrum of **24C8⊂S15**.

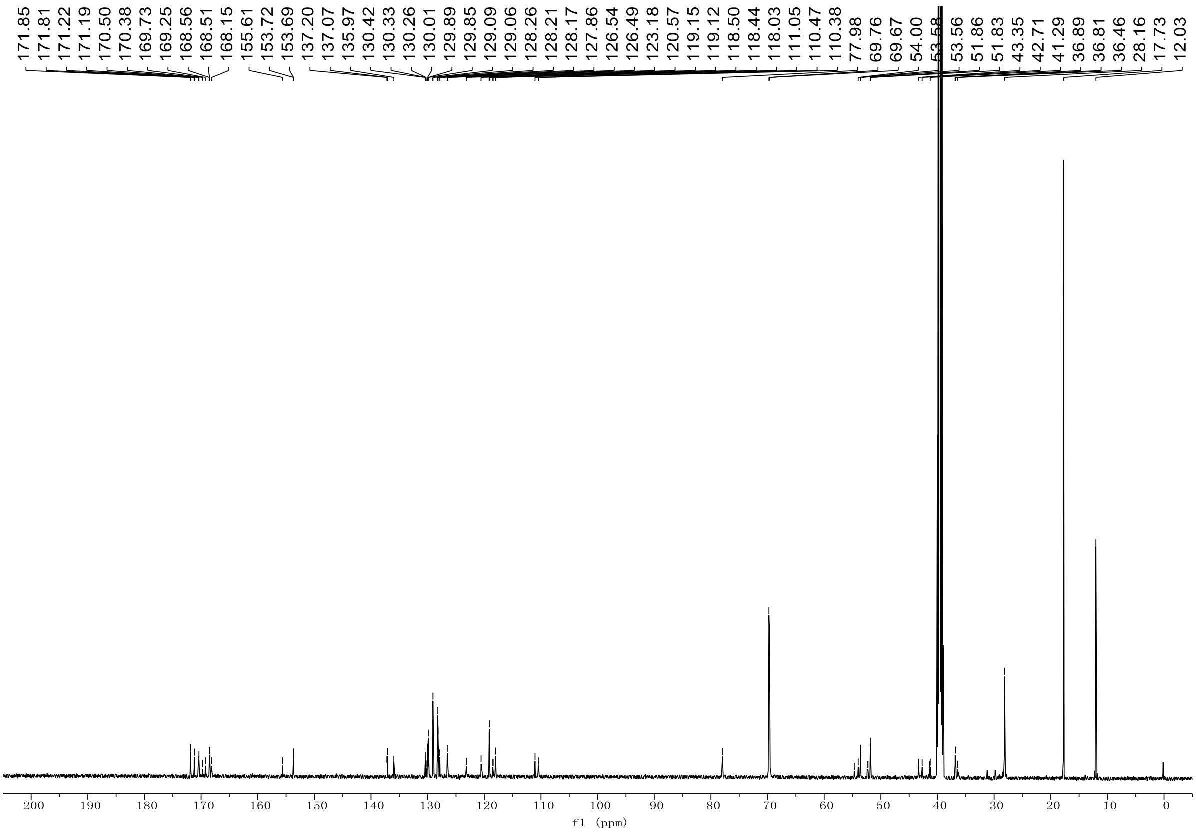

Spectrum S112: ^{13}C NMR (125 MHz, DMSO-d_6) spectrum of **24C8⊂S15**.

11. Coordinates of rotaxane 24C8⊂4 from molecular modelling studies

Center Number	Atomic Number	Atomic Type	Coordinates (Angstroms) X	Y	Z
1	8	0	-1.824703	0.355917	-1.641245
2	7	0	-1.385734	-1.362956	-0.134968
3	6	0	-1.219452	-0.690661	-1.300820
4	6	0	-0.047485	-1.205724	-2.134518
5	1	0	-0.072619	-0.728759	-3.111681
6	1	0	-0.083412	-2.284583	-2.249364
7	6	0	-2.128294	-0.919375	1.043937
8	1	0	-1.559604	-1.341275	1.885236
9	1	0	-0.954770	-2.285667	-0.078606
10	8	0	-2.537655	-3.044853	-2.500275
11	8	0	-0.324830	-3.994419	-0.606031
12	8	0	0.604661	2.493811	0.912285
13	8	0	1.787404	0.252414	2.352096
14	8	0	1.345922	-2.471076	1.145938
15	8	0	-1.977778	3.644694	-0.244747
16	8	0	-4.348447	1.981844	-1.544235
17	6	0	-4.703744	0.919770	-2.477832
18	1	0	-3.791508	0.447034	-2.852038
19	1	0	-5.259527	1.362751	-3.320695
20	6	0	-3.866956	-2.736018	-3.005445
21	1	0	-4.206278	-3.567689	-3.645415
22	1	0	-3.729994	-1.850026	-3.628589
23	6	0	1.513386	2.656412	2.034774
24	1	0	1.274526	3.582569	2.576649
25	1	0	2.550434	2.725309	1.678231
26	6	0	1.254324	-0.917506	3.035973
27	1	0	0.164337	-0.931958	2.936959
28	1	0	1.500218	-0.854086	4.106095
29	6	0	-2.451815	-4.292467	-1.745177
30	1	0	-3.031857	-5.071014	-2.263355
31	1	0	-2.847040	-4.152478	-0.735151
32	6	0	1.326102	1.494320	2.975634
33	1	0	1.895527	1.663663	3.900153
34	1	0	0.264877	1.399594	3.221975
35	6	0	1.640926	-3.853013	0.779711
36	1	0	1.164412	-4.523492	1.507150
37	1	0	2.724709	-4.012736	0.805170
38	6	0	0.407217	3.722683	0.143943
39	1	0	1.343976	3.995829	-0.347431
40	1	0	0.108395	4.534860	0.820078
41	6	0	1.124956	-4.153047	-0.604440
42	1	0	1.575634	-3.488464	-1.350740
43	1	0	1.386261	-5.189442	-0.856360
44	6	0	-1.003561	-4.724586	-1.679257
45	1	0	-0.937410	-5.798609	-1.460524
46	1	0	-0.512144	-4.527643	-2.640191
47	6	0	-0.672774	3.497207	-0.886639
48	1	0	-0.568793	4.257396	-1.675025
49	1	0	-0.580128	2.503796	-1.336211
50	6	0	-3.441642	2.936473	-2.161494
51	1	0	-3.954901	3.420393	-3.009589
52	1	0	-2.561475	2.408501	-2.541022
53	6	0	1.863556	-2.179092	2.484466
54	1	0	2.954301	-2.102620	2.436270
55	1	0	1.593290	-3.007911	3.153867
56	6	0	-3.053113	4.004606	-1.155197
57	1	0	-2.762733	4.916421	-1.700929
58	1	0	-3.908666	4.233013	-0.515857
59	8	0	-4.819653	-1.235599	-1.226672
60	6	0	-5.569046	-0.099815	-1.754957

61	1	0	-6.360898	-0.470477	-2.419574
62	1	0	-6.033795	0.389179	-0.893836
63	6	0	-4.937308	-2.494262	-1.944442
64	1	0	-4.890336	-3.263273	-1.171270
65	1	0	-5.922926	-2.550061	-2.430391
66	7	0	1.181668	-0.784166	-1.451510
67	1	0	1.337173	-1.135104	-0.506978
68	6	0	1.919632	0.258903	-1.907856
69	6	0	2.874971	0.877300	-0.879147
70	1	0	2.222241	1.513380	-0.270155
71	6	0	3.977388	1.750350	-1.538187
72	1	0	4.881975	1.647328	-0.931819
73	1	0	4.198221	1.351368	-2.529676
74	8	0	1.766045	0.764595	-3.050678
75	7	0	3.399195	-0.116804	0.055935
76	1	0	3.039604	-0.089684	1.010193
77	6	0	3.600045	3.214967	-1.623106
78	6	0	2.749824	3.692611	-2.636690
79	6	0	4.071513	4.122333	-0.658471
80	6	0	2.381936	5.041935	-2.679971
81	1	0	2.364578	2.986498	-3.362150
82	6	0	3.701192	5.472155	-0.697513
83	1	0	4.734459	3.766845	0.125654
84	6	0	2.853435	5.936775	-1.709950
85	1	0	1.725036	5.394635	-3.469074
86	1	0	4.074461	6.157148	0.057270
87	1	0	2.564833	6.982325	-1.743718
88	6	0	-3.481837	-1.623904	1.141891
89	8	0	-3.643407	-2.836343	0.931774
90	8	0	-4.464838	-0.819960	1.625803
91	6	0	-5.801097	-1.412823	1.774956
92	1	0	-6.399542	-0.635727	2.244763
93	1	0	-6.178658	-1.667351	0.784912
94	1	0	-5.749951	-2.305161	2.401834
95	6	0	-2.150623	0.605351	1.201201
96	1	0	-1.197765	0.988722	0.822672
97	1	0	-2.912597	1.041488	0.553853
98	6	0	-2.290466	1.126255	2.618841
99	6	0	-2.400970	0.315772	3.759515
100	6	0	-2.230986	2.523837	2.799187
101	6	0	-2.429261	0.876936	5.044467
102	1	0	-2.476149	-0.761667	3.664921
103	6	0	-2.261483	3.083924	4.078188
104	1	0	-2.144867	3.150772	1.917369
105	6	0	-2.354602	2.262303	5.211161
106	1	0	-2.513709	0.227697	5.910890
107	1	0	-2.210308	4.162726	4.194380
108	1	0	-2.374508	2.697069	6.205601
109	6	0	4.252824	-1.086580	-0.348050
110	8	0	4.575497	-1.929232	0.703934
111	6	0	5.752452	-2.869654	0.608366
112	6	0	7.015065	-2.050363	0.323340
113	1	0	6.960241	-1.585355	-0.662417
114	1	0	7.139867	-1.271221	1.082180
115	1	0	7.891667	-2.706055	0.354963
116	6	0	5.492189	-3.930380	-0.465847
117	1	0	4.570381	-4.479373	-0.250007
118	1	0	5.408084	-3.468180	-1.449144
119	1	0	6.320282	-4.647740	-0.473206
120	6	0	5.788032	-3.484703	2.007953
121	1	0	4.857874	-4.021688	2.217246
122	1	0	6.621263	-4.191095	2.080731
123	1	0	5.920448	-2.706415	2.765149
124	8	0	4.687116	-1.212040	-1.515625
